# Supplementary material for: Predictors of Adverse Pregnancy Outcomes in Pregnant Women Living with Obesity: A Systematic Review
Source: Int J Environ Res Public Health. 2022 Feb 12;19(4):2063. doi: 10.3390/ijerph19042063 (PMC8872310; doi:10.3390/ijerph19042063)
Supplement: Supplementary file 1 [file ijerph-19-02063-s001.zip › ijerph-1546324-supplementary.pdf]

Predictors of Adverse Pregnancy Outcomes in Pregnant Women Living with Obesity: A Systematic Review

File S1: Database Search Strategies

Database: Embase Classic+Embase <1947 to 2020 March 05>, Ovid MEDLINE(R) ALL <1946 to March 05, 2020>, PsycINFO <1806 to March Week 1 2020>, Joanna Briggs Institute EBP Database - <Current to February 26, 2020>, EBM Reviews - Cochrane Central Register of Controlled Trials <January 2020>, EBM Reviews - Cochrane Database of Systematic Reviews <2005 to March 4, 2020>
Search Strategy:

----

1 Pregnancy/ (1629651)
2 exp Pregnancy Complications/ (574252)
3 Pregnant Women/ (78249)
4 exp Pregnancy Trimesters/ (835936)
5 pregnan*.tw,kw. (1320762)
6 Prenatal Care/ (70527)
7 (prenatal* or antenatal* or ante natal* or antepartum or ante partum).tw,kw. (356488)
8 or/1-7 [PREGNANCY] (2313745)
9 exp Overweight/ (773480)
10 (obesity* or obese or overweight or over weight or adiposit*).tw,kw. (899327)
11 (body adj2 (size* or shape* or weight* or fat* or silhouette*)).tw,kw. (714530)
12 exp Adipose Tissue/ (271847)
13 exp Body Fat Distribution/ (21902)
14 ((abdominal* or central* or subcutaneous*) adj2 (obes* or fat* or adipos*)).tw,kw. (100944)
15 Anthropometry/ (97255)
16 Waist Circumference/ or Waist-Hip Ratio/ or Waist-Height Ratio/ (74903)
17 Skinfold Thickness/ (20075)
18 (anthropometr* or BMI or BMIs or skinfold* or SFT or SFTs).tw,kw. (614662)
19 (waist* adj2 (circumference* or hip* or height or thigh*)).tw,kw. (105266)
20 (waisthip or waistheight or waistthigh or WHR or WHtR or WTR).tw,kw. (16319)
21 or/9-20 [ADIPOSITY] (2155529)
22 8 and 21 [ADIPOSITY IN PREGNANCY] (127128)
23 exp Animals/ not (exp Animals/ and Humans/) (18145733)
24 22 not 23 [ANIMAL-ONLY REMOVED] (72726)
25 (comment or editorial or interview or news or newspaper article).pt. (2066673)
26 (letter not (letter and randomized controlled trial)).pt. (2164882)
27 24 not (25 or 26) [OPINION PIECES REMOVED] (71150)
28 exp Risk/ (3665410)
29 (risk or risked or risks or risky or risking or risk-related).tw,kw. (5946921)
30 predict*.tw,kw. (4167721)
31 logistic*.tw,kw. (856074)
32 (logit* adj1 model*).tw,kw. (6575)
33 Prevalence/ (990248)
34 prevalen*.tw,kw. (1984134)
35 Pregnancy Complications/ep [epidemiology] (12669)
36 or/28-35 [RISK/PREDICTION] (11650984)

37 27 and 36 (39093)
38 Pregnancy Outcome/ (110912)
39 ((pregnan* or prenatal* or antenatal* or ante natal* or antepartum or ante partum or perinatal* or peripartum) adj3 outcome*).tw,kw. (118174)
40 ((maternal* or mother* or baby or babies or f?etal* or f?etus* or neonat* or newborn*) adj3 outcome*).tw,kw. (88341)
41 exp Pregnancy Complications/mo [mortality] (6721)
42 Maternal Mortality/ (33772)
43 Fetal Mortality/ (5032)
44 Perinatal Mortality/ (16632)
45 (mortalit* or death* or fatal*).tw,kw. (3957410)
46 exp Fetal Death/ (74852)
47 ((baby or babies or f?etal or f?etus* or neonat* or newborn*) adj3 (dead or demise? or died or dying)).tw,kw. (18096)
48 (stillbirth* or stillborn*).tw,kw. (39507)
49 exp Abortion, Spontaneous/ (80320)
50 ((abort* adj2 spontaneous*) or miscarriage? or (recur* adj2 loss*) or (habit* adj2 abort*)).tw,kw. (75747)
51 exp Hospitalization/ (639998)
52 hospitali*.tw,kw. (746337)
53 ((admit* or admission* or readmit* or readmission*) adj3 (hospital? or critical care or intensive care or ICU or ICUs or NICU or NICUs or SICU or SICUs)).tw,kw. (389590)
54 exp Hypertension, Pregnancy-Induced/ (55111)
55 ((eclamp* or hypertensi* or preeclamp* or pre-eclamp* or toxemi* or toxaemi* or EPH or hemolys#s) adj3 (gestational* or maternal* or "new onset" or pregnancy-induced)).tw,kw. (31785)
56 PIH.tw,kw. (5310)
57 HELLP.tw,kw. (6878)
58 exp Diabetes, Gestational/ (49429)
59 ((diabet* or DM or T2DM) adj3 (gestational* or maternal* or "new onset" or pregnancy-induced)).tw,kw. (57931)
60 PID.tw,kw. (13056)
61 macrosomi*.tw,kw. (11229)
62 Venous Thromboembolism/ (47161)
63 (DVT or thromboemboli* or VTE).tw,kw. (188930)
64 exp Placenta Diseases/ (45964)
65 Placental Circulation/ (7099)
66 (placenta* adj3 (abnormal* or disease* or disorder* or dysfunction*)).tw,kw. (13087)
67 (placenta* adj2 mediate*).tw,kw. (1149)
68 Fetal Growth Retardation/ (33493)
69 ((f?etal or f?etus* or intrauterin*) adj grow* adj3 (restrict* or retard*)).tw,kw. (44703)
70 (FGR or IUGR).tw,kw. (20519)
71 Premature Birth/ (81916)
72 (preterm or prematur*).tw,kw. (532367)
73 exp Cesarean Section/ (149360)
74 (cesarean* or caesarean*).tw,kw. (164121)

75 (C-section* or Csection*).tw,kw. (5413)
76 (abdom* adj3 deliver*).tw,kw. (2426)
77 (postdate* or post-date* or postmatur* or post-matur* or postterm* or post-term*).tw,kw. (9116)
78 (42 week? or 43 week?).tw,kw. (11165)
79 ("42 0/7" or "42 1/7" or "42 2/7" or "42 3/7" or "42 4/7" or "42 5/7" or "42 6/7" or "42 7/7" or "43 0/7" or "43 1/7" or "43 2/7" or "43 3/7" or "43 4/7" or "43 5/7" or "43 6/7" or "43 7/7").tw,kw. (670)
80 Fetal Macrosomia/ (6974)
81 ("large-for-gestational age" or LGA).tw,kw. (9227)
82 Congenital Abnormalities/ (46941)
83 ((birth or congenital) adj3 (abnormal* or anomal* or defect* or deform*)).tw,kw. (159952)
84 exp Neural Tube Defects/ (61770)
85 (NTD or NTDs).tw,kw. (10633)
86 (neural tube? adj2 (defect* or deform* or malform*)).tw,kw. (18102)
87 (acrani* or craniorachischis* or diastematomyeli* or exencephal* or iniencephal* or neurenteric cyst* or neuroenteric cyst* or occult spinal dysraphism* or spinal cord myelodysplasi* or (tethered adj2 cord syndrome*)).tw,kw. (7305)
88 (anencephal* or aprosencephal*).tw,kw. (7561)
89 (congenital*.tw,kw. or cn.fs.) and ((absen* or lack* or missing) adj2 brain*).tw,kw. (122)
90 (congenital*.tw,kw. or cn.fs.) and ((absen* or lack* or missing) adj2 crani$2 vault*).tw,kw. (10)
91 (meroanencephal* or holoanencephal*).tw,kw. (15)
92 (encephalocele or bifid cranium or cephalocele or cerebellar hernia* or cerebral hernia* or cranial meningoencephalocele or craniocele or cranium bifidum or notoencephalocelecranial or tonsillar hernia*).tw,kw. (8218)
93 (rachischisis or schistorrhachis or status dysraphicus).tw,kw. (483)
94 ((cleft or open) adj1 (spine? or spinal)).tw,kw. (570)
95 (spina? adj (bifida? or bifidum? or dysraphia* or dysraphism*)).tw,kw. (21692)
96 dermal sinus*.tw,kw. (1246)
97 (Arnold-Chiari adj1 (syndrome* or malform* or deform*)).tw,kw. (2233)
98 Chiari malformation?.tw,kw. (6198)
99 (Cantrell* adj1 pentalog*).tw,kw. (171)
100 ((Cantrell Haller Ravitch or thoracoabdominal) adj syndrome*).tw,kw. (21)
101 exp Cardiovascular Abnormalities/ (416346)
102 ((heart or cardiac* or cardio* or aort* or arter* or ventric* or vascular*) adj3 (abnormal* or anomal* or defect* or deform* or malform*)).tw,kw. (277461)
103 ((septa? or septum) adj3 (abnormal* or anomal* or defect* or deform* or malform*)).tw,kw. (66688)
104 Cleft Lip/ (32086)
105 Cleft Palate/ (47211)
106 (cleft? adj1 (lip or lips or palat*)).tw,kw. (50862)
107 (congenital fissur* adj1 (lip or lips or palat*)).tw,kw. (0)
108 ((orofacial or oro facial) adj1 cleft?).tw,kw. (2498)
109 (labioschis* or cheiloschis* or abioschiz*).tw,kw. (71)
110 (harelip? or hare lip?).tw,kw. (1679)

111 (palatischis* or palatoschis* or palatoschiz* or palatum fissu*).tw,kw. (324)
112 Anorectal Malformations/ (3182)
113 ((anorectal* or ano-rectal*) adj3 (abnormal* or anomal* or atresia* or defect* or deform* or malform* or stenos*)).tw,kw. (6497)
114 ((anus$2 or anal$2) adj3 (abnormal* or anomal* or atresia* or defect* or deform* or malform* or stenos*)).tw,kw. (6133)
115 ((rectum? or rectal$2) adj3 (abnormal* or anomal* or atresia* or defect* or deform* or malform* or stenos*)).tw,kw. (4548)
116 Hydrocephalus/ (63958)
117 hydrocephal*.tw,kw. (69111)
118 (aqueductal stenos* or cerebral ventriculomegal* or Dandy-Walker or Luschka-Magendie foramina atresia* or Hakim$2).tw,kw. (6345)
119 exp Limb Deformities, Congenital/ (73214)
120 ((arm or arms or carpal or carpus or femur* or finger? or foot or feet or fibula? or forearm? or fore arm? or hand or hands or hip or hips or humeral or humerus or knee? or kneecap? or leg or legs or metacarp* or metatars* or patell* or radius or radial or talipedes or talipes or tarsal or tibia? or toe or toes or ulna? or wrist?) adj3 (abnormal* or anomal* or defect* or deform* or malform* or reduc*)).tw,kw. (107044)
121 ((limb or limbs) adj3 (abnormal* or anomal* or defect* or deform* or malform* or reduc*)).tw,kw. (21351)
122 (extremit* adj3 (abnormal* or anomal* or defect* or deform* or malform* or reduc*)).tw,kw. (6090)
123 (clubfoot or club foot or clubfeet or club feet).tw,kw. (7899)
124 (flatfoot or flat foot or flatfeet or flat feet).tw,kw. (5316)
125 (arachnodactyl* or brachydactyl* or ectromelia* or amelia* or hemimelia* or phocomelia* or sirenomelia* or hyperdactyl* or polydactyl* or polysyndactyl* or syndactyl*).tw,kw. (23028)
126 Gastroschisis/ (4995)
127 gastroschis*.tw,kw. (5672)
128 or/38-127 [OUTCOMES] (6964826)
129 37 and 128 (24104)
130 limit 129 to english [Limit not valid in Joanna Briggs Institute EBP Database -; records were retained] (22120)
131 130 use medall [MEDLINE RECORDS] (10591)
132 (2019* or 2020*).dt. (1530388)
133 131 and 132 [MEDLINE UPDATE] (1107)
134 pregnancy/ (1629651)
135 exp pregnancy complication/ (574252)
136 pregnant woman/ (91405)
137 first trimester pregnancy/ or second trimester pregnancy/ or third trimester pregnancy/ (117954)
138 pregnan*.tw,kw. (1320762)
139 prenatal care/ (70527)
140 (prenatal* or antenatal* or ante natal* or antepartum or ante partum).tw,kw. (356488)
141 or/134-140 [PREGNANCY] (2307012)
142 exp obesity/ (764280)

143 (obesity* or obese or overweight or over weight or adiposit*).tw,kw. (899327)
144 (body adj2 (size* or shape* or weight* or fat* or silhouette*)).tw,kw. (714530)
145 exp adipose tissue/ (271847)
146 ((abdominal* or central* or subcutaneous*) adj2 (obes* or fat* or adipos*)).tw,kw. (100944)
147 anthropometry/ (97255)
148 waist circumference/ or waist hip ratio/ or waist to height ratio/ or weight height ratio/ (75217)
149 skindfold thickness/ (0)
150 (anthropometr* or BMI or BMIs or skinfold* or SFT or SFTs).tw,kw. (614662)
151 (waist* adj2 (circumference* or hip* or height or thigh*)).tw,kw. (105266)
152 (waisthip or waistheight or waistthigh or WHR or WHtR or WTR).tw,kw. (16319)
153 or/142-152 [OBESITY/OVERWEIGHT/ADIPOSITY] (2148879)
154 141 and 153 [OBESITY/OVERWEIGHT/ADIPOSITY IN PREGNANCY] (126692)
155 exp animal experimentation/ or exp models animal/ or exp animal experiment/ or nonhuman/ or exp vertebrate/ (51893912)
156 exp human/ or exp human experimentation/ or exp human experiment/ (40882061)
157 155 not 156 (11013592)
158 154 not 157 [ANIMAL-ONLY REMOVED] (100516)
159 editorial.pt. (1164184)
160 letter.pt. not (letter.pt. and randomized controlled trial/) (2164763)
161 158 not (159 or 160) [OPINION PIECES REMOVED] (98523)
162 risk/ (644320)
163 risk assessment/ (828736)
164 risk factor/ (1894960)
165 (risk or risked or risks or risky or risking or risk-related).tw,kw. (5946921)
166 predict*.tw,kw. (4167721)
167 logistic*.tw,kw. (856074)
168 (logit* adj1 model*).tw,kw. (6575)
169 prevalence/ (990248)
170 prevalen*.tw,kw. (1984134)
171 exp pregnancy complication/ep [Epidemiology] (60810)
172 or/162-171 [RISK] (11452680)
173 161 and 172 (56594)
174 pregnancy outcome/ (110912)
175 ((pregnan* or prenatal* or antenatal* or ante natal* or antepartum or ante partum or perinatal* or peripartum) adj3 outcome*).tw,kw. (118174)
176 maternal mortality/ (33772)
177 fetus mortality/ (4534)
178 exp perinatal mortality/ (29820)
179 prenatal mortality/ (345)
180 (mortalit* or death* or fatal*).tw,kw. (3957410)
181 exp fetus death/ (45530)
182 ((baby or babies or f?etal or f?etus* or neonat* or newborn*) adj3 (dead or demise? or died or dying)).tw,kw. (18096)
183 (stillbirth* or stillborn*).tw,kw. (39507)

184 abortion, spontaneous/ (45374)
185 ((abort* adj2 spontaneous*) or miscarriage? or (recur* adj2 loss*) or (habit* adj2 abort*)).tw,kw. (75747)
186 exp hospitalization/ (639998)
187 hospital admission/ (193931)
188 hospitali*.tw,kw. (746337)
189 ((admit* or admission* or readmit* or readmission*) adj3 (hospital? or critical care or intensive care or ICU or ICUs or NICU or NICUs or SICU or SICUs)).tw,kw. (389590)
190 maternal hypertension/ (18121)
191 exp "eclampsia and preeclampsia"/ (63983)
192 ((eclamp* or hypertensi* or preeclamp* or pre-eclamp* or toxemi* or toxaemi* or EPH or hemolys#s) adj3 (gestational* or maternal* or "new onset" or pregnancy-induced)).tw,kw. (31785)
193 PIH.tw,kw. (5310)
194 HELLP syndrome/ (6864)
195 HELLP.tw,kw. (6878)
196 exp pregnancy diabetes mellitus/ (36439)
197 ((diabet* or DM or T2DM) adj3 (gestational* or maternal* or "new onset" or pregnancy-induced)).tw,kw. (57931)
198 PID.tw,kw. (13056)
199 macrosomia/ (6748)
200 macrosomi*.tw,kw. (11229)
201 exp venous thromboembolism/ (168467)
202 (DVT or thromboemboli* or VTE).tw,kw. (188930)
203 exp placenta disorder/ (45964)
204 (placenta* adj3 (abnormal* or disease* or disorder* or dysfunction*)).tw,kw. (13087)
205 (placenta* adj2 mediate*).tw,kw. (1149)
206 exp intrauterine growth retardation/ (59802)
207 ((f?etal or f?etus* or intrauterin*) adj grow* adj3 (restrict* or retard*)).tw,kw. (44703)
208 (FGR or IUGR).tw,kw. (20519)
209 preterm birth/ (61411)
210 (preterm or prematur*).tw,kw. (532367)
211 exp cesarean section/ (149360)
212 (cesarean* or caesarean*).tw,kw. (164121)
213 (C-section* or Csection*).tw,kw. (5413)
214 (abdom* adj3 deliver*).tw,kw. (2426)
215 postmaturity/ (990)
216 (postdate* or post-date* or postmatur* or post-matur* or postterm* or post-term*).tw,kw. (9116)
217 (42 week? or 43 week?).tw,kw. (11165)
218 ("42 0/7" or "42 1/7" or "42 2/7" or "42 3/7" or "42 4/7" or "42 5/7" or "42 6/7" or "42 7/7" or "43 0/7" or "43 1/7" or "43 2/7" or "43 3/7" or "43 4/7" or "43 5/7" or "43 6/7" or "43 7/7").tw,kw. (670)
219 large for gestational age/ (3136)
220 ("large-for-gestational age" or LGA).tw,kw. (9227)
221 congenital disorder/ (87367)

222 ((birth or congenital) adj3 (abnormal* or anomal* or defect* or deform*)).tw,kw. (159952)
223 neural tube defect/ (22510)
224 (NTD or NTDs).tw,kw. (10633)
225 (neural tube? adj2 (defect* or deform* or malform*)).tw,kw. (18102)
226 (acrani* or craniorachischis* or diastematomyeli* or exencephal* or iniencephal* or neurenteric cyst* or neuroenteric cyst* or occult spinal dysraphism* or spinal cord myelodysplasi* or (tethered adj2 cord syndrome*)).tw,kw. (7305)
227 (anencephal* or aprosencephal*).tw,kw. (7561)
228 (congenital*.tw,kw. or cn.fs.) and ((absen* or lack* or missing) adj2 brain*).tw,kw. (122)
229 (congenital*.tw,kw. or cn.fs.) and ((absen* or lack* or missing) adj2 crani$2 vault*).tw,kw. (10)
230 (meroanencephal* or holoanencephal*).tw,kw. (15)
231 (encephalocele or bifid cranium or cephalocele or cerebellar hernia* or cerebral hernia* or cranial meningoencephalocele or craniocele or cranium bifidum or notoencephalocelecranial or tonsillar hernia*).tw,kw. (8218)
232 (rachischisis or schistorrhachis or status dysraphicus).tw,kw. (483)
233 ((cleft or open) adj1 (spine? or spinal)).tw,kw. (570)
234 (spina? adj (bifida? or bifidum? or dysraphia* or dysraphism*)).tw,kw. (21692)
235 dermal sinus*.tw,kw. (1246)
236 (Arnold-Chiari adj1 (syndrome* or malform* or deform*)).tw,kw. (2233)
237 Chiari malformation?.tw,kw. (6198)
238 (Cantrell* adj1 pentalog*).tw,kw. (171)
239 ((Cantrell Haller Ravitch or thoracoabdominal) adj syndrome*).tw,kw. (21)
240 exp cardiovascular malformation/ (220636)
241 ((heart or cardiac* or cardio* or aort* or arter* or ventric* or vascular*) adj3 (abnormal* or anomal* or defect* or deform* or malform*)).tw,kw. (277461)
242 ((septa? or septum) adj3 (abnormal* or anomal* or defect* or deform* or malform*)).tw,kw. (66688)
243 cleft lip/ (32086)
244 cleft palate/ (47211)
245 (cleft? adj1 (lip or lips or palat*)).tw,kw. (50862)
246 (congenital fissur* adj1 (lip or lips or palat*)).tw,kw. (0)
247 ((orofacial or oro facial) adj1 cleft?).tw,kw. (2498)
248 (abioschiz* or cheiloschis* or labioschis*).tw,kw. (71)
249 (harelip? or hare lip?).tw,kw. (1679)
250 (palatischis* or palatoschis* or palatoschiz* or palatum fissu*).tw,kw. (324)
251 exp anorectal malformation/ (7654)
252 ((anorectal* or ano-rectal*) adj3 (abnormal* or anomal* or atresia* or defect* or deform* or malform* or stenos*)).tw,kw. (6497)
253 ((anus$2 or anal$2) adj3 (abnormal* or anomal* or atresia* or defect* or deform* or malform* or stenos*)).tw,kw. (6133)
254 ((rectum? or rectal$2) adj3 (abnormal* or anomal* or atresia* or defect* or deform* or malform* or stenos*)).tw,kw. (4548)
255 congenital hydrocephalus/ (22519)
256 hydrocephal*.tw,kw. (69111)

257 (aqueductal stenos* or cerebral ventriculomegal* or Dandy-Walker or Luschka-Magendie foramina atresia* or Hakim$2).tw,kw. (6345)
258 exp limb malformation/ (49459)
259 ((arm or arms or carpal or carpus or femur* or finger? or foot or feet or fibula? or forearm? or fore arm? or hand or hands or hip or hips or humeral or humerus or knee? or kneecap? or leg or legs or metacarp* or metatars* or patell* or radius or radial or talipedes or talipes or tarsal or tibia? or toe or toes or ulna? or wrist?) adj3 (abnormal* or anomal* or defect* or deform* or malform* or reduc*)).tw,kw. (107044)
260 ((limb or limbs) adj3 (abnormal* or anomal* or defect* or deform* or malform* or reduc*)).tw,kw. (21351)
261 (extremit* adj3 (abnormal* or anomal* or defect* or deform* or malform* or reduc*)).tw,kw. (6090)
262 (clubfoot or club foot or clubfeet or club feet).tw,kw. (7899)
263 (flatfoot or flat foot or flatfeet or flat feet).tw,kw. (5316)
264 (arachnodactyl* or brachydactyl* or ectromelia* or amelia* or hemimelia* or phocomelia* or sirenomelia* or hyperdactyl* or polydactyl* or polysyndactyl* or syndactyl*).tw,kw. (23028)
265 gastroschisis/ (4995)
266 gastroschis*.tw,kw. (5672)
267 or/174-266 [OUTCOMES] (6951111)
268 173 and 267 (35396)
269 conference abstract.pt. (3736394)
270 268 not 269 [CONFERENCE ABSTRACTS REMOVED] (27452)
271 limit 270 to english [Limit not valid in Joanna Briggs Institute EBP Database -; records were retained] (25023)
272 271 use emczd [EMBASE RECORDS] (12372)
273 (2019* or 2020*).dc. (2435396)
274 272 and 273 [EMBASE UPDATE] (1776)
275 exp Pregnancy/ (1738327)
276 pregnan*.tw,kw. (1320762)
277 exp Prenatal Care/ (183803)
278 (prenatal* or antenatal* or ante natal* or antepartum or ante partum).tw,kw. (356488)
279 or/275-278 [PREGNANCY] (2312861)
280 exp Overweight/ (773480)
281 (obesity* or obese or overweight or over weight or adiposit*).tw,kw. (899327)
282 (body adj2 (size* or shape* or weight* or fat* or silhouette*)).tw,kw. (714530)
283 Body Fat/ (119717)
284 ((abdominal* or central* or subcutaneous*) adj2 (obes* or fat* or adipos*)).tw,kw. (100944)
285 Anthropometry/ (97255)
286 (anthropometr* or BMI or BMIs or skinfold* or SFT or SFTs).tw,kw. (614662)
287 (waist* adj2 (circumference* or hip* or height or thigh*)).tw,kw. (105266)
288 (waisthip or waistheight or waistthigh or WHR or WHtR or WTR).tw,kw. (16319)
289 or/280-288 [ADIPOSITY] (2072808)
290 279 and 289 [ADIPOSITY IN PREGNANCY] (122676)
291 exp Animals/ not (exp Animals/ and Humans/) (18145733)

292 290 not 291 [ANIMAL-ONLY REMOVED] (70979)
293 Risk Factors/ (1530338)
294 Risk Assessment/ (828736)
295 Predisposition/ (97318)
296 (risk or risked or risks or risky or risking or risk-related).tw,kw. (5946921)
297 predict*.tw,kw. (4167721)
298 logistic*.tw,kw. (856074)
299 (logit* adj1 model*).tw,kw. (6575)
300 prevalen*.tw,kw. (1984134)
301 or/293-300 [RISK/PREDICTION] (11243979)
302 292 and 301 (38222)
303 Pregnancy Outcomes/ (53746)
304 ((pregnan* or prenatal* or antenatal* or ante natal* or antepartum or ante partum or perinatal* or peripartum) adj3 outcome*).tw,kw. (118174)
305 ((maternal* or mother* or baby or babies or f?etal* or f?etus* or neonat* or newborn*) adj3 outcome*).tw,kw. (88341)
306 Mortality Rate/ (101821)
307 (mortalit* or death* or fatal*).tw,kw. (3957410)
308 ((baby or babies or f?etal or f?etus* or neonat* or newborn*) adj3 (dead or demise? or died or dying)).tw,kw. (18096)
309 (stillbirth* or stillborn*).tw,kw. (39507)
310 Spontaneous Abortion/ (64685)
311 ((abort* adj2 spontaneous*) or miscarriage? or (recur* adj2 loss*) or (habit* adj2 abort*)).tw,kw. (75747)
312 exp Hospitalization/ (639998)
313 hospitali*.tw,kw. (746337)
314 ((admit* or admission* or readmit* or readmission*) adj3 (hospital? or critical care or intensive care or ICU or ICUs or NICU or NICUs or SICU or SICUs)).tw,kw. (389590)
315 Preeclampsia/ (89401)
316 ((eclamp* or hypertensi* or preeclamp* or pre-eclamp* or toxemi* or toxaemi* or EPH or hemolys#s) adj3 (gestational* or maternal* or "new onset" or pregnancy-induced)).tw,kw. (31785)
317 PIH.tw,kw. (5310)
318 HELLP.tw,kw. (6878)
319 Gestational Diabetes/ (43167)
320 ((diabet* or DM or T2DM) adj3 (gestational* or maternal* or "new onset" or pregnancy-induced)).tw,kw. (57931)
321 PID.tw,kw. (13056)
322 macrosomi*.tw,kw. (11229)
323 Embolisms/ (14060)
324 (DVT or thromboemboli* or VTE).tw,kw. (188930)
325 (placenta* adj3 (abnormal* or disease* or disorder* or dysfunction*)).tw,kw. (13087)
326 (placenta* adj2 mediate*).tw,kw. (1149)
327 ((f?etal or f?etus* or intrauterin*) adj grow* adj3 (restrict* or retard*)).tw,kw. (44703)
328 (FGR or IUGR).tw,kw. (20519)
329 Premature Birth/ (81916)

330 (preterm or prematur*).tw,kw. (532367)
331 Caesarean Birth/ (101937)
332 (cesarean* or caesarean*).tw,kw. (164121)
333 (C-section* or Csection*).tw,kw. (5413)
334 (abdom* adj3 deliver*).tw,kw. (2426)
335 (postdate* or post-date* or postmatur* or post-matur* or postterm* or post-term*).tw,kw. (9116)
336 (42 week? or 43 week?).tw,kw. (11165)
337 ("42 0/7" or "42 1/7" or "42 2/7" or "42 3/7" or "42 4/7" or "42 5/7" or "42 6/7" or "42 7/7" or "43 0/7" or "43 1/7" or "43 2/7" or "43 3/7" or "43 4/7" or "43 5/7" or "43 6/7" or "43 7/7").tw,kw. (670)
338 ("large-for-gestational age" or LGA).tw,kw. (9227)
339 Congenital Disorders/ (87367)
340 ((birth or congenital) adj3 (abnormal* or anomal* or defect* or deform*)).tw,kw. (159952)
341 (NTD or NTDs).tw,kw. (10633)
342 (neural tube? adj2 (defect* or deform* or malform*)).tw,kw. (18102)
343 (acrani* or craniorachischis* or diastematomyeli* or exencephal* or iniencephal* or neurenteric cyst* or neuroenteric cyst* or occult spinal dysraphism* or spinal cord myelodysplasi* or (tethered adj2 cord syndrome*)).tw,kw. (7305)
344 (anencephal* or aprosencephal*).tw,kw. (7561)
345 (congenital*.tw,kw. or cn.fs.) and ((absen* or lack* or missing) adj2 brain*).tw,kw. (122)
346 (congenital*.tw,kw. or cn.fs.) and ((absen* or lack* or missing) adj2 crani$2 vault*).tw,kw. (10)
347 (meroanencephal* or holoanencephal*).tw,kw. (15)
348 (encephalocele or bifid cranium or cephalocele or cerebellar hernia* or cerebral hernia* or cranial meningoencephalocele or craniocele or cranium bifidum or notoencephalocelecranial or tonsillar hernia*).tw,kw. (8218)
349 (rachischisis or schistorrhachis or status dysraphicus).tw,kw. (483)
350 ((cleft or open) adj1 (spine? or spinal)).tw,kw. (570)
351 Spina Bifida/ (16441)
352 (spina? adj (bifida? or bifidum? or dysraphia* or dysraphism*)).tw,kw. (21692)
353 dermal sinus*.tw,kw. (1246)
354 (Arnold-Chiari adj1 (syndrome* or malform* or deform*)).tw,kw. (2233)
355 Chiari malformation?.tw,kw. (6198)
356 (Cantrell* adj1 pentalog*).tw,kw. (171)
357 ((Cantrell Haller Ravitch or thoracoabdominal) adj syndrome*).tw,kw. (21)
358 ((heart or cardiac* or cardio* or aort* or arter* or ventric* or vascular*) adj3 (abnormal* or anomal* or defect* or deform* or malform*)).tw,kw. (277461)
359 ((septa? or septum) adj3 (abnormal* or anomal* or defect* or deform* or malform*)).tw,kw. (66688)
360 Cleft Palate/ (47211)
361 (cleft? adj1 (lip or lips or palat*)).tw,kw. (50862)
362 (congenital fissur* adj1 (lip or lips or palat*)).tw,kw. (0)
363 ((orofacial or oro facial) adj1 cleft?).tw,kw. (2498)
364 (labioschis* or cheiloschis* or abioschiz*).tw,kw. (71)

365 (harelip? or hare lip?).tw,kw. (1679)
366 (palatischis* or palatoschis* or palatoschiz* or palatum fissu*).tw,kw. (324)
367 ((anorectal* or ano-rectal*) adj3 (abnormal* or anomal* or atresia* or defect* or deform* or malform* or stenos*)).tw,kw. (6497)
368 ((anus$2 or anal$2) adj3 (abnormal* or anomal* or atresia* or defect* or deform* or malform* or stenos*)).tw,kw. (6133)
369 ((rectum? or rectal$2) adj3 (abnormal* or anomal* or atresia* or defect* or deform* or malform* or stenos*)).tw,kw. (4548)
370 Hydrocephalus/ (63958)
371 hydrocephal*.tw,kw. (69111)
372 (aqueductal stenos* or cerebral ventriculomegal* or Dandy-Walker or Luschka-Magendie foramina atresia* or Hakim$2).tw,kw. (6345)
373 ((arm or arms or carpal or carpus or femur* or finger? or foot or feet or fibula? or forearm? or fore arm? or hand or hands or hip or hips or humeral or humerus or knee? or kneecap? or leg or legs or metacarp* or metatars* or patell* or radius or radial or talipedes or talipes or tarsal or tibia? or toe or toes or ulna? or wrist?) adj3 (abnormal* or anomal* or defect* or deform* or malform* or reduc*)).tw,kw. (107044)
374 ((limb or limbs) adj3 (abnormal* or anomal* or defect* or deform* or malform* or reduc*)).tw,kw. (21351)
375 (extremit* adj3 (abnormal* or anomal* or defect* or deform* or malform* or reduc*)).tw,kw. (6090)
376 (clubfoot or club foot or clubfeet or club feet).tw,kw. (7899)
377 (flatfoot or flat foot or flatfeet or flat feet).tw,kw. (5316)
378 (arachnodactyl* or brachydactyl* or ectromelia* or amelia* or hemimelia* or phocomelia* or sirenomelia* or hyperdactyl* or polydactyl* or polysyndactyl* or syndactyl*).tw,kw. (23028)
379 gastroschis*.tw,kw. (5672)
380 or/303-379 [OUTCOMES] (6697094)
381 302 and 380 (22899)
382 limit 381 to english [Limit not valid in Joanna Briggs Institute EBP Database -; records were retained] (21023)
383 382 use medall,emczd,coch,cctr,jbi (20440)
384 382 not 383 [PSYCINFO RECORDS] (583)
385 (2019* or 2020*).up. (35531612)
386 384 and 385 [PSYCINFO UPDATE] (38)
387 Pregnancy/ (1629651)
388 exp Pregnancy Complications/ (574252)
389 Pregnant Women/ (78249)
390 exp Pregnancy Trimesters/ (835936)
391 pregnan*.tw,kw. (1320762)
392 Prenatal Care/ (70527)
393 (prenatal* or antenatal* or ante natal* or antepartum or ante partum).tw,kw. (356488)
394 or/387-393 [PREGNANCY] (2313745)
395 exp Overweight/ (773480)
396 (obesity* or obese or overweight or over weight or adiposit*).tw,kw. (899327)
397 (body adj2 (size* or shape* or weight* or fat* or silhouette*)).tw,kw. (714530)

398 exp Adipose Tissue/ (271847)
399 exp Body Fat Distribution/ (21902)
400 ((abdominal* or central* or subcutaneous*) adj2 (obes* or fat* or adipos*)).tw,kw. (100944)
401 Anthropometry/ (97255)
402 Waist Circumference/ or Waist-Hip Ratio/ or Waist-Height Ratio/ (74903)
403 Skinfold Thickness/ (20075)
404 (anthropometr* or BMI or BMIs or skinfold* or SFT or SFTs).tw,kw. (614662)
405 (waist* adj2 (circumference* or hip* or height or thigh*)).tw,kw. (105266)
406 (waisthip or waistheight or waistthigh or WHR or WHtR or WTR).tw,kw. (16319)
407 or/395-406 [ADIPOSITY] (2155529)
408 394 and 407 [ADIPOSITY IN PREGNANCY] (127128)
409 exp Animals/ not (exp Animals/ and Humans/) (18145733)
410 408 not 409 [ANIMAL-ONLY REMOVED] (72726)
411 (comment or editorial or interview or news or newspaper article).pt. (2066673)
412 (letter not (letter and randomized controlled trial)).pt. (2164882)
413 410 not (411 or 412) [OPINION PIECES REMOVED] (71150)
414 exp Risk/ (3665410)
415 (risk or risked or risks or risky or risking or risk-related).tw,kw. (5946921)
416 predict*.tw,kw. (4167721)
417 logistic*.tw,kw. (856074)
418 (logit* adj1 model*).tw,kw. (6575)
419 Prevalence/ (990248)
420 prevalen*.tw,kw. (1984134)
421 Pregnancy Complications/ep [epidemiology] (12669)
422 or/414-421 [RISK/PREDICTION] (11650984)
423 413 and 422 (39093)
424 Pregnancy Outcome/ (110912)
425 ((pregnan* or prenatal* or antenatal* or ante natal* or antepartum or ante partum or perinatal* or peripartum) adj3 outcome*).tw,kw. (118174)
426 ((maternal* or mother* or baby or babies or f?etal* or f?etus* or neonat* or newborn*) adj3 outcome*).tw,kw. (88341)
427 exp Pregnancy Complications/mo [mortality] (6721)
428 Maternal Mortality/ (33772)
429 Fetal Mortality/ (5032)
430 Perinatal Mortality/ (16632)
431 (mortalit* or death* or fatal*).tw,kw. (3957410)
432 exp Fetal Death/ (74852)
433 ((baby or babies or f?etal or f?etus* or neonat* or newborn*) adj3 (dead or demise? or died or dying)).tw,kw. (18096)
434 (stillbirth* or stillborn*).tw,kw. (39507)
435 exp Abortion, Spontaneous/ (80320)
436 ((abort* adj2 spontaneous*) or miscarriage? or (recur* adj2 loss*) or (habit* adj2 abort*)).tw,kw. (75747)
437 exp Hospitalization/ (639998)
438 hospitali*.tw,kw. (746337)

439 ((admit* or admission* or readmit* or readmission*) adj3 (hospital? or critical care or intensive care or ICU or ICUs or NICU or NICUs or SICU or SICUs)).tw,kw. (389590)
440 exp Hypertension, Pregnancy-Induced/ (55111)
441 ((eclamp* or hypertensi* or preeclamp* or pre-eclamp* or toxemi* or toxaemi* or EPH or hemolys#s) adj3 (gestational* or maternal* or "new onset" or pregnancy-induced)).tw,kw. (31785)
442 PIH.tw,kw. (5310)
443 HELLP.tw,kw. (6878)
444 exp Diabetes, Gestational/ (49429)
445 ((diabet* or DM or T2DM) adj3 (gestational* or maternal* or "new onset" or pregnancy-induced)).tw,kw. (57931)
446 PID.tw,kw. (13056)
447 macrosomi*.tw,kw. (11229)
448 Venous Thromboembolism/ (47161)
449 (DVT or thromboemboli* or VTE).tw,kw. (188930)
450 exp Placenta Diseases/ (45964)
451 Placental Circulation/ (7099)
452 (placenta* adj3 (abnormal* or disease* or disorder* or dysfunction*)).tw,kw. (13087)
453 (placenta* adj2 mediate*).tw,kw. (1149)
454 Fetal Growth Retardation/ (33493)
455 ((f?etal or f?etus* or intrauterin*) adj grow* adj3 (restrict* or retard*)).tw,kw. (44703)
456 (FGR or IUGR).tw,kw. (20519)
457 Premature Birth/ (81916)
458 (preterm or prematur*).tw,kw. (532367)
459 exp Cesarean Section/ (149360)
460 (cesarean* or caesarean*).tw,kw. (164121)
461 (C-section* or Csection*).tw,kw. (5413)
462 (abdom* adj3 deliver*).tw,kw. (2426)
463 (postdate* or post-date* or postmatur* or post-matur* or postterm* or post-term*).tw,kw. (9116)
464 (42 week? or 43 week?).tw,kw. (11165)
465 ("42 0/7" or "42 1/7" or "42 2/7" or "42 3/7" or "42 4/7" or "42 5/7" or "42 6/7" or "42 7/7" or "43 0/7" or "43 1/7" or "43 2/7" or "43 3/7" or "43 4/7" or "43 5/7" or "43 6/7" or "43 7/7").tw,kw. (670)
466 Fetal Macrosomia/ (6974)
467 ("large-for-gestational age" or LGA).tw,kw. (9227)
468 Congenital Abnormalities/ (46941)
469 ((birth or congenital) adj3 (abnormal* or anomal* or defect* or deform*)).tw,kw. (159952)
470 exp Neural Tube Defects/ (61770)
471 (NTD or NTDs).tw,kw. (10633)
472 (neural tube? adj2 (defect* or deform* or malform*)).tw,kw. (18102)
473 (acrani* or craniorachischis* or diastematomyeli* or exencephal* or iniencephal* or neurenteric cyst* or neuroenteric cyst* or occult spinal dysraphism* or spinal cord myelodysplasi* or (tethered adj2 cord syndrome*)).tw,kw. (7305)
474 (anencephal* or aprosencephal*).tw,kw. (7561)

475 (congenital*.tw,kw. or cn.fs.) and ((absen* or lack* or missing) adj2 brain*).tw,kw. (122)
476 (congenital*.tw,kw. or cn.fs.) and ((absen* or lack* or missing) adj2 crani$2 vault*).tw,kw. (10)
477 (meroanencephal* or holoanencephal*).tw,kw. (15)
478 (encephalocele or bifid cranium or cephalocele or cerebellar hernia* or cerebral hernia* or cranial meningoencephalocele or craniocele or cranium bifidum or notoencephalocelecranial or tonsillar hernia*).tw,kw. (8218)
479 (rachischisis or schistorrhachis or status dysraphicus).tw,kw. (483)
480 ((cleft or open) adj1 (spine? or spinal)).tw,kw. (570)
481 (spina? adj (bifida? or bifidum? or dysraphia* or dysraphism*)).tw,kw. (21692)
482 dermal sinus*.tw,kw. (1246)
483 (Arnold-Chiari adj1 (syndrome* or malform* or deform*)).tw,kw. (2233)
484 Chiari malformation?.tw,kw. (6198)
485 (Cantrell* adj1 pentalog*).tw,kw. (171)
486 ((Cantrell Haller Ravitch or thoracoabdominal) adj syndrome*).tw,kw. (21)
487 exp Cardiovascular Abnormalities/ (416346)
488 ((heart or cardiac* or cardio* or aort* or arter* or ventric* or vascular*) adj3 (abnormal* or anomal* or defect* or deform* or malform*)).tw,kw. (277461)
489 ((septa? or septum) adj3 (abnormal* or anomal* or defect* or deform* or malform*)).tw,kw. (66688)
490 Cleft Lip/ (32086)
491 Cleft Palate/ (47211)
492 (cleft? adj1 (lip or lips or palat*)).tw,kw. (50862)
493 (congenital fissur* adj1 (lip or lips or palat*)).tw,kw. (0)
494 ((orofacial or oro facial) adj1 cleft?).tw,kw. (2498)
495 (labioschis* or cheiloschis* or abioschiz*).tw,kw. (71)
496 (harelip? or hare lip?).tw,kw. (1679)
497 (palatischis* or palatoschis* or palatoschiz* or palatum fissu*).tw,kw. (324)
498 Anorectal Malformations/ (3182)
499 ((anorectal* or ano-rectal*) adj3 (abnormal* or anomal* or atresia* or defect* or deform* or malform* or stenos*)).tw,kw. (6497)
500 ((anus$2 or anal$2) adj3 (abnormal* or anomal* or atresia* or defect* or deform* or malform* or stenos*)).tw,kw. (6133)
501 ((rectum? or rectal$2) adj3 (abnormal* or anomal* or atresia* or defect* or deform* or malform* or stenos*)).tw,kw. (4548)
502 Hydrocephalus/ (63958)
503 hydrocephal*.tw,kw. (69111)
504 (aqueductal stenos* or cerebral ventriculomegal* or Dandy-Walker or Luschka-Magendie foramina atresia* or Hakim$2).tw,kw. (6345)
505 exp Limb Deformities, Congenital/ (73214)
506 ((arm or arms or carpal or carpus or femur* or finger? or foot or feet or fibula? or forearm? or fore arm? or hand or hands or hip or hips or humeral or humerus or knee? or kneecap? or leg or legs or metacarp* or metatars* or patell* or radius or radial or talipedes or talipes or tarsal or tibia? or toe or toes or ulna? or wrist?) adj3 (abnormal* or anomal* or defect* or deform* or malform* or reduc*)).tw,kw. (107044)

507 ((limb or limbs) adj3 (abnormal* or anomal* or defect* or deform* or malform* or reduc*)).tw,kw. (21351)
508 (extremit* adj3 (abnormal* or anomal* or defect* or deform* or malform* or reduc*)).tw,kw. (6090)
509 (clubfoot or club foot or clubfeet or club feet).tw,kw. (7899)
510 (flatfoot or flat foot or flatfeet or flat feet).tw,kw. (5316)
511 (arachnodactyl* or brachydactyl* or ectromelia* or amelia* or hemimelia* or phocomelia* or sirenomelia* or hyperdactyl* or polydactyl* or polysyndactyl* or syndactyl*).tw,kw. (23028)
512 Gastroschisis/ (4995)
513 gastroschis*.tw,kw. (5672)
514 or/424-513 [OUTCOMES] (6964826)
515 423 and 514 (24104)
516 limit 515 to english [Limit not valid in Joanna Briggs Institute EBP Database -; records were retained] (22120)
517 516 use jbi [J BRIGGS RECORDS] (123)
518 ("2019" or "2020").yr. (3794293)
519 517 and 518 [J BRIGGS UPDATE] (36)
520 Pregnancy/ (1629651)
521 exp Pregnancy Complications/ (574252)
522 Pregnant Women/ (78249)
523 exp Pregnancy Trimesters/ (835936)
524 pregnan*.tw,kw. (1320762)
525 Prenatal Care/ (70527)
526 (prenatal* or antenatal* or ante natal* or antepartum or ante partum).tw,kw. (356488)
527 or/520-526 [PREGNANCY] (2313745)
528 exp Overweight/ (773480)
529 (obesity* or obese or overweight or over weight or adiposit*).tw,kw. (899327)
530 (body adj2 (size* or shape* or weight* or fat* or silhouette*)).tw,kw. (714530)
531 exp Adipose Tissue/ (271847)
532 exp Body Fat Distribution/ (21902)
533 ((abdominal* or central* or subcutaneous*) adj2 (obes* or fat* or adipos*)).tw,kw. (100944)
534 Anthropometry/ (97255)
535 Waist Circumference/ or Waist-Hip Ratio/ or Waist-Height Ratio/ (74903)
536 Skinfold Thickness/ (20075)
537 (anthropometr* or BMI or BMIs or skinfold* or SFT or SFTs).tw,kw. (614662)
538 (waist* adj2 (circumference* or hip* or height or thigh*)).tw,kw. (105266)
539 (waisthip or waistheight or waistthigh or WHR or WHtR or WTR).tw,kw. (16319)
540 or/528-539 [ADIPOSITY] (2155529)
541 527 and 540 [ADIPOSITY IN PREGNANCY] (127128)
542 exp Animals/ not (exp Animals/ and Humans/) (18145733)
543 541 not 542 [ANIMAL-ONLY REMOVED] (72726)
544 (comment or editorial or interview or news or newspaper article).pt. (2066673)
545 (letter not (letter and randomized controlled trial)).pt. (2164882)
546 543 not (544 or 545) [OPINION PIECES REMOVED] (71150)

547 exp Risk/ (3665410)
548 (risk or risked or risks or risky or risking or risk-related).tw,kw. (5946921)
549 predict*.tw,kw. (4167721)
550 logistic*.tw,kw. (856074)
551 (logit* adj1 model*).tw,kw. (6575)
552 Prevalence/ (990248)
553 prevalen*.tw,kw. (1984134)
554 Pregnancy Complications/ep [epidemiology] (12669)
555 or/547-554 [RISK/PREDICTION] (11650984)
556 546 and 555 (39093)
557 Pregnancy Outcome/ (110912)
558 ((pregnan* or prenatal* or antenatal* or ante natal* or antepartum or ante partum or perinatal* or peripartum) adj3 outcome*).tw,kw. (118174)
559 ((maternal* or mother* or baby or babies or f?etal* or f?etus* or neonat* or newborn*) adj3 outcome*).tw,kw. (88341)
560 exp Pregnancy Complications/mo [mortality] (6721)
561 Maternal Mortality/ (33772)
562 Fetal Mortality/ (5032)
563 Perinatal Mortality/ (16632)
564 (mortalit* or death* or fatal*).tw,kw. (3957410)
565 exp Fetal Death/ (74852)
566 ((baby or babies or f?etal or f?etus* or neonat* or newborn*) adj3 (dead or demise? or died or dying)).tw,kw. (18096)
567 (stillbirth* or stillborn*).tw,kw. (39507)
568 exp Abortion, Spontaneous/ (80320)
569 ((abort* adj2 spontaneous*) or miscarriage? or (recur* adj2 loss*) or (habit* adj2 abort*)).tw,kw. (75747)
570 exp Hospitalization/ (639998)
571 hospitali*.tw,kw. (746337)
572 ((admit* or admission* or readmit* or readmission*) adj3 (hospital? or critical care or intensive care or ICU or ICUs or NICU or NICUs or SICU or SICUs)).tw,kw. (389590)
573 exp Hypertension, Pregnancy-Induced/ (55111)
574 ((eclamp* or hypertensi* or preeclamp* or pre-eclamp* or toxemi* or toxaemi* or EPH or hemolys#s) adj3 (gestational* or maternal* or "new onset" or pregnancy-induced)).tw,kw. (31785)
575 PIH.tw,kw. (5310)
576 HELLP.tw,kw. (6878)
577 exp Diabetes, Gestational/ (49429)
578 ((diabet* or DM or T2DM) adj3 (gestational* or maternal* or "new onset" or pregnancy-induced)).tw,kw. (57931)
579 PID.tw,kw. (13056)
580 macrosomi*.tw,kw. (11229)
581 Venous Thromboembolism/ (47161)
582 (DVT or thromboemboli* or VTE).tw,kw. (188930)
583 exp Placenta Diseases/ (45964)
584 Placental Circulation/ (7099)

585 (placenta* adj3 (abnormal* or disease* or disorder* or dysfunction*)).tw,kw. (13087)
586 (placenta* adj2 mediate*).tw,kw. (1149)
587 Fetal Growth Retardation/ (33493)
588 ((f?etal or f?etus* or intrauterin*) adj grow* adj3 (restrict* or retard*)).tw,kw. (44703)
589 (FGR or IUGR).tw,kw. (20519)
590 Premature Birth/ (81916)
591 (preterm or prematur*).tw,kw. (532367)
592 exp Cesarean Section/ (149360)
593 (cesarean* or caesarean*).tw,kw. (164121)
594 (C-section* or Csection*).tw,kw. (5413)
595 (abdom* adj3 deliver*).tw,kw. (2426)
596 (postdate* or post-date* or postmatur* or post-matur* or postterm* or post-term*).tw,kw. (9116)
597 (42 week? or 43 week?).tw,kw. (11165)
598 ("42 0/7" or "42 1/7" or "42 2/7" or "42 3/7" or "42 4/7" or "42 5/7" or "42 6/7" or "42 7/7" or "43 0/7" or "43 1/7" or "43 2/7" or "43 3/7" or "43 4/7" or "43 5/7" or "43 6/7" or "43 7/7").tw,kw. (670)
599 Fetal Macrosomia/ (6974)
600 ("large-for-gestational age" or LGA).tw,kw. (9227)
601 Congenital Abnormalities/ (46941)
602 ((birth or congenital) adj3 (abnormal* or anomal* or defect* or deform*)).tw,kw. (159952)
603 exp Neural Tube Defects/ (61770)
604 (NTD or NTDs).tw,kw. (10633)
605 (neural tube? adj2 (defect* or deform* or malform*)).tw,kw. (18102)
606 (acrani* or craniorachischis* or diastematomyeli* or exencephal* or iniencephal* or neurenteric cyst* or neuroenteric cyst* or occult spinal dysraphism* or spinal cord myelodysplasi* or (tethered adj2 cord syndrome*)).tw,kw. (7305)
607 (anencephal* or aprosencephal*).tw,kw. (7561)
608 (congenital*.tw,kw. or cn.fs.) and ((absen* or lack* or missing) adj2 brain*).tw,kw. (122)
609 (congenital*.tw,kw. or cn.fs.) and ((absen* or lack* or missing) adj2 crani$2 vault*).tw,kw. (10)
610 (meroanencephal* or holoanencephal*).tw,kw. (15)
611 (encephalocele or bifid cranium or cephalocele or cerebellar hernia* or cerebral hernia* or cranial meningoencephalocele or craniocele or cranium bifidum or notoencephalocelecranial or tonsillar hernia*).tw,kw. (8218)
612 (rachischisis or schistorrhachis or status dysraphicus).tw,kw. (483)
613 ((cleft or open) adj1 (spine? or spinal)).tw,kw. (570)
614 (spina? adj (bifida? or bifidum? or dysraphia* or dysraphism*)).tw,kw. (21692)
615 dermal sinus*.tw,kw. (1246)
616 (Arnold-Chiari adj1 (syndrome* or malform* or deform*)).tw,kw. (2233)
617 Chiari malformation?.tw,kw. (6198)
618 (Cantrell* adj1 pentalog*).tw,kw. (171)
619 ((Cantrell Haller Ravitch or thoracoabdominal) adj syndrome*).tw,kw. (21)
620 exp Cardiovascular Abnormalities/ (416346)

621 ((heart or cardiac* or cardio* or aort* or arter* or ventric* or vascular*) adj3 (abnormal* or anomal* or defect* or deform* or malform*)).tw,kw. (277461)
622 ((septa? or septum) adj3 (abnormal* or anomal* or defect* or deform* or malform*)).tw,kw. (66688)
623 Cleft Lip/ (32086)
624 Cleft Palate/ (47211)
625 (cleft? adj1 (lip or lips or palat*)).tw,kw. (50862)
626 (congenital fissur* adj1 (lip or lips or palat*)).tw,kw. (0)
627 ((orofacial or oro facial) adj1 cleft?).tw,kw. (2498)
628 (labioschis* or cheiloschis* or abioschiz*).tw,kw. (71)
629 (harelip? or hare lip?).tw,kw. (1679)
630 (palatischis* or palatoschis* or palatoschiz* or palatum fissu*).tw,kw. (324)
631 Anorectal Malformations/ (3182)
632 ((anorectal* or ano-rectal*) adj3 (abnormal* or anomal* or atresia* or defect* or deform* or malform* or stenos*)).tw,kw. (6497)
633 ((anus$2 or anal$2) adj3 (abnormal* or anomal* or atresia* or defect* or deform* or malform* or stenos*)).tw,kw. (6133)
634 ((rectum? or rectal$2) adj3 (abnormal* or anomal* or atresia* or defect* or deform* or malform* or stenos*)).tw,kw. (4548)
635 Hydrocephalus/ (63958)
636 hydrocephal*.tw,kw. (69111)
637 (aqueductal stenos* or cerebral ventriculomegal* or Dandy-Walker or Luschka-Magendie foramina atresia* or Hakim$2).tw,kw. (6345)
638 exp Limb Deformities, Congenital/ (73214)
639 ((arm or arms or carpal or carpus or femur* or finger? or foot or feet or fibula? or forearm? or fore arm? or hand or hands or hip or hips or humeral or humerus or knee? or kneecap? or leg or legs or metacarp* or metatars* or patell* or radius or radial or talipedes or talipes or tarsal or tibia? or toe or toes or ulna? or wrist?) adj3 (abnormal* or anomal* or defect* or deform* or malform* or reduc*)).tw,kw. (107044)
640 ((limb or limbs) adj3 (abnormal* or anomal* or defect* or deform* or malform* or reduc*)).tw,kw. (21351)
641 (extremit* adj3 (abnormal* or anomal* or defect* or deform* or malform* or reduc*)).tw,kw. (6090)
642 (clubfoot or club foot or clubfeet or club feet).tw,kw. (7899)
643 (flatfoot or flat foot or flatfeet or flat feet).tw,kw. (5316)
644 (arachnodactyl* or brachydactyl* or ectromelia* or amelia* or hemimelia* or phocomelia* or sirenomelia* or hyperdactyl* or polydactyl* or polysyndactyl* or syndactyl*).tw,kw. (23028)
645 Gastroschisis/ (4995)
646 gastroschis*.tw,kw. (5672)
647 or/557-646 [OUTCOMES] (6964826)
648 556 and 647 (24104)
649 limit 648 to english [Limit not valid in Joanna Briggs Institute EBP Database -; records were retained] (22120)
650 (conference abstract or journal conference abstract).pt. (3885879)
651 649 not 650 [CONFERENCE ABSTRACTS REMOVED] (18384)

652 649 use coch [COCHRANE DSR RECORDS] (557)
653 (2019* or 2020*).up. (35531612)
654 652 and 653 [COCHRANE DSR UPDATE] (156)
655 651 use cctr [CENTRAL RECORDS] (825)
656 (2019* or 2020*).up. (35531612)
657 655 and 656 [CENTRAL UPDATE] (649)
658 133 or 274 or 386 or 519 or 654 or 657 [ALL DATABASES - UPDATE PERIOD] (3762)
659 remove duplicates from 658 (2795)
660 659 use medall [MEDLINE UNIQUE RECORDS] (1095)
661 659 use emczd [EMBASE UNIQUE RECORDS] (871)
662 659 use jbi [J BRIGGS UNIQUE RECORDS] (36)
663 659 use cctr [CENTRAL UNIQUE RECORDS] (610)
664 659 use coch [COCHRANE DSR UNIQUE RECORDS] (156)
665 659 not (660 or 661 or 662 or 663 or 664) [PSYCINFO UNIQUE RECORDS] (27)

****

File S2: List of Included Studies

Sen, S. et al. Dietary Inflammatory Potential during Pregnancy Is Associated with Lower Fetal Growth and Breastfeeding Failure: Results from Project Viva. J. Nutr. 146, 728–736 (2016).

Davies-Tuck, M., Mockler, J. C., Stewart, L., Knight, M. & Wallace, E. M. Obesity and pregnancy outcomes: Do the relationships differ by maternal region of birth? A retrospective cohort study.BMC Pregnancy Childbirth 16, 288 (2016).

Marshall, N. E., Guild, C., Cheng, Y. W., Caughey, A. B. & Halloran, D. R. Racial disparities in pregnancy outcomes in obese women. J. Matern.-Fetal Neonatal Med. Off. J. Eur. Assoc. Perinat. Med. Fed. Asia Ocean.Perinat. Soc. Int. Soc. Perinat. Obstet. 27, 122–126 (2014).

Machtinger, R. et al. The effect of mode of conception on obstetrical outcomes differs by body mass index. Reprod. Biomed.Online 31, 531–537 (2015).

Snowden, J. M. et al. The Impact of maternal obesity and race/ethnicity on perinatal outcomes: Independent and joint effects. Obes.Silver Spring Md 24, 1590–1598 (2016).

Elkholi DGE & Nagy H.The effects of adipocytokines on the endocrino-metabolic features and obstetric outcome in pregnant obese women with polycystic ovary syndrome. Middle East Fertil. Soc. J. 19, 293–302 (2014).

Parker, M. H., Berghella, V. &Nijjar, J. B. Bariatric surgery and associated adverse pregnancy outcomes among obese women. J. Matern.-Fetal Neonatal Med. Off. J. Eur. Assoc. Perinat. Med. Fed. Asia Ocean.Perinat. Soc. Int. Soc. Perinat. Obstet. 29, 1747–1750 (2016).

Persson, M., Pasupathy, D., Hanson, U., Westgren, M. & Norman, M. Pre-pregnancy body mass index and the risk of adverse outcome in type 1 diabetic pregnancies: a population-based cohort study. BMJ Open 2, e000601 (2012).

Lamminpää, R., Vehviläinen-Julkunen, K., Gissler, M., Selander, T. &Heinonen, S. Pregnancy outcomes of overweight and obese women aged 35 years or older - A registry-based study in Finland.Obes. Res. Clin. Pract.10, 133–142 (2016).

Metsälä, J., Stach-Lempinen, B., Gissler, M., Eriksson, J. G. &Koivusalo, S. Risk of Pregnancy Complications in Relation to Maternal Prepregnancy Body Mass Index: Population-Based Study from Finland 2006-10. Paediatr.Perinat.Epidemiol.30, 28–37 (2016).

Houde, M. et al.The Effect of Adequate Gestational Weight Gain among Adolescents Relative to Adults of Equivalent Body Mass Index and the Risk of Preterm Birth, Cesarean Delivery, and Low Birth Weight. J. Pediatr. Adolesc.Gynecol. 28, 502–507 (2015).

Masho, S. W., Bishop, D. L. & Munn, M. Pre-pregnancy BMI and weight gain: where is the tipping point for preterm birth? BMC Pregnancy Childbirth 13, 120 (2013).

Kim, S. Y. et al. Fraction of gestational diabetes mellitus attributable to overweight and obesity by race/ethnicity, California, 2007-2009. Am. J. Public Health 103, e65-72 (2013).

Ducarme, G. et al. Neonatal outcomes in pregnancies after bariatric surgery: a retrospective multi-centric cohort study in three French referral centers. J. Matern.-Fetal Neonatal Med. Off. J. Eur. Assoc. Perinat. Med. Fed. Asia Ocean.Perinat. Soc. Int. Soc. Perinat. Obstet. 26, 275–278 (2013).

Halloran, D. R., Marshall, N. E., Kunovich, R. M. &Caughey, A. B. Obesity trends and perinatal outcomes in black and white teenagers. Am. J. Obstet. Gynecol. 207, 492.e1-7 (2012).

Louis, J. et al. Perinatal outcomes associated with obstructive sleep apnea in obese pregnant women. Obstet. Gynecol. 120, 1085–1092 (2012).

Hedderson, M. et al. Racial/ethnic disparities in the prevalence of gestational diabetes mellitus by BMI.Diabetes Care 35, 1492–1498 (2012).

Olivarez, S. A. et al. Obstructive sleep apnea screening in pregnancy, perinatal outcomes, and impact of maternal obesity. Am. J. Perinatol. 28, 651–658 (2011).

Salihu, H. et al. Nulliparity and preterm birth in the era of obesity epidemic. J. Matern.-Fetal Neonatal Med. Off. J. Eur. Assoc. Perinat. Med. Fed. Asia Ocean.Perinat. Soc. Int. Soc. Perinat. Obstet. 23, 1444–1450 (2010).

Aliyu, M. H. et al. Obesity in older mothers, gestational weight gain, and risk estimates for preterm phenotypes. Maturitas 66, 88–93 (2010).

Aliyu, M. H., Luke, S., Kristensen, S., Alio, A. P. &Salihu, H. M. Joint effect of obesity and teenage pregnancy on the risk of pre-eclampsia: a population-based study. J. Adolesc. Health Off. Publ. Soc. Adolesc. Med. 46, 77–82 (2010).

Shachar, B. Z. et al. Effects of race/ethnicity and BMI on the association between height and risk for spontaneous preterm birth.Am. J. Obstet. Gynecol. 213, 700.e1-9 (2015).

Salihu, H. M. et al. The superobese mother and ethnic disparities in preterm birth.J. Natl. Med. Assoc. 101, 1125–1131 (2009).

Thrift, A. P. & Callaway, L. K.The effect of obesity on pregnancy outcomes among Australian Indigenous and non-Indigenous women.Med. J. Aust. 201, 592–595 (2014).

Barton, J. R. et al. Spontaneously conceived pregnancy after 40: influence of age and obesity on outcome. Am. J. Perinatol. 31, 795–798 (2014).

Kim, C. et al. Are gestational diabetes mellitus and preconception diabetes mellitus less common in non-Hispanic black women than in non-Hispanic white women? Matern.Child Health J. 18, 698–706 (2014).

Reeske, A., Zeeb, H., Razum, O. &Spallek, J. Differences in the Incidence of Gestational Diabetes between Women of Turkish and German Origin: An Analysis of Health Insurance Data From a Statutory Health Insurance in Berlin, Germany (AOK), 2005-2007. GeburtshilfeFrauenheilkd. 72, 305–310 (2012).

Lynch, A. M. et al. Prepregnancy obesity and complement system activation in early pregnancy and the subsequent development of pre-eclampsia. Am. J. Obstet. Gynecol. 206, 428.e1-8 (2012).

Belogolovkin, V. et al. Impact of prior bariatric surgery on maternal and fetal outcomes among obese and non-obese mothers. Arch. Gynecol. Obstet. 285, 1211–1218 (2012).

Hogh S, Wolf HT, von Euler-Chelpin M, et al. Multivitamin use and risk of preeclampsia in a high-income population: A cohort study. *Sex Reprod Healthc*. Jun 2020;24:100500. doi:10.1016/j.srhc.2020.100500

Njagu R, Adkins L, Tucker A, et al. Maternal weight gain and neonatal outcomes in women with class III obesity. *J Matern Fetal Neonatal Med*. Feb 23 2020:1-5. doi:10.1080/14767058.2020.1729116

Malik S, Teh JL, Lomanto D, Kim G, So JB, Shabbir A. Maternal and fetal outcomes of Asian pregnancies after bariatric surgery. *Surg Obes Relat Dis*. Apr 2020;16(4):529-535. doi:10.1016/j.soard.2020.01.017

Porteous H, de Jersey S, Palmer M. Attendance rates and characteristics of women with obesity referred to the dietitian for individual weight management advice during pregnancy. *Aust N Z J Obstet Gynaecol*. Oct 2020;60(5):690-697. doi:10.1111/ajo.13128

Pratt A, Howat P, Hui L. Maternal and perinatal outcomes for women with body mass index >/=50 kg/m(2) in a non-tertiary hospital setting. *Aust N Z J Obstet Gynaecol*. Jun 2020;60(3):361-368. doi:10.1111/ajo.13064

Dolin CD, Chervenak J, Pivo S, Ude Welcome A, Kominiarek MA. Association between time interval from bariatric surgery to pregnancy and maternal weight outcomes. *J Matern Fetal Neonatal Med*. Nov 13 2019:1-7. doi:10.1080/14767058.2019.1683156

Browne K, Park BY, Goetzinger KR, Caughey AB, Yao R. The joint effects of obesity and pregestational diabetes on the risk of stillbirth. *J Matern Fetal Neonatal Med*. Feb 2019;34(3):332-338. doi:10.1080/14767058.2019.1607287

Ijas H, Koivunen S, Raudaskoski T, Kajantie E, Gissler M, Vaarasmaki M. Independent and concomitant associations of gestational diabetes and maternal obesity to perinatal outcome: A register-based study. *PLoS One*. 2019;14(8):e0221549. doi:10.1371/journal.pone.0221549

Karadag C, Demircan S, Caliskan E. Effects of laparoscopic sleeve gastrectomy on obstetric outcomes within 12 months after surgery. *J Obstet Gynaecol Res*. Feb 2020;46(2):266-271. doi:10.1111/jog.14165

Ram M, Berger H, Lipworth H, et al. The relationship between maternal body mass index and pregnancy outcomes in twin compared with singleton pregnancies. *Int J Obes (Lond)*. Jan 2020;44(1):33-44. doi:10.1038/s41366-019-0362-8

Meghelli L, Vambergue A, Drumez E, Deruelle P. Complications of pregnancy in morbidly obese patients: What is the impact of gestational diabetes mellitus? *J Gynecol Obstet Hum Reprod*. Jan 2020;49(1):101628. doi:10.1016/j.jogoh.2019.101628

Fallatah AM, Bahrawi AJ, Babatin H, Nassibi KM, AlEdreesi Y, Abduljabbar HS. Pregnancy Outcomes among Obese Pregnant Women with Varying Levels of Vitamin D in King Abdulaziz University Hospital: A Single-center Retrospective Study. *Cureus*. Nov 23 2019;11(11):e6220. doi:10.7759/cureus.6220

Bar-Zeev Y, Haile ZT, Chertok IA. Association Between Prenatal Smoking and Gestational Diabetes Mellitus. *Obstet Gynecol*. Jan 2020;135(1):91-99. doi:10.1097/AOG.0000000000003602

Kong L, Nilsson IAK, Gissler M, Lavebratt C. Associations of Maternal Diabetes and Body Mass Index With Offspring Birth Weight and Prematurity. *JAMA Pediatr*. Apr 1 2019;173(4):371-378. doi:10.1001/jamapediatrics.2018.5541

Ukah UV, Bayrampour H, Sabr Y, et al. Association between gestational weight gain and severe adverse birth outcomes in Washington State, US: A population-based retrospective cohort study, 2004-2013. *PLoS Med*. Dec 2019;16(12):e1003009. doi:10.1371/journal.pmed.1003009

Feghali MN, Catov JM, Zantow E, Mission J, Caritis SN, Scifres CM. Timing of Gestational Weight Gain and Adverse Perinatal Outcomes in Overweight and Obese Women. *Obstet Gynecol*. May 2019;133(5):962-970. doi:10.1097/AOG.0000000000003234

Roussel E, Touleimat S, Ollivier L, Verspyck E. Birthweight and pregnancy outcomes in obese class II women with low weight gain: A retrospective study. *PLoS One*. 2019;14(5):e0215833. doi:10.1371/journal.pone.0215833

Nowak M, Kalwa M, Oleksy P, Marszalek K, Radon-Pokracka M, Huras H. The relationship between pre-pregnancy BMI, gestational weight gain and neonatal birth weight: a retrospective cohort study. *Ginekol Pol*. 2019;90(1):50-54. doi:10.5603/GP.2019.0008

Thompson AM, Thompson JA. An evaluation of whether a gestational weight gain of 5 to 9 kg for obese women optimizes maternal and neonatal health risks. *BMC Pregnancy Childbirth*. Apr 11 2019;19(1):126. doi:10.1186/s12884-019-2273-z

Benjamin RH, Littlejohn S, Canfield MA, Ethen MK, Hua F, Mitchell LE. Interpregnancy change in body mass index and infant outcomes in Texas: a population-based study. *BMC Pregnancy Childbirth*. Apr 5 2019;19(1):119. doi:10.1186/s12884-019-2265-z

Thagaard IN, Hedley PL, Holm JC, et al. Leptin and Adiponectin as markers for preeclampsia in obese pregnant women, a cohort study. *Pregnancy Hypertens*. Jan 2019;15:78-83. doi:10.1016/j.preghy.2018.12.002

Grove G, Ziauddeen N, Harris S, Alwan NA. Maternal interpregnancy weight change and premature birth: Findings from an English population-based cohort study. *PLoS One*. 2019;14(11):e0225400. doi:10.1371/journal.pone.0225400

Shaukat S, Nur U. Effect of prepregnancy maternal BMI on adverse pregnancy and neonatal outcomes: results from a retrospective cohort study of a multiethnic population in Qatar. *BMJ Open*. Sep 8 2019;9(9):e029757. doi:10.1136/bmjopen-2019-029757

Moore Simas TA, Waring ME, Callaghan K, et al. Weight gain in early pregnancy and risk of gestational diabetes mellitus among Latinas. *Diabetes Metab*. Jan 2019;45(1):26-31. doi:10.1016/j.diabet.2017.10.006

Frankenthal D, Hirsh-Yechezkel G, Boyko V, et al. The effect of body mass index (BMI) and gestational weight gain on adverse obstetrical outcomes in pregnancies following assisted reproductive technology as compared to spontaneously conceived pregnancies. *Obes Res Clin Pract*. Mar - Apr 2019;13(2):150-155. doi:10.1016/j.orcp.2018.11.239

Laine MK, Masalin S, Rono K, et al. Risk of preterm birth in primiparous women with exposure to antidepressant medication before pregnancy and/or during pregnancy - impact of body mass index. *Ann Med*. Feb 2019;51(1):51-57. doi:10.1080/07853890.2018.1534265

Boudet-Berquier J, Salanave B, Desenclos JC, Castetbon K. Sociodemographic factors and pregnancy outcomes associated with prepregnancy obesity: effect modification of parity in the nationwide Epifane birth-cohort. *BMC Pregnancy Childbirth*. Aug 25 2017;17(1):273. doi:10.1186/s12884-017-1456-8

Janevic T, Zeitlin J, Egorova N, Balbierz A, Howell EA. The role of obesity in the risk of gestational diabetes among immigrant and U.S.-born women in New York City. *Ann Epidemiol*. Apr 2018;28(4):242-248. doi:10.1016/j.annepidem.2018.02.006

Anderson KG, Spicer P, Peercy MT. Obesity, Diabetes, and Birth Outcomes Among American Indians and Alaska Natives. *Matern Child Health J*. Dec 2016;20(12):2548-2556. doi:10.1007/s10995-016-2080-3

Zamora-Kapoor A, Nelson LA, Buchwald DS, Walker LR, Mueller BA. Pre-eclampsia in American Indians/Alaska Natives and Whites: The Significance of Body Mass Index. *Matern Child Health J*. Nov 2016;20(11):2233-2238. doi:10.1007/s10995-016-2126-6

Gernand AD, Simhan HN, Caritis S, Bodnar LM. Maternal vitamin D status and small-for-gestational-age offspring in women at high risk for preeclampsia. *Obstet Gynecol*. Jan 2014;123(1):40-48. doi:10.1097/AOG.0000000000000049

Subramaniam A, Jauk VC, Tita A, Harper LM. Interaction between maternal obesity and 1-hour glucose challenge test results on maternal and perinatal outcomes. *Am J Perinatol*. Jul 2015;32(8):771-8. doi:10.1055/s-0034-1396695

File S3: List of Excluded Studies (from Full Text Screening)

No full-text

A.M. Pauley,G.A. Moore,S.K. Mama,P. Molenaar,D.S. Downs. Associations Between Prenatal Sleep and Psychological Health: A Systematic Review. Journal of clinical sleep medicine : JCSM : official publication of the American Academy of Sleep Medicine. 2020/01/31/. 2020 Jan 31:#pages#

A.R. Tucker,H.L. Brown,S.K. Dotters-Katz. Maternal Weight Gain and Infant Birth Weight in Women with Class III Obesity. American journal of perinatology. 2019/12/31/. 2019 Dec 31:#pages#

Aamir AH. The obese pregnancy. JPMA J Pak Med Assoc 2016;66(Suppl 1)(9):S65-S68

Amaral WT, Souza SS, Peracoli JC. PP175. Gestational hypertensive syndromes: Risk factors identification and complications in a primiparous population. Pregnancy Hypertens 2012;2(3):335

Antsaklis P, Antsaklis A, Papantoniou N, Kurjak A. Application of KANET in special cases: Part I. Donald Sch J Ultrasound Obstet Gynecol 2013;7(2):200-7. [PMID: http://www.dsjuog.com/]

Ashley [BAppSc BHlthSc (Hons) PhD] Whitehorn. Antenatal Care: Fundamentals (Low Risk Pregnancy). #journal#. 2019///. 78:#pages#

Ashley [BAppSc BHlthSc (Hons) PhD] Whitehorn. Antenatal Care: Fundamentals (Low Risk Pregnancy). #journal#. 2019///. #volume#:#pages#

Ashley [BAppSc BHlthSc (Hons) PhD] Whitehorn. Gestational Diabetes: Screening Approaches. #journal#. 2019///. #volume#:#pages#

Ashley [BAppSc BHlthSc (Hons) PhD] Whitehorn. Gestational Diabetes: Management (Exercise). #journal#. 2019///. #volume#:#pages#

Ashley [BAppSc BHlthSc (Hons) PhD] Whitehorn. Miscarriage (Recurrent): Management. #journal#. 2019///. #volume#:#pages#

Ashley [BAppSc BHlthSc (Hons) PhD] Whitehorn. Pre-eclampsia: Risk Factors. #journal#. 2019///. #volume#:#pages#

Ashley [BAppSc BHlthSc (Hons) PhD] Whitehorn. Pre-eclampsia: Screening. #journal#. 2019///. #volume#:#pages#

Bogaerts A, Ameye L, Martens E, Devlieger R. Erratum: Weight Loss in Obese Pregnant Women and Risk for Adverse Perinatal Outcomes (Obstetrics and Gynecology (2015) 125 (566-575)). Obstet Gynecol 2015;126(2):452-3. [PMID: http://journals.lww.com/greenjournal]

Brocato, B., Lewis, D., Mulekar, M., and Baker, S.. 47th American Association of Gynecologic Laparoscopists (AAGL) Global Congress on Minimally Invasive Gynecologic Surgery (MIGS). Journal of Minimally Invasive Gynecology. 2018///. Conference::#pages#

Chronic Disease Node Group and Marin, Tania. Obesity: Smoking Cessation. #journal#. 2018. #volume#:#pages#

Chronic, Disease Node. Obesity (Smoking Cessation): Considerations for Patients. #journal#. 2017. #volume#:#pages#

Cidade DG, Margotto PR, Guedes ACBS, Rocha AA, Assis FR, Cardoso FF, et al. PP156. High prevalence of pre-pregnancy overweight and obesity associated with maternal and perinatal complications. Pregnancy Hypertens 2012;2(3):323

D. Dabelea,K.A. Sauder. Intrauterine exposure to maternal diabetes and childhood obesity. Contemporary Endocrinology. 2018///. 97:229

D. Setyorini,B. Santoso,S. Martini,Ernawati,I. Cahyono. Early detection score of preeclampsia risk. Indian Journal of Forensic Medicine and Toxicology. 2019///. 13:1717

D.M. Marks,M.-H. Park,B.-J. Ham,C. Han,A.A. Patkar,P.S. Masand,C.-U. Pae. Paroxetine: safety and tolerability issues. Expert Opinion on Drug Safety. 2008///. 7:783

D.R.J. Arachchillage,M. Laffan. Pathogenesis and Management of Thrombotic Disease in Myeloproliferative Neoplasms. Seminars in thrombosis and hemostasis. 2019///. 45:604

E. Kapoor,S.S. Faubion,J.M. Kling. Obesity Update in Women. Journal of women's health (2002). 2019/12//. 28:1601

E.A. Mohammed. Maternl and neonatal outcomes of elective and emergency cesarean sections. Indian Journal of Public Health Research and Development. 2019///. 10:422

F.B. Kampmann,A.C.B. Thuesen,L. Hjort,A.A. Bjerregaard,J.E. Chavarro,J. Frystyk,M. Bjerre,I. Tetens,S.F. Olsen,A.A. Vaag,P. Damm,L.G. Grunnet. Increased leptin, decreased adiponectin and FGF21 concentrations in adolescent offspring of women with gestational diabetes. European journal of endocrinology / European Federation of Endocrine Societies. 2019/12//. 181:691

Farren M, Daly N, O'Higgins AC, McKeating A, Maguire PJ, Turner MJ. The interplay between maternal obesity and gestational diabetes mellitus. J Perinat Med 2015;43(3):311-7.

G.M. Freeman. The efficacy of prophylactic antibiotics in high-risk patients undergoing cesarean section. Journal of the American Osteopathic Association. 1982///. 81:610

Gaillard R, Durmus B, Hofman A, Mackenbach J, Steegers E, Jaddoe V. OS021. Risk factors and outcomes of maternal obesity and excessive weight gain during pregnancy. Pregnancy Hypertens 2012;2(3):186

Gasse, C., Boutin, A., Demers, S., Chaillet, N., and Bujold, E.. CHEST 2019 Regional Congress Abstracts. Chest. 2019///. Conference::A327

Gestational Diabetes: Antenatal Care. #journal#. 2019///. #volume#:#pages#

Giles, Kristy. Breastfeeding and the Diabetic Woman. #journal#. 2018. #volume#:#pages#

H.K. Alkadhim,A.A.H. Albdairi. Surgical site infection after caesarean section in relation to operative time. Indian Journal of Forensic Medicine and Toxicology. 2019///. 13:625

Hayes L, Bell R, Robson S, Poston L, UPBEAT Consortium. Association between physical activity in obese pregnant women and offspring health. Pregnancy Hypertens 2014;4(3):234

I. Cetin,A. Laoreti. Role of Ultrasound Scan in Diabetic Pregnancy. Frontiers in Diabetes. 2019///. 28:155

Infant of a Woman with Diabetes: Management. #journal#. 2019///. #volume#:#pages#

J. Breckenkamp,O. Razum,W. Henrich,T. Borde,M. David. Effects of maternal obesity, excessive gestational weight gain and fetal macrosomia on the frequency of cesarean deliveries among migrant and non-migrant women - a prospective study. Journal of perinatal medicine. 2019/05/27/. 47:402

J. Lauenborg,M. Crusell,E.R. Mathiesen,P. Damm. Maternal Long-Term Outcomes after a Pregnancy Complicated by Gestational Diabetes Mellitus. Frontiers in Diabetes. 2019///. 28:223

J. Thereaux,T. Lesuffleur,S. Czernichow,A. Basdevant,S. Msika,D. Nocca,B. Millat,A. Fagot-Campagna. Long-term adverse events after sleeve gastrectomy or gastric bypass: a 7-year nationwide, observational, population-based, cohort study. The lancet.Diabetes & endocrinology. 2019/10//. 7:786

J.F. Mission,J. Catov,D. Comer,K.Z. Abebe,T.E. Deihl,M. Feghali,C.M. Scifres. Perinatal Outcomes Associated with Early Diabetes Testing in Pregnancies Complicated by Obesity. American journal of perinatology. 2019/03/20/. 2019 Mar 20:#pages#

J.M. Ferro,Sousa D. Aguiar de. Cerebral Venous Thrombosis: an Update. Current Neurology and Neuroscience Reports. 2019/08/23/. 19:74, 2019

J.M. Tovar Rodriguez,Molina L. Valle,V.M. Vargas Hernandez,F.J. Hernandez Aldana,L.E. Hernandez Vivar. Difference of the arterial pressure between arms in a group of pregnant and puerperal women with and without arterial hypertension and its impact on the newborn person. Clinica e Investigacion en Ginecologia y Obstetricia. 2019///. 46:69

K. Park,O. Quesada,G. Cook-Wiens,J. Wei,M. Minissian,E.M. Handberg,N.B. Merz,C.J. Pepine. Adverse Pregnancy Outcomes Are Associated with Reduced Coronary Flow Reserve in Women With Signs and Symptoms of Ischemia Without Obstructive Coronary Artery Disease: A Report from the Women's Ischemia Syndrome Evaluation-Coronary Vascular Dysfunction Study. Journal of women's health (2002). 2019/12/20/. 2019 Dec 20:#pages#

K. Szabelska-Zakrzewska,A. Durko,A. Socha-Banasiak,M. Majewska,M. Kolejwa,J. Kazanek-Zasada,E. Czkwianianc. Metabolic syndrome in overweight or obese children and adolescents based on own material Abstract Key words. Medycyna wieku rozwojowego. 2018///. 22:351

Kim SA, Park JB. OS 23-03 MID-TRIMESTER RISK PREDICTION OF SUPERIMPOSED PRE-ECLAMPSIA IN PREGNANT WOMEN WITH CHRONIC HYPERTENSION. J Hypertens 2016;34 Suppl 1 - ISH 2016 Abstract Book:e241

L. Widasari,M.T. Chalid,N. Jafar,A.R. Thaha. Effects of multimicronutrient and IFA supplementation in preconception period against birth length and birth weight: a randomized, double blind controlled trial in banggai regency, Central Sulawesi. Indian Journal of Public Health Research and Development. 2019///. 10:338

L.G.E. Hanem,O. Salvesen,P.B. Juliusson,S.M. Carlsen,M.C.F. Nossum,M.O. Vaage,R. Odegard,E. Vanky. Intrauterine metformin exposure and offspring cardiometabolic risk factors (PedMet study): a 5-10 year follow-up of the PregMet randomised controlled trial. The Lancet.Child & adolescent health. 2019/03//. 3:166

L.M. Semeniuk,V.K. Likhachov,T.Y. Yuzvenko,L.M. Dobrovolska,O.G. Makarov. Risk markers of reproductive loss in women with hyperandrogenism. Wiadomosci lekarskie (Warsaw, Poland : 1960). 2018///. 71:1550

Long Khanh-Dao Le. Antenatal: Routine Care. #journal#. 2019///. #volume#:#pages#

Long Khanh-Dao Le. Maternal: Postnatal Care. #journal#. 2019///. #volume#:#pages#

Lucylynn [PhD Lizarondo. Antenatal Care: Education for Healthy Lifestyle. #journal#. 2019///. #volume#:#pages#

M. Gallieni,M. Cozzolino. Uncomplicated central vein catheterization of high risk patients with real time ultrasound guidance. International Journal of Artificial Organs. 1995///. 18:117

M. Knight. UKOSS update. Obstetrician and Gynaecologist. 2019///. 21:223

M.O. Reis,Sousa T. Maia de,M.N.S. Oliveira,T.U. Maioli,L.C. Dos Santos. Factors Associated with Excessive Gestational Weight Gain Among Brazilian Mothers. Breastfeeding medicine : the official journal of the Academy of Breastfeeding Medicine. 2019/04//. 14:159

M.R. Altman,R.J. Baer,L.L. Jelliffe-Pawlowski. Patterns of Preterm Birth among Women of Native Hawaiian and Pacific Islander Descent. American journal of perinatology. 2019///. 36:1256

Madhava Sai [MBBS Student] Sivapuram. Congenital Hypothyroidism (Neonates): Detection and Treatment. #journal#. 2019///. #volume#:#pages#

Maier JT, Schalinski E, Gauger U, Hellmeyer L. Antenatal body mass index (BMI) and weight gain in pregnancy - its association with pregnancy and birthing complications. J Perinat Med 2016;44(4):397-404.

Manning S, Finer N, Elkalaawy M, Hashemi M, Jenkinson AD, Adamo M, et al. Timing of pregnancy in obese women after bariatric surgery. Pregnancy Hypertens 2014;4(3):235

Manzoor T, Baig MI, Ambreen A, Mushtaq R, Anwar K, Intsar A. Morbidity associated with obesity in pregnancy. Med Forum Monthly 2012;23(7):23-6.

Mariona FG. Proceedings of the XIIth World Congress of Perinatal Medicine, Madrid, Spain, 3-6 November 2015. Women's Health 2016;12(2):171-3. [PMID: http://www.futuremedicine.com/loi/whe]

Martin JC, Zhou SJ, Flynn AC, Malek L, Greco R, Moran L. The Assessment of Diet Quality and Its Effects on Health Outcomes Pre-pregnancy and during Pregnancy. Semin Reprod Med 2016;34(2):83-92.

N. Fleming,R. Cockerham. General anaesthesia for operative obstetrics. Anaesthesia and Intensive Care Medicine. 2019///. 20:495

N. Govender,J. Moodley,T. Naicker. Copeptin in preeclampsia development. Current Women's Health Reviews. 2019///. 15:159

N. Talank,E. Mirzaei,M. Mirjalili,M. Rangchian,Y. Mohammadi,M. Mehrpooya. Prevalence of dietary supplement use and its relation to maternal characteristics in iranian pregnant women. Current Women's Health Reviews. 2019///. 15:270

N.-A. Ankumah,M.A. Alrais,F.H. Amro,R.L. Wiley,B.M. Sibai. Prevalence and Risk Factors for New-Onset Hypertension in Labor. American journal of perinatology. 2019/03/20/. 16:#pages#

N.I. Parikh,B. Laria,G. Nah,M. Singhal,E. Vittinghoff,C. Vieten,N. Stotland,K. Coleman-Phox,N. Adler,M.A. Albert,E. Epel. Cardiovascular Disease-Related Pregnancy Complications Are Associated with Increased Maternal Levels and Trajectories of Cardiovascular Disease Biomarkers During and After Pregnancy. Journal of women's health (2002). 2020/01/14/. 2020 Jan 14:#pages#

Osorio J. Endocrine disorders in pregnancy: Excessive maternal weight increases risk of infant overgrowth. Nat Rev Endocrionol 2012;8(11):624

P. Hamet. The evaluation of the scientific evidence for a relationship between calcium and hypertension. Journal of Nutrition. 1995///. 125:311S

P. Hayward. Highlights from the 10th IAS Conference on Science. The Lancet HIV. 2019///. 6:e572

Papers and Abstracts of the 5 International Symposium on Women's Health Issues in Thrombosis and Haematosis. Thromb Res 2013;131:no

Parker CE, Doherty DA, Walters BN. PP030. Cardiovascular disease and risk in a pregnant woman's father as a risk factor for preeclampsia. Pregnancy Hypertens 2012;2(3):258

Proceedings of the Annual Meeting of Northern Obstetrical and Gynaecological Society of Scotland, NOGS 2013. J Obstet Gynaecol 2013;33(8):no

Sandi James. Shoulder Dystocia: Management. #journal#. 2019///. 78:#pages#

Scarpelini M, Sousa FLP, Garcia JM, Korkes HA, Aires FT, Paltronieri MRLN, et al. PP008. Lipotoxicity and preeclampsia: A probable correlation (preliminary results). Pregnancy Hypertens 2012;2(3):244

Sibai BM. Reproductive endocrinology: Predictive biomarkers of pre-eclampsia in women with T1DM. Nat Rev Endocrionol 2013;9(11):633-5.

Soomro S. Bano,R. Bosan,A.B. Shaikh,A.A. Shaikh,S. Shaikh. Frequency of pre-eclampsia in multigravida at Shaikh Zaid women hospital Larkana. Rawal Medical Journal. 2019///. 44:701

T. Premru-Srsen,Z. Kocic,Vodusek Fabjan,K. Gersak,I. Verdenik. Total gestational weight gain and the risk of preeclampsia by pre-pregnancy body mass index categories: a population-based cohort study from 2013 to 2017. Journal of perinatal medicine. 2019/08/27/. 47:585

T. Valadbeigi,H.R. Tabatabaee,K. Etemad,N. Keyghobadi,S. Mahdavi,M. Enayatrad,S. Saeidinejat,H. Yaghoobi,F. Zolfizadeh,A. Ghasemi,M. Hajipour. The association between low birth weight and mothers diseases in Iran: A case-control study. Journal of neonatal-perinatal medicine. 2019///. 12:449

van Poppel MNM, Ruchat SM, Mottola MF. Physical activity and gestational diabetes mellitus. Med sport sci 2014;60:104-12.

Vinturache, A., McKeating, A., Daly, N., Sheehan, S., and Turner, M.. Abstracts from the 16th International Society for Research in Human Milk and Lactation Conference, ISRHML 2012. Breastfeeding Medicine. 2012///. 7:2012

W.J. Su,Y.L. Chen,P.Y. Huang,X.L. Shi,F.F. Yan,Z. Chen,B. Yan,H.Q. Song,M.Z. Lin,X.J. Li. Effects of Prepregnancy Body Mass Index, Weight Gain, and Gestational Diabetes Mellitus on Pregnancy Outcomes: A Population-Based Study in Xiamen, China, 2011-2018. Annals of nutrition & metabolism. 2019///. 75:31

Whyte M, Pring C, Cooke D, Hart K, McGowan BM, Subramanian D, et al. Pregnancy after diabetes obesity surgery (PADOS): Incidence and outcomes. Pregnancy Hypertens 2014;4(3):239

Wong TY, Groen H, Faas MM, van Pampus MG. PP112. Prediction of preeclampsia based on clinical risk factors: A prospective high-risk cohort study. Pregnancy Hypertens 2012;2(3):300-1.

Wound Healing and Management Node Group and Gyi, Aye Aye MBBS MMedSc MPhil PhD. Venous Thromboembolism: Identification of Patients at Risk. #journal#. 2018. #volume#:#pages#

Zafari M, Kosarian M, Akbarzadeh H. Maternal obesity and pregnancy outcome. HealthMED 2012;6(9):3080-3. [PMID: http://www.drunpp.ba/pdf/healthmed_6_9_web.pdf]

Language

Flores-Padilla L, Solorio-Paez IC, Melo-Rey ML, Trejo-Franco J. Pregnancy and obesity: Risk of developing gestational diabetes in the northern border of Mexico. Gac Med Mex 2014;150:73-8. [PMID: http://www.medigraphic.com/pdfs/gaceta/gm-2014/gms141n.pdf]

Goncalves CV, Mendoza-Sassi RA, Cesar JA, de Castro NB, Bortolomedi AP. Body mass index and gestational weight gain as factors predicting complications and pregnancy outcome. Rev Bras Ginecol Obstet 2012;34(7):304-9. [PMID: http://www.scielo.br/pdf/rbgo/v34n7/03.pdf]

Kovacec S, Krajnc M, Cokolic M, Zavratnik A. Diabetes in pregnancy. Zdravniski Vestn 2012;81(10):745-52. [PMID: http://vestnik.szd.si/index.php/vestnik/article/view/1031/807]

Pandolfo MC, Rinoldo C, Catania M, Palumbo E, Puccio G, D'Anna MR. Maternal obesity and risk of preterm delivery. G Ital Ostet Ginecol 2014;36(1):235-9. [PMID: http://www.giog.it/common/php/portiere.php?ID=728be714e1d9ede1e241d8433106f21f]

Santos EMF, de Amorim LP, Costa OLN, Oliveira N, Guimaraes AC. Profile of gestational and metabolic risk in the prenatal care service of a public maternity in the Brazilian Northeast. Rev Bras Ginecol Obstet 2012;34(3):102-6. [PMID: http://www.scielo.br/pdf/rbgo/v34n3/a02v34n3.pdf]

Santos MMAS, Baiao MR, de Barros DC, Pinto AA, Pedrosa PM, Saunders C. Pre-pregnancy nutritional status, maternal weight gain, prenatal care, and adverse perinatal outcomes among adolescent mothers. Rev Bras Epidemiol 2012;15(1):143-54. [PMID: http://www.scielo.br/pdf/rbepid/v15n1/13.pdf]

V. Mladenovic,M. Dimitrijevic-Stojanovic,D. Macut,A. Djukic. Glycoregulation during pregnancy. Serbian Journal of Experimental and Clinical Research. 2019///. 20:9

Did not include obese pregnant women

A. Brembilla,N. Bernard,S. Pujol,A.L. Parmentier,A. Eckman,A.S. Mariet,H. Houot,Q. Tenailleau,G. Thiriez,D. Riethmuller,M. Barba-Vasseur,F. Mauny. Pregnancy vulnerability in urban areas: a pragmatic approach combining behavioral, medico-obstetrical, socio-economic and environmental factors. Sci Rep. 2019/12/11/. 9:18878, 2019

A. Fonseca,J. Lopes,N. Clode. Glucose intolerance in the third trimester is not predictive of adverse outcomes. International Journal of Gynecology and Obstetrics. 2019///. 147:108

A. Kautzky-Willer,J. Harreiter. The wave of sweetness and obesity continues. Nature Reviews Endocrinology. 2018///. 15:6

A. Magnusson,L. Nilsson,G. Olerod,A. Thurin-Kjellberg,C. Bergh. The addition of anti-Mullerian hormone in an algorithm for individualized hormone dosage did not improve the prediction of ovarian response-a randomized, controlled trial. Human reproduction (Oxford, England). 2017///. 32:811

A. Majewska,B. Godek,D. Bomba-Opon,M. Wielgos. Association between intrahepatic cholestasis in pregnancy and gestational diabetes mellitus. A retrospective analysis. Ginekologia polska. 2019///. 90:458

A. Ng,A. Liu,R. Nanan. Association between insulin and post-caesarean resuscitation rates in infants of women with GDM: A retrospective study. Journal of diabetes. 2020/02//. 12:151

A. Pujol,M.J. Zamora,A. Obradors,D. Garcia,A. Rodriguez,R. Vassena. Comparison of two different oocyte vitrification methods: A prospective, paired study on the same genetic background and stimulation protocol. Human Reproduction. 2019///. 34:989

A. Sirico,A. Lanzone,I. Mappa,L. Sarno,M. Slodki,D. Pitocco,F. Zullo,G.M. Maruotti,G. Rizzo. The role of first trimester fetal heart rate in the prediction of gestational diabetes: A multicenter study. European journal of obstetrics, gynecology, and reproductive biology. 2019/12//. 243:158

A. Zamora-Kapoor,K. Sinclair,L. Nelson,H. Lee,D. Buchwald. Obesity risk factors in American Indians and Alaska Natives: a systematic review. Public health. 2019///. 174:85

A.A. Muche,O.O. Olayemi,Y.K. Gete. Prevalence and determinants of gestational diabetes mellitus in Africa based on the updated international diagnostic criteria: a systematic review and meta-analysis. #journal#. 2019///. 77:36

A.C. Lee,A. Haddad,M.H. Fries,H.B. Al-Kouatly. Risk factors, clinical findings, and outcomes in pregnancies with coronary artery dissection: A case series. European journal of preventive cardiology. 2019///. 26:544

A.C. Staff. The two-stage placental model of preeclampsia: An update. Journal of reproductive immunology. 2019/09//. 134-135:1

A.D. George,M.C.L. Gay,M.E. Wlodek,D.T. Geddes. Breastfeeding a small for gestational age infant, complicated by maternal gestational diabetes: A case report. BMC pregnancy and childbirth. 2019///. 19:#pages#

A.S. Oyekale. Effect of Obesity and Other Risk Factors on Hypertension among Women of Reproductive Age in Ghana: An Instrumental Variable Probit Model. International journal of environmental research and public health. 2019/11/26/. 16:#pages#

A.T. Bawah,R.A. Ngala,H. Alidu,M.M. Seini,J.D.K. Wumbee,F.A. Yeboah. Gestational diabetes mellitus and obstetric outcomes in a Ghanaian community. The Pan African medical journal. 2019///. 32:94, 2019.:#pages#

A.T. Offianan,L.K. Penali,M.A. Coulibaly,N.L. Tiacoh,A.A.B. Ako,E.G. Adji,B. Coulibaly,D. Koffi,D. Sarr,R. Jambou,M. Kone. Comparative efficacy of uncontrolled and controlled intermittent preventive treatment during pregnancy (IPTp) with combined use of LLTNs in high resistance area to sulfadoxinepyrimethamine in Cote d'ivoire. #journal#. 2012///. 5:53

A.V. Skalny,A.A. Tinkov,T.G. Bohan,M.B. Shabalovskaya,O. Terekhina,S.B. Leshchinskaia,L.A. Agarkova,S.V. Notova,M.G. Skalnaya,Y. Kovas. The Impact of Maternal Overweight on Hair Essential Trace Element and Mineral Content in Pregnant Women and Their Children. Biological trace element research. 2020///. 193:64

Abou-Hussein S, Savona-Ventura C, Grima S, Felice A. Genetic factors in risk assessment for the development of type 2 diabetes mellitus in a small case series. INT J RISK SAF MED 2011;23(2):119-23.

Al RN, Al-Ruheili I, Al-Shezawi F, Al-Khabori M. Extreme preterm premature rupture of membranes: Risk factors and feto maternal outcomes. Oman med j 2013;28(2):108-11. [PMID: http://www.omjournal.org/fultext_PDF.aspx?DetailsID=352&pdf=images/352_M_Deatials_Pdf_.pdf&type=pdf]

Alanis MC, Villers MS, Law TL, Steadman EM, Robinson CJ. Complications of cesarean delivery in the massively obese parturient. Am J Obstet Gynecol 2010;203(3):271-7.

Alatishe A, Ammori BJ, New JP, Syed AA. Bariatric surgery in women of childbearing age. QJM 2013;106(8):717-20.

Alkasseh ASM, Zaki NM, Aljeesh YI, Soon LK. Risk factors of gestational diabetes mellitus in the refugee population in Gaza Strip: A case-control study. East Mediterr Health J 2013;19(SUPPL 3):S12-S18. [PMID: http://applications.emro.who.int/emhj/v19/Supp3/EMHJ_2013_19_Supp3_S12_S18.pdf]

Allen R, Rogozinska E, Sivarajasingam P, Khan KS, Thangaratinam S. Effect of diet- and lifestyle-based metabolic risk-modifying interventions on preeclampsia: a meta-analysis. Acta Obstet Gynecol Scand 2014;93(10):973-85.

Andraweera PH, Dekker GA, Jayasekara RW, Dissanayake VHW, Roberts CT. The obesity-related FTO gene variant associates with the risk of recurrent miscarriage. Acta Obstet Gynecol Scand 2015;94(7):722-6.

Andraweera PH, Dekker GA, Leemaqz S, McCowan L, Roberts CT, SCOPE consortium. The obesity associated FTO gene variant and the risk of adverse pregnancy outcomes: Evidence from the SCOPE study. Obesity (Silver Spring) 2016;24(12):2600-7.

Angeline G. Grace,Abhenil Mittal,Siddharth Jain,Jaya P. Tripathy,Srinath Satyanarayana,Prathap Tharyan,Richard Kirubakaran. Shortened treatment regimens versus the standard regimen for drug-sensitive pulmonary tuberculosis. Cochrane Database of Systematic Reviews 2019;(12). 2019/12/09/. 96:#pages#

Armanini D, Sabbadin C, Dona G, Andrisani A, Ambrosini G, Bordin L. Maternal and Fetal Outcomes in Preeclampsia: Interrelations Between Insulin Resistance, Aldosterone, Metabolic Syndrome, and Polycystic Ovary Syndrome. J Clin Hypertens 2015;17(10):783-5. [PMID: http://onlinelibrary.wiley.com/journal/10.1111/(ISSN)1751-7176]

Arne Ohlsson,Prakeshkumar S. Shah. Paracetamol (acetaminophen) for prevention or treatment of pain in newborns. Cochrane Database of Systematic Reviews 2020;(1). 2020/01/27/. 67:#pages#

Arne Ohlsson,Prakeshkumar S. Shah. Paracetamol (acetaminophen) for patent ductus arteriosus in preterm or low birth weight infants. Cochrane Database of Systematic Reviews 2020;(1). 2020/01/27/. 46:#pages#

Ay L, Kruithof CJ, Bakker R, Steegers EAP, Witteman JCM, Moll HA, et al. Maternal anthropometrics are associated with fetal size in different periods of pregnancy and at birth. the generation R study. BJOG Int J Obstet Gynaecol 2009;116(7):953-63.

Aydin C, Baloglu A, Yavuzcan A, Inci A. The effect of body mass index value during labor on pregnancy outcomes in Turkish population (obesity and pregnancy outcomes). Arch Gynecol Obstet 2010;281(1):49-54.

Aye ILMH, Jansson T, Powell TL. TNF-alpha stimulates System A amino acid transport in primary human trophoblast cells mediated by p38 MAPK signaling. Physiol Rep 2015;3(10):

B. Bassaw,H. Fletcher,C. Rattray,G. McIntyre,V. Sarkharkar,S. Sankat,A. Sirjusingh,J. Chinnia. Screening for gestational diabetes mellitus: a Caribbean perspective. Journal of Obstetrics and Gynaecology. 2018///. 38:1035

B. Cham,S. Scholes,L.N. Fat,O. Badjie,J.S. Mindell. Burden of hypertension in The Gambia: Evidence from a national World Health Organization (WHO) STEP survey. International journal of epidemiology. 2018///. 47:860

B. Lisowska-Myjak,A. Puchalska,N. Halasa,M. Plazinska,A. Strawa. The association between clinical and laboratory parameters in thyroid disease and nonthyroidal illness in young women. European review for medical and pharmacological sciences. 2019///. 23:2950

B. Moradi,A. Ghanbari,M. Rahmani,M.A. Kazemi,A.-R. Tahmasebpour,M. Shakiba. Evaluation of bi-iliac distance and timing of ossification of sacrum by sonography in the second trimester of pregnancy. Iranian Journal of Radiology. 2019///. 16:#pages#

B. Thilaganathan,E. Kalafat. Cardiovascular system in preeclampsia and beyond. Hypertension (Dallas, Tex.: 1979). 2019///. 73:522

B.K. Natamba,A.A. Namara,M.J. Nyirenda. Burden, risk factors and maternal and offspring outcomes of gestational diabetes mellitus (GDM) in sub-Saharan Africa (SSA): a systematic review and meta-analysis. BMC pregnancy and childbirth. 2019///. 19:#pages#

Badon SE, Wartko PD, Qiu C, Sorensen TK, Williams MA, Enquobahrie DA. Leisure Time Physical Activity and Gestational Diabetes Mellitus in the Omega Study. Med Sci Sports Exerc 2016;48(6):1044-52.

Bahadoer S, Gaillard R, Felix JF, Raat H, Renders CM, Hofman A, et al. Ethnic disparities in maternal obesity and weight gain during pregnancy. The Generation R Study. Eur J Obstet Gynecol Reprod Biol 2015;193:51-60.

Bartnik P, Kosinska-Kaczynska K, Kacperczyk J, Ananicz W, Sierocinska A, Wielgos M, et al. Twin Chorionicity and the Risk of Hypertensive Disorders: Gestational Hypertension and Pre-eclampsia. Twin Res Hum Genet 2016;19(4):377-82.

Baugh N, Harris DE, Aboueissa AM, Sarton C, Lichter E. The Impact of Maternal Obesity and Excessive Gestational Weight Gain on Maternal and Infant Outcomes in Maine: Analysis of Pregnancy Risk Assessment Monitoring System Results from 2000 to 2010. J Pregnancy 2016;2016:5871313

Bej P, Chhabra P, Sharma AK, Guleria K. Determination of Risk Factors for Pre-eclampsia and Eclampsia in a Tertiary Hospital of India: A Case Control Study. J family med prim 2013;2(4):371-5.

Belcastro MR, Neiger R, Ventolini G. Intrauterine growth restriction after bariatric surgery. J Neonatal Perinatal Med 2011;4(3):231-4.

Berlac JF, Skovlund CW, Lidegaard O. Obstetrical and neonatal outcomes in women following gastric bypass: a Danish national cohort study. Acta Obstet Gynecol Scand 2014;93(5):447-53.

Bisson M, Series F, Giguere Y, Pamidi S, Kimoff J, Weisnagel SJ, et al. Gestational diabetes mellitus and sleep-disordered breathing. Obstet Gynecol 2014;123(3):634-41.

Bogaerts A, Van den Bergh BRH, Ameye L, Witters I, Martens E, Timmerman D, et al. Interpregnancy weight change and risk for adverse perinatal outcome. Obstet Gynecol 2013;122(5):999-1009.

Bourjeily G. Sleep disorders in pregnancy. Obstet med 2009;2(3):100-6.

Bouthoorn SH, Silva LM, Murray SE, Steegers EAP, Jaddoe VWV, Moll H, et al. Low-educated women have an increased risk of gestational diabetes mellitus: the Generation R Study. Acta Diabetol 2015;52(3):445-52.

Brannon PM. Key questions in Vitamin D research. Scand J Clin Lab Invest 2012;72(SUPPL. 243):154-62.

Brink HS, van der Lely AJ, van der Linden J. The potential role of biomarkers in predicting gestational diabetes. Endocr connect 2016;5(5):R26-R34

C. Blegvad,Andersen A.-M. Nybo,A. Adam,C. Zachariae,L. Skov. Psoriasis as a predictor of cardiometabolic comorbidity in women: A study based on the Danish national birth cohort. Acta Dermato-Venereologica. 2019///. 99:274

C. Castano,A. Novials,M. Parrizas. Exosomes and diabetes. Diabetes/metabolism research and reviews. 2019///. 35:#pages#

C. Delgado-Sanz,C. Mazagatos-Ateca,J. Oliva,A. Gherasim,A. Larrauri. Illness severity in hospitalized influenza patients by virus type and subtype, Spain, 2010-2017. Emerging infectious diseases. 2020///. 26:220

C. McCormack,S. Leemaqz,D. Furness,G. Dekker,C. Roberts. Association between vitamin D status and hyperinsulinism. Journal of Maternal-Fetal and Neonatal Medicine. 2019///. 32:4005

C. Rarrick,N. Saccone,A. Hebbard,B. Jones. Assessing the Rate of Antipsychotic Use in Ambulatory Care Patients With a Venous Thromboembolism. Annals of Pharmacotherapy. 2020/02//. 54:97

C. Snehalatha,S. Priscilla,A. Nanditha,R. Arun,K. Satheesh,A. Ramachandran. Metformin in Prevention of Type 2 Diabetes. The Journal of the Association of Physicians of India. 2018///. 66:60

C. Sucker. Prophylaxis and Therapy of Venous Thrombotic Events (VTE) in Pregnancy and the Postpartum Period. Geburtshilfe und Frauenheilkunde. 2020/01//. 80:48

C.A. Crowther,L.W. Doyle,R.R. Haslam,J.E. Hiller,J.E. Harding,J.S. Robinson,ACTORDS Study Group. Outcomes at 2 years of age after repeat doses of antenatal corticosteroids. New England Journal of Medicine. 2007///. 357:1179

C.B. Shiferaw,W.W. Yallew,G.T. Tiruneh. Maternal Anthropometric Measurements Do Not Have Effect on Birth Weight of Term, Single, and Live Births in Addis Ababa City, Ethiopia. Journal of pregnancy. 2018///. 2018:1982134, 2018.:#pages#

C.E. Hernandez-Rodriguez,C.M. Estrada-Zuniga,la O.-C. De,F. Garcia-Rodriguez,I. Rodriguez-Balderrama,C.A. Zapata-Castilleja,C. Trevino-Garza. Differences in omentin-1 levels in term newborns according to birth weight. Early human development. 2019/12//. 139:104842

C.J. Petry,B.G. Fisher,K.K. Ong,I.A. Hughes,C.L. Acerini,D.B. Dunger. Temporal trends without seasonal effects on gestational diabetes incidence relate to reductions in indices of insulin secretion: the Cambridge Baby Growth Study. Acta diabetologica. 2019///. 56:1133

C.M. Jacob,M.L. Newell,M. Hanson. Narrative review of reviews of preconception interventions to prevent an increased risk of obesity and non-communicable diseases in children. Obesity reviews : an official journal of the International Association for the Study of Obesity. 2019/08//. 20 Suppl 1:5

C.M. Phillips,L.-W. Chen,B. Heude,J.Y. Bernard,N.C. Harvey,L. Duijts,S.M. Mensink-Bout,K. Polanska,G. Mancano,M. Suderman,N. Shivappa,J.R. Hebert. Dietary inflammatory index and non-communicable disease risk: A narrative review. Nutrients. 2019///. 11:#pages#

C.R. Taylor,J.E. Dominguez,A.S. Habib. Obesity And Obstetric Anesthesia: Current Insights. Local and regional anesthesia. 2019///. 12:111

Campbell KH, Savitz D, Werner EF, Pettker CM, Goffman D, Chazotte C, et al. Maternal morbidity and risk of death at delivery hospitalization. Obstet Gynecol 2013;122(3):627-33.

Can MM, Can E, Ozveren O, Okuyan E, Ayca B, Dinckal MH. Epicardial fat tissue thickness in preeclamptic and normal pregnancies. ISRN Obstet Gynecol 2012;no

Caroline S. Costa,Edileia Bagatin,Luiza Ana Martimbianco,MK Edina da Silva,Marilia M. Lucio,Parker Magin,Rachel Riera. Oral isotretinoin for acne. Cochrane Database of Systematic Reviews 2019;(2). 2019/02/20/. 121:#pages#

Cerqueira Heloisa Villar,Humberto Saconato,Orsine Valente,Alvaro N. Atallah. Thyroid hormone replacement for subclinical hypothyroidism. Cochrane Database of Systematic Reviews. 2008/11/02/. 21:#pages#

Chaar B. The prevalence and management of gestational diabetes. Aust J Pharm 2009;90(1):68-70.

Chee Keong.Turnbull See. Association of endogenous testosterone concentration with depression in men: a systematic review protocol. #journal#. 2019///. 78:#pages#

Chen Q, Wei J, Tong M, Yu L, Lee AC, Gao YF, et al. Associations between body mass index and maternal weight gain on the delivery of LGA infants in Chinese women with gestational diabetes mellitus. J Diabetes Complications 2015;29(8):1037-41.

Chiavaroli V, Castorani V, Guidone P, Derraik JGB, Liberati M, Chiarelli F, et al. Incidence of infants born small- and large-for-gestational-age in an Italian cohort over a 20-year period and associated risk factors. Ital J Pediatr 2016;42:42

Cho EH, Hur J, Lee KJ. Early Gestational Weight Gain Rate and Adverse Pregnancy Outcomes in Korean Women. PloS one 2015;10(10):e0140376

Cho GJ, Park JH, Lee H, Yoo S, Shin SA, Oh MJ. Prepregnancy Factors as Determinants of the Development of Diabetes Mellitus After First Pregnancy. J Clin Endocrinol Metab 2016;101(7):2923-30.

Choi SK, Lee G.. Determining optimal gestational weight gain in the Korean population: a retrospective cohort study. #journal#. 2017. 15:#pages#

Chrelias G, Makris GM, Papanota AM, Spathis A, Salamalekis G, Sergentanis TN, et al. Serum inhibin and leptin: Risk factors for pre-eclampsia? Clin Chim Acta 2016;463:84-7.

Cinar M, Timur H, Aksoy RT, Guzel AI, Tokmak A, Bedir Findik R, et al. Evaluation of maternal and perinatal outcomes among overweight women who experienced stillbirth. J Matern Fetal Neonatal Med 2016;1-20.

Cohen JL, Smilen KE, Bianco AT, Moshier EL, Ferrara LA, Stone JL. Predictive value of combined serum biomarkers for adverse pregnancy outcomes. Eur J Obstet Gynecol Reprod Biol 2014;181:89-94.

Coutinho PR, Cecatti JG, Surita FG, Souza JPd, Morais SSd. Factors associated with low birth weight in a historical series of deliveries in Campinas, Brazil. Rev Assoc Med Bras 2009;55(6):692-9.

Cruz J, Grandia R, Padilla L, Rodriguez S, Hernandez Garcia P, Lang Prieto J, et al. Macrosomia Predictors in Infants Born to Cuban Mothers with Gestational Diabetes. MEICC REV 2015;17(3):27-32.

D. Armanini,A. Andrisani,G. Ambrosini,G. Dona,L. Bordin,C. Sabbadin. Hypertension in pregnancy: Role of body mass index, insulin resistance, aldosterone, and calcium homeostasis. Journal of Clinical Hypertension. 2019///. 21:624

D. Leger,Y. Esquirol,C. Gronfier,A. Metlaine. Reprint of: Shift-workers and night-workers' health consequences: State of art and recommendations. Medecine du Sommeil. 2019///. 16:191

D. Mazurek,K. Lozna,M. Bronkowska. The concentration of selected elements in the placenta according to selected sociodemographic factors and their effect on birth mass and birth length of newborns. Journal of Trace Elements in Medicine and Biology. 2020///. 58:126425

D. Santoro,Bella G. Di,A. Toscano,O. Musumeci,M. Buemi,G.B. Piccoli. Mitochondrial Disease (MELAS Syndrome) Discovered at the Start of Pregnancy in a Patient with Advanced CKD: A Clinical and Ethical Challenge. #journal#. 2019/03/04/. 8:#pages#

D.A.M. Utomo,V. Andriolo,T. Barnighausen,I. Danquah. Linking malaria in pregnancy with dietary behavior of the next generation. Brain, behavior, and immunity. 2019///. 80:1

D.M. Glodean,D. Miclea,G. Zaharie,J.M. Mihaila,A.R. Popa. Observational Case-Control Study on the Risk Factors of Fetal Macrosomia and Fetal-Maternal Associated Pathology. Romanian Journal of Diabetes, Nutrition and Metabolic Diseases. 2019///. 26:11

Dantas EMDM, Pereira FVM, Queiroz JW, Dantas DLDM, Monteiro GRG, Duggal P, et al. Preeclampsia is associated with increased maternal body weight in a northeastern Brazilian population. BMC Pregnancy Childbirth 2013;13:no. [PMID: http://www.biomedcentral.com/1471-2393/13/159]

Dayan N, Pilote L, Opatrny L, Basso O, Messerlian C, El-Messidi A, et al. Combined impact of high body mass index and in vitro fertilization on preeclampsia risk: a hospital-based cohort study. Obesity (Silver Spring) 2015;23(1):200-6.

Dayan N, Pilote L, Opatrny L, Daskalopoulou SS. Assisted Reproductive Therapy in Women With Higher Body Mass Index. J Obstet Gynaecol Can 2014;36(6):513-4. [PMID: http://www.journals.elsevier.com/journal-of-obstetrics-and-gynaecology-canada/]

De Souza LR, Berger H, Retnakaran R, Maguire JL, Nathens AB, Connelly PW, et al. First-Trimester Maternal Abdominal Adiposity Predicts Dysglycemia and Gestational Diabetes Mellitus in Midpregnancy. Diabetes care 2016;39(1):61-4.

Ding XX, Xu SJ, Hao JH, Huang K, Su PY, Tao FB. Maternal pre-pregnancy BMI and adverse pregnancy outcomes among Chinese women: Results from the C-ABCS. J Obstet Gynaecol 2016;36(3):328-32.

Drife J. Risk factors for maternal death revisited. BJOG Int J Obstet Gynaecol 2016;123(10):1663. [PMID: http://onlinelibrary.wiley.com/journal/10.1111/(ISSN)1471-0528]

Duncan C. Highlights from the fifth international symposium on diabetes and pregnancy. Br J Diabetes Vasc Dis 2009;9(3):139-40.

E. Carmina,R. Azziz,W. Bergfeld,H.F. Escobar-Morreale,W. Futterweit,H. Huddleston,R. Lobo,E. Olsen. Female Pattern Hair Loss and Androgen Excess: A Report from the Multidisciplinary Androgen Excess and PCOS Committee. Journal of Clinical Endocrinology and Metabolism. 2019///. 104:2875

E. Galasso,A.M. Weber,C.P. Stewart,L. Ratsifandrihamanana,L.C.H. Fernald. Effects of nutritional supplementation and home visiting on growth and development in young children in Madagascar: a cluster-randomised controlled trial. The Lancet Global Health. 2019///. 7:e1257

E.G. Radke,A. Galizia,K.A. Thayer,G.S. Cooper. Phthalate exposure and metabolic effects: a systematic review of the human epidemiological evidence. Environment international. 2019///. 132:104768

E.L. Rauff,D.S. Downs. Mobile Health Technology in Prenatal Care: Understanding OBGYN Providers' Beliefs about Using Technology to Manage Gestational Weight Gain. #journal#. 2019/03//. 4:17

Ebert T, Platz M, Kralisch S, Lossner U, Jessnitzer B, Richter J, et al. Serum Levels of Copeptin are Decreased in Gestational Diabetes Mellitus. Exp Clin Endocrinol Diabetes 2016;124(4):257-60.

Edalat B, Sharifi F, Badamchizadeh Z, Hossein-Nezhad A, Larijani B, Mirarefin M, et al. Association of metabolic syndrome with inflammatory mediators in women with previous gestational diabetes mellitus. J diabetes metab disord 2013;12(1):8

Edison E, Whyte M, van Vlymen J, Jones S, Gatenby P, de Lusignan S, et al. Bariatric Surgery in Obese Women of Reproductive Age Improves Conditions That Underlie Fertility and Pregnancy Outcomes: Retrospective Cohort Study of UK National Bariatric Surgery Registry (NBSR). Obes Surg 2016;26(12):2837-42.

Ehrenberg HM, Iams JD, Goldenberg RL, Newman RB, Weiner SJ, Sibai BM, et al. Maternal obesity, uterine activity, and the risk of spontaneous preterm birth. Obstet Gynecol 2009;113(1):48-52.

Ehrlich SF, Sternfeld B, Krefman AE, Hedderson MM, Brown SD, Mevi A, et al. Moderate and Vigorous Intensity Exercise During Pregnancy and Gestational Weight Gain in Women with Gestational Diabetes. Matern Child Health J 2016;20(6):1247-57.

Elizondo-Montemayor L, Hernandez-Escobar C, Lara-Torre E, Nieblas B, Gomez-Carmona M. Gynecologic and Obstetric Consequences of Obesity in Adolescent Girls. J Pediatr Adolesc Gynecol 2016;

Endeshaw M, Abebe F, Worku S, Menber L, Assress M, Assefa M. Obesity in young age is a risk factor for preeclampsia: A facility based case-control study, northwest Ethiopia. BMC Pregnancy Childbirth 2016;16(1):no. [PMID: http://www.biomedcentral.com/bmcpregnancychildbirth/]

Enquobahrie DA, Wander PL, Tadesse MG, Qiu C, Holzman C, Williams MA. Maternal pre-pregnancy body mass index and circulating microRNAs in pregnancy. Obes Res Clin Pract 2016;

Everett TR, Mahendru A, McEniery CM, Lees CC, Wilkinson IB. A comparison of SphygmoCor and Vicorder devices for measuring aortic pulse wave velocity in pregnancy. Artery Res 2012;6(2):92-6.

F. Dewan,M.A. Chowdhury,K. Nessa. Thrombophilia in pregnancy and puerperium. Bangladesh Journal of Medical Science. 2019///. 18:178

F. Foroozanfard,Z. Asemi,F. Bazarganipour,S.A. Taghavi,H. Allan,S. Aramesh. Comparing pregnancy, childbirth, and neonatal outcomes in women with different phenotypes of polycystic ovary syndrome and healthy women: a prospective cohort study. Gynecological endocrinology : the official journal of the International Society of Gynecological Endocrinology. 2020/01//. 36:61

F. Gu,S. Li,L. Zheng,J. Gu,T. Li,H. Du,C. Gao,C. Ding,S. Quan,C. Zhou,P. Li,Y. Xu. Perinatal outcomes of singletons following vitrification versus slow-freezing of embryos: a multicenter cohort study using propensity score analysis. Human Reproduction. 2019/09/29/. 34:1788

F.A. Rasheed,R.H. Mshattat,U.M. Alnakkash,S.A. Hussain. Hypertriglyceridemia and waist phenotype as markers in the prediction of gestational diabetes in Iraqi women. Research Journal of Obstetrics and Gynecology. 2018///. 11:25

F.M. Brown,E. Isganaitis,T. James-Todd. Much to hapo Fus about: Increasing maternal glycemia in pregnancy is associated with worsening childhood glucose metabolism. Diabetes care. 2019///. 42:393

F.M. Brown,E. Isganaitis,T. James-Todd. Much to hapo Fus about: Increasing maternal glycemia in pregnancy is associated with worsening childhood glucose metabolism. Diabetes care. 2019///. 42:393

F.T. Spradley,A.C. Palei,C.D. Anderson,J.P. Granger. Melanocortin-4 Receptor Deficiency Attenuates Placental Ischemia-Induced Hypertension in Pregnant Rats. Hypertension (Dallas, Tex. 2019///. ::162

Fadl HE, Simmons D. Trends in diabetes in pregnancy in Sweden 1998-2012. BMJ open diabetes res care 2016;4(1):e000221

Favre L, Clarisse M, Pralong FP, Suter M, Fournier P, Baud D. [Pregnancy after bariatric surgery]. Rev Med Suisse 2016;12(511):606-10.

Fitzpatrick KE, Tuffnell D, Kurinczuk JJ, Knight M. Pregnancy at very advanced maternal age: a UK population-based cohort study. BJOG 2016;

Fujimoto W, Samoa R, Wotring A. Gestational diabetes in high-risk populations. Clin Diabetes 2013;31(2):90-4. [PMID: http://clinical.diabetesjournals.org/content/31/2/90.full.pdf+html]

Fuller-Tyszkiewicz M, Skouteris H, Hill B, Teede H, McPhie S. Classification tree analysis of postal questionnaire data to identify risk of excessive gestational weight gain. Midwifery 2016;32:38-44.

G. Aynaci,Z. Guksu. The effects of dietary folate and iron supplementation on restless legs and preeclampsia in pregnancy. Progress in Nutrition. 2019///. 21:398

G. Daniele,A. Tura,A. Dardano,A. Bertolotto,C. Bianchi,L. Giusti,J.J. Kurumthodathu,Prato S. Del. Effects of Treatment With Metformin and/or Sitagliptin on Beta-cell Function and Insulin Resistance in Prediabetic Women With Previous Gestational Diabetes. Diabetes, Obesity and Metabolism. 2019///. 2019.:#pages#

G. Sanabria-Martinez,R. Poyatos-Leon,B. Notario-Pacheco,C. Alvarez-Bueno,I. Cavero-Redondo,V. Martinez-Vizcaino. Effects of physical exercise during pregnancy on mothers' and neonates' health: a protocol for an umbrella review of systematic reviews and meta-analysis of randomised controlled trials. BMJ open. 2019/09/13/. 9:e030162, 2019

G.A. Foratori-Junior,B.M. da Silva,A.C. da Silva Pinto,H.M. Honorio,F.C. Groppo,de Carvalho Sales-Peres SH. Systemic and periodontal conditions of overweight/obese patients during pregnancy and after delivery: a prospective cohort. Clin Oral Investig. 2020/01//. 24:157

G.F. Mattina,R.J. Van Lieshout,M. Steiner. Inflammation, depression and cardiovascular disease in women: The role of the immune system across critical reproductive events. Therapeutic Advances in Cardiovascular Disease. 2019///. 13:1

G.M.H. Swaen,P. Boffetta,M. Zeegers. Impact of changes in human reproduction on the incidence of endocrine-related diseases. Critical reviews in toxicology. 2018///. 48:789

G.P. Mensah,D.R.M. van Rooyen,W. Ten Ham-Baloyi. Nursing management of gestational diabetes mellitus in Ghana: Perspectives of nurse-midwives and women. Midwifery. 2019/04//. 71:19

Gaillard R, Bakker R, Steegers EAP, Hofman A, Jaddoe VWV. Maternal age during pregnancy is associated with third trimester blood pressure level: the generation R study. Am J Hypertens 2011;24(9):1046-53.

Ge X, Tao F, Huang K, Mao L, Huang S, Niu Y, et al. Maternal Snoring May Predict Adverse Pregnancy Outcomes: A Cohort Study in China. PloS one 2016;11(2):e0148732

Gibbs B. Barone,J.L. Paley,M.A. Jones,K.M. Whitaker,C.P. Connolly,J.M. Catov. Validity of self-reported and objectively measured sedentary behavior in pregnancy. BMC pregnancy and childbirth. 2020/02/11/. 20:99, 2020

Gill SK. Cardiovascular risk factors and disease in women. Med Clin North Am 2015;99(3):535-52. [PMID: http://www.medical.theclinics.com/]

Gur EB, Ince O, Turan GA, Karadeniz M, Tatar S, Celik E, et al. Ultrasonographic visceral fat thickness in the first trimester can predict metabolic syndrome and gestational diabetes mellitus. Endocrine 2014;47(2):478-84.

Gyselaers W, Martens G. Increasing prevalence of macrosomia in Flanders, Belgium: an indicator of population health and a burden for the future. Facts views vis ObGyn 2012;4(2):141-3.

H. Rashid,E. Ma,F. Ferdous,E.C. Ekstrom,Y. Wagatsuma. First-trimester fetal growth restriction and the occurrence of miscarriage in rural Bangladesh: a prospective cohort study. PloS one. 2017///. 12:e0181967, 2017

H. Skouteris,H.J. Teede,S. Thangaratinam,C. Bailey,J.-A. Baxter,H.J. Bergmeier,C. Harrison,B. Hill,B. Jack,L. Jorgensen,S. Lim,T. Matsaseng,C. Montanaro,E. Steegers,J. Stephenson,H. Sundseth,A.L.V. Borges,R. Walker,L. Redman,J. Boyle. Commentary: Obesity and weight gain in pregnancy and

postpartum: An evidence review of lifestyle interventions to inform maternal and child health policies. Frontiers in endocrinology. 2019/03//. 10:#pages#

H. Yaribeygi,T. Sathyapalan,A. Sahebkar. Molecular mechanisms by which GLP-1 RA and DPP-4i induce insulin sensitivity. Life sciences. 2019///. 234:116776

H. Zhang,Y. Zhang,Z. Wang,J. Yan. Platelet count and mean platelet volume predict atypical pre-eclampsia. Pregnancy hypertension. 2019///. 18:29

H.F. Huber,A.H. Kuo,C. Li,S.L. Jenkins,K.G. Gerow,G.D. Clarke,P.W. Nathanielsz. Antenatal Synthetic Glucocorticoid Exposure at Human Therapeutic Equivalent Doses Predisposes Middle-Age Male Offspring Baboons to an Obese Phenotype That Emerges With Aging. Reproductive Sciences. 2019///. 26:591

H.L. Barrett,K.L. Gatford,C.M. Houda,M.J. De Blasio,H.D. McIntyre,L.K. Callaway,Nitert M. Dekker,S. Coat,J.A. Owens,W.M. Hague,J.A. Rowan. Maternal and neonatal circulating markers of metabolic and cardiovascular risk in the metformin in gestational diabetes (MiG) trial: responses to maternal metformin versus insulin treatment. Diabetes care. 2013///. 36:529

H.M. Picton,A.H. Balen. Transgenerational PCOS transmission. Nature Medicine. 2019///. 25:1818

H.M. Salihu,D. Dongarwar,L.M. King,K.K. Yusuf,S. Ibrahimi,A.A. Salinas-Miranda. Trends in the incidence of fetal macrosomia and its phenotypes in the United States, 1971-2017. Archives of gynecology and obstetrics. 2020/02//. 301:415

Han AR, Kim HO, Cha SW, Park CW, Kim JY, Yang KM, et al. Adverse pregnancy outcomes with assisted reproductive technology in non-obese women with polycystic ovary syndrome: a case-control study. Clin exp reprod med 2011;38(2):103-8.

Han SM, Kim WW, Moon R, Rosenthal RJ. Pregnancy outcomes after laparoscopic sleeve gastrectomy in morbidly obese Korean women. Obes Surg 2013;23(6):756-9.

Hankey CR. Obesity and Maternal Weight Gain. Curr obes rep 2015;4(1):60-4.

Harville EW, Juonala M, Viikari JSA, Raitakari OT. Preconception metabolic indicators predict gestational diabetes and offspring birthweight. Gynecol Endocrinol 2014;30(11):840-4.

Hayes C. Long-term prognostic factors in the diagnosis of gestational diabetes. Br J Nurs 2009;18(9):523-6.

Hayes-Ryan D, Byrne BM. Prevention of thrombosis in pregnancy: How practical are consensus derived clinical practice guidelines? J Obstet Gynaecol 2012;32(8):740-2.

Hedderson MM, Darbinian J, Havel PJ, Quesenberry CP, Sridhar S, Ehrlich S, et al. Low prepregnancy adiponectin concentrations are associated with a marked increase in risk for development of gestational diabetes mellitus. Diabetes care 2013;36(12):3930-7.

Heidema WM, Scholten RR, Lotgering FK, Spaanderman MEA. History of preeclampsia is more predictive of cardiometabolic and cardiovascular risk factors than obesity. Eur J Obstet Gynecol Reprod Biol 2015;194:189-93.

Hemond J, Robbins RB, Young PC. The Effects of Maternal Obesity on Neonates, Infants, Children, Adolescents, and Adults. Clin Obstet Gynecol 2016;59(1):216-27.

Hernandez Hernandez JD, Villasenor OR, Del Rio Alvarado J, Lucach RO, Zarate A, Saucedo R, et al. Morphological changes of red blood cells in peripheral blood smear of women with pregnancy-related hypertensive disorders. Arch Med Res 2015;46(6):479-83.

Horan MK, McGowan CA, Gibney ER, Donnelly JM, McAuliffe FM. The association between maternal dietary micronutrient intake and neonatal anthropometry - secondary analysis from the ROLO study. Nutr J 2015;14:105

Huang QT, Zhang M, Zhong M, Yu YH, Liang WZ, Hang LL, et al. Advanced glycation end products as an upstream molecule triggers ROS-induced sFlt-1 production in extravillous trophoblasts: a novel bridge between oxidative stress and preeclampsia. Placenta 2013;34(12):1177-82.

I. Iguacel,A. Chung,E. Gearon,L.A. Moreno,A. Peeters,K. Backholer. Influence of early-life risk factors on socioeconomic inequalities in weight gain. Journal of public health (Oxford, England). 2018///. 40:e447

I. Lee,K.S. Bang,H. Moon,J. Kim. Risk Factors for Obesity Among Children Aged 24 to 80 months in Korea: A Decision Tree Analysis. Journal of Pediatric Nursing. 2019/05//. 46:e15

I. Szymusik,K. Kosinska-Kaczynska,M. Krowicka,M. Sep,P. Marianowski,M. Wielgos. Perinatal outcome of in vitro fertilization singletons - 10 years' experience of one center. Archives of medical science : AMS. 2019/05//. 15:666

I.A. Ba-Saddik,T.O. Al-Asbahi. Anthropometric measurements of singleton live full-term newborns in Aden, Yemen. International Journal of Pediatrics and Adolescent Medicine. 2019///. 64:#pages#

I.W. Yen,C.N. Lee,M.W. Lin,K.C. Fan,J.N. Wei,K.Y. Chen,S.C. Chen,Y.Y. Tai,C.H. Kuo,C.H. Lin,C.Y. Hsu,L.M. Chuang,S.Y. Lin,H.Y. Li. Overweight and obesity are associated with clustering of metabolic risk factors in early pregnancy and the risk of GDM. PloS one. 2019///. 14:e0225978, 2019

Ianniello F, Quagliozzi L, Caruso A, Paradisi G. Low adiponectin in overweight/obese women: association with diabetes during pregnancy. Eur Rev Med Pharmacol Sci 2013;17(23):3197-205.

Ibrahim MH, Moustafa AN, Saedii AAF, Hassan EE. Cord blood erythropoietin and cord blood nucleated red blood cells for prediction of adverse neonatal outcome associated with maternal obesity in term pregnancy: prospective cohort study. J Matern -Fetal Neonatal Med 2016;1-6.

Ilaria Casetta,Gerardo Iuliano,Graziella Filippini. Azathioprine for multiple sclerosis. Cochrane Database of Systematic Reviews. 2008/11/10/. 49:#pages#

Imoh LC, Ocheke AN. Correlation between maternal weight and insulin resistance in second half of pregnancy. Niger Med J 2014;55(6):465-8.

Irina Benenson. Risk factors for hypertensive crisis in adult patients: a systematic review protocol. #journal#. 2019///. 79:#pages#

Izci Balserak B. Sleep disordered breathing in pregnancy. Breathe 2015;11(4):268-77.

J. Dereke,C. Nilsson,H. Strevens,M. Landin-Olsson,M. Hillman. Pregnancy-associated plasma protein-A2 levels are increased in early-pregnancy gestational diabetes: a novel biomarker for early risk estimation. Diabetic medicine : a journal of the British Diabetic Association. 2020/01//. 37:131

J. Liu,G. Song,T. Meng,G. Zhao,S. Guo. Weight retention at six weeks postpartum and the risk of gestational diabetes mellitus in a second pregnancy. BMC pregnancy and childbirth. 2019/08/01/. 19:272, 2019

J. Luo,A. Hodge,M. Hendryx,J.E. Byles. Age of obesity onset, cumulative obesity exposure over early adulthood and risk of type 2 diabetes. Diabetologia. 2020///. 63:519

J. Polivka,M. Pesta,V. Rohan,L. Celedova,S. Mahajani,O. Topolcan,O. Golubnitschaja. Risks associated with the stroke predisposition at young age: facts and hypotheses in light of individualized predictive and preventive approach. EPMA Journal. 2019///. 10:81

J. Rychik,A.M. Atz,D.S. Celermajer,B.J. Deal,M.A. Gatzoulis,M.H. Gewillig,T.-Y. Hsia,D.T. Hsu,A.H. Kovacs,B.W. McCrindle,J.W. Newburger,N.A. Pike,M. Rodefeld,D.N. Rosenthal,K.R. Schumacher,B.S. Marino,K. Stout,G. Veldtman,A.K. Younoszai,Y. D'Udekem. Evaluation and Management of the Child and Adult with Fontan Circulation: A Scientific Statement from the American Heart Association. Circulation. 2019///. 140:E234

J. Wang,E. Liu,Y. Wang,Y. Qiao,T. Zhang,B. Li,Z. Zhang,N. Li,G. Hu. Association of early pregnancy body mass index and children's birth weight with risk of being overweight in childhood. American journal of human biology : the official journal of the Human Biology Council. 2018///. 30:e23174

J. Zhang,H. Liu,X. Mao,Q. Chen,Y. Fan,Y. Xiao,Y. Wang,Y. Kuang. Effect of body mass index on pregnancy outcomes in a freeze-all policy: an analysis of 22,043 first autologous frozen-thawed embryo transfer cycles in China. BMC medicine. 2019/06/26/. 17:114, 2019

J.C. Hall,J.M. Watts,P.E. O'Brien,R.E. Dunstan,J.F. Walsh,A.H. Slavotinek,R.G. Elmslie. Gastric surgery for morbid obesity. The Adelaide Study. Annals of surgery. 1990///. 211:419

J.C. Paredes Palma,Byhen E. Lopez,L. Ibanez,Macedo L. Balladares,Palma C. Paredes,Velazquez C. Ramirez. Comparative treatment between sitagliptin vs. metformin, alone or in combination, in patients with polycystic ovary syndrome. A clinical entity at high risk for developing diabetes mellitus and gestational diabetes: a pilot study. Revista Medica del Hospital General de Mexico. 2018///. 81:15

J.F. Plows,J.M. Ramos Nieves,F. Budin,K. Mace,C.M. Reynolds,M.H. Vickers,I. Silva-Zolezzi,P.N. Baker,J.L. Stanley. The effects of myo-inositol and probiotic supplementation in a high-fat-fed preclinical model of glucose intolerance in pregnancy. British Journal of Nutrition. 2020///. 47:516

J.G. Alves,A.S.R. Souza,J.N. Figueiroa,C.A.L. de Araujo,A. Guimaraes,J.G. Ray. Visceral Adipose Tissue Depth in Early Pregnancy and Gestational Diabetes Mellitus - a Cohort Study. Sci Rep. 2020/02/06/. 10:2032, 2020

J.M. Falko. Familial chylomicronemia syndrome: A clinical guide for endocrinologists. Endocrine Practice. 2018///. 24:758

J.M. Walsh,C.A. McGowan,R. Mahony,M.E. Foley,F.M. McAuliffe. Low glycaemic index diet in pregnancy to prevent macrosomia (ROLO study): randomised control trial. BMJ (Clinical research ed. 2012///.). 345:e5605, 2012

J.P. Tran,S.S. Stribling,U.C. Ibezim,C. Omere,K.A. McEnery,L.D. PACHECO,G.D. Hankins,G.R. SAADE,A.F. Saad. Performance of Risk Assessment Models for Peripartum Thromboprophylaxis. Reproductive Sciences. 2019///. 26:1243

J.R. Bartholomew,N.S. Evans. Travel-related venous thromboembolism. Vascular Medicine (United Kingdom). 2019///. 24:93

Janetski J. Living on the edge of diabetes: How to integrate the diabetes prevention program into a community setting. DIABETES SPECTRUM 2009;22(3):179-82.

Jans G, Matthys C, Bogaerts A, Lannoo M, Verhaeghe J, Van der Schueren B, et al. Maternal micronutrient deficiencies and related adverse neonatal outcomes after bariatric surgery: a systematic review. Adv Nutr (Bethesda) 2015;6(4):420-9.

Jelsma JG, van Poppel MN Smith BJ Cinnadaio. Changing psychosocial determinants of physical activity and diet in women with a history of gestational diabetes mellitus. #journal#. 2017. (no pagination):#pages#

Jin J. Pregnancy after bariatric surgery associated with risks to offspring. J Am Med Assoc 2013;310(22):2388. [PMID: http://jama.jamanetwork.com/data/Journals/JAMA/929436/jmn130141.pdf]

Joham AE, Palomba S, Hart R. Polycystic Ovary Syndrome, Obesity, and Pregnancy. Semin Reprod Med 2016;34(2):93-101.

Johansson K, Cnattingius S, Naslund I, Roos N, Trolle Lagerros Y, Granath F, et al. Outcomes of pregnancy after bariatric surgery. N Engl J Med 2015;372(9):814-24.

Justine.Richardson Dol. Impact of mobile health (mHealth) interventions during the perinatal period for mothers in low- and middleincome countries: a systematic review. JBI Database of Systematic Reviews and Implementation Reports.17(8):1634-1667, 2019.. 2019///. 78:#pages#

K. Manerkar,J. Harding,C. Conlon,C. McKinlay. Maternal gestational diabetes and infant feeding, nutrition and growth: A systematic review and meta-analysis. British Journal of Nutrition. 2020///. 298:#pages#

K. Srivaratharajah,B.L. Abramson. Identifying and managing younger women at high risk of cardiovascular disease. CMAJ : Canadian Medical Association journal = journal de l'Association medicale canadienne. 2019///. 191:E159

K. Takagi,N. Iwama,H. Metoki,Y. Uchikura,Y. Matsubara,K. Matsubara,H. Nishigori,M. Saito,I. Fujiwara,K. Sakurai,S. Kuriyama,T. Arima,K. Nakai,N. Yaegashi,T. Sugiyama,Japan Environment and Children's Study Group. Paternal height has an impact on birth weight of their offspring in a Japanese population: the Japan Environment and Children's Study. Journal of developmental origins of health and disease. 2019/10//. 10:542

K. Wiles,L. Chappell,K. Clark,L. Elman,M. Hall,L. Lightstone,G. Mohamed,D. Mukherjee,C. Nelson-Piercy,P. Webster,R. Whybrow,K. Bramham. Clinical practice guideline on pregnancy and renal disease. BMC nephrology. 2019///. 20:#pages#

K.-H. Chen,L.-R. Chen. Provoking factors for postpartum chronic hypertension in women with preceding gestational hypertension/preeclampsia: A longitudinal cohort study of 22,798 pregnancies. International Journal of Medical Sciences. 2020///. 17:543

K.D. Wenstrom. Outcomes at 2 years of age after repeat doses of antenatal corticosteroids: commentary. Obstetrical and Gynecological Survey. 2008///. 63:18

K.K. Garnaes,S.A. Nyrnes,K.A. Salvesen,O. Salvesen,S. Morkved,T. Moholdt. Effect of supervised exercise training during pregnancy on neonatal and maternal outcomes among overweight and obese women. Secondary analyses of the ETIP trial: a randomised controlled trial. PloS one. 2017///. 12:e0173937, 2017

K.L. Gatford,D.J. Kennaway,H. Liu,D.O. Kleemann,T.R. Kuchel,T.J. Varcoe. Simulated shift work disrupts maternal circadian rhythms and metabolism, and increases gestation length in sheep. The Journal of physiology. 2019/04//. 597:1889

K.M. Ross,C. Guardino,Schetter C. Dunkel,C.J. Hobel. Interactions between race/ethnicity, poverty status, and pregnancy cardio-metabolic diseases in prediction of postpartum cardio-metabolic health. Ethnicity and health. 2018///. 22:1

Kahr MK, Suter MA, Ballas J, Ramin SM, Monga M, Lee W, et al. Geospatial analysis of food environment demonstrates associations with gestational diabetes. Am J Obstet Gynecol 2016;214(1):110-9.

Kahyaoglu I, Kinay T, Kayikcioglu F, Kahyaoglu S, Mollamahmutoglu L. Percentage change in body mass index or gestational weight gain: Which is a better predictor of foetal macrosomia? J Obstet Gynaecol 2015;35(8):817-20.

Kansu-Celik H, Kisa Karakaya B, Guzel AI, Tasci Y, Erkaya S. To evaluate the effect of pre-pregnancy body mass index on maternal and perinatal outcomes among adolescent pregnant women. J Matern Fetal Neonatal Med 2016;1-13.

Kasper S. Madsen,Pernille Kahler,Katrine Lise Kahler,Sten Madsbad,Filip Gnesin,Maria Inti Metzendorf,Bernd Richter,Bianca Hemmingsen. Metformin and second- or third-generation sulphonylurea combination therapy for adults with type 2 diabetes mellitus. Cochrane Database of Systematic Reviews 2019;(4). 2019/04/15/. 20:#pages#

Kawakita T, Reddy UM, Landy HJ, Iqbal SN, Huang C-C, Grantz KL. Indications for primary cesarean delivery relative to body mass index. Am J Obstet Gynecol 2016;215(4):515. [PMID: http://www.elsevier.com/inca/publications/store/6/2/3/2/7/7/index.htt]

Kim SY, England L, Wilson HG, Bish C, Satten GA, Dietz P. Percentage of gestational diabetes mellitus attributable to overweight and obesity. Am J Public Health 2010;100(6):1047-52.

Kim YH, Lee HJ, Shin JE, Lee Y, Shin JC, Park TC, et al. The predictive value of the uterine artery pulsatility index during the early third trimester for the occurrence of adverse pregnancy outcomes depending on the maternal obesity. Obes Res Clin Pract 2015;9(4):374-81.

Kjaer MM, Nilas L. Pregnancy after bariatric surgery--a review of benefits and risks. Acta Obstet Gynecol Scand 2013;92(3):264-71.

Knight BA, Shields BM, Hattersley AT, Vaidya B. Maternal hypothyroxinaemia in pregnancy is associated with obesity and adverse maternal metabolic parameters. EUR J ENDOCRINOL 2016;174(1):51-7.

Kolstad E, Veiby G, Gilhus NE, Bjork M. Overweight in epilepsy as a risk factor for pregnancy and delivery complications. Epilepsia 2016;57(11):1849-57.

Kongubol A, Phupong V. Prepregnancy obesity and the risk of gestational diabetes mellitus. BMC Pregnancy Childbirth 2011;11:59

Kosus N, Kosus A, Turhan N. Relation between abdominal subcutaneous fat tissue thickness and inflammatory markers during pregnancy. Arch Med Sci 2014;10(4):739-45.

Kothari D, Lim BH. Diabetes and pregnancy: time to rethink the focus on type 2 diabetes. Aust N Z J Obstet Gynaecol 2014;54(2):181-3.

Krishna M, Kalyanaraman K, Veena SR, Krishanveni GV, Karat SC, Cox V, et al. Cohort Profile: The 1934-66 Mysore Birth Records Cohort in South India. Int J Epidemiol 2015;44(6):1833-41. [PMID: http://ije.oxfordjournals.org/]

Kumar A, Chaudhary K, Prasad S. Maternal indicators and obstetric outcome in the north Indian population: a hospital-based study. J Postgrad Med 2010;56(3):192-5.

L. Freebairn,J.-A. Atkinson,P.M. Kelly,G. McDonnell,L. Rychetnik. Decision makers' experience of participatory dynamic simulation modelling: methods for public health policy. BMC medical informatics and decision making. 2018///. 18:131

L. Ibanez,Zegher F. De. Exploring the use of metformin in pregnant women with polycystic ovary syndrome: new evidence, new wisdom. The Lancet Diabetes and Endocrinology. 2019///. 7:242

L. Mary,K. Chennen,C. Stoetzel,M. Antin,A. Leuvrey,E. Nourisson,E. Alanio-Detton,M.C. Antal,T. Attie-Bitach,P. Bouvagnet,R. Bouvier,A. Buenerd,A. Clemenson,L. Devisme,B. Gasser,B. Gilbert-Dussardier,F. Guimiot,Kien P. Khau Van,B. Leroy,P. Loget,J. Martinovic,F. Pelluard,M.J. Perez,F. Petit,L. Pinson,C. Rooryck-Thambo,O. Poch,H. Dollfus,E. Schaefer,J. Muller. Bardet-Biedl syndrome: Antenatal presentation of forty-five fetuses with biallelic pathogenic variants in known Bardet-Biedl syndrome genes. Clinical Genetics. 2019/03//. 95:384

L. Nesti,A. Mengozzi,D. Trico. Impact of nutrient type and sequence on glucose tolerance: Physiological insights and therapeutic implications. Frontiers in endocrinology. 2019/03//. 10:#pages#

L. Tolentino,M. Yigeremu,S. Teklu,S. Attia,M. Weiler,N. Frank,J.B. Dixon,R.L. Gleason. Three-dimensional camera anthropometry to assess risk of cephalopelvic disproportion-related obstructed labour in Ethiopia. Interface focus. 2019/10/06/. 9:20190036, 2019

L.A.H. Haakstad,N. Voldner,K. Bo. Pregnancy and advanced maternal age-The associations between regular exercise and maternal and newborn health variables. Acta obstetricia et gynecologica Scandinavica. 2020/02//. 99:240

L.M. Procopciuc,G. Nemeti,E. Buzdugan,M. Iancu,F. Stamatian,G. Caracostea. Renin-angiotensin system gene variants and risk of early- and late-onset preeclampsia: A single center case-control study. Pregnancy hypertension. 2019///. 18:1

L.V. Gabis,S. Shefer,A. Raas-Rothschild. Ethical Dilemmas Linked to Fragile X Testing of Minors-a Preliminary Survey Among Professionals. Journal of Molecular Neuroscience. 2020///. 70:254

La MA, Broekmans FJ, Volpe A, Fauser BC, MacKlon NS. Anti-Mullerian hormone (AMH): What do we still need to know? Hum Reprod 2009;24(9):2264-75.

Laafira A, White SW, Griffin CJ, Graham D. Impact of the new IADPSG gestational diabetes diagnostic criteria on pregnancy outcomes in Western Australia. Aust N Z J Obstet Gynaecol 2016;56(1):36-41.

Lam DCL, Lui MMS, Lam JCM, Ong LHY, Lam KSL, Ip MSM. Prevalence and recognition of obstructive sleep apnea in Chinese women with type 2 diabetes mellitus. Chest 2010;138(5):1101-7. [PMID: http://chestjournal.chestpubs.org/content/138/5/1101.full.pdf+html]

Lee Hooper,Lena Al-Khudairy,Asmaa S. Abdelhamid,Karen Rees,Julii S. Brainard,Tracey J. Brown,Sarah M. Ajabnoor,Alex T. O'Brien,Lauren E. Winstanley,Daisy H. Donaldson,Fujian Song,HO Katherine Deane. Omega-6 fats for the primary and secondary prevention of cardiovascular disease. Cochrane Database of Systematic Reviews 2018;(11). 2018/11/28/. 54:#pages#

Legro RS. Obesity and PCOS: Implications for diagnosis and treatment. Semin Reprod Med 2012;30(6):496-506.

Lei Q, Niu J, Lv L, Duan D, Wen J, Lin X, et al. Clustering of metabolic risk factors and adverse pregnancy outcomes: a prospective cohort study. Diabetes Metab Res Rev 2016;32(8):835-42.

Leng J, Li W, Zhang S, Liu H, Wang L, Liu G, et al. GDM Women's Pre-Pregnancy Overweight/Obesity and Gestational Weight Gain on Offspring Overweight Status. PloS one 2015;10(6):e0129536

Leng J, Wang P, Shao P, Zhang C, Li W, Li N, et al. Passive smoking increased risk of gestational diabetes mellitus independently and synergistically with prepregnancy obesity in Tianjin, China. Diabetes Metab Res Rev 2016;

Leventakou V, Roumeliotaki T, Martinez D, Barros H, Brantsaeter AL, Casas M, et al. Fish intake during pregnancy, fetal growth, and gestational length in 19 European birth cohort studies. Am J Clin Nutr 2014;99(3):506-16.

Li G, Kong L, Zhang L, Fan L, Su Y, Rose JC, et al. Early Pregnancy Maternal Lipid Profiles and the Risk of Gestational Diabetes Mellitus Stratified for Body Mass Index. Reprod Sci 2015;22(6):712-7.

Li N, Liu E, Guo J, Pan L, Li B, Wang P, et al. Maternal prepregnancy body mass index and gestational weight gain on pregnancy outcomes. PloS one 2013;8(12):e82310

Li W, Liu H, Qiao Y, Lv F, Zhang S, Wang L, et al. Metabolic syndrome of weight change from pre-pregnancy to 1-5 years post-partum among Chinese women with prior gestational diabetes. Diabet Med 2015;32(11):1492-9.

Li X, Tan H, Huang X, Zhou S, Hu S, Wang X, et al. Similarities and differences between the risk factors for gestational hypertension and preeclampsia: A population based cohort study in south China. Pregnancy Hypertens 2016;6(1):66-71.

Liang S, Liu X, Fan P, Liu R, Zhang J, He G, et al. Association Between Val158Met Functional Polymorphism in the COMT Gene and Risk of Preeclampsia in a Chinese Population. Arch Med Res 2012;43(2):154-8.

Liu L, Hong Z, Zhang L. Associations of prepregnancy body mass index and gestational weight gain with pregnancy outcomes in nulliparous women delivering single live babies. Sci rep 2015;5:12863

Liu X, Du J, Wang G, Chen Z, Wang W, Xi Q. Effect of pre-pregnancy body mass index on adverse pregnancy outcome in north of China. Arch Gynecol Obstet 2011;283(1):65-70.

Loire P, Vicaut E, Cruaud P, Charnaux N, Carbillon L. Limited value of angiogenic factors in obese women. Pregnancy Hypertens 2012;2(4):368-70.

Loretta.Kynoch Anderson. Effectiveness of breast massage for the treatment of women with breastfeeding problems: a systematic review. JBI Database of Systematic Reviews and Implementation Reports.17(8):1668-1694, 2019.. 2019///. 45:#pages#

Lussana F, Coppens M, Cattaneo M, Middeldorp S. Pregnancy-related venous thromboembolism: Risk and the effect of thromboprophylaxis. Thromb Res 2012;129(6):673-80.

Lutsiv O, McKinney B, Foster G, Taylor VH, Pullenayegum E, McDonald SD. Pregnancy complications associated with the co-prevalence of excess maternal weight and depression. Int J Obes (Lond) 2015;39(12):1710-6.

M. Cai,S.L. Loy,K.H. Tan,K.M. Godfrey,P.D. Gluckman,Y.S. Chong,L.P. Shek,Y.B. Cheung,N. Lek,Y.S. Lee,S.Y. Chan,J.K.Y. Chan,F. Yap,S.B. Ang. Association of Elective and Emergency Cesarean Delivery With Early Childhood Overweight at 12 Months of Age. JAMA network open. 2018/11/02/. 1:e185025

M. Calan,T. Arkan,T. Kume,F. Bayraktar. The relationship between urotensin II and insulin resistance in women with gestational diabetes mellitus. Hormones (Athens, Greece). 2019///. 18:91

M. Calatayud,O. Koren,M.C. Collado. Maternal Microbiome and Metabolic Health Program Microbiome Development and Health of the Offspring. Trends in Endocrinology and Metabolism. 2019/10//. 30:735

M. Franzago,F. Fraticelli,Nicola M. Di,F. Bianco,D. Marchetti,C. Celentano,M. Liberati,Caterina R. De,L. Stuppia,E. Vitacolonna. Early Subclinical Atherosclerosis in Gestational Diabetes: The Predictive Role of Routine Biomarkers and Nutrigenetic Variants. Journal of diabetes research. 2018///. 2018:9242579, 2018.:#pages#

M. Guha,H. Banerjee,P. Mitra,M. Das. The Demographic Diversity of Food Intake and Prevalence of Kidney Stone Diseases in the Indian Continent. Foods (Basel, Switzerland). 2019/01/21/. 8:#pages#

M. Hashim,H. Radwan,H. Hasan,R.S. Obaid,Ghazal H. Al,Hilali M. Al,R. Rayess,N. Chehayber,H.J.J. Mohamed,F. Naja. Gestational weight gain and gestational diabetes among Emirati and Arab women in the United Arab Emirates: results from the MISC cohort. BMC pregnancy and childbirth. 2019/12/03/. 19:463, 2019

M. Katzow,M.J. Messito,A.L. Mendelsohn,M.A. Scott,R.S. Gross. The Protective Effect of Prenatal Social Support on Infant Adiposity in the First 18 Months of Life. The Journal of pediatrics. 2019/06//. 209:77

M. Vargas-Terrones,T.S. Nagpal,R. Barakat. Impact of exercise during pregnancy on gestational weight gain and birth weight: an overview. Brazilian journal of physical therapy. 2019///. 23:164

M.A. Abdulmalik,J.J. Ayoub,A. Mahmoud,M.I.N.A. collaborators,L. Nasreddine,F. Naja. Pre-pregnancy BMI, gestational weight gain and birth outcomes in Lebanon and Qatar: Results of the MINA cohort. PloS one. 2019///. 14:e0219248, 2019

M.B. Alves,D.P. Laureano,Molle R. Dalle,T.D. Machado,A.P.A. Salvador,P.M. Miguel,D. Lupinsky,C. Dalmaz,P.P. Silveira. Intrauterine growth restriction increases impulsive behavior and is associated with altered dopamine transmission in both medial prefrontal and orbitofrontal cortex in female rats. Physiology and Behavior. 2019/05/15/. 204:336-346, 2019 05 15.:#pages#

M.C. Srivastava,R. Srivastava,P.K. Verma,A. Gautam. Metabolic syndrome and periodontal disease: An overview for physicians. Journal of family medicine and primary care. 2019/11//. 8:3492

M.D. Rybstein,M.T. DeSancho. Risk factors for and clinical management of venous thromboembolism during pregnancy. Clinical Advances in Hematology and Oncology. 2019///. 17:396

M.H. Lauridsen,N. Uldbjerg,O.B. Petersen,E.M. Vestergaard,N.B. Matthiesen,T.B. Henriksen,J.R. Ostergaard,V.E. Hjortdal. Fetal Heart Defects and Measures of Cerebral Size. The Journal of pediatrics. 2019/07//. 210:146

M.J. Rosa,F. Nentin,Enlow M. Bosquet,M.R. Hacker,N. Pollas,B. Coull,R.J. Wright. Sex-specific associations between prenatal negative life events and birth outcomes. Stress. 2019/11//. 22:647

M.K. Ali,K.M. Bullard,G. Imperatore,S.R. Benoit,D.B. Rolka,A.L. Albright,E.W. Gregg. Reach and use of diabetes prevention services in the United States, 2016-2017. JAMA network open. 2019///. 2:#pages#

M.M. Adeva-Andany,M. Gonzalez-Lucan,C. Fernandez-Fernandez,N. Carneiro-Freire,M. Seco-Filgueira,A.M. Pedre-Pineiro. Effect of diet composition on insulin sensitivity in humans. Clin nutr espen. 2019/10//. 33:29

M.P. Lubis,H. Hariman,S.N. Lumbanraja,A. Bachtiar. The role of placental growth factor, soluble endoglin, and uterine artery diastolic notch to predict the early onset of preeclampsia. Open access Macedonian journal of medical sciences. 2019///. 7:1153

M.R. Araneta. Engaging the ASEAN diaspora: Type 2 diabetes prevalence, pathophysiology, and unique risk factors among filipino migrants in the United States. Journal of the ASEAN Federation of Endocrine Societies. 2019///. 34:126

M.R. Battin,V. Obolonkin,E. Rush,W. Hague,S. Coat,J. Rowan. Blood pressure measurement at two years in offspring of women randomized to a trial of metformin for GDM: follow up data from the MiG trial. BMC pediatrics. 2015///. 15:54, 2015

M.S. Christianson,R.S. Legro,S. Jin,E. Eisenberg,M.P. Diamond,K.R. Hansen,W. Vitek,A.K. Styer,P. Casson,C. Coutifaris,G.M. Christman,R. Alvero,E.E. Puscheck,A.Y. Christy,F. Sun,H. Zhang,A.J. Polotsky,N. Santoro. Comparison of sonohysterography to hysterosalpingogram for tubal patency assessment in a multicenter fertility treatment trial among women with polycystic ovary syndrome. Journal of assisted reproduction and genetics. 2018///. 35:2173

Madsen NL, Schwartz SM, Lewin MB, Mueller BA. Prepregnancy body mass index and congenital heart defects among offspring: a population-based study. Congenit heart dis 2013;8(2):131-41.

Malgorzata Klimek,Magdalena Nitecka,Grazyna Dutkowska,Maja Gilarska,Przemko Kwinta. Temperament traits in 4-year-old children born prematurely-May they suggest a threat for mental functioning?. Psychiatria Polska. 2018///. 52:371

Man J, Hutchinson JC, Ashworth M, Heazell AE, Jeffrey I, Sebire NJ. Stillbirth and intrauterine fetal death: contemporary demographic features of >1000 cases from an urban population. Ultrasound Obstet Gynecol 2016;

Mancia G, Fagard R, Narkiewicz K, Redon J, Zanchetti A, Bohm M, et al. The task force for the management ofarterial hypertension of the european society ofhypertension (esh) and of the european society of cardiology (esc). J Hypertens 2013;31(7):1281-357.

Marcello Di Nisio,Iris M. Wichers,Saskia Middeldorp. Treatment for superficial thrombophlebitis of the leg. Cochrane Database of Systematic Reviews 2018;(2). 2018/02/20/. 217:#pages#

Maria Makrides,Danielle D. Crosby,Emily Shepherd,Caroline A. Crowther. Magnesium supplementation in pregnancy. Cochrane Database of Systematic Reviews 2019;(5). 2019/05/09/. 28:#pages#

Martis R, Brown J, Alsweiler J, Crawford TJ, Crowther CA. Different intensities of glycaemic control for women with gestational diabetes mellitus. Cochrane Database Syst Rev 2016;4:CD011624

Maternal body mass index and cervical length among women with a history of spontaneous preterm birth. Journal of Maternal-Fetal and Neonatal Medicine. 2018///. 2018.:#pages#

Maya Tickell-Painter,Nicola Maayan,Rachel Saunders,Cheryl Pace,David Sinclair. Mefloquine for preventing malaria during travel to endemic areas. Cochrane Database of Systematic Reviews 2017;(10). 2017/10/24/. 48:#pages#

McClelland SH, Bogod DG, Hardman JG. Pre-oxygenation and apnoea in pregnancy: changes during labour and with obstetric morbidity in a computational simulation. Anaesthesia 2009;64(4):371-7.

McDonald SD, McKinney B, Foster G, Taylor V, Lutsiv O, Pullenayegum E. The combined effects of maternal depression and excess weight on neonatal outcomes. Int J Obes (Lond) 2015;39(7):1033-40.

Mei J, Liao S, Liu Y, Tan Y, Wang H, Liang Y, et al. Association of variants in CDKN2A/2B and CDKAL1 genes with gestational insulin sensitivity and disposition in pregnant Han Chinese women. J diabetes investig 2015;6(3):295-301.

Mendieta Zeron H, Garcia Solorio VJ, Nava Diaz PM, Garduno Alanis A, Santillan Benitez JG, Dominguez Garcia V, et al. Hyperleptinemia as a prognostic factor for preeclampsia: a cohort study. Acta Medica (Hradec Kralove) 2012;55(4):165-71.

Millene DC, Dalva De Barros CM, Marisa PS. Pregnancy after bariatric surgery: Implications for mother and newborn. Obes Surg 2011;21(6):699-706.

Mimouni-Zerguini S, Smail M, Boudiba A, Derguini M. Gestational diabetes: Risk factors, development, and perinatal outcomes: A survey at the University hospital Mustapha Bacha, Algiers (Algeria). Med Mal Metab 2009;3(6):626-33.

More N. Shah,S. Das,U. Bapat,M. Rajguru,G. Alcock,W. Joshi,S. Pantvaidya,D. Osrin. Community resource centres to improve the health of women and children in Mumbai slums: study protocol for a cluster randomized controlled trial. Trials. 2013///. 14:132, 2013

Morikawa M, Yamada T, Yamada T, Sato S, Cho K, Minakami H. Prevalence of hyperglycemia during pregnancy according to maternal age and pre-pregnancy body mass index in Japan, 2007-2009. Int J Gynaecol Obstet 2012;118(3):198-201.

Mottola MF, Artal R. Role of Exercise in Reducing Gestational Diabetes Mellitus. Clin Obstet Gynecol 2016;59(3):620-8.

Mumford SL, Garbose RA Kim. Association of preconception serum 25-hydroxyvitamin D concentrations with livebirth and pregnancy loss: a prospective cohort study. #journal#. 2018. (no pagination):#pages#

Mutsaerts MAQ, Groen H, Buiter-Van der Meer A, Sijtsma A, Sauer PJJ, Land JA, et al. Effects of paternal and maternal lifestyle factors on pregnancy complications and perinatal outcome. A population-based birth-cohort study: the GECKO Drenthe cohort. Hum Reprod 2014;29(4):824-34.

Mwanri AW, Kinabo J, Ramaiya K, Feskens EJM. Gestational diabetes mellitus in sub-Saharan Africa: systematic review and metaregression on prevalence and risk factors. Trop Med Int Health 2015;20(8):983-1002.

N. Kaseva,M. Vaarasmaki,J. Sundvall,H.M. Matinolli,M. Sipola,M. Tikanmaki,K. Heinonen,A. Lano,K. Wehkalampi,D. Wolke,A. Ruokonen,S. Andersson,M.R. Jarvelin,K. Raikkonen,J.G. Eriksson,E. Kajantie. Gestational Diabetes But Not Prepregnancy Overweight Predicts for Cardiometabolic Markers in Offspring Twenty Years Later. The Journal of clinical endocrinology and metabolism. 2019/07/01/. 104:2785

N. Sukumar,H. Dallosso,P. Saravanan,T. Yates,C. Telling,K. Shorthose,A. Northern,S. Schreder,C. Brough,L.J. Gray,M.J. Davies,K. Khunti. Baby Steps - A structured group education programme with accompanying mobile web application designed to promote physical activity in women with a history of gestational diabetes: Study protocol for a randomised controlled trial. Trials. 2018///. 19:#pages#

N.K. Wenger. Female-friendly focus: 2019 ACC/AHA Guideline on the Primary Prevention of Cardiovascular Disease. Clinical Cardiology. 2019///. 42:706

N.L. Segal. Co-Twin Control Studies: Natural Events, Experimental Interventions and Rare Happenings/Twin Research: Cancer Risk in Overweight Twins; Prognosis after Fetal Loss of One Twin; Twin Concordance for Parkinson's Disease; Neuroanatomy of Musically Discordant MZ Twins/News Articles: Twin Birth with Two Wombs; Twins' Prenatal Interactions; Switched-at-Birth Twins; Fetus-in-Fetu; Unsolved Paternity. Twin Research and Human Genetics. 2019///. 22:272

N.V. Kizirian,K.I. Black,L. Musgrave,C. Hespe,A. Gordon. Understanding and provision of preconception care by general practitioners. The Australian & New Zealand journal of obstetrics & gynaecology. 2019/12//. 59:799

Nagalla SR, Snyder CK, Michaels JE, Laughlin MJ, Roberts CT, Balaji M, et al. Maternal serum biomarkers for risk assessment in gestational diabetes. A potential universal screening test to predict GDM status. Indian J Endocrinol Metab 2015;19(1):155-9.

Najafian M, Cheraghi M. Occurrence of fetal macrosomia rate and its maternal and neonatal complications: a 5-year cohort study. ISRN Obstet Gynecol 2012;2012:353791

Navi Khan GA, Ishrat N, Afshan SN. Blood pressure pattern in pregnant women of different body mass index in three trimesters of pregnancy. Indian J Public Health Res Dev 2013;4(3):98-102. [PMID: http://www.indianjournals.com/ijor.aspx?target=ijor:ijphrd&volume=4&issue=3&article=022&type=pdf]

Nazari M, Sharifah Zainiyah SY, Lye MS, Zalilah MS, Heidarzadeh M. Comparison of maternal characteristics in low birth weight and normal birth weight infants. East Mediterr Health J 2013;19(9):775-81. [PMID: http://applications.emro.who.int/emhj/v19/09/EMHJ_2013_19_9_775_781.pdf]

Nerenberg KA, Johnson JA, Leung B, Savu A, Ryan EA, Chik CL, et al. Risks of gestational diabetes and preeclampsia over the last decade in a cohort of Alberta women. J Obstet Gynaecol Can 2013;35(11):986-94.

Nicklas JM, Miller LJ Zera CA Davis RB Levkoff SE Seely EW. Factors associated with depressive symptoms in the early postpartum period among women with recent gestational diabetes mellitus. #journal#. 2013. 17:#pages#

Nilofer AR, Raju VS, Dakshayini BR, Zaki SA. Screening in high-risk group of gestational diabetes mellitus with its maternal and fetal outcomes. Indian J Endocrinol Metab 2012;16 Suppl 1:S74-S78

Oken E. Excess gestational weight gain amplifies risks among obese mothers. Epidemiology 2009;20(1):82-3.

P. Ioannidou,D. Papanikolaou,T. Mikos,G. Mastorakos,D.G. Goulis. Predictive factors of Hyperemesis Gravidarum: A systematic review. European journal of obstetrics, gynecology, and reproductive biology. 2019/07//. 238:178

P. Javadian,S. Alimohamadi,M.H. Gharedaghi,S. Hantoushzadeh. Gestational diabetes mellitus and iron supplement; effects on pregnancy outcome. Acta Medica Iranica. 2014///. 52:385

P. Mazumder,S. Dutta,J. Kaur,A. Narang. Single versus multiple courses of antenatal betamethasone and neonatal outcome: a randomized controlled trial. Indian pediatrics. 2008///. 45:661

P.L. Hebert,J.G. Katon. Repurposing of administrative data for research: Still useful but for how much longer?. Pediatrics. 2019///. 143:#pages#

P.P. Devarshi,R.W. Grant,C.J. Ikonte,S.H. Mitmesser. Maternal omega-3 nutrition, placental transfer and fetal brain development in gestational diabetes and preeclampsia. Nutrients. 2019///. 11:#pages#

P.S. Magnani,H. Bettiol,A.A.M. da Silva,M.A. Barbieri,Cavalli R. de Carvalho,L.G.O. Brito. Urinary incontinence between 12 and 24 months postpartum: a cross-sectional study nested in a Brazilian cohort from two cities with different socioeconomic characteristics. International urogynecology journal. 2019/06//. 30:1003

Park S, Kim M-Y, Baik SH, Woo J-T, Kwon YJ, Daily JW, et al. Gestational diabetes is associated with high energy and saturated fat intakes and with low plasma visfatin and adiponectin levels independent of prepregnancy BMI. Eur J Clin Nutr 2013;67(2):196-201.

Parveen, M., Ismaile, M., and Masood, R.. Obesity as a predictor of adverse maternal outcomes among pregnant women of a rural community. #journal#. 2017. 11:#pages#

Persson M, Johansson S, Cnattingius S. Inter-pregnancy Weight Change and Risks of Severe Birth-Asphyxia-Related Outcomes in Singleton Infants Born at Term: A Nationwide Swedish Cohort Study. PLoS Med 2016;13(6):e1002033

Phillippe HM, Sparkman AY. Venous thrombosis: Preventing clots in women at risk. J Fam Pract 2010;59(6):315-21.

Q. Chen,Y. Feng,H. Yang,W. Wu,P. Zhang,K. Wang,Y. Wang,J. Ko,J. Shen,L. Guo,F. Zhao,W. Du,S. Ru,S. Wang,Y. Zhang. A Vitamin Pattern Diet Is Associated with Decreased Risk of Gestational Diabetes Mellitus in Chinese Women: Results from a Case Control Study in Taiyuan, China. Journal of diabetes research. 2019///. 2019:5232308, 2019.:#pages#

Q. Fu. Sex differences in sympathetic activity in obesity and its related hypertension. Annals of the New York Academy of Sciences. 2019/10//. 1454:31

Qiu C, Frederick IO, Sorensen TK, Enquobahrie DA, Williams MA. Sleep duration and plasma leptin concentrations in early pregnancy among lean and overweight/obese women: a cross sectional study. BMC Res Notes 2014;7:20

R. Khursheed,S.K. Singh,S. Wadhwa,B. Kapoor,M. Gulati,R. Kumar,A.K. Ramanunny,A. Awasthi,K. Dua. Treatment strategies against diabetes: Success so far and challenges ahead. European Journal of Pharmacology. 2019///. 862:172625

R. Obeid,W. Holzgreve,K. Pietrzik. Folate supplementation for prevention of congenital heart defects and low birth weight: an update. #journal#. 2019/10//. 9:S424

R. Patel,A. Gupta,S. Chauhan,D.W. Bansod. Effects of sanitation practices on adverse pregnancy outcomes in India: a conducive finding from recent Indian demographic health survey. BMC pregnancy and childbirth. 2019/10/24/. 19:378, 2019

R. Rais,R. Starikov,W. Robert,P. Has,M. He. Clinicopathological correlation of large-for-gestational age placenta in pregnancies with pregestational diabetes. Pathology, research and practice. 2019/03//. 215:405

R. Townsend,A. Khalil,Y. Premakumar,J. Allotey,K.I.E. Snell,C. Chan,L.C. Chappell,R. Hooper,M. Green,B.W. Mol,B. Thilaganathan,S. Thangaratinam. Prediction of pre-eclampsia: review of reviews. Ultrasound in obstetrics and gynecology : the official journal of the International Society of Ultrasound in Obstetrics and Gynecology. 2019///. 54:16

R.F. Zhao,W.Y. Zhang,L. Zhou,Y. Chen. Building a predictive model for successful vaginal delivery in nulliparas with term cephalic singleton pregnancies using decision tree analysis. The journal of obstetrics and gynaecology research. 2019/08//. 45:1536

R.J. Rodgers,J.C. Avery,V.M. Moore,M.J. Davies,R. Azziz,E. Stener-Victorin,L.J. Moran,S.A. Robertson,N.K. Stepto,R.J. Norman,H.J. Teede. Complex diseases and co-morbidities: Polycystic ovary syndrome and type 2 diabetes mellitus. Endocrine connections. 2019///. 8:R71

R.M. Post,T.A. Ketter,T. Uhde,J.C. Ballenger. Thirty years of clinical experience with carbamazepine in the treatment of bipolar illness: principles and practice. CNS Drugs. 2007///. 21:47

R.M. Rossi,C.R. Warshak,H.R. Masters,J.K. Regan,S.A. Kritzer,K.P. Magner. Comparison of prostaglandin and mechanical cervical ripening in the setting of small for gestational age neonates*. Journal of Maternal-Fetal and Neonatal Medicine. 2019///. 32:3841

R.S. Martins,T. Ahmed,S. Farhat,S.S. Fatima,S. Shahid. Epidermal growth factor receptor rs17337023 polymorphism in hypertensive gestational diabetic women: A pilot study. World journal of diabetes. 2019///. 10:396

Ramakrishnan U, Grant F, Goldenberg T, Zongrone A, Martorell R. Effect of women's nutrition before and during early pregnancy on maternal and infant outcomes: A systematic review. Paediatr Perinat Epidemiol 2012;26(SUPPL. 1):285-301.

Ranganath D. Rattehalli,Sai Zhao,Guo Bao Li,Mahesh B. Jayaram,Jun Xia,Stephanie Sampson. Risperidone versus placebo for schizophrenia. Cochrane Database of Systematic Reviews 2016;(12). 2016/12/15/. 26:#pages#

Ravnsborg T, Andersen LLT, Trabjerg ND, Rasmussen LM, Jensen DM, Overgaard M. First-trimester multimarker prediction of gestational diabetes mellitus using targeted mass spectrometry. Diabetologia 2016;59(5):970-9. [PMID: http://link.springer.de/link/service/journals/00125/index.htm]

Rees DA, Jenkins-Jones S, Morgan CL. Contemporary Reproductive Outcomes for Women With Polycystic Ovary Syndrome: A Retrospective Observational Study. J Clin Endocrinol Metab 2016;101(4):1664-72.

Rehder PM, Pereira BG, Silva E. The prognostic value of a normal oral glucose tolerance test in pregnant women who tested positive at screening: a validation study. Diabetol Metab Syndr 2012;4(1):10

Rocha, R. S., Gurgel Alves, J. A., Bezerra Maia E Holanda Moura, Araujo, E., Peixoto, A. B., Santana, E. F. M., Martins, W. P., Vasconcelos, C. T. M., Costa, F. D. S., and Oria, M. O. B.. Simple approach based on maternal characteristics and mean arterial pressure for the prediction of preeclampsia in the first trimester of pregnancy. #journal#. 2017. 45:#pages#

Romano ME, Enquobahrie DA, Simpson CD, Checkoway H, Williams MA. A Case-Cohort Study of Cadmium Body Burden and Gestational Diabetes Mellitus in American Women. Environ Health Perspect 2015;123(10):993-8.

Ronald L. Koretz,Maria Pleguezuelo,Vasiliki Arvaniti,Pilar Barrera Baena,Ruben Ciria,Selvan Kurinchi Gurusamy,Brian R. Davidson,Andrew K. Burroughs. Interferon for interferon nonresponding and relapsing patients with chronic hepatitis C. Cochrane Database of Systematic Reviews 2013;(1). 2012/12/14/. 72:#pages#

Rowan JA, Budden A, Sadler LC. Women with a nondiagnostic 75 g glucose tolerance test but elevated HbA1c in pregnancy: an additional group of women with gestational diabetes. Aust N Z J Obstet Gynaecol 2014;54(2):177-80.

Ruifrok AE, Althuizen E, Oostdam N, van Mechelen W, Mol BW, de Groot CJM, et al. The relationship of objectively measured physical activity and sedentary behaviour with gestational weight gain and birth weight. J Pregnancy 2014;2014:567379

S. Adam,S. Dias,P. Rheeder,C. Pheiffer. Does South Africa need a diabetes-in-pregnancy study group?. South African Medical Journal. 2019///. 109:455

S. Adam,S. Dias,P. Rheeder,C. Pheiffer. Does South Africa need a diabetes-in-pregnancy study group?. South African Medical Journal. 2019///. 109:455

S. Atashkhoei,R. Abri,B. Naghipour,Marandi P. Hatami,M.T. Fazeli Danesh. Effect of glucose containing crystalloid infusion on maternal hemodynamic status after spinal anesthesia for cesarean section. #journal#. 2018///. 8:2018

S. Behboudi-Gandevani,F.R. Tehrani,M. Rahmati,M. Amiri,F. Azizi. Trend of various adiposity indices in women with and without history of gestational diabetes: A population-based cohort study. BMC endocrine disorders. 2019///. 19:#pages#

S. Dadabhai,L. Gadama,R. Chamanga,R. Kawalazira,C. Katumbi,B. Makanani,D. Dula,N. Hua,B. Lau,M. Mallewa,T.E. Taha. Pregnancy Outcomes in the Era of Universal Antiretroviral Treatment in Sub-Saharan Africa (POISE Study). Journal of Acquired Immune Deficiency Syndromes. 2019///. 80:7

S. Desmedt,V. Desmedt,Vos L. De,J.R. Delanghe,R. Speeckaert,M.M. Speeckaert. Growth differentiation factor 15: A novel biomarker with high clinical potential. Critical reviews in clinical laboratory sciences. 2019///. 56:333

S. Dhawan,R. Natarajan. Epigenetics and Type 2 Diabetes Risk. Current diabetes reports. 2019///. 19:#pages#

S. Frantz,J. Parinaud,M. Kret,G. Rocher-Escriva,A. Papaxanthos-Roche,H. Creux,L. Chansel-Debordeaux,A. Benard,C. Hocke. Decrease in pregnancy rate after endometrial scratch in women undergoing a first or second in vitro fertilization. A multicenter randomized controlled trial. Human Reproduction. 2019///. 34:92

S. Hoirisch-Clapauch,A.E. Nardi. Autism spectrum disorders: let's talk about glucose?. Transl Psychiatry. 2019/01/31/. 9:51, 2019

S. Hot,S. Egin,B. Gokcek,M. Yesiltas,D.O. Karakas. Acute biliary pancreatitis during pregnancy and in the post-delivery period. Ulusal Travma ve Acil Cerrahi Dergisi. 2019///. 25:253

S. Mills,J.A. Lane,G.J. Smith,K.A. Grimaldi,R.P. Ross,C. Stanton. Precision nutrition and the microbiome part ii: Potential opportunities and pathways to commercialisation. Nutrients. 2019///. 11:#pages#

S. Sharma,S. Yadav,K. Chandiok,R.S. Sharma,V. Mishra,K.N. Saraswathy. Protein signatures linking history of miscarriages and metabolic syndrome: A proteomic study among North Indian women. Peerj. 2019///. 2019:#pages#

S. Singh,S. Picardo,C.H. Seow. Management of Inflammatory Bowel Diseases in Special Populations: Obese, Old, or Obstetric. Clinical Gastroenterology and Hepatology. 2019/11/08/. 2019 Nov 08:#pages#

S. Vannuccini,C. Ferrata,F. Perelli,S. Pinzauti,F.M. Severi,F.M. Reis,F. Petraglia,Tommaso M. Di. Peripartum and postpartum outcomes in uncomplicated term pregnancy following ART: a retrospective cohort study from two Italian obstetric units. Human Reproduction Open. 2018///. 2018:hoy012

S. Yaya,K.S. Reddy,J.M. Belizan,V. Pingray. Non-communicable diseases and reproductive health in sub-Saharan Africa: Bridging the policy-implementation gaps. Reproductive health. 2020///. 17:#pages#

S. Yaya,K.S. Reddy,J.M. Belizan,V. Pingray. Non-communicable diseases and reproductive health in sub-Saharan Africa: Bridging the policy-implementation gaps. Reproductive health. 2020///. 17:#pages#

S. Young,K. Murray,J. Mwesigwa,P. Natureeba,B. Osterbauer,J. Achan,E. Arinaitwe,T. Clark,V. Ades,A. Plenty,E. Charlebois,T. Ruel,M. Kamya,D. Havlir,D. Cohan. Maternal nutritional status predicts adverse birth outcomes among HIV-infected rural Ugandan women receiving combination antiretroviral therapy. PloS one. 2012///. 7:e41934, 2012

S.D. Brown,M.M. Hedderson,S.F. Ehrlich,M.N. Galarce,A.L. Tsai,C.P. Quesenberry,A. Ferrara. Gestational weight gain and optimal wellness (GLOW): rationale and methods for a randomized controlled trial of a lifestyle intervention among pregnant women with overweight or obesity. BMC pregnancy and childbirth. 2019/04/30/. 19:145, 2019

S.H. Ley,J.E. Chavarro,M. Li,W. Bao,S.N. Hinkle,P.L. Wander,J. Rich-Edwards,S. Olsen,A. Vaag,P. Damm,L.G. Grunnet,J.L. Mills,F.B. Hu,C. Zhang. Lactation Duration and Long-term Risk for Incident

Type 2 Diabetes in Women With a History of Gestational Diabetes Mellitus. Diabetes care. 2020/02/10/. 20:#pages#

S.H. Shaw,J.E. Herbers,J.J. Cutuli. Medical and Psychosocial Risk Profiles for Low Birthweight and Preterm Birth. Women's Health Issues. 2019///. 29:400

S.M. Nimbalkar,V.K. Patel,D.V. Patel,A.S. Nimbalkar,A. Sethi,A. Phatak. Effect of early skin-to-skin contact following normal delivery on incidence of hypothermia in neonates more than 1800 g: randomized control trial. Journal of Perinatology. 2014///. 34:364

S.S. Coughlin. Epidemiology of Breast Cancer in Women. Advances in experimental medicine and biology. 2019///. 1152:9

Saunders L, Guldner L, Costet N, Kadhel P, Rouget F, Monfort C, et al. Effect of a Mediterranean diet during pregnancy on fetal growth and preterm delivery: results from a French Caribbean Mother-Child Cohort Study (TIMOUN). Paediatr Perinat Epidemiol 2014;28(3):235-44.

Sawada M, Masuyama H, Hayata K, Kamada Y, Nakamura K, Hiramatsu Y. Pregnancy complications and glucose intolerance in women with polycystic ovary syndrome. Endocr J 2015;62(11):1017-23.

Saxena P, Tyagi S, Prakash A, Nigam A, Trivedi SS. Pregnancy outcome of women with gestational diabetes in a tertiary level hospital of north India. Indian J Community Med 2011;36(2):120-3.

Schoenaker DAJM, Soedamah-Muthu SS, Callaway LK, Mishra GD. Pre-pregnancy dietary patterns and risk of gestational diabetes mellitus: results from an Australian population-based prospective cohort study. Diabetologia 2015;58(12):2726-35.

Scholl TO, Chen X, Goldberg GS, Khusial PR, Stein TP. Maternal diet, C-reactive protein, and the outcome of pregnancy. J Am Coll Nutr 2011;30(4):233-40.

Scifres CM, Feghali MN, Althouse AD, Caritis SN, Catov JM. Effect of excess gestational weight gain on pregnancy outcomes in women with type 1 diabetes. Obstet Gynecol 2014;123(6):1295-302.

Sfandiary, M., Parvizi, S., Almasi, A., and Sharifipour, F.. Effect of pre-pregnancy maternal body mass index on pregnancy outcomes in nulliparous women in the Islamic Republic of Iran. #journal#. 2017. 23:#pages#

Sharam SH, Tangestani A, Faraji R, Zahiri Z, Amiri A. Role of dyslipidemia in preeclamptic overweight pregnant women. Iran J Reprod Med 2012;10(2):105-12. [PMID: http://www.ijrm.ir/library/upload/article/af_62694296)%2089-105-3%20ok2f.pdf]

Shopen N, Schiff E, Koren-Morag N, Grossman E. Factors That Predict the Development of Hypertension in Women With Pregnancy-Induced Hypertension. Am J Hypertens 2016;29(1):141-6.

Sibai BM. Subfertility/infertility and assisted reproductive conception are independent risk factors for pre-eclampsia. BJOG Int J Obstet Gynaecol 2015;122(7):923. [PMID: http://onlinelibrary.wiley.com/journal/10.1111/(ISSN)1471-0528]

Sim SYT, Chin SL, Tan JLK, Brown SJ, Cussons AJ, Stuckey BGA. Polycystic ovary syndrome in type 2 diabetes: does it predict a more severe phenotype? Fertil Steril 2016;106(5):1258-63.

Sina M, Hoy WE, Callaway L, Wang Z. The associations of anthropometric measurements with subsequent gestational diabetes in Aboriginal women. Obes Res Clin Pract 2015;9(5):499-506.

Singh S, Ahmed EB, Egondu SC, Ikechukwu NE. Hypertensive disorders in pregnancy among pregnant women in a Nigerian Teaching Hospital. Niger Med J 2014;55(5):384-8.

Sliwa SA, Must A.. Occupational Physical Activity and Weight-Related Outcomes in Immigrant Mothers. #journal#. 2016. 51:#pages#

Sommer C, Jenum AK, Waage CW, Morkrid K, Sletner L, Birkeland KI. Ethnic differences in BMI, subcutaneous fat, and serum leptin levels during and after pregnancy and risk of gestational diabetes. European journal of endocrinology 2015;172(6):649-56. [PMID: http://www.eje-online.org/content/172/6/649.full.pdf+html]

Sommer C, Morkrid K, Jenum AK, Sletner L, Mosdol A, Birkeland KI. Weight gain, total fat gain and regional fat gain during pregnancy and the association with gestational diabetes: a population-based cohort study. Int J Obes (Lond) 2014;38(1):76-81.

Somprasit C, Tanprasertkul C, Rattanasiri T, Saksiriwutth P, Wongkum J, Kovavisarach E, et al. High pre-pregnancy body mass index and the risk of poor obstetrics outcomes among Asian women using BMI criteria for Asians by World Health Organization Western Pacific Region (WPRO): a large cohort study. J Med Assoc Thai 2015;98 Suppl 2:S101-S107

Sonek, J., Krantz, D., Carmichael, J., Downing, C., Jessup, K., Haidar, Z., Ho, S., Hallahan, T., Kliman, H. J., and McKenna, D.. First-trimester screening for early and late preeclampsia using maternal characteristics, biomarkers, and estimated placental volume. #journal#. 2018. 218:#pages#

Spiegler J, Stichtenoth G, Weichert J, Konig IR, Schlaud M, Wense VD, et al. Pregnancy risk factors for very premature delivery: what role do hypertension, obesity and diabetes play? Arch Gynecol Obstet 2013;288(1):57-64.

Stefanie Rosumeck,Alexander Nast,Corinna Dressler. Ivermectin and permethrin for treating scabies. Cochrane Database of Systematic Reviews 2018;(4). 2018/03/29/. 20:#pages#

Stillbirth linked to obesity and smoking. Nurs N Z 2014;20(6):6

Streiff MB, Haut ER. The CMS ruling on venous thromboembolism after total knee or hip arthroplasty: Weighing risks and benefits. J Am Med Assoc 2009;301(10):1063-5. [PMID: http://jama.ama-assn.org/cgi/reprint/301/10/1063]

Subramaniam A, Jauk VC, Tita A, Harper LM. Interaction between maternal obesity and 1-hour glucose challenge test results on maternal and perinatal outcomes. Am J Perinatol 2015;32(8):771-8.

Swierzewska P, Kosinski M, Wojcik M, Dworacka M, Cypryk K. Family, anthropometric and biochemical factors affecting birth weight of infants born to GDM women. Ginekol Pol 2015;86(7):499-503.

T. Gondwe,K. Betha,G.N. Kusneniwar,C.H. Bunker,G. Tang,H. Simhan,P.S. Reddy,C.L. Haggerty. Maternal Factors Associated with Mode of Delivery in a Population with a High Cesarean Section Rate. J Epidemiol Glob Health. 2019/12//. 9:252

T.C. Plowden,S.M. Zarek,S. Rafique,L.A. Sjaarda,E.F. Schisterman,R.M. Silver,E.H. Yeung,R. Radin,S.N. Hinkle,N. Galai,S.L. Mumford. Preconception leptin levels and pregnancy outcomes: A prospective cohort study. Obesity Science and Practice. 2020///. 298:#pages#

Tabet M, Flick LH, Tuuli MG, Macones GA, Chang JJ. Prepregnancy body mass index in a first uncomplicated pregnancy and outcomes of a second pregnancy. Am J Obstet Gynecol 2015;213(4):548-7.

Tryggvadottir EA, Medek H, Birgisdottir BE, Geirsson RT, Gunnarsdottir I. Association between healthy maternal dietary pattern and risk for gestational diabetes mellitus. Eur J Clin Nutr 2016;70(2):237-42.

Tsvieli O, Sergienko R, Sheiner E. Risk factors and perinatal outcome of pregnancies complicated with cephalopelvic disproportion: a population-based study. Arch Gynecol Obstet 2012;285(4):931-6.

Tuuri AL, Jauhiainen MS, Tikkanen MJ, Kaaja RJ. Systolic blood pressure and fatty acid-binding protein 4 predict pregnancy-induced hypertension in overweight nulliparous women. Placenta 2014;35(10):797-801.

U.M. Schaefer-Graf,S.L. Kjos,O.H. Fauzan,K.J. Buhling,G. Siebert,C. Buhrer,B. Ladendorf,J.W. Dudenhausen,K. Vetter. A randomized trial evaluating a predominantly fetal growth-based strategy to guide management of gestational diabetes in Caucasian women. Diabetes care. 2004///. 27:297

Usynina AA, Grjibovski AM, Odland JO, Krettek A. Social correlates of term small for gestational age babies in a Russian Arctic setting. Int J Circumpolar Health 2016;75:32883

Usynina AA, Postoev VA, Grjibovski AM, Krettek A, Nieboer E, Odland JO, et al. Maternal Risk Factors for Preterm Birth in Murmansk County, Russia: A Registry-Based Study. Paediatr Perinat Epidemiol 2016;30(5):462-72.

V. Stanekova,R.J. Woodman,K. Tremellen. The rate of euploid miscarriage is increased in the setting of adenomyosis. Human Reproduction Open. 2018///. 2018:hoy011

V.K. Kawai,S.K. Nwosu,D. Kurnik,F.E. Harrell,C.M. Stein. Variants in BMI-Associated Genes and Adrenergic Genes are not Associated with Gestational Weight Trajectory. Obesity (Silver Spring, Md.). 2019/07//. 27:1184

Valeska Jennifer Brown,Verena Walsh,William McGuire. Birth room transition support for preterm infants: a Cochrane overview [Protocol]. Cochrane Database of Systematic Reviews 2019;(9). 2019/09/12/. 359:#pages#

Valkama, A.. The effect of dietary counselling on food intakes in pregnant women at risk for gestational diabetes: a secondary analysis of a randomised controlled trial RADIEL. #journal#. 2016. 70:#pages#

van Poppel MNM, Oostdam N, Eekhoff MEW, Wouters MGAJ, van Mechelen W, Catalano PM. Longitudinal relationship of physical activity with insulin sensitivity in overweight and obese pregnant women. J Clin Endocrinol Metab 2013;98(7):2929-35.

Varghese R, Thomas B, Al HM, Rauf A, Al SM, Al SA, et al. The prevalence, risk factors, maternal and fetal outcomes in gestational diabetes mellitus. Int J Drug Dev Res 2012;4(3):356-68. [PMID: http://www.ijddr.in/Dacuments/6/40.pdf]

Vilmi-Kerala T, Palomaki O, Kankkunen P, Juurinen L, Uotila J, Palomaki A. Oxidized LDL, insulin resistance and central blood pressure after gestational diabetes mellitus. Acta Obstet Gynecol Scand 2016;95(12):1425-32.

W. Perng,C.W. Hockett,K.A. Sauder,D. Dabelea. In utero exposure to gestational diabetes mellitus and cardiovascular risk factors in youth: A longitudinal analysis in the EPOCH cohort. Pediatric obesity. 2020///. 49:312611

W. Yang,J. Liu,J. Li,J. Liu,H. Liu,Y. Wang,J. Leng,S. Wang,H. Chen,J.C.N. Chan,Z. Yu,G. Hu,X. Yang. Interactive effects of prepregnancy overweight and gestational diabetes on macrosomia and large for gestational age: A population-based prospective cohort in Tianjin, China. Diabetes research and clinical practice. 2019/08//. 154:82

W. Yu,X. Wang,X. Hu,H. Deng,L. Yang,H. Liang,Q. Si,X. Chen,Q. Li,X. Gu,F. Shen. PREVIOUS DELIVERY OF MACROSOMIA IS ASSOCIATED WITH MATERNAL ADIPOSITY IN LATER LIFE IN CHINESE PAROUS WOMEN WITH NORMAL WEIGHT BEFORE AND/OR AFTER PREGNANCY. Endocrine practice : official journal of the American College of Endocrinology and the American Association of Clinical Endocrinologists. 2019/11//. 25:1176

W. Zheng,W. Huang,Z. Zhang,L. Zhang,Z. Tian,G. Li,W. Zhang. Patterns of Gestational Weight Gain in Women with Overweight or Obesity and Risk of Large for Gestational Age. Obesity facts. 2019///. 12:407

W.H. Teoh,E. Thomas,H.M. Tan. Ultra-low dose combined spinal-epidural anesthesia with intrathecal bupivacaine 3.75 mg for cesarean delivery: a randomized controlled trial. International journal of obstetric anesthesia. 2006///. 15:273

W.L. Lowe,L.P. Lowe,A. Kuang,P.M. Catalano,M. Nodzenski,O. Talbot,W.H. Tam,D.A. Sacks,D. McCance,B. Linder,Y. Lebenthal,J.M. Lawrence,M. Lashley,J.L. Josefson,J. Hamilton,C. Deerochanawong,P. Clayton,W.J. Brickman,A.R. Dyer,D.M. Scholtens,B.E. Metzger,HAPO Follow-up Study Cooperative Research Group. Maternal glucose levels during pregnancy and childhood adiposity in the Hyperglycemia and Adverse Pregnancy Outcome Follow-up Study. Diabetologia. 2019/04//. 62:598

W.R. Bender,N.C. Koelper,M.D. Sammel,C. Durnwald. Validation of a Breastfeeding History Questionnaire for the Risk of In-Hospital Formula Supplementation Among Multiparous Women. Journal of human lactation : official journal of International Lactation Consultant Association. 2019///. 35:665

Walfisch A, Matok I, Sermer C, Koren G. Weight-depression association in a high-risk maternal population. J Matern -Fetal Neonatal Med 2012;25(7):1017-20.

Wang J, Kotani T, Tsuda H, Mano Y, Sumigama S, Li H, et al. Is the serum L-arginine level during early pregnancy a predictor of pregnancy-induced hypertension? J Clin Biochem Nutr 2015;57(1):74-81. [PMID: https://www.jstage.jst.go.jp/article/jcbn/57/1/57_14-104/_pdf]

Wang LF, Wang HJ, Ao D, Liu Z, Wang Y, Yang HX. Influence of pre-pregnancy obesity on the development of macrosomia and large for gestational age in women with or without gestational diabetes mellitus in Chinese population. J Perinatol 2015;35(12):985-90.

Wang T, Leng J, Li N, Martins de Carvalho A, Huang T, Zheng Y, et al. Genetic Predisposition to Polycystic Ovary Syndrome, Postpartum Weight Reduction, and Glycemic Changes: A Longitudinal Study in Women With Prior Gestational Diabetes. J Clin Endocrinol Metab 2015;100(12):E1560-E1567

Wang T, Zhang J, Lu X, Xi W, Li Z. Maternal early pregnancy body mass index and risk of preterm birth. Arch Gynecol Obstet 2011;284(4):813-9.

Wei YM, Yang HX, Zhu WW, Liu XY, Meng WY, Wang YQ, et al. Risk of adverse pregnancy outcomes stratified for pre-pregnancy body mass index. J Matern Fetal Neonatal Med 2016;29(13):2205-9.

Wise J. Women who have had surgery for obesity have raised risk of preterm babies. BMJ 2013;347:no

Wong VW. Gestational diabetes mellitus in five ethnic groups: A comparison of their clinical characteristics. Diabet Med 2012;29(3):366-71.

X. Huang,X. Li,C. Gao,J. Liu,Z. Chen,L. Sheng,J. Xu,Y. Li,R. Zhang,Z. Yu,B. Zha,Y. Wu,M. Yang,H. Ding,T. Sun,Y. Zhang,L. Ma. GESTATIONAL WEIGHT GAIN AS AN INDEPENDENT RISK FACTOR FOR MACROSOMIA IN WOMEN WITH INTERMEDIATE STATE GESTATIONAL BLOOD GLUCOSE. Endocrine practice : official journal of the American College of Endocrinology and the American Association of Clinical Endocrinologists. 2019/11//. 25:1158

X.-L. Li,R. Huang,C. Fang,Y.-F. Wang,X.-Y. Liang. Logistic Regression Analysis of Risk Factors Associated with Spontaneous Abortion after In Vitro Fertilization/Intracytoplasmic Sperm Injection-Embryo Transfer in Polycystic Ovary Syndrome Patients. Reproductive and Developmental Medicine. 2018///. 2:105

Xiao J, Shen F, Xue Q, Chen G, Zeng K, Stone P, et al. Is ethnicity a risk factor for developing preeclampsia? An analysis of the prevalence of preeclampsia in China. J Hum Hypertens 2014;28(11):694-8.

Xie YJ, Peng R, Han L, Zhou X, Xiong Z, Zhang Y, et al. Associations of neonatal high birth weight with maternal pre-pregnancy body mass index and gestational weight gain: a case-control study in women from Chongqing, China. BMJ open 2016;6(8):e010935

Xinxo S, Bimbashi A, Kakarriqi Z, Zaimi E. Association between maternal nutritional status of pre pregnancy, gestational weight gain and preterm birth. Mater socio-med 2013;25(1):6-8.

Y. Gu,J. Lu,W. Li,H. Liu,L. Wang,J. Leng,W. Li,S. Zhang,S. Wang,J. Tuomilehto,Z. Yu,X. Yang,A.A. Baccarelli,L. Hou,G. Hu. Joint Associations of Maternal Gestational Diabetes and Hypertensive Disorders of Pregnancy With Overweight in Offspring. Frontiers in endocrinology. 2019///. 10:645, 2019.:#pages#

Y. Huang,W. Zhang,K. Go,K.J. Tsuchiya,J. Hu,D.W. Skupski,S.Y. Sie,Y. Nomura. Altered growth trajectory in children born to mothers with gestational diabetes mellitus and preeclampsia. Archives of gynecology and obstetrics. 2020/01//. 301:151

Y. Nozawa,M.D.H. Hawlader,F. Ferdous,R. Raqib,F. Tofail,E.C. Ekstrom,Y. Wagatsuma. Effects of intrauterine growth restriction and postnatal nutrition on pediatric asthma in Bangladesh. Journal of developmental origins of health and disease. 2019/12//. 10:627

Y. Shen,J. Leng,W. Li,S. Zhang,H. Liu,P. Shao,P. Wang,L. Wang,H. Tian,C. Zhang,X. Yang,Z. Yu,L. Hou,J. Tuomilehto,G. Hu. Lactation intensity and duration to postpartum diabetes and prediabetes risk in women with gestational diabetes. Diabetes/metabolism research and reviews. 2019///. 35:#pages#

Y. Wu,Y. Kataria,Z. Wang,W.K. Ming,C. Ellervik. Factors associated with successful vaginal birth after a cesarean section: a systematic review and meta-analysis. BMC pregnancy and childbirth. 2019/10/17/. 19:360, 2019

Y. Yang,Q. Lin,Y. Liang,Z. Ruan,B.K. Acharya,S. Zhang,Z. Qian,S.E. McMillin,L. Hinyard,J. Sun,C. Wang,H. Ge,X. Wu,X. Guo,H. Lin. Maternal air pollution exposure associated with risk of congenital heart defect in pre-pregnancy overweighted women. The Science of the total environment. 2020/04/10/. 712:136470

Y. Zhang,L. Wang,W. Yang,D. Niu,C. Li,L. Wang,P. Gu,Y. Xia,Y. Shen,J. Yan,Q. Zhao,K. Mu,W. Yan. Effectiveness of Low Glycemic Index Diet Consultations Through a Diet Glycemic Assessment App Tool on Maternal and Neonatal Insulin Resistance: A Randomized Controlled Trial. JMIR mHealth and uHealth. 2019/04/18/. 7:e12081, 2019

Y.-H. Chen,L. Fu,J.-H. Hao,H. Wang,C. Zhang,F.-B. Tao,D.-X. Xu. Influent factors of gestational vitamin D deficiency and its relation to an increased risk of preterm delivery in Chinese population. Sci Rep. 2018///. 8:3608

Y.E.G. Timmermans,K.D.G. van de Kant,D. Reijnders,L.M.P. Kleijkers,E. Dompeling,B.W. Kramer,L.J.I. Zimmermann,R.P.M. Steegers-Theunissen,M.E.A. Spaanderman,A.C.E. Vreugdenhil. Towards Prepared mums (TOP-mums) for a healthy start, a lifestyle intervention for women with overweight and a child wish: study protocol for a randomised controlled trial in the Netherlands. BMJ open. 2019/11/19/. 9:e030236, 2019

Y.L. Chen,L.L. Han,X.L. Shi,W.J. Su,W. Liu,L.Y. Wang,P.Y. Huang,M.Z. Lin,H.Q. Song,X.J. Li. Adverse pregnancy outcomes on the risk of overweight offspring: a population-based retrospective study in Xiamen, China. Sci Rep. 2020/01/31/. 10:1549, 2020

Y.Q. Xiong,Y.M. Liu,Y.N. Qi,C.R. Liu,J. Wang,L. Li,K. Zou,J. Tan,X. Sun. Association between prepregnancy subnormal body weight and obstetrical outcomes after autologous in vitro fertilization cycles: systematic review and meta-analysis. Fertility and sterility. 2020/02//. 113:344

Yang H, Wei Y, Gao X, Xu X, Fan L, He J, et al. Risk factors for gestational diabetes mellitus in Chinese women: a prospective study of 16,286 pregnant women in China. Diabet Med 2009;26(11):1099-104.

Yang S, Zhou A, Xiong C, Yang R, Bassig BA, Hu R, et al. Parental Body Mass Index, Gestational Weight Gain, and Risk of Macrosomia: a Population-Based Case-Control Study in China. Paediatr Perinat Epidemiol 2015;29(5):462-71.

Yoo HJ, Choi KM, Baik SH, Park JH, Shin SA, Hong SC, et al. Influences of body size phenotype on the incidence of gestational diabetes needing prescription; analysis by Korea National Health Insurance (KNHI) claims and the National Health Screening Examination (NHSE) database. Metabolism 2016;65(9):1259-66.

Yousuf F, Naru T, Sheikh S. Effect of body mass index on outcome of labour induction. JPMA J Pak Med Assoc 2016;66(5):598-601.

Zhang CM, Zhao Y, Li R, Yu Y, Yan Ly, Li L, et al. Metabolic heterogeneity of follicular amino acids in polycystic ovary syndrome is affected by obesity and related to pregnancy outcome. BMC Pregnancy Childbirth 2014;14:11

Zhang Y, Zhang HH, Lu JH, Zheng SY, Long T, Li YT, et al. Changes in serum adipocyte fatty acid-binding protein in women with gestational diabetes mellitus and normal pregnant women during mid- and late pregnancy. J diabetes investig 2016;7(5):797-804.

Zhao YN, Li Q, Li YC. Effects of body mass index and body fat percentage on gestational complications and outcomes. J Obstet Gynaecol Res 2014;40(3):705-10.

Zhou A, Xiong C, Hu R, Zhang Y, Bassig BA, Triche E, et al. Pre-Pregnancy BMI, Gestational Weight Gain, and the Risk of Hypertensive Disorders of Pregnancy: A Cohort Study in Wuhan, China. PloS one 2015;10(8):e0136291

Zhou H, Liu Y, Liu L, Zhang M, Chen X, Qi Y. Maternal pre-pregnancy risk factors for miscarriage from a prevention perspective: a cohort study in China. Eur J Obstet Gynecol Reprod Biol 2016;206:57-63.

Zolotusca L, Jorgensen P, Popovici O, Pistol A, Popovici F, Widdowson MA, et al. Risk factors associated with fatal influenza, Romania, October 2009-May 2011. Influenza other respi viruses 2014;8(1):8-12.

No relevant comparator (obese women with predictor not being compared to obese women without predictor)

A. Simon,M. Pratt,B. Hutton,B. Skidmore,R. Fakhraei,N. Rybak,D.J. Corsi,M. Walker,M.P. Velez,G.N. Smith,L.M. Gaudet. Guidelines for the management of pregnant women with obesity: A systematic review. Obesity reviews : an official journal of the International Association for the Study of Obesity. 2020/03//. 21:e12972, 2020

A.A. Adane,C.C.J. Shepherd,F.J. Lim,S.W. White,B.M. Farrant,H.D. Bailey. The impact of pre-pregnancy body mass index and gestational weight gain on placental abruption risk: a systematic review and meta-analysis. Archives of gynecology and obstetrics. 2019///. 300:1201

A.A. Panjwani,Y. Ji,J.W. Fahey,A. Palmer,G. Wang,X. Hong,B. Zuckerman,X. Wang. Maternal Obesity/Diabetes, Plasma Branched-Chain Amino Acids, and Autism Spectrum Disorder Risk in Urban Low-Income Children: Evidence of Sex Difference. Autism Research. 2019///. 12:1562

A.G. Euser,A. Hammes,J.T. Ahrendsen,B. Neshek,D.A. Weitzenkamp,J. Gutierrez,P. Koivunen,C.G. Julian,L.G. Moore. Gestational diabetes prevalence at moderate and high altitude. High Altitude Medicine and Biology. 2018///. 19:367

A.M. Eudy,A.M. Siega-Riz,S.M. Engel,N. Franceschini,A.G. Howard,M.E.B. Clowse,M. Petri. Preconceptional cardiovascular health and pregnancy outcomes in women with systemic lupus erythematosus. Journal of Rheumatology. 2019///. 46:70

A.M. Fallatah,H.M. Babatin,K.M. Nassibi,M.K. Banweer,M.N. Fayoumi,A.M. Oraif. Maternal and Neonatal Outcomes among Obese Pregnant Women in King Abdulaziz University Hospital: A Retrospective Single-Center Medical Record Review. Med Arh. 2019/12//. 73:425

A.M. Panaitescu,D. Rotaru,I. Ban,G. Peltecu,A.M. Zagrean. The prevalence of underweight, overweight and obesity in a Romanian population in the first trimester of pregnancy - clinical implications. Acta Endocrinologica. 2019/07//. 15:323

A.N. Battarbee,S. Vaz,D.M. Stamilio. The association between delayed amniotomy and adverse outcomes in labor induction. European Journal of Obstetrics and Gynecology and Reproductive Biology. 2020///. 247:85

A.S. Basha,K.M. Fram,F.M. Thekrallah,Z.A. Irshaid,A.M. Maswady,Z.N. Obeidat. Prevalence of gestational diabetes and contributing factors among pregnant Jordanian women attending Jordan University Hospital. International Journal of Diabetes in Developing Countries. 2019///. 39:132

A.W.G. Ratnasiri,H.C. Lee,S. Lakshminrusimha,S.S. Parry,V.N. Arief,I.H. DeLacy,J.S. Yang,R.J. DiLibero,J. Logan,K.E. Basford. Trends in maternal prepregnancy body mass index (BMI) and its association with birth and maternal outcomes in California, 2007-2016: A retrospective cohort study. PloS one. 2019///. 14:e0222458, 2019

Abbasi N, Balayla J, Laporta DP, Kezouh A, Abenhaim HA. Trends, risk factors and mortality among women with venous thromboembolism during labour and delivery: a population-based study of 8 million births. Arch Gynecol Obstet 2014;289(2):275-84.

Abdelmaboud MO, Ryan H, Hession M, Avalos G, Morrison JJ. Moderate and extreme maternal obesity. Ir Med J 2012;105(5):146-8.

Abell SK, Boyle JA, de Court, Soldatos G, Wallace EM, Zoungas S, et al. Impact of type 2 diabetes, obesity and glycaemic control on pregnancy outcomes. Aust N Z J Obstet Gynaecol 2016;

Abenhaim HA, Alrowaily N, Czuzoj-Shulman N, Spence AR, Klam SL. Pregnancy outcomes in women with bariatric surgery as compared with morbidly obese women. J Matern Fetal Neonatal Med 2016;29(22):3596-601.

Abrar S, Abrar T, Sayyed E. Frequency of intrapartum complications in pregnant obese women at term. J Med Sci 2016;24(2):103-6. [PMID: http://www.jmedsci.com/admin/uploadpic/JMS-12-April2016Vol24No2.pdf]

Adams TD, Hammoud AO, Davidson LE, Laferrere B, Fraser A, Stanford JB, et al. Maternal and neonatal outcomes for pregnancies before and after gastric bypass surgery. Int J Obes (Lond) 2015;39(4):686-94.

Adesina K, Aderibigbe S, Fawole A, Ijaiya M, Olarinoye A. Pregnancy outcome of the obese in Ilorin. Obstet med 2011;4(4):160-3.

Agrawal S, Singh A. Obesity or Underweight-What is Worse in Pregnancy? J Obstet Gynaecol India 2016;66(6):448-52.

Ahmed SR, Ellah MAA, Mohamed OA, Eid HM. Prepregnancy obesity and pregnancy outcome. Int J Health Sci (Qassim) 2009;3(2):203-8.

Aimukhametova G, Ukybasova T, Hamidullina Z, Zhubanysheva K, Harun-Or-Rashid M, Yoshida Y, et al. The impact of maternal obesity on mother and neonatal health: study in a tertiary hospital of Astana, Kazakhstan. Nagoya J Med Sci 2012;74(1-2):83-92.

Akhmetovna KL, Eduardovna TA, Vladimirovna CL, Vladislavovich MN. Metabolic Disturbances in Obese Pregnant Residents of an Industrial Region (The Urals, Russia). Oman med j 2016;31(3):211-6.

Al Busaidi I, Al-Farsi Y, Ganguly S, Gowri V. Obstetric and non-obstetric risk factors for cesarean section in oman. Oman med j 2012;27(6):478-81.

Al-Hakmani FM, Al-Fadhil FA, Al-Balushi LH, Al-Harthy NA, Al-Bahri ZA, Al-Rawahi NA, et al. The Effect of Obesity on Pregnancy and its Outcome in the Population of Oman, Seeb Province. Oman med j 2016;31(1):12-7.

Al-Khaduri MM, Abudraz RM, Rizvi SG, Al-Farsi YM. Risk factors profile of shoulder dystocia in Oman: A case control study. Oman med j 2014;29(5):325-9. [PMID: http://www.omjournal.org/fultext_PDF.aspx?DetailsID=560&pdf=images/560_M_Deatials_Pdf_.pdf&type=pdf]

Al-Obaidly S, Parrish J, Murphy KE, Maxwell C. Maternal pre-gravid body mass index and obstetric outcomes in twin gestations. J Perinatol 2014;34(6):425-8.

Alberico S, Montico M, Barresi V, Monasta L, Businelli C, Soini V, et al. The role of gestational diabetes, pre-pregnancy body mass index and gestational weight gain on the risk of newborn macrosomia: results from a prospective multicentre study. BMC Pregnancy Childbirth 2014;14:23

Alhaj AM, Radi EA, Adam I. Epidemiology of preterm birth in Omdurman Maternity hospital, Sudan. J Matern Fetal Neonatal Med 2010;23(2):131-4.

Aliyu MH, Alio AP, Lynch O'N, Mbah A, Salihu HM. Maternal pre-gravid body weight and risk for placental abruption among twin pregnancies. J Matern Fetal Neonatal Med 2009;22(9):745-50.

Alves E, Azevedo A, Rodrigues T, Santos AC, Barros H. Impact of risk factors on hypertensive disorders in pregnancy, in primiparae and multiparae. Ann Hum Biol 2013;40(5):377-84.

Amsalem D, Aricha-Tamir B, Levi I, Shai D, Sheiner E. Obstetric outcomes after restrictive bariatric surgery: what happens after 2 consecutive pregnancies? Surg obes relat dis 2014;10(3):445-9.

Ananth CV, Keyes KM, Wapner RJ. Pre-eclampsia rates in the United States, 1980-2010: age-period-cohort analysis. BMJ 2013;347:f6564

Anderson KG, Spicer P, Peercy MT. Obesity, Diabetes, and Birth Outcomes Among American Indians and Alaska Natives. Matern Child Health J 2016;20(12):2548-56.

Anderson NH, Sadler LC, Stewart AW, Fyfe EM, McCowan LME. Independent risk factors for infants who are small for gestational age by customised birthweight centiles in a multi-ethnic New Zealand population. Aust N Z J Obstet Gynaecol 2013;53(2):136-42.

Antony KM, Ma J, Mitchell KB, Racusin DA, Versalovic J, Aagaard K. The preterm placental microbiome varies in association with excess maternal gestational weight gain. Am J Obstet Gynecol 2015;212(5):653-16.

Armstrong EM, Bellone JM, Hornsby LB, Treadway S, Phillippe HM. Pregnancy-Related Venous Thromboembolism. J Pharm Pract 2014;27(3):243-52.

Arrowsmith S, Wray S, Quenby S. Maternal obesity and labour complications following induction of labour in prolonged pregnancy. BJOG 2011;118(5):578-88.

Asif S, Ejaz S, Waheed K. Maternal outcome in obese and non-obese women. Pak J Med Health Sci 2016;10(1):278-80. [PMID: http://www.pjmhsonline.com/2016/jan_march/pdf/278%20%20%20Maternal%20Outcome%20in%20Obese%20and%20Non-Obese%20Women.pdf]

Asim SS, Naeem H. Pregnancy with obesity -a risk factor for PIH. J Liaquat Univ Med Health Sci 2010;9(3):125-9. [PMID: http://beta.lumhs.edu.pk/jlumhs/Vol09No03/pdfs/v9n3oa02.pdf]

Athukorala C, Rumbold AR, Willson KJ, Crowther CA. The risk of adverse pregnancy outcomes in women who are overweight or obese. BMC Pregnancy Childbirth 2010;10:56

Aune D, Saugstad OD, Henriksen T, Tonstad S. Maternal body mass index and the risk of fetal death, stillbirth, and infant death: a systematic review and meta-analysis. JAMA 2014;311(15):1536-46.

Avci ME, Sanlikan F, Celik M, Avci A, Kocaer M, Gocmen A. Effects of maternal obesity on antenatal, perinatal and neonatal outcomes. J Matern Fetal Neonatal Med 2015;28(17):2080-3.

B. Eshetu,Y. Sintayehu,B. Mekonnen,W. Daba. Birth Outcomes among Diabetic Mothers Who Delivered in Tikur Anbessa Specialized Hospital, Addis Ababa, Ethiopia. #journal#. 2019///. 2019:6942617

B. Masturzo,V. Franze,C. Germano,R. Attini,G. Gennarelli,A. Lezo,A. Rolfo,C. Plazzotta,E. Brunelli,A. Youssef,T. Todros,A. Farina. Risk of adverse pregnancy outcomes by pre-pregnancy Body Mass Index among Italian population: a retrospective population-based cohort study on 27,807 deliveries. Archives of gynecology and obstetrics. 2019/04//. 299:983

B.R. Allman,Fuentes E. Diaz,D.K. Williams,D.E. Turner,A. Andres,E. Borsheim. Obesity Status Affects the Relationship Between Protein Intake and Insulin Sensitivity in Late Pregnancy. Nutrients. 2019/09/11/. 11:#pages#

Badon SE, Dyer AR, Josefson JL, HAPO Study Cooperative Research Group. Gestational weight gain and neonatal adiposity in the Hyperglycemia and Adverse Pregnancy Outcome study-North American region. Obesity (Silver Spring) 2014;22(7):1731-8.

Badran EF, Abu Libdeh AM, Kasaleh F, Saleh S, Basha A, Khader Y, et al. Impact of maternal overweight and obesity on perinatal outcomes. Jordan Med J 2014;48(2):121-31. [PMID: http://journals.ju.edu.jo/JMJ/article/viewFile/6835/3937]

Baer RJ, Chambers CD, Jones KL, Shew SB, MacKenzie TC, Shaw GM, et al. Maternal factors associated with the occurrence of gastroschisis. Am J Med Genet A 2015;167(7):1534-41.

Barnes RA, Wong T, Ross GP, Jalaludin BB, Wong VW, Smart CE, et al. A novel validated model for the prediction of insulin therapy initiation and adverse perinatal outcomes in women with gestational diabetes mellitus. Diabetologia 2016;59(11):2331-8.

Baron CM, Girling LG, Mathieson AL, Menticoglou SM, Seshia MM, Cheang MS, et al. Obstetrical and neonatal outcomes in obese parturients. J Matern Fetal Neonatal Med 2010;23(8):906-13.

Bartsch E, Medcalf KE, Park AL, Ray JG, High Risk of Pre-eclampsia Identification Group. Clinical risk factors for pre-eclampsia determined in early pregnancy: systematic review and meta-analysis of large cohort studies. BMJ 2016;353:i1753

Basraon SK, Mele L, Myatt L, Roberts JM, Hauth JC, Leveno KJ, et al. Relationship of Early Pregnancy Waist-to-Hip Ratio versus Body Mass Index with Gestational Diabetes Mellitus and Insulin Resistance. Am J Perinatol 2016;33(1):114-21.

Basu JK, Jeketera CM, Basu D. Obesity and its outcomes among pregnant South African women. Int J Gynaecol Obstet 2010;110(2):101-4.

Bautista-Castano I, Henriquez-Sanchez P, Aleman-Perez N, Garcia-Salvador JJ, Gonzalez-Quesada A, Garcia-Hernandez JA, et al. Maternal obesity in early pregnancy and risk of adverse outcomes. PloS one 2013;8(11):e80410

Belogolovkin V, Crisan L, Lynch O'N, Weldeselasse H, August EM, Alio AP, et al. Neonatal outcomes of successful VBAC among obese and super-obese mothers. J Matern Fetal Neonatal Med 2012;25(6):714-8.

Bener A, Saleh NM. The impact of socio-economic, lifestyle habits, and obesity in developing of pregnancy-induced hypertension in fast-growing country: global comparisons. Clin Exp Obstet Gynecol 2013;40(1):52-7.

Berger KE, Masterson J, Mascardo J, Grapa J, Appanaitis I, Temengil E, et al. The Effects of Chewing Betel Nut with Tobacco and Pre-pregnancy Obesity on Adverse Birth Outcomes Among Palauan Women. Matern Child Health J 2016;20(8):1696-703.

Berglund SK, Garcia-Valdes L, Torres-Espinola FJ, Segura MT, Martinez-Zaldivar C, Aguilar MJ, et al. Maternal, fetal and perinatal alterations associated with obesity, overweight and gestational diabetes: an observational cohort study (PREOBE). BMC public health 2016;16:207

Block SR, Watkins SM, Salemi JL, Rutkowski R, Tanner JP, Correia JA, et al. Maternal pre-pregnancy body mass index and risk of selected birth defects: evidence of a dose-response relationship. Paediatr Perinat Epidemiol 2013;27(6):521-31.

Blomberg MI, Kallen B. Maternal obesity and morbid obesity: the risk for birth defects in the offspring. Birth Defects Res Part A Clin Mol Teratol 2010;88(1):35-40.

Blondon M, Harrington LB, Boehlen F, Robert-Ebadi H, Righini M, Smith NL. Pre-pregnancy BMI, delivery BMI, gestational weight gain and the risk of postpartum venous thrombosis. Thromb Res 2016;145:151-6.

Bodnar LM, Parks WT, Perkins K, Pugh SJ, Platt RW, Feghali M, et al. Maternal prepregnancy obesity and cause-specific stillbirth. Am J Clin Nutr 2015;102(4):858-64.

Boghossian NS, Yeung E, Mendola P, Hinkle SN, Laughon SK, Zhang C, et al. Risk factors differ between recurrent and incident preeclampsia: a hospital-based cohort study. Ann Epidemiol 2014;24(12):871-7e3.

Bonnesen B, Secher NJ, Moller LK, Rasmussen S, Andreasen KR, Renault K. Pregnancy outcomes in a cohort of women with a preconception body mass index >50 kg/m2. Acta Obstet Gynecol Scand 2013;92(9):1111-4.

Briese V, Voigt M, Wisser J, Borchardt U, Straube S. Risks of pregnancy and birth in obese primiparous women: an analysis of German perinatal statistics. Arch Gynecol Obstet 2011;283(2):249-53.

Bryant M, Santorelli G, Lawlor DA, Farrar D, Tuffnell D, Bhopal R, et al. A comparison of South Asian specific and established BMI thresholds for determining obesity prevalence in pregnancy and predicting pregnancy complications: findings from the Born in Bradford cohort. Int J Obes (Lond) 2014;38(3):444-50.

C. Skoglund,Kallner H. Kopp,A. Skalkidou,A.K. Wikstrom,C. Lundin,S. Hesselman,A. Wikman,Poromaa Sundstrom. Association of Attention-Deficit/Hyperactivity Disorder With Teenage Birth Among Women and Girls in Sweden. JAMA network open. 2019/10/02/. 2:e1912463

C.L. Moraes,C.R. Mendonca,N.C.E. Melo,W.N.D. Amaral. Prevalencia e associacao de anomalias congenitas de acordo com indice de massa corporal materno: Estudo transversal, Prevalence and Association of Congenital Anomalies According to the Maternal Body Mass Index: Cross-Sectional Study. Revista brasileira de ginecologia e obstetricia : revista da Federacao Brasileira das Sociedades de Ginecologia e Obstetricia. 2019///. 41:280

Campbell SK, Lynch J, Esterman A, McDermott R. Pre-pregnancy predictors of hypertension in pregnancy among Aboriginal and Torres Strait Islander women in north Queensland, Australia; a prospective cohort study. BMC public health 2013;13:138

Carmichael SL, Blumenfeld YJ, Mayo J, Wei E, Gould JB, Stevenson DK, et al. Prepregnancy Obesity and Risks of Stillbirth. PloS one 2015;10(10):e0138549

Cavicchia PP, Liu J, Adams SA, Steck SE, Hussey JR, Daguise VG, et al. Proportion of gestational diabetes mellitus attributable to overweight and obesity among non-Hispanic black, non-Hispanic white, and Hispanic women in South Carolina. Matern Child Health J 2014;18(8):1919-26.

Chen M, McNiff C, Madan J, Goodman E, Davis JM, Dammann O. Maternal obesity and neonatal Apgar scores. J Matern Fetal Neonatal Med 2010;23(1):89-95.

Chevrot A, Kayem G, Coupaye M, Lesage N, Msika S, Mandelbrot L. Impact of bariatric surgery on fetal growth restriction: experience of a perinatal and bariatric surgery center. Am J Obstet Gynecol 2016;214(5):655-7.

Chung JH, Melsop KA, Gilbert WM, Caughey AB, Walker CK, Main EK. Increasing pre-pregnancy body mass index is predictive of a progressive escalation in adverse pregnancy outcomes. J Matern Fetal Neonatal Med 2012;25(9):1635-9.

Clapperton M, Jarvis J, Mungrue K. Is gestational diabetes mellitus an important contributor to metabolic disorders in trinidad and tobago? Obstet Gynecol Int 2009;2009:289329

Cnattingius S, Villamor E, Johansson S, Edstedt Bonamy AK, Persson M, Wikstrom AK, et al. Maternal obesity and risk of preterm delivery. JAMA 2013;309(22):2362-70.

Cnattingius S, Villamor E. Weight change between successive pregnancies and risks of stillbirth and infant mortality: a nationwide cohort study. Lancet 2016;387(10018):558-65.

Cody F, Unterscheider J, Daly S, Geary MP, Kennelly MM, McAuliffe FM, et al. The effect of maternal obesity on sonographic fetal weight estimation and perinatal outcome in pregnancies complicated by fetal growth restriction. J Clin Ultrasound 2016;44(1):34-9.

Coghill AE, Hansen S, Littman AJ. Risk factors for eclampsia: a population-based study in Washington State, 1987-2007. Am J Obstet Gynecol 2011;205(6):553-7.

Conner SN, Tuuli MG, Longman RE, Odibo AO, Macones GA, Cahill AG. Impact of obesity on incision-to-delivery interval and neonatal outcomes at cesarean delivery. Am J Obstet Gynecol 2013;209(4):386

Conner SN, Verticchio JC, Tuuli MG, Odibo AO, Macones GA, Cahill AG. Maternal obesity and risk of postcesarean wound complications. Am J Perinatol 2014;31(4):299-304.

Crane JMG, Murphy P, Burrage L, Hutchens D. Maternal and perinatal outcomes of extreme obesity in pregnancy. J Obstet Gynaecol Can 2013;35(7):606-11.

Cunningham CE, Teale GR. A profile of body mass index in a large rural Victorian obstetric cohort. Med J Aust 2013;198(1):39-42.

D. Leonard,P. Buttner,F. Thompson,M. Makrides,R. McDermott. Anaemia in pregnancy among Aboriginal and Torres Strait Islander women of Far North Queensland: A retrospective cohort study. Nutrition and dietetics: the journal of the Dietitians Association of Australia. 2018///. 75:457

da Fonseca CRB, Strufaldi MWL, de Carvalho LR, Puccini RF. Risk factors for low birth weight in Botucatu city, SP state, Brazil: a study conducted in the public health system from 2004 to 2008. BMC Res Notes 2012;5:60

Dalfra MG, Busetto L, Chilelli NC, Lapolla A. Pregnancy and foetal outcome after bariatric surgery: a review of recent studies. J Matern Fetal Neonatal Med 2012;25(9):1537-43.

Dave A, Maru L, Daksha S, Natu N. Weight does matter! A study of effect of obesity on pregnancy and its outcome. J SAFOG 2013;5(3):107-10. [PMID: http://www.jsafog.com/]

David MH, Gibbons KS, Flenady VJ, Callaway LK. Overweight and obesity in Australian mothers: Epidemic or endemic? Med J Aust 2012;196(3):184-8. [PMID: https://www.mja.com.au/sites/default/files/issues/196_03_200212/mci11120_fm.pdf]

de Graaff EC, Wijs LA, Leemaqz S, Dekker GA. Risk Factors for Stillbirth in a Socio-Economically Disadvantaged Urban Australian Population. J Matern Fetal Neonatal Med 2016;1-15.

de Jongh BE, Paul DA, Hoffman M, Locke R. Effects of pre-pregnancy obesity, race/ethnicity and prematurity. Matern Child Health J 2014;18(3):511-7.

Denison FC, Norwood P, Bhattacharya S, Duffy A, Mahmood T, Morris C, et al. Association between maternal body mass index during pregnancy, short-term morbidity, and increased health service costs: a population-based study. BJOG 2014;121(1):72-82.

Dennis AT, Lamb KE Story. Associations between maternal size and health outcomes for women undergoing caesarean section: a multicentre prospective observational study (The MUM SIZE Study). #journal#. 2017. 7:#pages#

Deshmukh VL, Jadhav M, Yelikar K. Impact of HIGH BMI on Pregnancy: Maternal and Foetal Outcome. J Obstet Gynaecol India 2016;66(Suppl 1):192-7.

Djelantik AAAM, Kunst AE, van der Wal MF, Smit HA, Vrijkotte TGM. Contribution of overweight and obesity to the occurrence of adverse pregnancy outcomes in a multi-ethnic cohort: population attributive fractions for Amsterdam. BJOG 2012;119(3):283-90.

Dodd JM, Grivell RM, Nguyen AM, Chan A, Robinson JS. Maternal and perinatal health outcomes by body mass index category. Aust N Z J Obstet Gynaecol 2011;51(2):136-40.

Dosch NC, Guslits EF, Weber MB, Murray SE, Ha B, Coe CL, et al. Maternal obesity affects inflammatory and iron indices in umbilical cord blood. J Pediatr 2016;172:20-8. [PMID: http://www.elsevier.com/inca/publications/store/6/2/3/3/1/1/index.htt]

Dubourdeau AL, Berdin A, Mangin M, Ramanah R, Maillet R, Riethmuller D. [Obesity and primiparity: Risky delivery?]. J Gynecol Obstet Biol Reprod (Paris) 2015;44(8):699-705.

Ducarme G, Chesnoy V, Lemarie P, Koumare S, Krawczykowski D. Pregnancy outcomes after laparoscopic sleeve gastrectomy among obese women. Int J Gynaecol Obstet 2015;130(2):127-31.

E. Larque,I. Labayen,C.-E. Flodmark,I. Lissau,S. Czernin,L.A. Moreno,A. Pietrobelli,K. Widhalm. From conception to infancy - early risk factors for childhood obesity. Nature Reviews Endocrinology. 2019///. 15:456

E.O. Ugwu,H.E. Onah,S.N. Obi,C.I. Onwuka,I.E. Menuba,I.I. Okafor. Patterns of gestational weight gain and its association with birthweight in Nigeria. Nigerian journal of clinical practice. 2017///. 20:754

Ebrahimi-Mameghani M, Mehrabi E, Kamalifard M, Yavarikia P. Correlation between Body Mass Index and Central Adiposity with Pregnancy Complications in Pregnant Women. Health promot perspect 2013;3(1):73-9.

Egeland GM, Klungsoyr K, Oyen N, Tell GS, Naess O, Skjaerven R. Preconception Cardiovascular Risk Factor Differences Between Gestational Hypertension and Preeclampsia: Cohort Norway Study. Hypertension 2016;67(6):1173-80.

Ehrenthal DB, Jurkovitz C, Hoffman M, Jiang X, Weintraub WS. Prepregnancy body mass index as an independent risk factor for pregnancy-induced hypertension. J Womens Health (Larchmt) 2011;20(1):67-72.

El-Chaar D, Finkelstein SA, Tu X, Fell DB, Gaudet L, Sylvain J, et al. The impact of increasing obesity class on obstetrical outcomes. J Obstet Gynaecol Can 2013;35(3):224-33.

El-Gilany AH, Hammad S. Body mass index and obstetric outcomes in pregnant in Saudi Arabia: a prospective cohort study. Ann Saudi Med 2010;30(5):376-80.

Engelstad HJ, Roghair RD, Calarge CA, Colaizy TT, Stuart S, Haskell SE. Perinatal outcomes of pregnancies complicated by maternal depression with or without selective serotonin reuptake inhibitor therapy. Neonatology 2014;105(2):149-54.

Ephraim RKD, Osakunor DNM, Denkyira SW, Eshun H, Amoah S, Anto EO. Serum calcium and magnesium levels in women presenting with pre-eclampsia and pregnancy-induced hypertension: a case-control study in the Cape Coast metropolis, Ghana. BMC Pregnancy Childbirth 2014;14:390

Erem C, Kuzu UB, Deger O, Can G. Prevalence of gestational diabetes mellitus and associated risk factors in Turkish women: the Trabzon GDM Study. Arch Med Sci 2015;11(4):724-35.

Ezeanochie MC, Ande AB, Olagbuji BN. Maternal obesity in early pregnancy and subsequent pregnancy outcome in a Nigerian population. Afr J Reprod Health 2011;15(4):55-9.

F. Fang,Q.Y. Zhang,J. Zhang,X.P. Lei,Z.C. Luo,H.D. Cheng. Risk factors for recurrent macrosomia and child outcomes. World journal of pediatrics : WJP. 2019/06//. 15:289

F.G. Tela,A.M. Bezabih,A.K. Adhanu,K.B. Tekola. Fetal macrosomia and its associated factors among singleton live-births in private clinics in Mekelle city, Tigray, Ethiopia. BMC pregnancy and childbirth. 2019/07/01/. 19:219, 2019

Facco FL, Ouyang DW, Zee PC, Strohl AE, Gonzalez AB, Lim C, et al. Implications of sleep-disordered breathing in pregnancy. Am J Obstet Gynecol 2014;210(6):559-6.

Fang R, Dawson A, Lohsoonthorn V, Williams MA. Risk factors of early and late onset preeclampsia among Thai women. Asian Biomed 2009;3(5):477-86.

Farah N, McGoldrick A, Fattah C, O'Connor N, Kennelly MM, Turner MJ. Body Mass Index (BMI) and Glucose Intolerance during Pregnancy in White European Women. J reprod infertil 2012;13(2):95-9.

Farah, N.. Body Mass Index (BMI) and glucose intolerance during pregnancy in white European women. #journal#. 2012. 13:#pages#

Farrar D. Hyperglycemia in pregnancy: prevalence, impact, and management challenges. Int J Women Health 2016;8:519-27.

Flenady V, Koopmans L, Middleton P, Froen JF, Smith GC, Gibbons K, et al. Major risk factors for stillbirth in high-income countries: a systematic review and meta-analysis. Lancet 2011;377(9774):1331-40.

Foo XY, Greer RM, Kumar S. Impact of Maternal Body Mass Index on Intrapartum and Neonatal Outcomes in Brisbane, Australia, 2007 to 2013. Birth 2016;43(4):358-65.

Foo, X. Y., Greer, R. M., and Kumar, S.. Impact of Maternal Body Mass Index on Intrapartum and Neonatal Outcomes in Brisbane, Australia, 2007 to 2013. #journal#. 2016. 43:#pages#

Fouelifack FY, Fouedjio JH, Fouogue JT, Sando Z, Fouelifa LD, Mbu RE. Associations of body mass index and gestational weight gain with term pregnancy outcomes in urban Cameroon: a retrospective cohort study in a tertiary hospital. BMC Res Notes 2015;8:806

Fox NS, Roman AS, Saltzman DH, Hourizadeh T, Hastings J, Rebarber A. Risk factors for preeclampsia in twin pregnancies. Am J Perinatol 2014;31(2):163-6.

Fox NS, Roman AS, Saltzman DH, Klauser CK, Rebarber A. Obesity and adverse pregnancy outcomes in twin pregnancies. J Matern Fetal Neonatal Med 2014;27(4):355-9.

Fyfe EM, Thompson JMD, Anderson NH, Groom KM, McCowan LM. Maternal obesity and postpartum haemorrhage after vaginal and caesarean delivery among nulliparous women at term: a retrospective cohort study. BMC Pregnancy Childbirth 2012;12:no. [PMID: http://www.biomedcentral.com/1471-2393/12/112]

G. Aydin,C.D. Sayan. Is body mass index a risk factor for low cerebral oxygenation during spinal anesthesia in women undergoing cesarean section? A preliminary study. Turkish Journal of Medical Sciences. 2019/06/18/. 49:854

G. Bourjeily,V.A. Danilack,M.H. Bublitz,J. Muri,K. Rosene-Montella,H. Lipkind. Maternal obstructive sleep apnea and neonatal birth outcomes in a population based sample. Sleep medicine. 2020/02//. 66:233

G. Domanski,A.E. Lange,T. Ittermann,H. Allenberg,R.A. Spoo,M. Zygmunt,M. Heckmann. Evaluation of neonatal and maternal morbidity in mothers with gestational diabetes: a population-based study. BMC pregnancy and childbirth. 2018///. 18:367

Gaillard R, Steegers EAP, Hofman A, Jaddoe VWV. Associations of maternal obesity with blood pressure and the risks of gestational hypertensive disorders. The Generation R Study. J Hypertens 2011;29(5):937-44.

Garabedian MJ, Williams CM, Pearce CF, Lain KY, Hansen WF. Extreme morbid obesity and labor outcome in nulliparous women at term. Am J Perinatol 2011;28(9):729-34.

Gardosi J, Madurasinghe V, Williams M, Malik A, Francis A. Maternal and fetal risk factors for stillbirth: population based study. BMJ 2013;346:f108

Gaudet L, Tu X, Fell D, El-Chaar D, Wu Wen S, Walker M. The effect of maternal Class III obesity on neonatal outcomes: a retrospective matched cohort study. J Matern Fetal Neonatal Med 2012;25(11):2281-6.

Gaudet L, Wen SW, Walker M. The combined effect of maternal obesity and fetal macrosomia on pregnancy outcomes. J Obstet Gynaecol Can 2014;36(9):776-84.

Ghosh SK, Raheja S, Tuli A, Raghunandan C, Agarwal S. Serum placental growth factor as a predictor of early onset preeclampsia in overweight/obese pregnant women. J Am Soc Hypertens 2013;7(2):137-48.

Gibson KS, Waters TP, Bailit JL. A risk of waiting: The weekly incidence of hypertensive disorders and associated maternal and neonatal morbidity in low-risk term pregnancies Presented in a poster presentation at the 34th annual meeting of the Society for Maternal-Fetal Medicine, Feb. 3-8, 2014, New Orleans, LA. Am J Obstet Gynecol 2016;214(3):389. [PMID: http://www.elsevier.com/inca/publications/store/6/2/3/2/7/7/index.htt]

Girsen AI, Osmundson SS, Naqvi M, Garabedian MJ, Lyell DJ. Body mass index and operative times at cesarean delivery. Obstet Gynecol 2014;124(4):684-9.

Goetzinger KR, Tuuli MG, Cahill AG, Macones GA, Odibo AO. Development and validation of a risk factor scoring system for first-trimester prediction of preeclampsia. Am J Perinatol 2014;31(12):1049-56.

Gonzalez I, Rubio MA, Cordido F, Breton I, Morales MJ, Vilarrasa N, et al. Maternal and perinatal outcomes after bariatric surgery: a Spanish multicenter study. Obes Surg 2015;25(3):436-42.

Gonzalez NLG, Goya M, Bellart J, Lopez J, Sancho MA, Mozas J, et al. Obstetric and perinatal outcome in women with twin pregnancy and gestational diabetes. J Matern -Fetal Neonatal Med 2012;25(7):1084-9.

Gould JB, Mayo J, Shaw GM, Stevenson DK, March of Dimes Prematurity Research Center at Stanford University School of Medicine. Swedish and American studies show that initiatives to decrease maternal obesity could play a key role in reducing preterm birth. Acta Paediatr 2014;103(6):586-91.

Graves E, Hill DJ Evers. The impact of abnormal glucose tolerance and obesity on fetal growth. #journal#. 2015. #volume#:#pages#

Graves E, Hill DJ, Evers S, Van Aarsen K, Yama B, Yuan S, et al. The impact of abnormal glucose tolerance and obesity on fetal growth. J Diabetes Res 2015;2015:847674

Green C, Shaker D. Impact of morbid obesity on the mode of delivery and obstetric outcome in nulliparous singleton pregnancy and the implications for rural maternity services. Aust N Z J Obstet Gynaecol 2011;51(2):172-4.

Guedes-Martins L, Carvalho M, Silva C, Cunha A, Saraiva J, Macedo F, et al. Relationship between body mass index and mean arterial pressure in normotensive and chronic hypertensive pregnant women: a prospective, longitudinal study. BMC Pregnancy Childbirth 2015;15:281

H. Kashyap,D. Sharma,A. Gala,O.T. Pratap,S. Murki. Effect of second trimester and third trimester weight gain on immediate outcomes in neonates born to mothers with gestational diabetes: a retrospective observational study from India. Journal of Maternal-Fetal and Neonatal Medicine. 2019///. 32:4133

Hahn KA, Hatch EE, Rothman KJ, Mikkelsen EM, Brogly SB, Sorensen HT, et al. Body size and risk of spontaneous abortion among danish pregnancy planners. Paediatr Perinat Epidemiol 2014;28(5):412-23.

Hameed AB, Lawton ES, McCain CL, Morton CH, Mitchell C, Main EK, et al. Pregnancy-related cardiovascular deaths in California: beyond peripartum cardiomyopathy. Am J Obstet Gynecol 2015;213(3):379-10.

Hancke K, Gundelach T, Hay B, Sander S, Reister F, Weiss JM. Pre-pregnancy obesity compromises obstetric and neonatal outcomes. J Perinat Med 2015;43(2):141-6.

Hantoushzadeh S, Sheikh M, Bosaghzadeh Z, Ghotbizadeh F, Tarafdari A, Panahi Z, et al. The impact of gestational weight gain in different trimesters of pregnancy on glucose challenge test and gestational diabetes. Postgrad Med J 2016;92(1091):520-4.

HAPO Study Cooperative Research Group. Hyperglycaemia and Adverse Pregnancy Outcome (HAPO) Study: associations with maternal body mass index. BJOG 2010;117(5):575-84.

Harper LM, Renth A, Cade WT, Colvin R, Macones GA, Cahill AG. Impact of obesity on maternal and neonatal outcomes in insulin-resistant pregnancy. Am J Perinatol 2014;31(5):383-8.

Hartge D, Spiegler J, Schroeer A, Deckwart V, Weichert J. Maternal super-obesity. Arch Gynecol Obstet 2016;293(5):987-92.

Hawley NL, Johnson W, Hart CN, Triche EW, Ah Ching J, Muasau-Howard B, et al. Gestational weight gain among American Samoan women and its impact on delivery and infant outcomes. BMC Pregnancy Childbirth 2015;15:10

Hayward CE, Cowley EJ, Mills TA, Sibley CP, Wareing M. Maternal obesity impairs specific regulatory pathways in human myometrial arteries. Biol Reprod 2014;90(3):65

Hazart, J., Le, Guennec D., Accoceberry, M., Lemery, D., Mulliez, A., Farigon, N., Lahaye, C., Miolanne-Debouit, M., and Boirie, Y.. Maternal Nutritional Deficiencies and Small-for-Gestational-Age Neonates at Birth of Women Who Have Undergone Bariatric Surgery. #journal#. 2017. 2017:#pages#

Herzog M, Cerar LK, Srsen TP, Verdenik I, Lucovnik M. Impact of risk factors other than prematurity on periventricular leukomalacia. A population-based matched case control study. Eur J Obstet Gynecol Reprod Biol 2015;187:57-9.

Hincz P, Borowski D, Krekora M, Podciechowski L, Horzelski W, Wilczynski J. Maternal obesity as a perinatal risk factor. Ginekol Pol 2009;80(5):334-7.

Hinkle SN, Sjaarda LA, Albert PS, Mendola P, Grantz KL. Comparison of methods for identifying small-for-gestational-age infants at risk of perinatal mortality among obese mothers: a hospital-based cohort study. BJOG Int J Obstet Gynaecol 2016;123(12):1983-8. [PMID: http://onlinelibrary.wiley.com/journal/10.1111/(ISSN)1471-0528]

Huvinen E, Grotenfelt NE, Eriksson JG, Rono K, Klemetti MM, Roine R, et al. Heterogeneity of maternal characteristics and impact on gestational diabetes (GDM) risk-Implications for universal GDM screening? Ann Med 2016;48(1-2):52-8.

Huy C, Loerbroks A, Hornemann A, Rohrig S, Schneider S. Prevalence, Trend and Determining Factors of Gestational Diabetes in Germany. Geburtshilfe Frauenheilkd 2012;72(4):311-5.

I. Melchor,J. Burgos,Campo A. Del,A. Aiartzaguena,J. Gutierrez,J.C. Melchor. Effect of maternal obesity on pregnancy outcomes in women delivering singleton babies: a historical cohort study. Journal of perinatal medicine. 2019/08/27/. 47:625

I.D. Ozodiegwu,H.M. Mamudu,L. Wang,R. Wallace,M. Quinn,Y. Liu,H.V. Doctor. Country-Level Analysis of the Association between Maternal Obesity and Neonatal Mortality in 34 Sub-Saharan African Countries. Annals of global health. 2019/12/06/. 85:139, 2019

I.S. Gomes-Filho,J.E.T. Batista,S.C. Trindade,J.S. Passos-Soares,E.M.M. Cerqueira,T.S.D. Costa,A.C.M.G. Figueiredo,M.D.C.N. Costa,L.F.F. Adan,G.S. Orrico,E.C.L. Porto,R.M.C. Pimenta,F.A. Scannapieco,P.M. Loomer,S.S.D. Cruz. Obesity and periodontitis are not associated in pregnant women. J Periodontal Res. 2020/01//. 55:77

Iyoke CA, Ugwu GO, Ezugwu FO, Lawani OL, Onyebuchi AK. Retrospective cohort study of the effects of obesity in early pregnancy on maternal weight gain and obstetric outcomes in an obstetric population in Africa. Int J Women Health 2013;5:501-7.

J. Lim,K. Han,S.Y. Kim,Y.H. Cho,Y.S. Yoon,H.S. Park,S.J. Yoo,K.K. Kim,Taskforce Team of the Obesity Fact Sheet of the Korean Society for the Study of Obesity. Effects of central obesity on maternal complications in Korean women of reproductive age. Obesity research & clinical practice. 2019/03//. 13:156

J. Lin,J. Huang,N. Wang,Y. Kuang,R. Cai. Effects of pre-pregnancy body mass index on pregnancy and perinatal outcomes in women with PCOS undergoing frozen embryo transfer. BMC pregnancy and childbirth. 2019/12/10/. 19:487, 2019

J. Mayrink,R.T. Souza,F.E. Feitosa,E.A. Rocha Filho,D.F. Leite,J. Vettorazzi,I.M. Calderon,M.H. Sousa,M.L. Costa,P.N. Baker,J.G. Cecatti,Preterm SAMBA study group. Incidence and risk factors for Preeclampsia in a cohort of healthy nulliparous pregnant women: a nested case-control study. Sci Rep. 2019/07/02/. 9:9517, 2019

J. McCoy,K.L. Downes,S.K. Srinivas,L.D. Levine. Postdates induction with an unfavorable cervix and risk of cesarean*. Journal of Maternal-Fetal and Neonatal Medicine. 2019///. 32:2874

J. Rao,D. Fan,S. Wu,D. Lin,H. Zhang,S. Ye,X. Luo,L. Wang,J. Yang,M. Pang,J. Zhang,Q. Xia,X. Yang,W. Wang,Y. Fu,Y. Liu,X. Guo,Z. Liu. Trend and risk factors of low birth weight and macrosomia in south China, 2005-2017: a retrospective observational study. Sci Rep. 2018///. 8:3393

J. Robertson,M. Lindgren,M. Schaufelberger,M. Adiels,L. Bjorck,C.E. Lundberg,N. Sattar,A. Rosengren,M. Aberg. Body Mass Index in Young Women and Risk of Cardiomyopathy: A Long-Term Follow-Up Study in Sweden. Circulation. 2020/02/18/. 141:520

J. Zhu,J. Zhang,M.J. Ng,B. Chern,G.S.H. Yeo,K.H. Tan. Angiogenic factors during pregnancy in Asian women with elevated blood pressure in early pregnancy and the risk of preeclampsia: A longitudinal cohort study. BMJ open. 2019///. 9:#pages#

J.G.B. Derraik,D. Pasupathy,L.M.E. McCowan,L. Poston,R.S. Taylor,N.A.B. Simpson,G.A. Dekker,J. Myers,M.C. Vieira,W.S. Cutfield,F. Ahlsson,SCOPE consortium. Paternal contributions to large-for-gestational-age term babies: findings from a multicenter prospective cohort study. Journal of developmental origins of health and disease. 2019/10//. 10:529

J.G.B. Derraik,S.E. Maessen,J.D. Gibbins,W.S. Cutfield,M. Lundgren,F. Ahlsson. Large-for-gestational-age phenotypes and obesity risk in adulthood: a study of 195,936 women. Sci Rep. 2020/02/07/. 10:2157, 2020

J.R. Corona-Rivera,F.J. Martinez-Macias,L. Bobadilla-Morales,A. Corona-Rivera,C. Pena-Padilla,I.M. Rios-Flores,P.A. Flores-Guevara,M. Orozco-Vela,C.I. Aranda-Sanchez,S.A. Brukman-Jimenez. Prevalence and risk factors for Down syndrome: A hospital-based single-center study in Western Mexico. American journal of medical genetics.Part A. 2019/03//. 179:435

J.R. Goodman,J.D. Peck,A. Landmann,M. Williams,A. Elimian. An evaluation of nutritional and vasoactive stimulants as risk factors for gastroschisis: a pilot study. Journal of Maternal-Fetal and Neonatal Medicine. 2019///. 32:2346

J.W. Dudenhausen,M. Kunze,U. Wittwer-Backofen,H.P. Hagenah,A. Strauss,V. Gunther,I. Alkatout,A. Grunebaum,M. Voigt. The relationship between maternal age, body mass index, and the rate of preterm birth. Journal of the Turkish German Gynecology Association. 2018///. 19:182

J.Y. Mei,A.L. Havard,A.J. Mularz,M.M. Maykin,S.L. Gaw. Impact of obesity class on trial of labor after cesarean success: does pre-pregnancy or at-delivery obesity status matter?. Journal of perinatology : official journal of the California Perinatal Association. 2019/08//. 39:1042

Jacob L, Kostev K, Kalder M. Risk of stillbirth in pregnant women with obesity in the United Kingdom. Obes Res Clin Pract 2016;10(5):574-9.

Janet M. Catov,Corette B. Parker,Bethany Barone Gibbs,Carla M. Bann,Benjamin Carper,Robert M. Silver,Hyagriv N. Simhan,Samuel Parry,Judith H. Chung,David M. Haas,Ronald J. Wapner,George R. Saade,Brian M. Mercer,C.Noel Bairey-Merz,Philip Greenland,Deborah B. Ehrenthal,Shannon E. Barnes,Anthony L. Shanks,Uma M. Reddy,William A. Grobman. Patterns of leisure-time physical activity across pregnancy and adverse pregnancy outcomes. The international journal of behavioral nutrition and physical activity. 2018///. 15:68

Jensen TB, Gerds TA, Gron R, Bretler DM, Schmiegelow MD, Andersson C, et al. Risk factors for venous thromboembolism during pregnancy. Pharmacoepidemiol Drug Saf 2013;22(12):1283-91.

Jiang, S., Chipps, D., Cheung, W. N., and Mongelli, M.. Comparison of adverse pregnancy outcomes based on the new IADPSG 2010 gestational diabetes criteria and maternal body mass index. #journal#. 2017. 57:#pages#

Johansson S, Villamor E, Altman M, Bonamy AKE, Granath F, Cnattingius S. Maternal overweight and obesity in early pregnancy and risk of infant mortality: a population based cohort study in Sweden. BMJ 2014;349:g6572

Jorgensen JS, Vinter CA, Lamont RF, Frederiksen-Moller B, Ronde Kristensen B, Mogensen O. First Nordic Conference on Obesity in Gynecology and Obstetrics (NOCOGO). Acta Obstet Gynecol Scand 2013;92(8):982-7.

Josefsson A, Bladh M, Wirehn A-B, Sydsjo G. Risk for congenital malformations in offspring of women who have undergone bariatric surgery. A national cohort. BJOG Int J Obstet Gynaecol 2013;120(12):1477-82.

Josefsson A, Blomberg M, Bladh M, Frederiksen SG, Sydsjo G. Bariatric surgery in a national cohort of women: Sociodemographics and obstetric outcomes. Am J Obstet Gynecol 2011;205(3):206

Joshi S, Unni J, Vijay S, Khanijo V, Gupte N, Divate U. Obesity and pregnancy outcome in a private tertiary hospital in India. Int J Gynaecol Obstet 2011;114(1):82-3.

Joy S, Istwan N, Rhea D, Desch C, Stanziano G. The impact of maternal obesity on the incidence of adverse pregnancy outcomes in high-risk term pregnancies. Am J Perinatol 2009;26(5):345-9.

Ju H, Chadha Y, Donovan T, O'Rourke P. Fetal macrosomia and pregnancy outcomes. Aust N Z J Obstet Gynaecol 2009;49(5):504-9.

K. Giannakou,E. Evangelou,P. Yiallouros,C.A. Christophi,N. Middleton,E. Papatheodorou,S.I. Papatheodorou. Risk factors for gestational diabetes: An umbrella review of meta-analyses of observational studies. PloS one. 2019///. 14:e0215372, 2019

K. Hidayat,S.Y. Zou,B.M. Shi. The influence of maternal body mass index, maternal diabetes mellitus, and maternal smoking during pregnancy on the risk of childhood-onset type 1 diabetes mellitus in the offspring: Systematic review and meta-analysis of observational studies. Obesity reviews : an official journal of the International Association for the Study of Obesity. 2019/08//. 20:1106

K. Hilden,U. Hanson,M. Persson,A. Magnuson,D. Simmons,H. Fadl. Gestational diabetes and adiposity are independent risk factors for perinatal outcomes: a population based cohort study in Sweden. Diabetic medicine : a journal of the British Diabetic Association. 2019/02//. 36:151

K. Ozgur,H. Bulut,M. Berkkanoglu,P. Humaidan,K. Coetzee. Increased body mass index associated with increased preterm delivery in frozen embryo transfers. Journal of obstetrics and gynaecology : the journal of the Institute of Obstetrics and Gynaecology. 2019/04//. 39:377

Kalk P, Guthmann F, Krause K, Relle K, Godes M, Gossing G, et al. Impact of maternal body mass index on neonatal outcome. Eur J Med Res 2009;14(5):216-22.

Kalyvas AV, Kalamatianos T, Pantazi M, Lianos GD, Stranjalis G, Alexiou GA. Maternal environmental risk factors for congenital hydrocephalus: a systematic review. Neurosurg focus 2016;41(5):E3

Kaplan-Sturk R, Akerud H, Volgsten H, Hellstrom-Westas L, Wiberg-Itzel E. Outcome of deliveries in healthy but obese women: obesity and delivery outcome. BMC Res Notes 2013;6:50

Kayem G, Kurinczuk J, Lewis G, Golightly S, Brocklehurst P, Knight M. Risk factors for progression from severe maternal morbidity to death: a national cohort study. PloS one 2011;6(12):e29077

Kazemian E, Sotoudeh G, Dorosty-Motlagh AR, Eshraghian MR, Bagheri M. Maternal obesity and energy intake as risk factors of pregnancy-induced hypertension among Iranian women. J Health Popul Nutr 2014;32(3):486-93.

Kennedy NJ, Peek MJ, Quinton AE, Lanzarone V, Martin A, Benzie R, et al. Maternal abdominal subcutaneous fat thickness as a predictor for adverse pregnancy outcome: a longitudinal cohort study. BJOG 2016;123(2):225-32.

Kerrigan AM, Kingdon C. Maternal obesity and pregnancy: a retrospective study. Midwifery 2010;26(1):138-46.

Khashan AS, Kenny LC. The effects of maternal body mass index on pregnancy outcome. Eur J Epidemiol 2009;24(11):697-705.

Khatibi A, Brantsaeter AL, Sengpiel V, Kacerovsky M, Magnus P, Morken NH, et al. Prepregnancy maternal body mass index and preterm delivery. Am J Obstet Gynecol 2012;207(3):212-7.

Khattabi Z. Filali,M. Biolcati,A. Fois,A. Chatrenet,D. Laroche,R. Attini,M.T. Cheve,G.B. Piccoli. Chronic kidney disease in preeclamptic patients: not found unless searched for-Is a nephrology evaluation useful after an episode of preeclampsia?. Journal of nephrology. 2019/12//. 32:977

Kichou B, Henine N, Kichou L, Benbouabdellah M. [Epidemiology of pre-eclampsia in Tizi-ouzou city (Algeria)]. Ann Cardiol Angeiol (Paris) 2015;64(3):164-8.

Kim SS, Zhu Y, Grantz KL, Hinkle SN, Chen Z, Wallace ME, et al. Obstetric and Neonatal Risks Among Obese Women Without Chronic Disease. Obstet Gynecol 2016;128(1):104-12.

Klemetti M, Nuutila M, Tikkanen M, Kari MA, Hiilesmaa V, Teramo K. Trends in maternal BMI, glycaemic control and perinatal outcome among type 1 diabetic pregnant women in 1989-2008. Diabetologia 2012;55(9):2327-34.

Knight M, Kurinczuk JJ, Spark P, Brocklehurst P, UK Obstetric SS. Extreme obesity in pregnancy in the United Kingdom. Obstet Gynecol 2010;115(5):989-97.

Knight-Agarwal CR, Williams LT, Davis D, Davey R, Cochrane T, Zhang H, et al. Association of BMI and interpregnancy BMI change with birth outcomes in an Australian obstetric population: a retrospective cohort study. BMJ open 2016;6(5):e010667

Koch L. Obesity: Effect of maternal obesity on neonatal outcomes. Nat Rev Endocrionol 2013;9(8):439

Korkmaz L, Bastug O, Kurtoglu S. Maternal Obesity and its Short- and Long-Term Maternal and Infantile Effects. J Clin Res Pediatr Endocrinol 2016;8(2):114-24.

Kosa JL, Guendelman S, Pearl M, Graham S, Abrams B, Kharrazi M. The association between pre-pregnancy BMI and preterm delivery in a diverse southern California population of working women. Matern Child Health J 2011;15(6):772-81.

Kosinska-Kaczynska K, Wielgos M. Do normal-weight women pregnant with twins are at the lowest risk of developing preeclampsia? J Matern -Fetal Neonatal Med 2017;30(2):191-3.

Kyvernitakis I, Kohler C, Schmidt S, Misselwitz B, Grossmann J, Hadji P, et al. Impact of maternal body mass index on the cesarean delivery rate in Germany from 1990 to 2012. J Perinat Med 2015;43(4):449-54. [PMID: http://www.degruyter.com/view/j/jpme]

L. Doi,A.J. Williams,L. Marryat,J. Frank. Cohort study of high maternal body mass index and the risk of adverse pregnancy and delivery outcomes in Scotland. BMJ open. 2020/02/20/. 10:e026168, 2020

L. Liu,Y. Ma,N. Wang,W. Lin,Y. Liu,D. Wen. Maternal body mass index and risk of neonatal adverse outcomes in China: a systematic review and meta-analysis. BMC pregnancy and childbirth. 2019/03/29/. 19:105, 2019

L.L. Albers,B. Greulich,P. Peralta. Body mass index, midwifery intrapartum care, and childbirth lacerations. Journal of Midwifery and Women's Health. 2006///. 51:249

Lapolla A, Marangon M, Dalfra MG, Segato G, De Luca M, Fedele D, et al. Pregnancy outcome in morbidly obese women before and after laparoscopic gastric banding. Obes Surg 2010;20(9):1251-7.

Leon-Garcia SM, Roeder HA, Nelson KK, Liao X, Pizzo DP, Laurent LC, et al. Maternal obesity and sex-specific differences in placental pathology. Placenta 2016;38:33-40. [PMID: http://www.elsevier.com/inca/publications/store/6/2/3/0/6/4/index.htt]

Lesko J, Peaceman A. Pregnancy outcomes in women after bariatric surgery compared with obese and morbidly obese controls. Obstet Gynecol 2012;119(3):547-54.

Leth RA, Uldbjerg N, Norgaard M, Moller JK, Thomsen RW. Obesity, diabetes, and the risk of infections diagnosed in hospital and post-discharge infections after cesarean section: a prospective cohort study. Acta Obstet Gynecol Scand 2011;90(5):501-9.

Leung C, Saaid R, Pedersen L, Park F, Poon L, Hyett J. Demographic factors that can be used to predict early-onset pre-eclampsia. J Matern Fetal Neonatal Med 2015;28(5):535-9.

Lindam A, Johansson S, Stephansson O, Wikstrom AK, Cnattingius S. High Maternal Body Mass Index in Early Pregnancy and Risks of Stillbirth and Infant Mortality-A Population-Based Sibling Study in Sweden. Am J Epidemiol 2016;184(2):98-105.

Liu P, Xu L, Wang Y, Zhang Y, Du Y, Sun Y, et al. Association between perinatal outcomes and maternal pre-pregnancy body mass index. Obes Rev 2016;17(11):1091-102.

Luealon P, Phupong V. Risk factors of preeclampsia in Thai women. J Med Assoc Thai 2010;93(6):661-6.

Lutsiv O, Mah J, Beyene J, McDonald SD. The effects of morbid obesity on maternal and neonatal health outcomes: a systematic review and meta-analyses. Obes Rev 2015;16(7):531-46.

M. Kianpour,A. Aminorroaya,M. Amini,A. Feizi,M. Janghorbani. Thyroid function test reference ranges in the first trimester of gestation and pregnancy outcomes: Protocol and preliminary results for cohort population-based study Isfahan, Iran. Journal of Research in Medical Sciences. 2018///. 23:#pages#

M. Lahti-Pulkkinen,S. Bhattacharya,S.H. Wild,R.S. Lindsay,K. Raikkonen,J.E. Norman,S. Bhattacharya,R.M. Reynolds. Consequences of being overweight or obese during pregnancy on diabetes in the offspring: a record linkage study in Aberdeen, Scotland. Diabetologia. 2019/08//. 62:1412

M. Mokhtari,K. Nasri,F. Tara,E. Zarean,S. Hantoushzadeh,M. Radmehr,M. Kashanian. A Survey of Venous Thromboembolism (VTE) Prophylaxis in Obstetrics Patients in Iran. J Family Reprod Health. 2019/03//. 13:21

M. Moreau,M. Remy,S. Nusinovici,V. Rouger,L. Molines,C. Flamant,G. Legendre,J.C. Roze,A. Salle,Bogaert P. Van,R. Coutant,G. Gascoin. Neonatal and neurodevelopmental outcomes in preterm infants according to maternal body mass index: A prospective cohort study. PloS one. 2019///. 14:e0225027, 2019

M. Simko,A. Totka,D. Vondrova,M. Samohyl,J. Jurkovicova,M. Trnka,A. Cibulkova,J. Stofko,L. Argalasova. Maternal Body Mass Index and Gestational Weight Gain and Their Association with

Pregnancy Complications and Perinatal Conditions. International journal of environmental research and public health. 2019/05/17/. 16:#pages#

M.-Y. Li,S. Rawal,S.N. Hinkle,Y.-Y. Zhu,F. Tekola-Ayele,M.Y. Tsai,S.-M. Liu,C.-L. Zhang. Sex Hormone-binding Globulin, Cardiometabolic Biomarkers, and Gestational Diabetes: A Longitudinal Study and Meta-Analysis. Maternal-Fetal Medicine. 2020///. 2:2

M.D. Merc,M. Lucovnik,A.T. Bregar,I. Verdenik,N. Tul,I. Blickstein. Stillbirths in women with pre-gravid obesity. Journal of perinatal medicine. 2019///. 47:319

M.K. Maducolil,S. Al-Obaidly,T. Olukade,H. Salama,M. AlQubaisi,Rifai H. Al. Maternal characteristics and pregnancy outcomes of women with chronic hypertension: a population-based study. Journal of perinatal medicine. 2020/02/25/. 48:139

M.S. Harrison,A.P. Betran,J.P. Vogel,R.L. Goldenberg,A.M. Gulmezoglu. Mode of delivery among nulliparous women with single, cephalic, term pregnancies: The WHO global survey on maternal and perinatal health, 2004-2008. International journal of gynaecology and obstetrics: the official organ of the International Federation of Gynaecology and Obstetrics. 2019/11//. 147:165

Maayan-Metzger A, Schushan-Eisen I, Strauss T, Globus O, Leibovitch L. Gestational weight gain and body mass indexes have an impact on the outcomes of diabetic mothers and infants. Acta Paediatr 2015;104(11):1150-5.

Madan J, Chen M, Goodman E, Davis J, Allan W, Dammann O. Maternal obesity, gestational hypertension, and preterm delivery. J Matern Fetal Neonatal Med 2010;23(1):82-8.

Magann EF, Doherty DA, Sandlin AT, Chauhan SP, Morrison JC. The effects of an increasing gradient of maternal obesity on pregnancy outcomes. Aust N Z J Obstet Gynaecol 2013;53(3):250-7.

Magriples U, Boynton MH, Kershaw TS, Duffany KO, Rising SS, Ickovics JR. Blood pressure changes during pregnancy: impact of race, body mass index, and weight gain. Am J Perinatol 2013;30(5):415-24.

Mandal D, Manda S, Rakshi A, Dey RP, Biswas SC, Banerjee A. Maternal obesity and pregnancy outcome: a prospective analysis. J Assoc Physicians India 2011;59:486-9.

Mantakas A, Farrell T. The influence of increasing BMI in nulliparous women on pregnancy outcome. Eur J Obstet Gynecol Reprod Biol 2010;153(1):43-6.

Marchi J, Berg M, Dencker A, Olander EK, Begley C. Risks associated with obesity in pregnancy, for the mother and baby: a systematic review of reviews. Obes Rev 2015;16(8):621-38.

Marengo L, Farag NH, Canfield M. Body mass index and birth defects: Texas, 2005-2008. Matern Child Health J 2013;17(10):1898-907.

Marshall NE, Guild C, Cheng YW, Caughey AB, Halloran DR. The effect of maternal body mass index on perinatal outcomes in women with diabetes. Am J Perinatol 2014;31(3):249-56.

Mathur P, Maru L, Dave A. A Prospective Study of Placental Growth Factor Assay as a Novel Biomarker in Predicting Early-Onset Preeclampsia in High-Risk Women. J Obstet Gynecol India 2016;66:98-103. [PMID: http://medind.nic.in/jaq/jaqm.shtml]

Mbah AK, Kornosky JL, Kristensen S, August EM, Alio AP, Marty PJ, et al. Super-obesity and risk for early and late pre-eclampsia. BJOG 2010;117(8):997-1004.

McDonald SD, Han Z, Mulla S, Beyene J, Knowledge Synthesis Group. Overweight and obesity in mothers and risk of preterm birth and low birth weight infants: systematic review and meta-analyses. BMJ 2010;341:c3428

McIntyre HD, Gibbons KS, Flenady VJ, Callaway LK. Overweight and obesity in Australian mothers: epidemic or endemic? Med J Aust 2012;196(3):184-8.

Meehan S, Beck CR, Mair-Jenkins J, Leonardi-Bee J, Puleston R. Maternal obesity and infant mortality: a meta-analysis. Pediatrics 2014;133(5):863-71.

Meenakshi, Srivastava R, Sharma NR, Kushwaha KP, Aditya V. Obstetric behavior and pregnancy outcome in overweight and obese women: maternal and fetal complications and risks in relation to maternal overweight and obesity. J Obstet Gynaecol India 2012;62(3):276-80.

Meher UN, Aslam M, Ahmed SR, Rajab M, Kattea L. Impact of obesity on fetomaternal outcome in pregnant saudi females. Int J Health Sci (Qassim) 2009;3(2):187-95.

Mehrabi E, Kamalifard M, Yavarikia P, Ebrahimi Mameghani M. The Relation between Early Pregnancy Anthropometric Indices among Primiparous Women and Macrosomia. J Caring Sci 2012;1(3):153-8.

Mehta SH, Kerver JM, Sokol RJ, Keating DP, Paneth N. The association between maternal obesity and neurodevelopmental outcomes of offspring. J Pediatr 2014;165(5):891-6.

Mhaske N, Agarwal R, Wadhwa RD, Basannar DR. Study of the Risk Factors for Cesarean Delivery in Induced Labors at Term. J Obstet Gynaecol India 2015;65(4):236-40.

Minsart AF, N'guyen TS, Dimtsu H, Ratsimanresy R, Dada F, Ali Hadji R. Maternal obesity and rate of cesarean delivery in Djibouti. Int J Gynaecol Obstet 2014;127(2):167-70.

Mochhoury L, Razine R, Kasouati J, Kabiri M, Barkat A. Body mass index, gestational weight gain, and obstetric complications in Moroccan population. J Pregnancy 2013;2013:379461

Monari F, Pedrielli G, Vergani P, Pozzi E, Mecacci F, Serena C, et al. Adverse Perinatal Outcome in Subsequent Pregnancy after Stillbirth by Placental Vascular Disorders. PloS one 2016;11(5):e0155761

Moore Simas TA, Crawford SL, Bathgate S, Yan J, Robidoux L, Moore M, et al. Angiogenic biomarkers for prediction of early preeclampsia onset in high-risk women. J Matern Fetal Neonatal Med 2014;27(10):1038-48.

Morgan ES, Wilson E, Melody T, Parmar K, Zhang Y, Gao F, et al. An observational study of haemostatic changes, leptin and soluble endoglin during pregnancy in women with different BMIs. Blood Coagul Fibrinolysis 2016;

Morken NH, Klungsoyr K, Magnus P, Skjaerven R. Pre-pregnant body mass index, gestational weight gain and the risk of operative delivery. Acta Obstet Gynecol Scand 2013;92(7):809-15.

Mourad M, Silverstein M, Bender S, Melka S, Klauser CK, Gupta S, et al. The effect of maternal obesity on outcomes in women undergoing tertiary or higher cesarean delivery. J Matern Fetal Neonatal Med 2015;28(9):989-93.

Muto H, Yamamoto R, Ishii K, Kakubari R, Takaoka S, Mabuchi A, et al. Risk assessment of hypertensive disorders in pregnancy with maternal characteristics in early gestation: A single-center cohort study. Taiwan j obstet gynecol 2016;55(3):341-5.

N. Hikita,M. Haruna,M. Matsuzaki,E. Sasagawa,M. Murata,A. Yura,O. Oidovsuren. Is High Maternal Body Mass Index Associated with Cesarean Section Delivery in Mongolia? A Prospective Observational Study. #journal#. 2019///. 4:128

N. Jancar,Ponikvar B. Mihevc,S. Tomsic,Bokal E. Vrtacnik,S. Korosec. Is IVF/ICSI [corrected] an Independent Risk Factor for Spontaneous Preterm Birth in Singletons? A Population-Based Cohort Study. BioMed research international. 2018///. 2018:7124362, 2018.:#pages#

N. Pritchard,A. Lindquist,I.D.A. Siqueira,S.P. Walker,M. Permezel. INTERGROWTH-21st compared with GROW customized centiles in the detection of adverse perinatal outcomes at term. Journal of Maternal-Fetal and Neonatal Medicine. 2020///. 33:961

N. Rodriguez-Mesa,P. Robles-Benayas,Y. Rodriguez-Lopez,E.M. Perez-Fernandez,A.I. Cobo-Cuenca. Influence of Body Mass Index on Gestation and Delivery in Nulliparous Women: A Cohort Study. International journal of environmental research and public health. 2019/06/06/. 16:#pages#

N.D. Pace,A.M. Siega-Riz,A.F. Olshan,N.C. Chescheir,S.R. Cole,T.A. Desrosiers,S.C. Tinker,A.T. Hoyt,M.A. Canfield,S.L. Carmichael,R.E. Meyer,National Birth Defects Prevention Study. Survival of infants with spina bifida and the role of maternal prepregnancy body mass index. Birth defects research.Part A, Clinical and molecular teratology. 2019/10/01/. 111:1205

Nani FS, Torres MLA. Correlation between the body mass index (BMI) of pregnant women and the development of hypotension after spinal anesthesia for cesarean section. Rev Bras Anestesiol 2011;61(1):21-30.

Narchi H, Skinner A. Overweight and obesity in pregnancy do not adversely affect neonatal outcomes: new evidence. J Obstet Gynaecol 2010;30(7):679-86.

Ngoga E, Hall D, Mattheyse F, Grove D. Outcome of pregnancy in the morbidly obese woman. S Afr Fam Pract 2009;51(1):39-41. [PMID: http://www.safpj.co.za/index.php/safpj/article/view/989/1403]

Nohr EA, Timpson NJ, Andersen CS, Davey Smith G, Olsen J, Sorensen TIA. Severe obesity in young women and reproductive health: the Danish National Birth Cohort. PloS one 2009;4(12):e8444

North RA, McCowan LM Dekker GA Poston. Clinical risk prediction for pre-eclampsia in nulliparous women: development of model in international prospective cohort. #journal#. 2011. 342:#pages#

O. Nkoka,P.A.M. Ntenda,T. Senghore,P. Bass. Maternal overweight and obesity and the risk of caesarean birth in Malawi. Reproductive health. 2019/04/03/. 16:40, 2019

Oddy WH, de Klerk NH, Miller M, Payne J, Bower C. Association of maternal pre-pregnancy weight with birth defects: evidence from a case-control study in Western Australia. Aust N Z J Obstet Gynaecol 2009;49(1):11-5.

Ogonowski J, Miazgowski T, Kuczynska M, Krzyzanowska-Swiniarska B, Celewicz Z. Pregravid body mass index as a predictor of gestational diabetes mellitus. Diabet Med 2009;26(4):334-8.

Okafor UV, Efetie ER, Nwoke O, Okezie O, Umeh U. Anaesthetic and obstetric challenges of morbid obesity in caesarean deliveries--a study in South-eastern Nigeria. Afr Health Sci 2012;12(1):54-7.

Onubi OJ, Marais D, Aucott L, Okonofua F, Poobalan AS. Maternal obesity in Africa: a systematic review and meta-analysis. J Public Health (Oxf) 2016;38(3):e218-e231

Ornaghi S, Tyurmorezova A, Algeri P, Giardini V, Ceruti P, Vertemati E, et al. Influencing factors for late-onset preeclampsia. J Matern Fetal Neonatal Med 2013;26(13):1299-302.

Ovesen P, Rasmussen S, Kesmodel U. Effect of prepregnancy maternal overweight and obesity on pregnancy outcome. Obstet Gynecol 2011;118(2 Pt 1):305-12.

Owiredu WKBA, Ahenkorah L, Turpin CA, Amidu N, Laing EF. Putative risk factors of pregnancy-induced hypertension among ghanaian pregnant women. J Med Biomed Sci 2012;1(3):62-76. [PMID: http://www.ajol.info/index.php/jmbs/article/download/80233/70491]

P. Alves,M.F. Malheiro,J.C. Gomes,T. Ferraz,N. Montenegro. Riscos da obesidade materna na gravidez: um estudo caso-controle em uma oopulacao obstetrica portuguesa, Risks of Maternal Obesity in Pregnancy: A Case-control Study in a Portuguese Obstetrical Population. Revista brasileira de ginecologia e obstetricia : revista da Federacao Brasileira das Sociedades de Ginecologia e Obstetricia. 2019///. 41:682

P. Alves,M.F. Malheiro,J.C. Gomes,T. Ferraz,N. Montenegro. Risks of Maternal Obesity in Pregnancy: A Case-control Study in a Portuguese Obstetrical Population. Revista Brasileira de Ginecologia e Obstetricia. 2019/12//. 41:682

Pallasmaa N, Ekblad U, Gissler M, Alanen A. The impact of maternal obesity, age, pre-eclampsia and insulin dependent diabetes on severe maternal morbidity by mode of delivery-a register-based cohort study. Arch Gynecol Obstet 2015;291(2):311-8.

Papachatzi E, Papadopoulos V, Dimitriou G, Paparrodopoulos S, Papadimitriou-Olivgeris M, Vantarakis A. Prepregnancy maternal obesity and fetal-perinatal death in a Mediterranean country. J Perinat Med 2015;43(3):291-8.

Papachatzi E, Paparrodopoulos S, Papadopoulos V, Dimitriou G, Vantarakis A. Pre-pregnancy maternal obesity in Greece: A case-control analysis. Early Hum Dev 2016;93:57-61.

Papoutsis D, Antonakou A, Gornall A, Tzavara C, Mohajer M. The SaTH risk-assessment tool for the prediction of emergency cesarean section in women having induction of labor for all indications: a large-cohort based study. Arch Gynecol Obstet 2016;

Pare E, Parry S, Mcelrath TF, Pucci D, Newton A, Lim KH. Clinical risk factors for preeclampsia in the 21st century. Obstet Gynecol 2014;124(4):763-70.

Parker MG, Ouyang F, Pearson C, Gillman MW, Belfort MB, Hong X, et al. Prepregnancy body mass index and risk of preterm birth: association heterogeneity by preterm subgroups. BMC Pregnancy Childbirth 2014;14:153

Penn N, Oteng-Ntim E, Oakley LL, Doyle P. Ethnic variation in stillbirth risk and the role of maternal obesity: analysis of routine data from a London maternity unit. BMC Pregnancy Childbirth 2014;14:404

Persson M, Cnattingius S, Wikstrom AK, Johansson S. Maternal overweight and obesity and risk of pre-eclampsia in women with type 1 diabetes or type 2 diabetes. Diabetologia 2016;59(10):2099-105.

Pevzner, L.. Factors predicting successful labor induction with dinoprostone and misoprostol vaginal inserts. #journal#. 2009. 114:#pages#

Pirjani R, Shirzad N, Qorbani M, Phelpheli M, Nasli-Esfahani E, Bandarian F, et al. Gestational diabetes mellitus its association with obesity: a prospective cohort study. Eat Weight Disord 2016;

Popova PV, Grineva EN, Gerasimov AS, Kravchuk EN, Ryazantseva EM, Shelepova ES. The new combination of risk factors determining a high risk of gestational diabetes mellitus. Minerva Endocrinol 2015;40(4):239-47.

Poston L, Caleyachetty R, Cnattingius S, Corvalan C, Uauy R, Herring S, et al. Preconceptional and maternal obesity: epidemiology and health consequences. Lancet Diabetes Endocrinol 2016;4(12):1025-36.

Pugh SK, Doherty DA, Magann EF, Chauhan SP, Hill JB, Morrison JC. Does hypoglycemia following a glucose challenge test identify a high risk pregnancy? Reprod Health 2009;6:10

R. Buffarini,A.J.D. Barros,A. Matijasevich,Mola C. Loret de,I.S. Santos. Gestational diabetes mellitus, pre-gestational BMI and offspring BMI z-score during infancy and childhood: 2004 Pelotas Birth Cohort. BMJ open. 2019/07/09/. 9:e024734, 2019

R. Granese,E. Gitto,G. D'Angelo,R. Falsaperla,G. Corsello,D. Amadore,G. Calagna,I. Fazzolari,R. Grasso,O. Triolo. Preterm birth: seven-year retrospective study in a single centre population. Italian journal of pediatrics. 2019/04/11/. 45:45

R. Olmedo-Requena,J. Gomez-Fernandez,C. Amezcua-Prieto,J. Mozas-Moreno,K.S. Khan,J.J. Jimenez-Moleon. Pre-Pregnancy Adherence to the Mediterranean Diet and Gestational Diabetes Mellitus: A Case-Control Study. Nutrients. 2019/05/01/. 11:#pages#

R.A. Brown,H. Dakkak,J. Gilliland,J.A. Seabrook. Predictors of drug use during pregnancy: The relative effects of socioeconomic, demographic, and mental health risk factors. Journal of neonatal-perinatal medicine. 2019///. 12:179

R.A. Tinius,J.D. Lopez,W.T. Cade,R.I. Stein,D. Haire-Joshu,A.G. Cahill. Patient and obstetric provider communication regarding weight gain management among socioeconomically disadvantaged African American women who are overweight/obese. Women and Health. 2020/02//. 60:156

R.F. Zhao,L. Zhou,W.Y. Zhang. Identifying appropriate pre-pregnancy body mass index classification to improve pregnancy outcomes in women of childbearing age in Beijing, China: a retrospective cohort study. Asia Pacific journal of clinical nutrition. 2019///. 28:567

R.H. Jessel,F.J. Rosario,Y.Y. Chen,K. Erickson,S.B. Teal,A. Kramer,E. Cotton,S. Ryan,T. Jansson,T.L. Powell. Decreased placental folate transporter expression and activity in first and second trimester in obese mothers. J nutr biochem. 2020/03//. 77:108305, 2020 Mar.:#pages#

Radulescu L, Munteanu O, Popa F, Cirstoiu M. The implications and consequences of maternal obesity on fetal intrauterine growth restriction. J med life 2013;6(3):292-8.

Raja UA, Mcaree T, Bassett P, Sharma S. The implications of a raised maternal BMI: a DGH experience. J Obstet Gynaecol 2012;32(3):247-51.

Rajasingam D, Seed PT, Briley AL, Shennan AH, Poston L. A prospective study of pregnancy outcome and biomarkers of oxidative stress in nulliparous obese women. Am J Obstet Gynecol 2009;200(4):395-9.

Rankin J, Tennant PWG, Stothard KJ, Bythell M, Summerbell CD, Bell R. Maternal body mass index and congenital anomaly risk: a cohort study. Int J Obes (Lond) 2010;34(9):1371-80.

Rao DP, Rao VA. Morbidly obese parturient: Challenges for the anaesthesiologist, including managing the diffifi cult airway in obstetrics. What is new? Indian J Anaesth 2010;54(6):508-21. [PMID: http://medind.nic.in/iad/t10/i6/iadt10i6p508.pdf]

Rayis DA, Abbaker AO, Salih Y, Adam I. Obesity and pregnancy outcome in Khartoum, Sudan. Int J Gynaecol Obstet 2011;113(2):160-1.

Reeves IV, Bamji ZD, Rosario GB, Lewis KM, Young MA, Washington KN. Vitamin D deficiency in pregnant women of ethnic minority: a potential contributor to preeclampsia. J Perinatol 2014;34(10):767-73.

Reyes LM, Garcia RG, Ruiz SL, Camacho PA, Ospina MB, Aroca G, et al. Risk factors for preeclampsia in women from Colombia: a case-control study. PloS one 2012;7(7):e41622

Richardson C, Trotman H. Risk factors for the delivery of macrosomic infants at the University Hospital of the West Indies. Am J Perinatol 2014;31(11):927-32.

Robinson CJ, Hill EG, Alanis MC, Chang EY, Johnson DD, Almeida JS. Examining the effect of maternal obesity on outcome of labor induction in women with preeclampsia. HYPERTENS PREGNANCY 2010;29(4):446-56.

Rockhill K, Dorfman H, Srinath M, Hogue C. The Effects of Prepregnancy Body Mass Index and Gestational Weight Gain on Fetal Macrosomia Among American Indian/Alaska Native Women. Matern Child Health J 2015;19(11):2480-91.

Roman AS, Rebarber A, Fox NS, Klauser CK, Istwan N, Rhea D, et al. The effect of maternal obesity on pregnancy outcomes in women with gestational diabetes. J Matern Fetal Neonatal Med 2011;24(5):723-7.

Rowan JA, Gao W.. Glycemia and its relationship to outcomes in the metformin in gestational diabetes trial. #journal#. 2010. 33:#pages#

Rudman S. Sabolovic,I. Djakovic,V. Gall,Z. Djakovic,V. Kosec. Pregnancy outcome in gestational diabetes compared to body mass index. Acta clinica Croatica. 2019/03//. 58:37

Ruhstaller KE, Bastek JA, Thomas A, Mcelrath TF, Parry SI, Durnwald CP. The Effect of Early Excessive Weight Gain on the Development of Hypertension in Pregnancy. Am J Perinatol 2016;33(12):1205-10.

Ruifrok AE, Althuizen E Oostdam. The relationship of objectively measured physical activity and sedentary behaviour with gestational weight gain and birth weight. #journal#. 2014. 2014:#pages#

Russell Z, Salihu HM, Lynch O'N, Alio AP, Belogolovkin V. The association of prepregnancy body mass index with pregnancy outcomes in triplet gestations. Am J Perinatol 2010;27(1):41-6.

S. Christinajoice,S. Misra,S. Bhattacharya,S.S. Kumar,B.D. Nandhini,C. Palanivelu,P.P. Raj. Impact of Bariatric Surgery on Female Reproductive Health and Maternal Outcomes. Obesity surgery. 2020/02//. 30:383

S. Soomro,R. Kumar,H. Lakhan,F. Shaukat. Risk Factors for Pre-eclampsia and Eclampsia Disorders in Tertiary Care Center in Sukkur, Pakistan. Cureus. 2019/11/10/. 11:e6115, 2019

S. Vonck,D. Lanssens,A.S. Staelens,K. Tomsin,J. Oben,L. Bruckers,W. Gyselaers. Obesity in pregnancy causes a volume overload in third trimester. European journal of clinical investigation. 2019/11//. 49:e13173, 2019

S.-S. Hashemi-Nazari,F. Najafi,M.-A. Rahimi,N. Izadi,F. Heydarpour,H. Forooghirad. Estimation of gestational diabetes mellitus and dose-response association of BMI with the occurrence of diabetes mellitus in pregnant women of the west of Iran. Health care for women international. 2020///. 41:121

S.E. Starnes,F. Nardi,P. Fitchev,B.A. Plunkett,C. Thorpe,C.H. Wang,C. Vogler,S.E. Crawford. Influence of maternal obesity and metabolic and vascular mediators in twin-twin transfusion syndrome. Reprod Biol. 2019/06//. 19:165

S.J. McCall,Z. Li,J.J. Kurinczuk,E. Sullivan,M. Knight. Maternal and perinatal outcomes in pregnant women with BMI >50: An international collaborative study. PloS one. 2019///. 14:e0211278, 2019

S.N. Smith,L. Thorp,E. Karreman,A. Adanlawo. Review of Stillbirth in a Canadian Tertiary Care Centre. Journal of obstetrics and gynaecology Canada : JOGC = Journal d'obstetrique et gynecologie du Canada : JOGC. 2020/02//. 42:126

S.R.C. Madi,R.M.R. Garcia,V.C. Souza,R.L. Rombaldi,B.F. Araujo,J.M. Madi. Efeito da obesidade sobre os resultados gestacionais e perinatais, Effect of Obesity on Gestational and Perinatal Outcomes. Revista brasileira de ginecologia e obstetricia : revista da Federacao Brasileira das Sociedades de Ginecologia e Obstetricia. 2017///. 39:330

S.X. Chen,K.M. Rasmussen,J. Finkelstein,H. Stovring,E.A. Nohr,H. Kirkegaard. Maternal reproductive history and premenopausal risk of hypertension and cardiovascular disease: a Danish cohort study. BMJ open. 2019/11/04/. 9:e030702, 2019

Saereeporncharenkul K. Correlation of BMI to pregnancy outcomes in Thai women delivered in Rajavithi Hospital. J Med Assoc Thai 2011;94 Suppl 2:S52-S58

Salihu HM, August EM, de la Cruz C, Mogos MF, Weldeselasse H, Alio AP. Infant mortality and the risk of small size for gestational age in the subsequent pregnancy: a retrospective cohort study. Matern Child Health J 2013;17(6):1044-51.

Salihu HM, Luke S, Alio AP, Deutsch A, Marty PJ. The impact of obesity on spontaneous and medically indicated preterm birth among adolescent mothers. Arch Gynecol Obstet 2010;282(2):127-34.

Salihu HM, Lynch O, Alio AP, Kornosky JL, Clayton HB, Mbah AK. Extreme obesity and risk of placental abruption. Hum Reprod 2009;24(2):438-44.

Salles GF, Schlussel MM, Farias DR, Franco-Sena AB, Rebelo F, Lacerda EMA, et al. Blood pressure in healthy pregnancy and factors associated with no mid-trimester blood pressure drop: A prospective cohort study. Am J Hypertens 2015;28(5):680-9. [PMID: http://ajh.oxfordjournals.org/?371http://ajh.oxfordjournals.org/?371]

Samonte VI, Ngalob QG, Mata GD, Aherrera JA, Reyes E, Punzalan FE. Clinical and echocardiographic profile and outcomes of peripartum cardiomyopathy: the Philippine General Hospital experience. Heart Asia 2013;5(1):245-9.

Schneider S, Hoeft B, Freerksen N, Fischer B, Roehrig S, Yamamoto S, et al. Neonatal complications and risk factors among women with gestational diabetes mellitus. Acta Obstet Gynecol Scand 2011;90(3):231-7.

Schrauwers C, Dekker G. Maternal and perinatal outcome in obese pregnant women. J Matern Fetal Neonatal Med 2009;22(3):218-26.

Schummers L, Hutcheon JA, Bodnar LM, Lieberman E, Himes KP. Risk of adverse pregnancy outcomes by prepregnancy body mass index: a population-based study to inform prepregnancy weight loss counseling. Obstet Gynecol 2015;125(1):133-43.

Scott-Pillai R, Spence D, Cardwell CR, Hunter A, Holmes VA. The impact of body mass index on maternal and neonatal outcomes: a retrospective study in a UK obstetric population, 2004-2011. BJOG 2013;120(8):932-9.

Sebastian Manzanares G, Angel Santalla H, Irene Vico Z, Lopez Criado MS, Alicia Pineda L, Jose Luis Gallo V. Abnormal maternal body mass index and obstetric and neonatal outcome. J Matern Fetal Neonatal Med 2012;25(3):308-12.

Sekhavat L, Fallah R. Could maternal pre-pregnancy body mass index affect Apgar score? Arch Gynecol Obstet 2013;287(1):15-8.

Shahbazian H, Nouhjah S, Shahbazian N, Jahanfar S, Latifi SM, Aleali A, et al. Gestational diabetes mellitus in an Iranian pregnant population using IADPSG criteria: Incidence, contributing factors and outcomes. Diabetes Metab Syndr 2016;10(4):242-6.

Shai D, Shoham-Vardi I, Amsalem D, Silverberg D, Levi I, Sheiner E. Pregnancy outcome of women following bariatric surgery as compared with obese women: a population-based study. J Matern Fetal Neonatal Med 2014;27(3):275-8.

Sharashova EE, Anda EE, Grjibovski AM. Early pregnancy body mass index and spontaneous preterm birth in Northwest Russia: a registry-based study. BMC Pregnancy Childbirth 2014;14:303

Shaw GM, Wise PH, Mayo J, Carmichael SL, Ley C, Lyell DJ, et al. Maternal prepregnancy body mass index and risk of spontaneous preterm birth. Paediatr Perinat Epidemiol 2014;28(4):302-11.

Sheiner E, Edri A, Balaban E, Levi I, Aricha-Tamir B. Pregnancy outcome of women who conceive during or after the first year following bariatric surgery. Am J Obstet Gynecol 2011;204(1):50-6.

Shin D, Song WO. Prepregnancy body mass index is an independent risk factor for gestational hypertension, gestational diabetes, preterm labor, and small- and large-for-gestational-age infants. J Matern Fetal Neonatal Med 2015;28(14):1679-86.

Singh J, Huang CC, Driggers RW, Timofeev J, Amini D, Landy HJ, et al. The impact of pre-pregnancy body mass index on the risk of gestational diabetes. J Matern Fetal Neonatal Med 2012;25(1):5-10.

Sit D, Luther J, Dills JLJ, Eng H, Wisniewski S, Wisner KL. Abnormal screening for gestational diabetes, maternal mood disorder, and preterm birth. Bipolar Disord 2014;16(3):308-17.

Smith DM, Whitworth M, Sibley C, Taylor W, Gething J, Chmiel C, et al. The design of a community lifestyle programme to improve the physical and psychological well-being of pregnant women with a BMI of 30 kg/m2 or more. BMC public health 2010;10:284

Sohlberg S, Stephansson O, Cnattingius S, Wikstrom AK. Maternal body mass index, height, and risks of preeclampsia. Am J Hypertens 2012;25(1):120-5.

Song Y, Gao J, Qu Y, Wang S, Wang X, Liu J. Serum levels of leptin, adiponectin and resistin in relation to clinical characteristics in normal pregnancy and preeclampsia. Clin Chim Acta 2016;458:133-7. [PMID: http://www.elsevier.com/locate/clinchim]

Sorbye LM, Klungsoyr K, Samdal O, Owe KM, Morken NH. Pre-pregnant body mass index and recreational physical activity: effects on perinatal mortality in a prospective pregnancy cohort. BJOG 2015;122(10):1322-30.

Spain JE, Tuuli MG, Macones GA, Roehl KA, Odibo AO, Cahill AG. Risk factors for serious morbidity in term nonanomalous neonates. Am J Obstet Gynecol 2015;212(6):799-7.

Stacey T, Prady S, Haith-Cooper M, Downe S, Simpson N, Pickett K. Ethno-Specific Risk Factors for Adverse Pregnancy Outcomes: Findings from the Born in Bradford Cohort Study. Matern Child Health J 2016;20(7):1394-404.

Stacey T, Thompson JMD, Mitchell EA, Ekeroma AJ, Zuccollo JM, McCowan LME. Relationship between obesity, ethnicity and risk of late stillbirth: a case control study. BMC Pregnancy Childbirth 2011;11:3

Stillbirth Collaborative Research Network Writing Group. Association between stillbirth and risk factors known at pregnancy confirmation. JAMA 2011;306(22):2469-79.

Stuber TN, Kunzel EC, Zollner U, Rehn M, Wockel A, Honig A. Prevalence and Associated Risk Factors for Obesity During Pregnancy Over Time. Geburtshilfe Frauenheilkd 2015;75(9):923-8.

Stuebe AM, Landon MB, Lai Y, Spong CY, Carpenter MW, Ramin SM, et al. Maternal BMI, glucose tolerance, and adverse pregnancy outcomes. Am J Obstet Gynecol 2012;207(1):62-7.

Subramaniam A, Jauk VC, Goss AR, Alvarez MD, Reese C, Edwards RK. Mode of delivery in women with class III obesity: planned cesarean compared with induction of labor. Am J Obstet Gynecol 2014;211(6):700-9.

Sugiyama T, Nagao K, Metoki H, Nishigori H, Saito M, Tokunaga H, et al. Pregnancy outcomes of gestational diabetes mellitus according to pre-gestational BMI in a retrospective multi-institutional study in Japan. Endocr J 2014;61(4):373-80.

Suidan RS, Apuzzio JJ, Williams SF. Obesity, comorbidities, and the cesarean delivery rate. Am J Perinatol 2012;29(8):623-7.

Suk D, Kwak T, Khawar N, Vanhorn S, Salafia CM, Gudavalli MB, et al. Increasing maternal body mass index during pregnancy increases neonatal intensive care unit admission in near and full-term infants. J Matern -Fetal Neonatal Med 2016;29(20):3249-53.

Sullivan EA, Dickinson JE, Vaughan GA, Peek MJ, Ellwood D, Homer CSE, et al. Maternal super-obesity and perinatal outcomes in Australia: a national population-based cohort study. BMC Pregnancy Childbirth 2015;15:322

Sultan AA, Tata LJ, West J, Fiaschi L, Fleming KM, Nelson-Piercy C, et al. Risk factors for first venous thromboembolism around pregnancy: a population-based cohort study from the United Kingdom. Blood 2013;121(19):3953-61.

Surapaneni T, Fernandez E. Obesity in gestational diabetes: Emerging twin challenge for perinatal care in India. Intl J Infertil Fetal Med 2010;1(1):35-9. [PMID: http://www.jaypeejournals.com/eJournals/ShowText.aspx?ID=762&Type=FREE&TYP=TOP&IN=_eJournals/International%20Journal%20of%20Infertility%20and%20Fetal%20Medicine.jpg&IID=70&AID=16&Year=2010&isPDF=YES]

Suresh A, Liu A, Poulton A, Quinton A, Amer Z, Mongelli M, et al. Comparison of maternal abdominal subcutaneous fat thickness and body mass index as markers for pregnancy outcomes: A stratified cohort study. Aust N Z J Obstet Gynaecol 2012;52(5):420-6.

Suzuki S, Inde Y, Miyake H. Maternal obesity as a risk factor for very pre-term delivery in dichorionic twin pregnancies. J Obstet Gynaecol 2010;30(4):354-6.

Svenvik M, Brudin L, Blomberg M. Preterm Birth: A Prominent Risk Factor for Low Apgar Scores. Biomed Res Int 2015;2015:978079

T. Premru-Srsen,I. Verdenik,Ponikvar B. Mihevc,O. Hugh,A. Francis,J. Gardosi. Customised birthweight standard for a Slovenian population. Journal of perinatal medicine. 2019/04/24/. 47:270

T.O. Egbe,E.S. Tsaku,R. Tchounzou,M.N. Ngowe. Prevalence and risk factors of gestational diabetes mellitus in a population of pregnant women attending three health facilities in Limbe, Cameroon: A cross-sectional study. Pan African Medical Journal. 2018///. 31:195

Tabet M, Jakhar S, Williams CA, Rawat U, Hailegiorgis YD, Flick LH, et al. Racial/Ethnic Differences in Correlates of Spontaneous and Medically-Indicated Late Preterm Births among Adolescents. J Pediatr Adolesc Gynecol 2016;

Taebi M, Sadat Z, Saberi F, Kalahroudi MA. Early pregnancy waist-to-hip ratio and risk of preeclampsia: a prospective cohort study. Hypertens Res 2015;38(1):80-3.

Tahseen S, Qurban S, Mazher R, Sultana N, Mahmood Z, Majeed T. Frequency of factors leading to gestational diabetes. Pak J Med Health Sci 2016;10(1):219-21. [PMID: http://www.pjmhsonline.com/2016/jan_march/pdf/219%20%20%20Frequency%20of%20Factors%20Leading%20to%20Gestational%20Diabetes.pdf]

Talebian A, Soltani B, Sehat M, Zahedi A, Noorian A, Talebian M. Incidence and Risk Factors of Neural Tube Defects in Kashan, Central Iran. Iran j child neurol 2015;9(3):50-6.

Temming L, Franco A, Istwan N, Rhea D, Desch C, Stanziano G, et al. Adverse pregnancy outcomes in women with nausea and vomiting of pregnancy. J Matern Fetal Neonatal Med 2014;27(1):84-8.

Temple R, Smith S. Intrauterine fetal demise: Care in the aftermath, and beyond. J Fam Pract 2014;63(6):E9-E13. [PMID: http://www.jfponline.com/fileadmin/qhi/jfp/pdfs/6306/JFP_06306_ArticleW2.pdf]

Tennant PWG, Rankin J, Bell R. Maternal body mass index and the risk of fetal and infant death: a cohort study from the North of England. Hum Reprod 2011;26(6):1501-11.

Tonidandel A, Booth J, D'Angelo R, Harris L, Tonidandel S. Anesthetic and obstetric outcomes in morbidly obese parturients: a 20-year follow-up retrospective cohort study. INT J OBSTET ANESTH 2014;23(4):357-64.

Torloni MR, Betran AP, Daher S, Widmer M, Dolan SM, Menon R, et al. Maternal BMI and preterm birth: a systematic review of the literature with meta-analysis. J Matern Fetal Neonatal Med 2009;22(11):957-70.

Torloni MR, Betran AP, Horta BL, Nakamura MU, Atallah AN, Moron AF, et al. Prepregnancy BMI and the risk of gestational diabetes: a systematic review of the literature with meta-analysis. Obes Rev 2009;10(2):194-203.

Van Der Linden EL, Browne JL, Vissers KM, Antwi E, Agyepong IA, Grobbee DE, et al. Maternal body mass index and adverse pregnancy outcomes: A ghanaian cohort study. Obesity (Silver Spring) 2016;24(1):215-22.

Van Mackelenbergh MT, Marotte M, Alkatout I, Von Kaisenberg CS, Eckmann-Scholz C. Increasing maternal body mass index is associated with fetal defects. Int J Women's Health Reproduction Sci 2016;4(4):164-70. [PMID: http://www.ijwhr.net/pdf.php?id=158]

Van Oostwaard MF, Langenveld J, Schuit E, Wigny K, Van Susante H, Beune I, et al. Prediction of recurrence of hypertensive disorders of pregnancy in the term period, a retrospective cohort study. Pregnancy Hypertens 2014;4(3):194-202.

Vaswani PR, Balachandran L. Pregnancy outcomes in a population with high prevalence of obesity: How bad is it? Clin Epidemiol Global Health 2013;1(1):5-11. [PMID: http://www.elsevier.com/journals/clinical-epidemiology-and-global-health/2213-3984]

Vellinga A, Zawiejska A, Harreiter J, Buckley B, Di Cianni G, Lapolla A, et al. Associations of Body Mass Index (Maternal BMI) and Gestational Diabetes Mellitus with Neonatal and Maternal Pregnancy Outcomes in a Multicentre European Database (Diabetes and Pregnancy Vitamin D and Lifestyle Intervention for Gestational Diabetes Mellitus Prevention). ISRN Obes 2012;2012:424010

Verma A, Shrimali L. Maternal body mass index and pregnancy outcome. J Clin Diagn Res 2012;6(9):1531-3.

Vidakovic AJ, Jaddoe VWV, Gishti O, Felix JF, Williams MA, Hofman A, et al. Body mass index, gestational weight gain and fatty acid concentrations during pregnancy: the Generation R Study. Eur J Epidemiol 2015;30(11):1175-85.

Vidal AC, Benjamin Neelon SE, Liu Y, Tuli AM, Fuemmeler BF, Hoyo C, et al. Maternal stress, preterm birth, and DNA methylation at imprint regulatory sequences in humans. Genet epigenet 2014;6:37-44.

Vinturache A, Moledina N, McDonald S, Slater D, Tough S. Pre-pregnancy Body Mass Index (BMI) and delivery outcomes in a Canadian population. BMC Pregnancy Childbirth 2014;14:422

Vinturache AE, McDonald S, Slater D, Tough S. Perinatal outcomes of maternal overweight and obesity in term infants: a population-based cohort study in Canada. Sci rep 2015;5:9334

Vintzileos AM, Finamore PS, Ananth CV. Inclusion of body mass index in the history of present illness. Obstet Gynecol 2013;121(1):59-64.

Virkus RA, Lokkegaard E, Lidegaard O, Langhoff-Roos J, Nielsen AK, Rothman KJ, et al. Risk factors for venous thromboembolism in 1.3 million pregnancies: a nationwide prospective cohort. PloS one 2014;9(5):e96495

Volgt M, Zels K, Guthmann F, Hesse V, Gorlich Y, Straube S. Somatic classification of neonates based on birth weight, length, and head circumference: Quantification of the effects of maternal BMI and smoking. J Perinat Med 2011;39(3):291-7.

W. Yang,F. Han,X. Gao,Y. Chen,L. Ji,X. Cai. Relationship Between Gestational Weight Gain and Pregnancy Complications or Delivery Outcome. Sci Rep. 2017///. 7:12531

Waldenstrom U, Aasheim V, Nilsen ABV, Rasmussen S, Pettersson HJ, Schytt E, et al. Adverse pregnancy outcomes related to advanced maternal age compared with smoking and being overweight. Obstet Gynecol 2014;123(1):104-12.

Waldman M, Sheiner E, Sergienko R, Shoham-Vardi I. Can we identify risk factors during pregnancy for thrombo-embolic events during the puerperium and later in life? J Matern Fetal Neonatal Med 2015;28(9):1005-9.

Wallace JM, Horgan GW, Bhattacharya S. Placental weight and efficiency in relation to maternal body mass index and the risk of pregnancy complications in women delivering singleton babies. Placenta 2012;33(8):611-8.

Wallis J. Perinatal outcomes in women with high BMI. RCM Midwives 2012;15(5):33

Walsh S, Donnan J, Morrissey A, Sikora L, Bowen S, Collins K, et al. A systematic review of the risks factors associated with the onset and natural progression of hydrocephalus. Neurotoxicology 2016;

Wang D, Hong Y, Zhu L, Wang X, Lv Q, Zhou Q, et al. Risk factors and outcomes of macrosomia in China: a multicentric survey based on birth data. J Matern -Fetal Neonatal Med 2016;1-5.

Whiteman VE, Salemi JL, Mejia De Grubb MC, Ashley Cain M, Mogos MF, Zoorob RJ, et al. Additive effects of Pre-pregnancy body mass index and gestational diabetes on health outcomes and costs. Obesity (Silver Spring) 2015;23(11):2299-308.

Willis K, Alexander C, Sheiner E. Bariatric Surgery and the Pregnancy Complicated by Gestational Diabetes. Curr Diab Rep 2016;16(4):21

Wilson BL, Dyer JM, Latendresse G, Wong B, Baksh L. Exploring the Psychosocial Predictors of Gestational Diabetes and Birth Weight. Journal of obstetric, gynecologic, and neonatal nursing : JOGNN / NAACOG 2015;44(6):760-71.

Witteveen T, Zwart JJ, Gast KB, Bloemenkamp KWM, van Roosmalen J. Overweight and severe acute maternal morbidity in a low-risk pregnant population in the Netherlands. PloS one 2013;8(9):e74494

Wong TY, Groen H, Faas MM, van Pampus MG. Clinical risk factors for gestational hypertensive disorders in pregnant women at high risk for developing preeclampsia. Pregnancy Hypertens 2013;3(4):248-53.

X. Jiang,M. Liu,Y. Song,J. Mao,M. Zhou,Z. Ma,X. Qian,Z. Han,T. Duan. The Institute of Medicine recommendation for gestational weight gain is probably not optimal among non-American pregnant women: a retrospective study from China. Journal of Maternal-Fetal and Neonatal Medicine. 2019///. 32:1353

X. Liu,G. Ding,W. Yang,X. Feng,Y. Li,H. Liu,Q. Zhang,L. Ji,D. Li. Maternal Body Mass Index and Risk of Congenital Heart Defects in Infants: A Dose-Response Meta-Analysis. BioMed research international. 2019///. 2019:1315796, 2019.:#pages#

Y. Zhu,M.M. Hedderson,C.P. Quesenberry,J. Feng,A. Ferrara. Central Obesity Increases the Risk of Gestational Diabetes Partially Through Increasing Insulin Resistance. Obesity. 2019///. 27:152

Y.E.G. Timmermans,K.D.G. van de Kant,J.S.M. Krumeich,L.J.I. Zimmermann,E. Dompeling,B.W. Kramer,L.L.J. Maassen,M.A.E. Spaanderman,A.C.E. Vreugdenhil. Socio-ecological determinants of lifestyle behavior of women with overweight or obesity before, during and after pregnancy: qualitative interview analysis in the Netherlands. BMC pregnancy and childbirth. 2020/02/12/. 20:105, 2020

Y.H. Chen,L. Li,W. Chen,Z.B. Liu,L. Ma,X.X. Gao,J.L. He,H. Wang,M. Zhao,Y.Y. Yang,X. Xu. Pre-pregnancy underweight and obesity are positively associated with small-for-gestational-age infants in a Chinese population. Sci Rep. 2019/10/29/. 9:15544, 2019

Y.H. Yu,L.M. Bodnar,K.P. Himes,M.M. Brooks,A.I. Naimi. Association of Overweight and Obesity Development Between Pregnancies With Stillbirth and Infant Mortality in a Cohort of Multiparous Women. Obstetrics and gynecology. 2020/03//. 135:634

Yalvac S, Esin S, Kocak O, Yirci B, Kandemir O. Effect of body mass index on latency periods after history-indicated cervical cerclage. Aust N Z J Obstet Gynaecol 2014;54(2):121-5.

Yao R, Park BY, Caughey AB. The effects of maternal obesity on perinatal outcomes among those born small for gestational age. J Matern Fetal Neonatal Med 2016;1-6.

Yazdani S, Yosofniyapasha Y, Nasab BH, Mojaveri MH, Bouzari Z. Effect of maternal body mass index on pregnancy outcome and newborn weight. BMC Res Notes 2012;5:34

Young OM, Twedt R, Catov JM. Pre-pregnancy maternal obesity and the risk of preterm preeclampsia in the American primigravida. Obesity (Silver Spring) 2016;24(6):1226-9.

Yuan W, Duffner AM, Chen L, Hunt LP, Sellers SM, Bernal AL. Analysis of preterm deliveries below 35 weeks' gestation in a tertiary referral hospital in the UK. A case-control survey. BMC Res Notes 2010;3:119

Z. Yang,H. Phung,L. Freebairn,R. Sexton,A. Raulli,P. Kelly. Contribution of maternal overweight and obesity to the occurrence of adverse pregnancy outcomes. Australian and New Zealand Journal of Obstetrics and Gynaecology. 2019///. 59:367

Z.F. Neuhaus,G. Gutvirtz,G. Pariente,T. Wainstock,D. Landau,E. Sheiner. Maternal obesity and long-term neuropsychiatric morbidity of the offspring. Archives of gynecology and obstetrics. 2020/01//. 301:143

Zaballa K, Liu A, Peek MJ, Mongelli M, Nanan R. Association between World Health Organization categories of body mass index and relative risks for weight-related pregnancy outcomes: a retrospective cohort study. Obstet med 2012;5(3):112-8.

Zander-Fox DL, Henshaw R, Hamilton H, Lane M. Does obesity really matter? The impact of BMI on embryo quality and pregnancy outcomes after IVF in women aged <38 years. Aust N Z J Obstet Gynaecol 2012;52(3):270-6.

Zawiejska A, Wender-Ozegowska E, Radzicka S, Brazert J. Maternal hyperglycemia according to IADPSG criteria as a predictor of perinatal complications in women with gestational diabetes: a retrospective observational study. J Matern Fetal Neonatal Med 2014;27(15):1526-30.

Zhang L, Wang XH, Zheng XM, Liu TZ, Zhang WB, Zheng H, et al. Maternal gestational smoking, diabetes, alcohol drinking, pre-pregnancy obesity and the risk of cryptorchidism: a systematic review and meta-analysis of observational studies. PloS one 2015;10(3):e0119006

Zhang Y, Wang ZL, Liu B, Cai J. Pregnancy outcome of overweight and obese Chinese women with gestational diabetes. J Obstet Gynaecol 2014;34(8):662-5.

Zhong Y, Cahill AG, Macones GA, Zhu F, Odibo AO. The association between prepregnancy maternal body mass index and preterm delivery. Am J Perinatol 2010;27(4):293-8.

Zilberlicht A, Feferkorn I, Younes G, Damti A, Auslender R, Riskin-Mashiah S. The mutual effect of pregestational body mass index, maternal hyperglycemia and gestational weight gain on adverse pregnancy outcomes. Gynecol Endocrinol 2016;32(5):416-20.

Zwink N, Jenetzky E, Brenner H. Parental risk factors and anorectal malformations: systematic review and meta-analysis. Orphanet J Rare Dis 2011;6:25

<u>No outcomes of interest</u>

Aly H, Hammad T, Nada A, Mohamed M, Bathgate S, El-Mohandes A. Maternal obesity, associated complications and risk of prematurity. J Perinatol 2010;30(7):447-51.

Baker AM, Haeri S. Estimating risk factors and perinatal outcomes for gestational diabetes and impaired glucose tolerance in teen mothers. Diabetes Metab Res Rev 2012;28(8):688-91.

Boots CE, Bernardi LA, Stephenson MD. Frequency of euploid miscarriage is increased in obese women with recurrent early pregnancy loss. Fertil Steril 2014;102(2):455-9.

Chakraborty, P.. Aspirin and Low-Molecular Weight Heparin Combination Therapy Effectively Prevents Recurrent Miscarriage in Hyperhomocysteinemic Women. #journal#. 2013. 8:#pages#

Chakraborty, P.. Aspirin and Low-Molecular Weight Heparin Combination Therapy Effectively Prevents Recurrent Miscarriage in Hyperhomocysteinemic Women. #journal#. 2013. 8:#pages#

Declercq E, MacDorman M, Cabral H, Stotland N. Prepregnancy Body Mass Index and Infant Mortality in 38 U.S. States, 2012-2013. Obstet Gynecol 2016;127(2):279-87.

Ellerbe CN, Gebregziabher M, Korte JE, Mauldin J, Hunt KJ. Quantifying the impact of gestational diabetes mellitus, maternal weight and race on birthweight via quantile regression. PloS one 2013;8(6):e65017

F. Weschenfelder,T. Lehmann,E. Schleussner,T. Groten. Gestational Weight Gain Particularly Affects the Risk of Large for Gestational Age Infants in Non-obese Mothers. Geburtshilfe und Frauenheilkunde. 2019/11//. 79:1183

F. Weschenfelder,T. Lehmann,E. Schleussner,T. Groten. Gestational Weight Gain Particularly Affects the Risk of Large for Gestational Age Infants in Non-obese Mothers. Geburtshilfe und Frauenheilkunde. 2019/11//. 79:1183

Facco FL, Liu CS, Cabello AA, Kick A, Grobman WA, Zee PC. Sleep-disordered breathing: a risk factor for adverse pregnancy outcomes? Am J Perinatol 2012;29(4):277-82.

Farah N, Maher N, Barry S, Kennelly M, Stuart B, Turner MJ. Maternal morbid obesity and obstetric outcomes. Obes Facts 2009;2(6):352-4.

Founds SA, Ren D, Roberts JM, Jeyabalan A, Powers RW. Follistatin-like 3 across gestation in preeclampsia and uncomplicated pregnancies among lean and obese women. Reprod Sci 2015;22(4):402-9.

Fridman M, Korst LM, Chow J, Lawton E, Mitchell C, Gregory KD. Trends in maternal morbidity before and during pregnancy in California. Am J Public Health 2014;104 Suppl 1:S49-S57

Goh JY, He S, Allen JC, Malhotra R, Tan TC. Maternal obesity is associated with a low serum progesterone level in early pregnancy. Horm mol biol clin investig 2016;27(3):97-100.

Gomez-Arango LF, Barrett HL, McIntyre HD, Callaway LK, Morrison M, Dekker Nitert M, et al. Increased Systolic and Diastolic Blood Pressure Is Associated With Altered Gut Microbiota Composition and Butyrate Production in Early Pregnancy. Hypertension 2016;68(4):974-81.

H. Amark,M. Westgren,M. Persson. Prediction of large-for-gestational-age infants in pregnancies complicated by obesity: A population-based cohort study. Acta obstetricia et gynecologica Scandinavica. 2019/06//. 98:769

H. Amark,M. Westgren,M. Persson. Prediction of large-for-gestational-age infants in pregnancies complicated by obesity: A population-based cohort study. Acta obstetricia et gynecologica Scandinavica. 2019/06//. 98:769

J.M. Petersen,S.E. Parker,C.M. Benedum,A.A. Mitchell,S.C. Tinker,M.M. Werler. Periconceptional folic acid and risk for neural tube defects among higher risk pregnancies. Birth defects research.Part A, Clinical and molecular teratology. 2019/11/15/. 111:1501

J.M. Petersen,S.E. Parker,C.M. Benedum,A.A. Mitchell,S.C. Tinker,M.M. Werler. Periconceptional folic acid and risk for neural tube defects among higher risk pregnancies. Birth defects research.Part A, Clinical and molecular teratology. 2019/11/15/. 111:1501

Kim SY, Sharma AJ, Sappenfield W, Wilson HG, Salihu HM. Association of maternal body mass index, excessive weight gain, and gestational diabetes mellitus with large-for-gestational-age births. Obstet Gynecol 2014;123(4):737-44.

L. Kong,I.A.K. Nilsson,K. Brismar,M. Gissler,C. Lavebratt. Associations of Different Types of Maternal Diabetes and Body Mass Index With Offspring Psychiatric Disorders. JAMA network open. 2020/02/05/. 3:e1920787

Li S, Rosenberg L, Palmer JR, Phillips GS, Heffner LJ, Wise LA. Central adiposity and other anthropometric factors in relation to risk of macrosomia in an African American population. Obesity (Silver Spring) 2013;21(1):178-84.

Lindsay KL, Gibney ER, McNulty BA, McAuliffe FM. Pregnant immigrant Nigerian women: an exploration of dietary intakes. Public health 2014;128(7):647-53.

Lo W, Rai R, Hameed A, Brailsford SR, Al-Ghamdi AA, Regan L. The effect of body mass index on the outcome of pregnancy in women with recurrent miscarriage. J Family Community Med 2012;19(3):167-71.

Lucovnik M, Blickstein I, Verdenik I, Trojner-Bregar A, Tul N. Maternal obesity in singleton versus twin gestations: a population-based matched case-control study. J Matern Fetal Neonatal Med 2015;28(6):623-5.

Luke B, Brown MB, Stern JE, Missmer SA, Fujimoto VY, Leach R. Racial and ethnic disparities in assisted reproductive technology pregnancy and live birth rates within body mass index categories. Fertil Steril 2011;95(5):1661-6.

M. Dias,A. Dick,R.M. Reynolds,M. Lahti-Pulkkinen,F.C. Denison. Predictors of surgical site skin infection and clinical outcome at caesarean section in the very severely obese: A retrospective cohort study. PloS one. 2019///. 14:e0216157, 2019

M. Dias,A. Dick,R.M. Reynolds,M. Lahti-Pulkkinen,F.C. Denison. Predictors of surgical site skin infection and clinical outcome at caesarean section in the very severely obese: A retrospective cohort study. PloS one. 2019///. 14:e0216157, 2019

Mestan K, Ouyang F, Matoba N, Pearson C, Ortiz K, Wang X. Maternal obesity, diabetes mellitus and cord blood biomarkers in large-for-gestational age infants. J Pediatr Biochem 2010;1(3):217-24.

Mutz-Dehbalaie I, Scheier M, Jerabek-Klestil S, Brantner C, Windbichler GH, Leitner H, et al. Perinatal mortality and advanced maternal age. Gynecol Obstet Invest 2014;77(1):50-7.

N. Auger,M. Bilodeau-Bertrand,R.M. Tith,L. Arbour. Bariatric surgery and the risk of congenital anomalies in subsequent pregnancies. The American journal of clinical nutrition. 2019/11/01/. 110:1168

N. Auger,M. Bilodeau-Bertrand,R.M. Tith,L. Arbour. Bariatric surgery and the risk of congenital anomalies in subsequent pregnancies. The American journal of clinical nutrition. 2019/11/01/. 110:1168

Olmos PR, Araya-Del-Pino AP, Gonzalez-Carvello CA, Laso-Ulloa P, Hodgson MI, Irribarra V, et al. Near-optimal glycemic control in Chilean women with pregestational type-2 diabetes: Persistent macrosomia relates to maternal pre-pregnancy overweight. Diabetes Res Clin Pract 2009;85(1):53-60.

Pu J, Zhao B, Wang EJ, Nimbal V, Osmundson S, Kunz L, et al. Racial/Ethnic Differences in Gestational Diabetes Prevalence and Contribution of Common Risk Factors. Paediatr Perinat Epidemiol 2015;29(5):436-43.

R.H. Benjamin,M.K. Ethen,M.A. Canfield,F. Hua,L.E. Mitchell. Association of interpregnancy change in body mass index and spina bifida. Birth defects research.Part A, Clinical and molecular teratology. 2019/11/01/. 111:1389

R.H. Benjamin,M.K. Ethen,M.A. Canfield,F. Hua,L.E. Mitchell. Association of interpregnancy change in body mass index and spina bifida. Birth defects research.Part A, Clinical and molecular teratology. 2019/11/01/. 111:1389

Reiss K, Breckenkamp J, Borde T, Brenne S, David M, Razum O. Contribution of overweight and obesity to adverse pregnancy outcomes among immigrant and non-immigrant women in Berlin, Germany. Eur J Public Health 2015;25(5):839-44.

S.F. Ehrlich,M.M. Hedderson,F. Xu,A. Ferrara. Diagnostic thresholds for pregnancy hyperglycemia, maternal weight status and the risk of childhood obesity in a diverse Northern California cohort using health care delivery system data. PloS one. 2019///. 14:e0216897, 2019

S.F. Ehrlich,M.M. Hedderson,F. Xu,A. Ferrara. Diagnostic thresholds for pregnancy hyperglycemia, maternal weight status and the risk of childhood obesity in a diverse Northern California cohort using health care delivery system data. PloS one. 2019///. 14:e0216897, 2019

Sridhar SB, Ferrara A, Ehrlich SF, Brown SD, Hedderson MM. Risk of large-for-gestational-age newborns in women with gestational diabetes by race and ethnicity and body mass index categories. Obstet Gynecol 2013;121(6):1255-62.

Uebel, K.. Effect of maternal obesity with and without gestational diabetes on offspring subcutaneous and preperitoneal adipose tissue development from birth up to year-1. #journal#. 2014. 14:#pages#

Uebel, K.. Effect of maternal obesity with and without gestational diabetes on offspring subcutaneous and preperitoneal adipose tissue development from birth up to year-1. #journal#. 2014. 14:#pages#

Zera CA, Seely EW, Wilkins-Haug LE, Lim KH, Parry SI, Mcelrath TF. The association of body mass index with serum angiogenic markers in normal and abnormal pregnancies. Am J Obstet Gynecol 2014;211(3):247

Not a predictor of interest

Baci Y, Ustuner I, Keskin HL, Ersoy R, Avsar AF. Effect of maternal obesity and weight gain on gestational diabetes mellitus. Gynecol Endocrinol 2013;29(2):133-6.

Baker AM, Haeri S. Estimating risk factors for development of preeclampsia in teen mothers. Arch Gynecol Obstet 2012;286(5):1093-6.

Balani J, Hyer S, Johnson A, Shehata H. The importance of visceral fat mass in obese pregnant women and relation with pregnancy outcomes. Obstet med 2014;7(1):22-5.

Baliutaviciene D, Buinauskiene JB, Petrenko V, Danyte E, Zalinkevicius R. Gestational diabetes, obesity, and metabolic syndrome diagnosed during pregnancy. Metab syndr relat disord 2012;10(3):214-7.

Baragou S, Goeh-Akue E, Pio M, Afassinou YM, Atta B. [Hypertension and pregnancy in Lome (sub-Saharan Africa): epidemiology, diagnosis and risk factors]. Ann Cardiol Angeiol (Paris) 2014;63(3):145-50.

Barquiel B, Herranz L, Grande C, Castro-Dufourny I, Llaro M, Parra P, et al. Body weight, weight gain and hyperglycaemia are associated with hypertensive disorders of pregnancy in women with gestational diabetes. Diabetes Metab 2014;40(3):204-10.

Bener A, Al-Hamaq AO, Saleh NM. Association between vitamin D insufficiency and adverse pregnancy outcome: global comparisons. Int J Women Health 2013;5:523-31.

Bigelow CA, Pereira GA, Warmsley A, Cohen J, Getrajdman C, Moshier E, et al. Risk factors for new-onset late postpartum preeclampsia in women without a history of preeclampsia. Am J Obstet Gynecol 2014;210(4):338

Black MH, Sacks DA, Xiang AH, Lawrence JM. The relative contribution of prepregnancy overweight and obesity, gestational weight gain, and IADPSG-defined gestational diabetes mellitus to fetal overgrowth. Diabetes care 2013;36(1):56-62.

Block-Abraham DM, Turan OM, Doyle LE, Kopelman JN, Atlas RO, Jenkins CB, et al. First-trimester risk factors for preeclampsia development in women initiating aspirin by 16 weeks of gestation. Obstet Gynecol 2014;123(3):611-7.

Blomberg M. Maternal obesity and risk of postpartum hemorrhage. Obstet Gynecol 2011;118(3):561-8.

Blomberg M. Maternal obesity, mode of delivery, and neonatal outcome. Obstet Gynecol 2013;122(1):50-5.

Bodnar LM, Siminerio LL, Himes KP, Hutcheon JA, Lash TL, Parisi SM, et al. Maternal obesity and gestational weight gain are risk factors for infant death. Obesity (Silver Spring) 2016;24(2):490-8.

Bodnar LM, Pugh SJ, Lash TL, Hutcheon JA, Himes KP, Parisi SM, et al. Low Gestational Weight Gain and Risk of Adverse Perinatal Outcomes in Obese and Severely Obese Women. Epidemiology 2016;27(6):894-902.

Boyle KE, Hwang H, Janssen RC, DeVente JM, Barbour LA, Hernandez TL, et al. Gestational diabetes is characterized by reduced mitochondrial protein expression and altered calcium signaling proteins in skeletal muscle. PloS one 2014;9(9):e106872

Bozkurt L, Gobl CS, Hormayer AT, Luger A, Pacini G, Kautzky-Willer A. The impact of preconceptional obesity on trajectories of maternal lipids during gestation. Sci rep 2016;6:29971

Brite J, Laughon SK, Troendle J, Mills J. Maternal overweight and obesity and risk of congenital heart defects in offspring. Int J Obes 2014;38(6):878-82. [PMID: http://www.nature.com/ijo/index.html]

Callegari LS, Sterling LA, Zelek ST, Hawes SE, Reed SD. Interpregnancy body mass index change and success of term vaginal birth after cesarean delivery. Am J Obstet Gynecol 2014;210(4):330-7.

Catalano PM, McIntyre HD, Cruickshank JK, McCance DR, Dyer AR, Metzger BE, et al. The hyperglycemia and adverse pregnancy outcome study: associations of GDM and obesity with pregnancy outcomes. Diabetes care 2012;35(4):780-6.

Catalano PM, Mele L, Landon MB, Ramin SM, Reddy UM, Casey B, et al. Inadequate weight gain in overweight and obese pregnant women: what is the effect on fetal growth? Am J Obstet Gynecol 2014;211(2):137

Catov JM, Abatemarco D, Althouse A, Davis EM, Hubel C. Patterns of gestational weight gain related to fetal growth among women with overweight and obesity. Obesity (Silver Spring) 2015;23(5):1071-8.

Chandrasekaran S, Levine LD, Durnwald CP, Elovitz MA, Srinivas SK. Excessive weight gain and hypertensive disorders of pregnancy in the obese patient. J Matern Fetal Neonatal Med 2015;28(8):964-8.

Chen A, Feresu SA, Fernandez C, Rogan WJ. Maternal obesity and the risk of infant death in the United States. Epidemiology 2009;20(1):74-81.

Clark-Ganheart CA, Reddy UM, Kominiarek MA, Huang CC, Landy HJ, Grantz KL. Pregnancy Outcomes Among Obese Women and Their Offspring by Attempted Mode of Delivery. Obstet Gynecol 2015;126(5):987-93.

Co AL, Walker HC, Hade EM, Iams JD. Relation of body mass index to frequency of recurrent preterm birth in women treated with 17-alpha hydroxyprogesterone caproate. Am J Obstet Gynecol 2015;213(2):233-5.

Colmorn LB, Ladelund S, Rasmussen S, Secher NJ. Risk of a venous thromboembolic episode due to caesarean section and BMI: a study in northern Denmark covering 2000-2010. J Obstet Gynaecol 2014;34(4):313-6.

Colombara DV, Soh JD, Menacho LA, Schiff MA, Reed SD. Birth injury in a subsequent vaginal delivery among women with a history of shoulder dystocia. J Perinat Med 2011;39(6):709-15.

Cox Bauer CM, Bernhard KA, Greer DM, Merrill DC. Maternal and neonatal outcomes in obese women who lose weight during pregnancy. J Perinatol 2016;36(4):278-83.

Davenport MH, Campbell MK, Mottola MF. Increased incidence of glucose disorders during pregnancy is not explained by pre-pregnancy obesity in London, Canada. BMC Pregnancy Childbirth 2010;10:85

de Paiva LV, Nomura RMY, Dias MCG, Zugaib M. Maternal obesity in high-risk pregnancies and postpartum infectious complications. Rev Assoc Med Bras 2012;58(4):453-8. [PMID: http://www.scielo.br/pdf/ramb/v58n4/en_v58n4a16.pdf]

Dickey RP, Xiong X, Xie Y, Gee RE, Pridjian G. Effect of maternal height and weight on risk for preterm singleton and twin births resulting from IVF in the United States, 2008-2010. Am J Obstet Gynecol 2013;209(4):349-6.

Dzakpasu S, Fahey J, Kirby RS, Tough SC, Chalmers B, Heaman MI, et al. Contribution of prepregnancy body mass index and gestational weight gain to adverse neonatal outcomes: population attributable fractions for Canada. BMC Pregnancy Childbirth 2015;15:21

Faucher MA, Hastings-Tolsma M, Song JJ, Willoughby DS, Bader SG. Gestational weight gain and preterm birth in obese women: a systematic review and meta-analysis. BJOG 2016;123(2):199-206.

Gante I, Amaral N, Dores J, Almeida MC. Impact of gestational weight gain on obstetric and neonatal outcomes in obese diabetic women. BMC Pregnancy Childbirth 2015;15:249

Gaskins AJ, Rich-Edwards JW, Colaci DS, Afeiche MC, Toth TL, Gillman MW, et al. Prepregnancy and early adulthood body mass index and adult weight change in relation to fetal loss. Obstet Gynecol 2014;124(4):662-9.

Gavard JA, Artal R. Gestational weight gain and maternal and neonatal outcomes in term twin pregnancies in obese women. Twin Res Hum Genet 2014;17(2):127-33.

Ghosh S, Ghosh K. Maternal and neonatal outcomes in gestational diabetes mellitus. J Indian Med Assoc 2013;111(5):330. [PMID: http://www.jima.in/images/may-2013-web.pdf]

Gudnadottir TA, Bateman BT, Hernadez-Diaz S, Luque-Fernandez MA, Valdimarsdottir U, Zoega H. Body Mass Index, Smoking and Hypertensive Disorders during Pregnancy: A Population Based Case-Control Study. PloS one 2016;11(3):e0152187

Gunatilake RP, Smrtka MP, Harris B, Kraus DM, Small MJ, Grotegut CA, et al. Predictors of failed trial of labor among women with an extremely obese body mass index. Am J Obstet Gynecol 2013;209(6):562

Hermann M, Le Ray C, Blondel B, Goffinet F, Zeitlin J. The risk of prelabor and intrapartum cesarean delivery among overweight and obese women: possible preventive actions. Am J Obstet Gynecol 2015;212(2):241-9.

Hilden K, Hanson U, Persson M, Fadl H. Overweight and obesity: a remaining problem in women treated for severe gestational diabetes. Diabet Med 2016;33(8):1045-51.

Hilliard AM, Chauhan SP, Zhao Y, Rankins NC. Effect of obesity on length of labor in nulliparous women. Am J Perinatol 2012;29(2):127-32.

Hollowell J, Pillas D, Rowe R, Linsell L, Knight M, Brocklehurst P. The impact of maternal obesity on intrapartum outcomes in otherwise low risk women: secondary analysis of the Birthplace national prospective cohort study. BJOG 2014;121(3):343-55.

Huang A, Ji Z, Zhao W, Hu H, Yang Q, Chen D. Rate of gestational weight gain and preterm birth in relation to prepregnancy body mass indices and trimester: a follow-up study in China. Reprod Health 2016;13(1):93

Hung TH, Hsieh TT. Pregestational body mass index, gestational weight gain, and risks for adverse pregnancy outcomes among Taiwanese women: A retrospective cohort study. Taiwan j obstet gynecol 2016;55(4):575-81.

Ip F, Bradford J, Hng TM, Hendon S, McLean M. The obese woman with gestational diabetes: effects of body mass index and weight gain in pregnancy on obstetric and glycaemic outcomes. Obstet med 2012;5(2):65-70.

Joy S, Roman A, Istwan N, Rhea D, Desch C, Stanziano G, et al. The effect of maternal obesity on pregnancy outcomes of women with gestational diabetes controlled with diet only, glyburide, or insulin. Am J Perinatol 2012;29(8):643-8.

Kapadia MZ, Park CK, Beyene J, Giglia L, Maxwell C, McDonald SD. Weight Loss Instead of Weight Gain within the Guidelines in Obese Women during Pregnancy: A Systematic Review and Meta-Analyses of Maternal and Infant Outcomes. PloS one 2015;10(7):e0132650

Kasim K, Roshdy A. Body mass index and pregnancy outcome after assisted reproduction treatment. int j reprod med 2014;2014:257974

Khalak R, Cummings J, Dexter S. Maternal obesity: significance on the preterm neonate. Int J Obes (Lond) 2015;39(10):1433-6.

Kominiarek MA, Rankin K, Handler A. Provider adherence to recommended prenatal care content: does it differ for obese women? Matern Child Health J 2014;18(5):1114-22.

Kurnit KC, Overcash RT, Ramos GA, LaCoursiere DY. The impact of inadequate gestational weight gain in obese diabetic women. J Perinatol 2016;36(2):86-9.

La MM, Stein CR, Landrigan P, Engel SM, Savitz DA. Prepregnancy Body Mass Index, Smoking During Pregnancy, and Infant Birth Weight. Ann Epidemiol 2011;21(6):413-20.

Lapolla A, Bonomo M, Dalfra MG, Parretti E, Mannino D, Mello G, et al. Prepregnancy BMI influences maternal and fetal outcomes in women with isolated gestational hyperglycaemia: a multicentre study. Diabetes Metab 2010;36(4):265-70.

Lengyel CS, Ehrlich S, Iams JD, Muglia LJ, DeFranco EA. Effect of Modifiable Risk Factors on Preterm Birth: A Population Based-Cohort. Matern Child Health J 2016;

Li C, Liu Y, Zhang W. Joint and Independent Associations of Gestational Weight Gain and Pre-Pregnancy Body Mass Index with Outcomes of Pregnancy in Chinese Women: A Retrospective Cohort Study. PloS one 2015;10(8):e0136850

Lin L-T, Tsui K-H, Cheng J-T, Cheng J-S, Huang W-C, Liou W-S, et al. Increased Risk of Intracranial Hemorrhage in Women with Pregnancy-Induced Hypertension. Medicine (United States) 2016;95(20):no. [PMID: http://journals.lww.com/md-journal]

Liu J, Gallagher AE, Carta C, Torres ME, Moran R, Wilcox S. Racial differences in gestational weight gain and pregnancy-related hypertension. Ann Epidemiol 2014;24(6):441-7.

Lynch AM, Hart JE, Agwu OC, Fisher BM, West NA, Gibbs RS. Association of extremes of prepregnancy BMI with the clinical presentations of preterm birth. Am J Obstet Gynecol 2014;210(5):428-9.

MacInnis N, Woolcott CG, McDonald S, Kuhle S. Population Attributable Risk Fractions of Maternal Overweight and Obesity for Adverse Perinatal Outcomes. Sci rep 2016;6:22895

Marrs CC, Moussa HN, Sibai BM, Blackwell SC. The relationship between primary cesarean delivery skin incision type and wound complications in women with morbid obesity. Am J Obstet Gynecol 2014;210(4):319-4.

Marshall NE, Guild C, Cheng YW, Caughey AB, Halloran DR. Maternal superobesity and perinatal outcomes. Am J Obstet Gynecol 2012;206(5):417-6.

Martin KE, Grivell RM, Yelland LN, Dodd JM. The influence of maternal BMI and gestational diabetes on pregnancy outcome. Diabetes Res Clin Pract 2015;108(3):508-13.

Masho SW, Urban P, Cha S, Ramus R. Body Mass Index, Weight Gain, and Hypertensive Disorders in Pregnancy. Am J Hypertens 2016;29(6):763-71.

McBain RD, Dekker GA, Clifton VL, Mol BW, Grzeskowiak LE. Impact of inter-pregnancy BMI change on perinatal outcomes: a retrospective cohort study. Eur J Obstet Gynecol Reprod Biol 2016;205:98-104.

McDonnold M, Mele LM, Myatt L, Hauth JC, Leveno KJ, Reddy UM, et al. Waist-to-Hip Ratio versus Body Mass Index as Predictor of Obesity-Related Pregnancy Outcomes. Am J Perinatol 2016;33(6):618-24.

Minsart AF, Buekens P, De Spiegelaere M, Englert Y. Neonatal outcomes in obese mothers: a population-based analysis. BMC Pregnancy Childbirth 2013;13:36

Murphy NM, McCarthy FP, Khashan AS, Myers JE, Simpson NAB, Kearney PM, et al. Compliance with National Institute of Health and Care Excellence risk-based screening for Gestational Diabetes Mellitus in nulliparous women. Eur J Obstet Gynecol Reprod Biol 2016;199:60-5.

Nayak M, Peinhaupt M, Heinemann A, Eekhoff MEW, van Mechelen W, Desoye G, et al. Sedentary behavior in obese pregnant women is associated with inflammatory markers and lipid profile but not with glucose metabolism. Cytokine 2016;88:91-8.

Nohr EA, Villamor E, Vaeth M, Olsen J, Cnattingius S. Mortality in infants of obese mothers: is risk modified by mode of delivery? Acta Obstet Gynecol Scand 2012;91(3):363-71.

Owens LA, O'Sullivan EP, Kirwan B, Avalos G, Gaffney G, Dunne F, et al. ATLANTIC DIP: the impact of obesity on pregnancy outcome in glucose-tolerant women. Diabetes care 2010;33(3):577-9.

Palatnik A, Miller ES, Son M, Kominiarek MA. Association among Maternal Obesity, Cervical Length, and Preterm Birth. Am J Perinatol 2016;

Parellada CB, Asbjornsdottir B, Ringholm L, Damm P, Mathiesen ER. Fetal growth in relation to gestational weight gain in women with type 2 diabetes: an observational study. Diabet Med 2014;31(12):1681-9.

Ramirez VI, Miller E, Meireles CL, Gelfond J, Krummel DA, Powell TL. Adiponectin and IGFBP-1 in the development of gestational diabetes in obese mothers. BMJ open diabetes res care 2014;2(1):e000010

Reddy UM, Laughon SK, Sun L, Troendle J, Willinger M, Zhang J. Prepregnancy risk factors for antepartum stillbirth in the United States. Obstet Gynecol 2010;116(5):1119-26.

Riley KL, Carmichael SL, Mayo JA, Shachar BZ, Girsen AI, Wallenstein MB, et al. Body Mass Index Change between Pregnancies and Risk of Spontaneous Preterm Birth. Am J Perinatol 2016;33(10):1017-22.

Salihu HM, Mbah AK, Alio AP, Kornosky JL, Bruder K, Belogolovkin V. Success of programming fetal growth phenotypes among obese women. Obstet Gynecol 2009;114(2 Pt 1):333-9.

Salihu HM, Weldeselasse HE, Rao K, Marty PJ, Whiteman VE. The impact of obesity on maternal morbidity and feto-infant outcomes among macrosomic infants. J Matern Fetal Neonatal Med 2011;24(9):1088-94.

Scifres C, Feghali M, Althouse AD, Caritis S, Catov J. Adverse Outcomes and Potential Targets for Intervention in Gestational Diabetes and Obesity. Obstet Gynecol 2015;126(2):316-25.

Sun D, Li F, Zhang Y, Xu X. Associations of the pre-pregnancy BMI and gestational BMI gain with pregnancy outcomes in Chinese women with gestational diabetes mellitus. Int J Clin Exp Med 2014;7(12):5784-9.

Swank ML, Caughey AB, Farinelli CK, Main EK, Melsop KA, Gilbert WM, et al. The impact of change in pregnancy body mass index on the development of gestational hypertensive disorders. J Perinatol 2014;34(3):181-5.

Sween LK, Althouse AD, Roberts JM. Early-pregnancy percent body fat in relation to preeclampsia risk in obese women. Am J Obstet Gynecol 2015;212(1):84-7.

Thuot M, Coursol MA, Nguyen S, Lacasse-Guay V, Beauchesne MF, Fillion A, et al. Impact of obesity on perinatal outcomes among asthmatic women. Can Respir J 2013;20(5):345-50.

Timofeev J, Feghali M, Boyle A, Istwan N, Rhea D, Driggers RW. Rates of recurrent preterm birth by maternal body habitus in women receiving 17alpha-hydroxyprogesterone caproate. J Matern Fetal Neonatal Med 2013;26(9):881-4.

Tucker CM, Berrien K, Menard MK, Herring AH, Daniels J, Rowley DL, et al. Predicting Preterm Birth Among Women Screened by North Carolina's Pregnancy Medical Home Program. Matern Child Health J 2015;19(11):2438-52.

Uebel K, Pusch K, Gedrich K, Schneider KT, Hauner H, Bader BL. Effect of maternal obesity with and without gestational diabetes on offspring subcutaneous and preperitoneal adipose tissue development from birth up to year-1. BMC Pregnancy Childbirth 2014;14:138

Vanderlelie J, Scott R, Shibl R, Lewkowicz J, Perkins A, Scuffham PA. First trimester multivitamin/mineral use is associated with reduced risk of pre-eclampsia among overweight and obese women. Matern Child Nutr 2016;12(2):339-48. [PMID: http://onlinelibrary.wiley.com/journal/10.1111/(ISSN)1740-8709]

Vassilaki M, Chatzi L, Georgiou V, Philalithis A, Kritsotakis G, Koutis A, et al. Pregestational excess weight, maternal obstetric complications and mode of delivery in the Rhea cohort in Crete. Eur J Public Health 2015;25(4):632-7.

Vidanalage CJK, Senarth U, Silva KD, Lekamge U, Liyanage IJ. Effects of initial body mass index on development of gestational diabetes in a rural Sri Lankan population: A case-control study. Diabetes Metab Syndr 2016;10(2 Suppl 1):S110-S113

Vinturache AE, Chaput KH, Tough SC. Pre-pregnancy body mass index (BMI) and macrosomia in a Canadian birth cohort. J Matern Fetal Neonatal Med 2016;1-8.

Wahabi HA, Fayed AA, Alzeidan RA, Mandil AA. The independent effects of maternal obesity and gestational diabetes on the pregnancy outcomes. BMC Endocr Disord 2014;14:47

Wang Z, Wang P, Liu H, He X, Zhang J, Yan H, et al. Maternal adiposity as an independent risk factor for pre-eclampsia: a meta-analysis of prospective cohort studies. Obes Rev 2013;14(6):508-21.

Whiteman VE, Crisan L, McIntosh C, Alio AP, Duan J, Marty PJ, et al. Interpregnancy body mass index changes and risk of stillbirth. Gynecol Obstet Invest 2011;72(3):192-5.

Wolfe H, Timofeev J, Tefera E, Desale S, Driggers RW. Risk of cesarean in obese nulliparous women with unfavorable cervix: elective induction vs expectant management at term. Am J Obstet Gynecol 2014;211(1):53-5.

Yao R, Ananth CV, Park BY, Pereira L, Plante LA, Perinatal Research Consortium. Obesity and the risk of stillbirth: a population-based cohort study. Am J Obstet Gynecol 2014;210(5):457-9.

Yeung EH, McLain AC, Anderson N, Lawrence D, Boghossian NS, Druschel C, et al. Newborn Adipokines and Birth Outcomes. Paediatr Perinat Epidemiol 2015;29(4):317-25.

Intent of the study

Bashiri A, Heo HJ, Ben-Avraham D, Mazor M, Budagov T, Einstein FH, et al. Pregnancy complicated by obesity induces global transcript expression alterations in visceral and subcutaneous fat. Mol Genet Genomics 2014;289(4):695-705.

Carlson NS, Lowe NK. Intrapartum management associated with obesity in nulliparous women. J Midwifery Womens Health 2014;59(1):43-53.

Daemers DOA, Wijnen HAA, van Limbeek EBM, Bude LM, Nieuwenhuijze MJ, Spaanderman MEA, et al. The impact of obesity on outcomes of midwife-led pregnancy and childbirth in a primary care population: a prospective cohort study. BJOG 2014;121(11):1403-13.

Facco FL, Ouyang DW, Zee PC, Grobman WA. Sleep disordered breathing in a high-risk cohort prevalence and severity across pregnancy. Am J Perinatol 2014;31(10):899-904.

Feichtinger M, Stopp T, Hofmann S, Springer S, Pils S, Kautzky-Willer A, et al. Altered glucose profiles and risk for hypoglycaemia during oral glucose tolerance testing in pregnancies after gastric bypass surgery. Diabetologia 2017;60(1):153-7.

Hartley E, McPhie S, Skouteris H, Fuller-Tyszkiewicz M, Hill B. Psychosocial risk factors for excessive gestational weight gain: A systematic review. Women Birth 2015;28(4):e99-e109

He M, Curran P, Raker C, Martin S, Larson L, Bourjeily G. Placental findings associated with maternal obesity at early pregnancy. Pathol Res Pract 2016;212(4):282-7.

Homer CSE, Kurinczuk JJ, Spark P, Brocklehurst P, Knight M. Planned vaginal delivery or planned caesarean delivery in women with extreme obesity. BJOG 2011;118(4):480-7.

Kandil M, Sanad Z, Sayyed T, Ellakwa H. Body mass index is linked to cervical length and duration of pregnancy: An observational study in low risk pregnancy. J Obstet Gynaecol 2016;1-5.

Liong S, Lappas M. Endoplasmic reticulum stress is increased in adipose tissue of women with gestational diabetes. PloS one 2015;10(4):e0122633

Perdu S, Castellana B, Kim Y, Chan K, DeLuca L, Beristain AG. Maternal obesity drives functional alterations in uterine NK cells. JCI insight 2016;1(11):e85560

Salem SY, Shahaf G, Sheiner E, Levinson J, Baron J, Madar T, et al. Diminished activity of circulating alpha1-antitrypsin is associated with pre-gestational isolated obesity. J Matern Fetal Neonatal Med 2015;28(5):500-3.

Sharadha SO, Punithavathi N, Renuka Devi TK. Better Predictor of Adverse Pregnancy Outcome: Asian or WHO International Cutoff? A Single-Centre Prospective Study. J Obstet Gynaecol India 2016;66(Suppl 1):181-6.

Slavin VJ, Fenwick J, Gamble J. Pregnancy care and birth outcomes for women with moderate to super-extreme obesity. Women Birth 2013;26(3):179-84.

van Poppel MNM, Peinhaupt M, Eekhoff MEW, Heinemann A, Oostdam N, Wouters MGAJ, et al. Physical activity in overweight and obese pregnant women is associated with higher levels of proinflammatory cytokines and with reduced insulin response through interleukin-6. Diabetes care 2014;37(4):1132-9.

Pre-pregnancy BMI not reported

Logie JJ, Denison FC, Riley SC, Ramaesh T, Forbes S, Norman JE, et al. Evaluation of kisspeptin levels in obese pregnancy as a biomarker for pre-eclampsia. Clin Endocrinol (Oxf) 2012;76(6):887-93.

Lucovnik M, Tul N, Verdenik I, Novak Z, Blickstein I. Risk factors for preeclampsia in twin pregnancies: a population-based matched case-control study. J Perinat Med 2012;40(4):379-82.

Roos N, Neovius M, Cnattingius S, Trolle Lagerros Y, Saaf M, Granath F, et al. Perinatal outcomes after bariatric surgery: nationwide population based matched cohort study. BMJ 2013;347:f6460

Scifres CM, Catov JM, Simhan H. Maternal serum fatty acid binding protein 4 (FABP4) and the development of preeclampsia. J Clin Endocrinol Metab 2012;97(3):E349-E356. [PMID: http://jcem.endojournals.org/content/97/3/E349.full.pdf+html]

Study design

A.M. Valent,C. Dearmond,J.M. Houston,S. Reddy,H.R. Masters,A. Gold,M. Boldt,E. Defranco,A.T. Evans,C.R. Warshak. Effect of Post-Cesarean Delivery Oral Cephalexin and Metronidazole on Surgical Site Infection among Obese Women: a Randomized Clinical Trial. Obstetrical and Gynecological Survey. 2018///. 73:855210

Abell SK, Nankervis A, Khan KS, Teede HJ. Type 1 and Type 2 Diabetes Preconception and in Pregnancy: Health Impacts, Influence of Obesity and Lifestyle, and Principles of Management. Semin Reprod Med 2016;34(2):110-20.

Barbour LA. Changing perspectives in pre-existing diabetes and obesity in pregnancy: maternal and infant short- and long-term outcomes. Curr opin endocrinol diabetes obes 2014;21(4):257-63.

Bjelanovic V, Babic D, Hodzic D, Bjelanovic A, Kresic T, Dugandzic-Simic A, et al. Correlation of psychological symptoms with cortisol and CRP levels in pregnant women with metabolic syndrome. PSYCHIATR DANUB 2015;27 Suppl 2:578-85.

Burket BA. Obstetric characteristics of two Mayan populations in the highlands of Guatemala. Ethn Health 2016;1-10.

C. Marrs,S. Blackwell,A. Hester,G. Olson,G.R. SAADE,J. Faro,C. Pedroza,B. Sibai. Pfannenstiel versus Vertical Skin Incision for Cesarean Delivery in Women with Class III Obesity: A Randomized Trial. American journal of perinatology. 2019///. 36:97

Cain MA, Louis JM. Sleep Disordered Breathing and Adverse Pregnancy Outcomes. Clin Lab Med 2016;36(2):435-46.

Campbell EE, Dworatzek PDN, Penava D, de Vrijer B, Gilliland J, Matthews JI, et al. Factors that influence excessive gestational weight gain: moving beyond assessment and counselling. J Matern Fetal Neonatal Med 2016;29(21):3527-31.

Chevrot A, Lesage N, Msika S, Mandelbrot L. [Digestive surgical complications during pregnancy following bariatric surgery: Experience of a center for perinatology and obesity]. J Gynecol Obstet Biol Reprod (Paris) 2016;45(4):372-9.

Cooper DL, Petherick ES, Wright J. Lifestyle related risk factors in a multi-ethnic cohort of pregnant women: Preliminary results from the Born in Bradford study. Public health 2013;127(11):1034-7.

Coton SJ, Nazareth I, Petersen I. A cohort study of trends in the prevalence of pregestational diabetes in pregnancy recorded in UK general practice between 1995 and 2012. BMJ open 2016;6(1):e009494

de WL, Jelsma JGM, van Poppel MNM, Bogaerts A, Simmons D, Desoye G, et al. Physical activity, depressed mood and pregnancy worries in European obese pregnant women: Results from the DALI

study. BMC Pregnancy Childbirth 2015;15(1):no. [PMID: http://www.biomedcentral.com/bmcpregnancychildbirth/]

Deruelle P, Vambergue A. Diabetes and obesity: An obstetrical challenge! Med Mal Metab 2012;6(4):294-9.

Dinatale A, Ermito S, Fonti I, Giordano R, Cacciatore A, Romano M, et al. Obesity and fetal-maternal outcomes. J Prenat Med 2010;4(1):5-8.

Egerman R, Ramsey R, Istwan N, Rhea D, Stanziano G. Maternal characteristics influencing the development of gestational diabetes in obese women receiving 17-alpha-hydroxyprogesterone caproate. J Obes 2014;2014:563243

Faucher MA, Barger MK. Gestational weight gain in obese women by class of obesity and select maternal/newborn outcomes: A systematic review. Women Birth 2015;28(3):e70-e79

Ferraro ZM, Chaput JP, Gruslin A, Adamo KB. The potential value of sleep hygiene for a healthy pregnancy: a brief review. ISRN Family Med 2014;2014:928293

Franco RC, Ferreira CR, Vieira CR, Silva RR. Ethnicity, Obesity and Emotional Factors Associated With Gestational Hypertension. J Community Health 2015;40(5):899-904.

Gidiri M, Greer IA. Pregnancy after bariatric surgery: no problem? Obstet med 2009;2(1):11-6.

Goldstein R, Teede H, Thangaratinam S, Boyle J. Excess Gestational Weight Gain in Pregnancy and the Role of Lifestyle Intervention. Semin Reprod Med 2016;34(2):e14-e21

Harrison CL, Brown WJ, Hayman M, Moran LJ, Redman LM. The Role of Physical Activity in Preconception, Pregnancy and Postpartum Health. Semin Reprod Med 2016;34(2):e28-e37

Hayes L, Bell R, Robson S, Poston L, UPBEAT Consortium. Association between physical activity in obese pregnant women and pregnancy outcomes: the UPBEAT pilot study. Ann Nutr Metab 2014;64(3-4):239-46.

Hezelgrave NL, Oteng-Ntim E. Pregnancy after bariatric surgery: a review. J Obes 2011;2011:501939

Higher risk of preterm delivery in overweight and obese women. BMJ 2013;346:no

K.A.M. Okesene-Gafa,M. Li,C.J.D. McKinlay,R.S. Taylor,E.C. Rush,C.R. Wall,J. Wilson,R. Murphy,R. Taylor,J.M.D. Thompson,C.A. Crowther,L.M.E. McCowan. Effect of antenatal dietary interventions in maternal obesity on pregnancy weight-gain and birthweight: Healthy Mums and Babies (HUMBA) randomized trial. American journal of obstetrics and gynecology. 2019/08//. 221:152

Kennelly MA, McAuliffe FM. Prediction and prevention of Gestational Diabetes: an update of recent literature. Eur J Obstet Gynecol Reprod Biol 2016;202:92-8.

Kevane B, Donnelly J, D'Alton M, Cooley S, Preston RJS, Ni Ainle F. Risk factors for pregnancy-associated venous thromboembolism: a review. J Perinat Med 2014;42(4):417-25.

Lim WY, Kwek K, Chong YS, Lee YS, Yap F, Chan YH, et al. Maternal adiposity and blood pressure in pregnancy: varying relations by ethnicity and gestational diabetes. J Hypertens 2014;32(4):857-64.

Maitland RA, Seed PT, Briley AL, Homsy M, Thomas S, Pasupathy D, et al. Prediction of gestational diabetes in obese pregnant women from the UK Pregnancies Better Eating and Activity (UPBEAT) pilot trial. Diabet Med 2014;31(8):963-70.

Mariona FG. DOES MATERNAL OBESITY IMPACT PREGNANCY RELATED DEATHS? Michigan Experience. J Matern Fetal Neonatal Med 2016;1-13.

Meinila J, Koivusalo SB, Valkama A, Rono K, Erkkola M, Kautiainen H, et al. Nutrient intake of pregnant women at high risk of gestational diabetes. Food Nutr Res 2015;59:26676

Mitanchez D, Burguet A, Simeoni U. Infants born to mothers with gestational diabetes mellitus: Mild neonatal effects, a long-term threat to global health. J Pediatr 2014;164(3):445-50.

Moran LJ, Dodd J, Nisenblat V, Norman RJ. Obesity and reproductive dysfunction in women. Endocrinol Metab Clin North Am 2011;40(4):895-906.

Morgan ES, Wilson E, Watkins T, Gao F, Hunt BJ. Maternal obesity and venous thromboembolism. INT J OBSTET ANESTH 2012;21(3):253-63.

Nagl M, Linde K, Stepan H, Kersting A. Obesity and anxiety during pregnancy and postpartum: A systematic review. J Affect Disord 2015;186:293-305.

Narayanan RP, Syed AA. Pregnancy Following Bariatric Surgery-Medical Complications and Management. Obes Surg 2016;26(10):2523-9.

Nwabueze PO, Abanobi OC, Nwankwo BO, Nwabueze AE. Occurrence of pregnancy-induced hypertension in selected health facilities in South East Nigeria. Int J Trop Med 2012;7(2):86-92. [PMID: http://docsdrive.com/pdfs/medwelljournals/ijtmed/2012/86-92.pdf]

Omar NL, Nor Aliza AG, Ismail MP, Zaki NM, Shukri OM. The factors associated with the incidence of hypertensive disorders among obese pregnant women in Hospital Universiti Sains Malaysia. Int Med J 2012;19(1):39-41.

Prathima P, Anuchitra S. Correlation between BMI and pregnancy outcome among postnatal mothers with pregnancy induced hypertension in selected hospitals Bangalore. Nitte Univ J Health Sci 2015;5(1):62-7. [PMID: http://nitte.edu.in/journal/december2014/CBBAP.pdf]

Reynolds RM. Maternal overweight and obesity in early pregnancy are associated with an increase in infant mortality risk. Evid -Based Med 2015;20(2):74

Sauvant P, Feart C, Atgie C. Vitamin A supply to mothers and children: Challenges and opportunities. Curr Opin Clin Nutr Metab Care 2012;15(3):310-4.

Seneviratne SN, Parry GK, McCowan LM, Ekeroma A, Jiang Y, Gusso S, et al. Antenatal exercise in overweight and obese women and its effects on offspring and maternal health: design and rationale of the IMPROVE (Improving Maternal and Progeny Obesity Via Exercise) randomised controlled trial. BMC Pregnancy Childbirth 2014;14:148

Steven S, Woodcock S, Small PK, Taylor R. Type 2 diabetes, bariatric surgery and the risk of subsequent gestational diabetes. Obstet med 2011;4(4):171-3.

Stotland NE. Obesity and pregnancy. BMJ (Online) 2009;338(7686):107-10. [PMID: http://www.bmj.com/cgi/content/full/337/dec15_1/a2450]

Sui Z, Dodd JM. Exercise in obese pregnant women: positive impacts and current perceptions. Int J Women Health 2013;5:389-98.

W.B. Sales,I.B.D. Nascimento,G. Dienstmann,M.L.R. Souza,G.D.D. Silva,J.C. Silva. Efetividade da metformina na prevencao do diabetes mellitus gestacional em gestantes obesas, Effectiveness of Metformin in the Prevention of Gestational Diabetes Mellitus in Obese Pregnant Women. Revista brasileira de ginecologia e obstetricia : revista da Federacao Brasileira das Sociedades de Ginecologia e Obstetricia. 2018///. 40:180

Wispelwey BP, Sheiner E. Cesarean delivery in obese women: a comprehensive review. J Matern Fetal Neonatal Med 2013;26(6):547-51.

Table S1: Summary of Eligibility Criteria from Included Studies

Study (Year)	Inclusion Criteria	Exclusion Criteria
Sen[13] (2016)	Women with a pre-pregnancy BMI (in kg/m2) ≥ 18.5 without pre-existing type 1 or 2 diabetes mellitus who completed first- and/or second-trimester Food Frequency Questionnaires (FFQs)	None reported
Davies-Tuck[14] (2016)	Singleton births ≥24 weeks gestation, free from congenital anomalies and seen at Monash Women's Services, Monash Health, a metropolitan maternity servicein Melbourne, Australia between 2009-2013. Only women who had a pre-gravid BMI at booking recorded were included.	Women of other nationalities were excluded
Marshall[15] (2014)	All live born singleton infants ≥37 weeks gestation born to African-American or Caucasian Missouri residents between 1 January 2000 and 31 December 2006.	Fetuses with major congenital anomalies and women with diabetes or chronic hypertension.
Machtinger[16] (2015)	All women with either spontaneous or IVF singleton pregnancies followed at the outpatient clinics of the hospital between January 2007 and December 2011 were included. IVF deliveries from women after undergoing IVF at the hospital and delivered at the hospital were also eligible, even if followed in other practices. Only those IVF pregnancies achieved following day 3 embryo transfer were included.	Pregnancies from day 5 transfers, multiple pregnancies, pregnancies with vanishing twins, cryopreservedcycles, oocyte donors and gestational carrier cycles were excluded.
Snowden[17] (2016)	All births occurring in California in 2007	American Indian/Alaska Native, multiracial, and other ethnicities were not included. Underweight women (BMI <18.5 kg/m2) were excluded from analysis. Records that were missing for maternal weight and maternal height were excluded.
Elkholi[18] (2014)	Obese (BMI ≥30) primigravidae with single pregnancy, with/without PCOS	Other etiologies of hyper-androgenism as late onset congenital adrenal hyperplasia, androgen secreting tumors of ovaries and adrenals and Cushing's syndrome.
Parker[19] (2015)	All records that contained singleton-delivery related discharges. In order to remove obesity as a potential confounder, the study population was further narrowed to include only obese women (BMI 30 kg/m^2) in the singleton delivery population.	Hospitalizations for conditions other than childbirth were excluded.
Persson[20] (2012)	Infants from singleton pregnancies and born to mothers with type 1 diabetes mellitus in Sweden between 1998 and 2007 were included. Type 1 diabetic pregnancies were identified based on ICD-10 code O240 and ICD-9code 250 for pre-gestational diabetes.	Of all pregnancies, 441 records (0.05%) with extreme values on maternal age, maternal weight or height were excluded. We excluded 90 records. The reference population included 764,498 singleton pregnancies to mothers without a diagnosis of diabetes, excluding 28,018 records from multiple pregnancies and 147,835 records with missing data on maternal BMI or BMI <18.5. In women with T1DM, 652 records (15%) were excluded due to missing data on maternal BMI or BMI <18.5 and 116 (2.8%) due to multiple pregnancies.

Study (Year)	Inclusion Criteria	Exclusion Criteria
Lamminpaa[21] (2016)	The study population of the current study included women whose pre-pregnancy BMI had been recorded in the data (N = 249,650). The data included both nulliparous and multiparous women with singleton births.	Cases with major congenital anomalies were excluded (N = 9354) as well as women with multiple pregnancies (N = 8472).
Metsälä (2015)	Not reported	Not reported
Houde[23] (2015)	A cohort was constructed using data for 2012 and selected from a database of all records for the contiguous United States, Hawaii, and Alaska. The analysis was restricted to singleton pregnancies with cephalic presentation at delivery, for which maternal age, pre-pregnancy BMI, and gestational weight gain information were available.	The territories Puerto Rico, Guam, and the Virgin Islands were excluded from the final aggregate file because of coding differences related to birth certificates. All entries with multiple pregnancies, recorded mal-placentation, mal-presentation, congenital anomalies, chromosomal abnormalities, and/or missing information on age, BMI, and gestational weight gain were also excluded.
Masho[24] (2012)	Singleton, live-born infants with no severe congenital abnormalities were included.	Multiple births, missing plurality, severe congenital anomalies, non-live births, including abortion, molar pregnancies, stillbirths and unknown etiology fetal deaths. Additionally mother-child pairs with missing data for: pre-pregnancy BMI, gestational weight gain, labor or gestational age.
Kim[38] (2013)	California resident women aged 20 years or older with a live singleton birth between 2007-2009 whose hospital delivery records were linked to the birth certificate.	Age < 20 years, multiple birth, pre-existing diabetes, missing race/ethnicity, missing BMI.
Ducarme[26] (2013)	Women who became pregnant after LAGBor Roux-en-Y gastric bypass (all singleton pregnancies)	Intra uterine death and fetal loss before 22 weeks.
Halloran[27] (2012)	Nulliparous, singleton live birth, Black or White ethnicity, aged 11-17 years.	Major congenital anomalies, missing BMI data.
Louis[28] (2012)	Obese (pre-pregnancy BMI ≥30 kg/m2), age ≥18 years and willing to be adherent with the study protocol.	Chronic use of narcotic or other drugs affecting the central nervous system, and inability to maintain sleep beyond 2 hours. Women with a documented history of non-adherence (missing >3 clinic visits) were also excluded.
Hedderson[29] (2012)	No pre-gravid diabetes, aged 15-45 years, delivery matched to California birth certificate, first delivery in study period	Missing plasma glucose values, height, weight, race/ethnicity (or 'other' race/ethnicity), parity, gestation at weight ascertainment
Olivarez[30](2011)	Inclusion criteria were gravidae of 18 to 50 years of age, with confirmed viable singleton gestations, in the first trimester of pregnancy.	The only exclusion criteria were subjects with known sleep-disordered breathing and women with significant underlying pulmonary or cardiac comorbidities, or with known multiple gestations.
Salihu[31](2010)	The study population initially consisted of 767,130 live-born singleton infants	27,118 (3.5%) were excluded because they did not fall within the gestational age range of 20-44 weeks. Further exclusions were made for missing or implausible weight/height values (73,178 (9.5%)) and for mothers with missing parity status (4,123 (0.5%)).
Aliyu[32] (2010)	Not reported	Criteria were not explicitly reported (excluded population described: from an available study population of 917,449 live

Study (Year)	Inclusion Criteria	Exclusion Criteria
		births, the following were sequentially excluded: 2542 (0.28%) births outside the range of 20–44 weeks of gestation; 28,727 (3.4%) multiple gestations; 92,006 (10.4%) records with missing information on maternal pre-pregnancy weight; 30,798 (3.9%) mothers who were not white, black, or Hispanic; and 451,954 (59.2%) mothers who were outside the ages of 20–24 or 35 and older)
Aliyu[33] (2010)	For analysis, data were restricted to include singleton births, women<25 years of age, deliveries that occurred between 20 and 44 weeks of gestation, and women who were non-Hispanic black, non-Hispanic white, and Hispanic.	Records were excluded if they were missing maternal pre-pregnancy BMI or information regarding pre-eclampsia or eclampsia. Approximately 11.6% of records were excluded because of these reasons.
Shachar[34] (2015)	Inclusion criteria were linked, singleton, live births, born 20-41 weeks of gestation to non-Hispanic white, non-Hispanic black, Hispanic, and Asian mothers.	Exclusion criteria were gestational week at delivery<20 or>41 (often resulting from erroneous gestational dating), missing education, maternal age <13 or >55 years, height <53 or >77 inches (these height cutoffs reflect at least 3 SD for any race/ethnicity), pre-pregnancy weight <75 or >450 pounds, and parity missing or >10.
Salihu[35] (2009)	Singleton births that were 20 to 44 weeks of gestation and women who were black, white, or Hispanic.	Records were excluded if they were missing maternal pre-pregnancy BMI
Thrift[36] (2014)	The study included 15 050 singleton births to Queensland resident Indigenous women and 250 798 to non-Indigenous women between 1 July 2007 and 31December 2011	Not repoted
Barton[37] (2014)	Nulliparous women aged 20 to 29 or 40 years who delivered at > 20 weeks' gestation.	Women reporting heart disease, chronic hypertension, pre-gestational diabetes, asthma, hypo- or hyperthyroidism thrombophilia, tobacco use during pregnancy, and conception with any assistive reproductive technology including ovulation induction were excluded.
Kim[38] (2014)	Women who self-reported as non-Hispanic Black or Non-Hispanic White	Women with missing information on race/ethnicity, with more than one race/ethnicity, or who were Hispanic were excluded
Reeske[39] (2012)	Singleton pregnancies	miscarriages, foetal deaths, stillborn infants, extrauterine pregnancies, hydatiform moles, more than one pregnancy or settlement for one woman in the period 2005–2007, multiple pregnancies (n = 44), mother older than 52 or younger than 15 at the time of birth, or age missing, inconclusive diagnosis of gestational diabetes
Lynch[40] (2012)	Singleton gestations	Women with a loss to follow-up (n=49) and chronic medical disease (cardiac disease, chronic hypertension, type 1 diabetes, and autoimmune disease, n =114). Women with a missing plasma sample (because of a deviation from the study protocol at the initial blood draw, n = 48) were also removed from the analysis.

Study (Year)	Inclusion Criteria	Exclusion Criteria
Belogolovkin [41] (2012)	Women with a singleton birth within the gestational age of 20–44 weeks were selected. The dataset includes all institutions in the state of Florida, except for military and Veterans Administration institutions.	During the period of the study, 11.5% of the records did not have a known maternal social security number, mainly because the mothers were not legal residents. These records were excluded from the linkage process since their follow-up would not be possible. Military and Veterans Administration institutions.
Hogh[42] (2020)	Women presenting for nuchal translucency US examination from 16 sept 2012 to 31 oct 2016	Women who miscarried before completing the questionnaire, multiple pregnancies, women with chronic diseases (kidney disease, diabetes 1 and 2, chronic hypertension prior to pregnancy), women without information on multivitamin use, women who only reported preconception multivitamin use
Njagu[43] (2020)	New patient obstetrical visit prior to 14+0 weeks GA with a recorded baseline BMI of 40+ who delivered at term (>37+0 weeks)	Multiple gestations, preterm delivery, fetal anomalies, intrauterine fetal demise; missing data related to maternal weight, height, or delivery timings
Malik[44] (2020)	Patients who underwent either laparascopic adjustable gastric band, laparoscopic sleeve gastrectomy (LSG), or laparoscpic Roux-en-Y gastric bypass (LRygB) duering the study period from January 2010 to May 2019	Not reported
Porteous[45] (2020)	Pregnant women at least 18years old who were birthing at Logan Hospital, who had an estimated date of confinement between 1 January 2012 and 13 February 2018 and who has a self-reported pregnancy weight and measured height that calculated to a BMI 30+ as documented in peri-natal data collection records	All other women who did not meet inclusion criteria. If eligible women had more than 1 pregnancy, the first pregnancy that resulted in a referral to the dietician was included, all others were excluded.
Pratt[46] (2019)	Women with singleton pregnancies 20+ weeks gestation delivering between 1 January 2011 and 31 December 2016	Women with multiple pregnancies, those with missing BMI data. Women weighing >180kg had their care transferred to a tertiary centre as per established health service capacity criteria
Dolin[47] (2019)	Women with singleton live births after bariatric surgery that received care between 2009-2017	Spontaneous abortion, elective abortion, multifetal gestation, incomplete medical records
Browne[48] (2019)	Singleton births between 24 weeks - days and 41 week 6 days weeks of gestation based on best clinical estimate of gestational age	Multifetal gesetations, pregnancies outside of the gestational age range, and those complicated by severe fetal anomalied (including anencephaly, spina bifida, cardiac anomalies, diaphragmatic hernia, gastroschisis, omphalocele, and chromosomal abnormalities). Additionally, maternal weight less than 70lbs were excluded as extremely underweight. We further excluded GDM, chronic hypertension and gestational hypertension, so as to not artifically increase the baseline risk of stillbirth when assessing for the effects of PGDM and obesity.
Ijas[49] (2019)	Women with a singleton pregnancy and OGTT performed during pregnancy in 2009	Not reported
Karadag[50] (2020)	Women who had undergone laparascopic sleeve gastrectomy (LSG) and goteen pregnant between 2010 and 2019. Patients with BMI >30 kg/m2 at the beginning of pregnancy and who had delivered in our clinic were observed retrospectively as the control group	Not reported

Study (Year)	Inclusion Criteria	Exclusion Criteria
Ram[51] (2019)	Not reported	Birth before 24+0 weeks of gestation, maternal pre-existing medical conditions (including chronic hypertension, pre-gestational diabetes mellitus, renal disease, and autoimmune disorders), monochorionic twins complicated by twin-to-twin transfusion syndrome (TTTS), monoamniotic twins, higher order multifetal pregnancies, reduction/termination of one or both fetuses, missing pre-pregnancy BMI, or pregnancies complicated by genetic or structural fetal anomalies.
Meghelli[52] (2020)	Not reported	Multiple pregnancy, pregestational diabetes mellitus or chronic hypertension, fetal malformations and medical termination of pregnancy
Fallatah[53] (2019)	Obese pregnant women attending King Abdulaziz University Hosptial (KAUH), Jaddah, KSA from January 2013 to December 2018	Pregnant women with BMI less than 30 and missing vitamin D level
Bar-Zeev[54] (2020)	Singleton pregnancies from 2009 to 2015	Not reported
Kong[55] (2019)	All pregnancies ending in live births in Finland between January 1, 2004 and December 31, 2014	Not reported
Ukah[56] (2019)	Singleton births between 20 and 45 weeks gestation that occurred in Washington State hospitals from 1 January 2004 to 31 December 2013	Not reported
Feghali[57] (2019)	Overweight and obese women with ginsletone pregnancies who delivered from October 1, 2012 until August 20, 2014. We included women who had at least one measured weight between 24 and 28 weeks of gestation and documented prenatal care in the general obstetrics, midwifery, and maternal–fetal medicine clinics in our hospital system. Only the first delivery was included if women had multiple deliveries during the study period.	We excluded women who did not have pre- natal care documented in the outpatient record because the majority of these women were transferred to our facility for acute specialty care and were there- fore a unique, high-risk population. We also excluded women who did not have either a self-reported pre- pregnancy weight or a measured weight before 13 weeks of gestation and women with pregestational diabetes.
Roussel[58] (2019)	Singleton pregnancy, term delivery (between 37 and 41 weeks of gestation), and a BMI comprised between 35-40 kg/m2 beginning on 1st of January 2006 and ending on the 31st of December 2015	Weight gain exceeded 9 kg, if the last weight measure was recorded more than a week preceding delivery and in cases of incomplete/missing data
Nowak[59] (2019)	Singleton pregnancy, no maternal chronic diseases, no congenital fetal abnormalities	Not reported
Thompson[60] (2019)	Singleton births between 2014-2016	Not reported
Benjamin[61] (2019)	Infants born 2006-2012 with a livebirth of a sibling born in 2005-2012 to Texas residents. The birth certificate for the index infant had to link to at least one older liveborn sibling. When an infant was lnked to more than one older sibling, only the linked sibling with the date of birth closest to the index infant was retained	Index infant was from a multiple birth, information on maternal prepregnancy weight was missing from either birth certificate, or maternal height on the index infant and sibling birth certificates differed by more than 2 inches
Thagaard[62] (2019)	Women who had a serum sample from the first trimester stored frozen at the Danish National Biobank at States Serum Institut (SSI), Copenhagen, Denmark between January 2006 to December 2011	Underweight and overweight women

Study (Year)	Inclusion Criteria	Exclusion Criteria
Grove[63] (2019)	Women who have their first and second or second and third consecutive live-born singleton pregnancies within the dataset from 2003 to 2018	Women missing key data (weight or height at booking, or gestation at birth) or who had inconsistent or outlying values for key data that were likely to be errors were excluded from the analysis. In order to mitigate for inaccurate self-reported heights, women who lost height between pregnancies (>5cm), and those who gained height (>5 cm and were over the age of 22 at first pregnancy), were excluded from the analysis. Women who were booked after 24 weeks gestation were excluded, as the study centre is a specialist centre, and some of these women booking later may have been high risk pregnancies referred from another unit. Multiple births were excluded due to their strong association with PB [10]. Women with multiple miscarriages were also excluded as these women are likely to have different risk factors to the general population
Shaukat[64] (2019)	Nulliparous women with singleton pregnancies who had their first antenatal visit at a PHCC facility between 1 June 2016 and 1 March 2017 and their pregnancy outcome at a HMB facility before 10 November 2017	Women with high-risk pregnancy, that is, those with a pregnancy outcome prior to 24 weeks of gestation (n=8), those who were obese class II and higher (BMI⩾35kg/m2, n=80) or who were under the age of 18 at their first antenatal visit (n=23). Women who gave birth to babies with indeterminate sex (n=2), or who experi-enced stillbirth (n=3), fetal death (n=4) or neonatal death (n=1) were also excluded
Moore Simas[65] (2019)	Women who self-identified as Latinas, were ages 16049 years, without heart disease, chronic kidney disease, pregestational diabetes and/or chornic hypertension and who delivered singleton gestations between 2006 and 2011	Women missing prepregnancy height or who were not screened for GDM due to having a spontaneous or therapeutic abortion prior to GDM screen or no longer receiving prenatal care at the study hospitals
Frankenthal[66] (2019)	Women with singleton pregnancies recruited between June 2006 and December 2008	Not reported
Laine[67] (2019)	Women with singleton pregnancies who gave birth between 1 January 2009 and 31 December 2015 who have not previously been diagnosed with diabetes mellitus	Not reported
Boudet-Berquier[68] (2017)	Age over 18, not institutionalized, able to speak, read or write French or to get help from someone who did. The newborn had to be born at 33 amenorrhea weeks (AW) or later, without severe path- ology requiring hospitalization, and had not been transferred to a unit other than the maternity ward in the days following birth. Between January and April 2012.	Not reported
Janevic[69] (2018)	Births between 2010 to 2014	Women with pregestational diabetes, women with multiple gestations, records with missing values for BMI and maternal education, women with race-ethnicity categorized as "other"
Anderson[70] (2016)	Singleton first births from 2009-2013	Multiple births; ethnicity other than American Indian, Alaska Native, non-Hispanic white, non-Hispanic African American, or Hispanic

Study (Year)	Inclusion Criteria	Exclusion Criteria
Zamora-Kapoor[71] (2016)	Singleton live births to American Indian/Alaskan Native women and a randomly selected 10:1 comparison group of all other women with deliveries, frequency matched by delivery year from 2003-2013. Women with singleton pregnancies without prior live births	Not reported
Gernand[72] (2014)	Women with singletons pregnancies from 1991-1995 with a serum sample at 26 weeks of gestation or less available for vitamin D assessment	Stillbirths, pregnancies missing birth weight, pregnancies missing pre-pregnancy body mass index
Subramaniam[73] (2015)	Singleton pregnancies delivered from 2000 to 2010 who had a glucose challenge test (GCT) performed after 20 weeks' gestation and had a documented BMI at entry to care	Multifetal gestations, delivery before 20 weeks, fetal anomalies, and maternal medical comorbidies (including but not limited to pregestational diabetes, cardiac disease, HIV, hypertension, autoimmune disease, and placenta pre- via). In addition, women with a GCT < 40 mg/dL were excluded as this was likely due to laboratory error; women with a GCT > 200 mg/dL were also excluded as these women did not undergo confirmatory GTT. Only the first pregnancy during the ascertainment period was considered.)

The distributions of means and ranges of maternal pre-pregnancy body mass index and maternal age across exposure groups in each study are shown below. In studies where there existed sub-populations of both obese and non-obese women and demographic measures were unavailable for the subgroup of obese women, distributions reported are based upon the full study population.

Table S2: Distribution of Maternal BMI and Age Amongst Included Studies

**Author (Year)**	**Total Study Size**	**# of Exposure Groups**	**Maternal Pre-Pregnancy BMI (range of means or medians across groups)**	**Maternal Age (range of means or medians across groups)**
Sen[13] (2016)	1,808*	4 groups (quartiles of DII)	24.1 to 25.8**	30.4 to 33.4**
Davies-Tuck[14] (2016)	6,038*	2 groups (Australian obese women, South Asian obese women)	Means not reported; All BMI >30 amongst the groups of obese women	Range 0.5%-2.7%<20 years; range 39.4%-44.3% 20-30 years; range 53.0%-60.1% >30 years
Marshall[15] (2014)	61,191*	2 groups (African American, Caucasian)	<18.5: range 4.3-5.5%; 18.5-24.9: range 43.0-52.9%; 25-29.9: range 22.5-25.6% ; 30-39.9:range 15.9-21.2% ; 40-49.9: range 2.9-5.0%; >50: range 0.3-0.9%**	<18: range 2.7-8.2%; 18-34: range 85.9%-86.4%, ≥35: range 5.9-10.9%**
Machtinger[16] (2015)	1,015*	2 groups (spontaneous versus IVF pregnancy)	30.1 to 65.2	18.4 to 44.3
Snowden[17] (2016)	76,174*	4 groups (White, Black, Hispanic, Asian American)	Range 6.0%-28.0% >BMI 30	Range 12.5-27.2% >35 years**
Elkholi[18] (2014)	400	4 groups (formed by simultaneous groupings by obesity type and adipocytokines)	Range 31.3 to 32.1	Range 22.4 to 23.4
Parker[19] (2015)	186,705	2 groups (gastric bypass versus no gastric bypass)	Means not reported; All BMI >30 amongst the groups of obese women	Range 28.4 to 33.0
Persson[20] (2012)	82,949*	2 groups (presence versus absence of type 1 diabetes history)	23.6 to 25.1**	Median 30 in both groups**
Lamminpaa[21] (2016)	29,995*	2 groups (<35 years versus >35 years)	Means not reported	Means not reported
Metsälä[22] (2015)	11,404*	4 groups (absence/presence of pre-existing diabetes, pre-existing hypertension)	Totals of 7%, 2% and 1% of entire population belonged to BMI groups of 30-34.9, 35-39.9 and >40	Means of 28 across groups**
Houde[23] (2015)	3,960,796 births*	2 groups (age 12-19, age>19)	Totals of 19.7%, 8.5% and 5.3% in BMI groups of 30-34.9, 35-39.9 and >40	Range of means 17.9-28.8**
Masho[24] (2012)	45,824	Maternal weight gain during pregnancy(quartiles)	Means not reported	24.1 to 28.1
Kim[25] (2013)	251,237*	Ethnicity (white, black, Asian, Hispanic, American Indian)	Means not reported; range 6.6% to 35.4% with BMI>30	Means not reported; range 12.2% to 27.3% with maternal age >35 years
Ducarme[26] (2013)	79 women (94 pregnancies)	Type of bariatric surgery (LAGB versus RYGB)	Mean 46.5 (range 27.2-54.5) across groups (data not provided by group)	Mean of 30.8 (range 22-45) across groups (data not provided by group)

Author (Year)	Total Study Size	# of Exposure Groups	Maternal Pre-Pregnancy BMI (range of means or medians across groups)	Maternal Age (range of means or medians across groups)
Halloran[27] (2012)	2,815*	Ethnicity (Caucasian versus African American)	Means not reported	Means not reported (study performed in teenagers <18 years of age)
Louis[28] (2012)	161	Obstructive sleep apnea (yes versus no)	39.1 to 48.3	27.3 to 30.0
Hedderson[29] (2012)	40,279**	Race (White, Hispanic, African American, Asian, Filipina)	Means not reported; range 7.1% to 35.1% with BMI>31	27.2 to 29.5
Olivarez[30] (2011)	50*	Obstructive sleep apnea (yes versus no)	35.2 to 35.3	28.3 to 30.2
Salihu[31] (2010)	132,894*	Nulliparity (nulliparious versus multiparous), race (white, black, hispanic)	Means not reported; range 15.73% to 22.37% with BMI>30	Means not reported; range 7.61% to 18.21% with maternal age $\geq$35 years
Aliyu[32] (2010)	3,278*	Maternal age (20-24 versus$\geq$35)	Means not reported; range 19.79% to 20.64% had BMI>30	Means not reported
Aliyu[33] (2010)	51,427*	Adolescent maternal age (<20 years versus 20-24 years)	Means not reported; range 12.2% to 19.8% with BMI>30	Means not reported (age is primary exposure of interest)
Shachar[34] (2015)	19,664*	Maternal height (five size categories considered)	Means not reported; range 4.6%-27.5% with BMI>30	Means not reported; range 9.7%-43.5% <25 years, range 12.8%-27.3% $\geq$35 years
Salihu[35] (2009)	149,532*	Ethnicity (Hispanic, Caucasian, African American)	Means not reported; 27.7% of the full study population had BMI>30	Means not reported; range 13.8% to 14.7%
Thrift[36] (2014)	55,275*	Indigenous status (indigenous versus non-indigenous)	Means not reported; ranges of 4.5%-6.31%, 36.6%-46.6%, 27.7%-27.8%, 18.2%-24.7% in BMI categories of <18.5, 18.5-25, 25-30, and >30	Data not reported
Barton[37] (2014)	9,452*	Maternal age (20-29 versus>40)	35.0 to 35.2	25.4 to 41.1
Kim[38] (2014)	462,296*	Ethnicity (Caucasian, African American), Age (20-29, >40)	Means not reported; range 17.2% to 27.5% with BMI>30	Means not reported; ranges of 8.7%-17.4%, 52.2%-58.7%, 22%-36.2% and 1.9%-2.9% in the age groups of <20 years, 20-29 years, 30-39 years and $\geq$40 years
Reeske[39] (2012)	3,338	Ethnicity (Turkish versus German)	Means not reported; range 15.9%-17.8% with BMI>30	Means not reported; range 28%-38% <25 years, range 46.5%-56.2% 25-34 years, range 15.5%-15.9% >34 years
Lynch[40] (2012)	1,013	Complement activation fragments (Bb, C3a; quartiles)	Means not reported; totals of 4%, 62%, 23% and 11% were underweight, normal weight, overweight and obese, respectively	Means not reported; 62% of participants were <35 years of age
Belogolovkin[41] (2012)	131,166*	Prior bariatric surgery (yes versus no)	Means not reported; range 19.96% to 48.81% with BMI>30	Means not reported; range 14.43% to 38.91% of advanced maternal age
Hogh[42] (2020)	15,154*	3 group (non-vitamin users, periconceptional multivitamin use, early pregnancy multivitamin use)	Means not reported; range 3.8% to 4.2% with BMI$\geq$30	Means not reported; ranges of 2.32%-3.69%, 24.4%-29%, 30.1%-41.7%, 20.8%-24.5% and 4.33%-6.97% in the age groups of

Author (Year)	Total Study Size	# of Exposure Groups	Maternal Pre-Pregnancy BMI (range of means or medians across groups)	Maternal Age (range of means or medians across groups)
				<25 years, 25-29 years, 30-34 years, 35-39 and >40 years
Njagu[43] (2020)	374*	2 groups (gained ≤20 lbs versus gained >20 lbs)	gained ≤20 lbs: 44.7 (41.6, 49.4)<br>gained >20 lbs: 43.7 (41.5, 48.0)	gained ≤20 lbs: 29.3 (25.4, 34.7)<br>gained >20 lbs: 30.0 (26.5, 33.6)
Malik[44] (2020)	55*	2 groups for postbariatric surgery (yes versus no)	Yes: 32.8<br>No: 40.8	Yes: 32.7<br>No: 31.4
Porteous[45] (2020)	5,426	5 groups for referral to an Ante-natal dietitian (not referred, referred, referred and attended ≥3 appointments, referred and attended 1-2 appointments, referred and attended none)	Not referred: 33.9<br>Referred: 40.6<br>Referred, attended ≥3 appointments: 40.8<br>Referred, attended 1-2 appointments: 40.2<br>Referred and attended none: 41.2	Not referred: 29<br>Referred: 29<br>Referred, attended ≥3 appointments: 29<br>Referred, attended 1-2 appointments: 29<br>Referred and attended none: 28
Pratt[46] (2019)	18,402*	5 groups of BMI class (<18.5, 18.5-24.9, 25-29.9, 30-49.9, ≥50)	Means not reported	<18.5: 26;<br>18.5-24.9: 28;<br>25-29.9: 29;<br>30-49.9: 29;<br>≥50: 31
Dolin[47] (2019)	76*	2 groups bariatric surgery (<12 months before versus ≥12 months before pregnancy)	<12 months before: 34.3<br>≥12 months before: 31.2	<12 months before: 31.5<br>≥12 months before: 32
Browne[48] (2019)	3,097,123*	5 groups of BMI class (<18.5, 18.5-24.9, 25-29.9, 30-49.9, ≥50)	Means not reported; ranges are<br>BMI <18.5: 45.4%<br>BMI 18.5 - <25: 50.2%<br>BMI 25 - <30: 24.9%<br>BMI >35: 17.3%<br>BMI >40: 3.12%	<18.5: 23;<br>18.5-24.9: 26;<br>25-29.9: 27;<br>30-39.9: 27;<br>≥40: 28
Ijas[49] (2019)	24,577*	2 groups (no GDM versus GDM)	No GDM: 24.3<br>GDM: 24.3	No GDM: 30<br>GDM: 30
Karadag[50] (2020)	144*	3 groups (pregnancies ≤1 year of LSG, >1 year after LSG, no LSG)	≤1 year: 32.83<br>>1 year: 28.8<br>no LSG: 31.05	≤1 year: 30.29<br>>1 year: 28.9<br>no LSG: 27.48
Ram[51] (2019)	487,870*	6 groups (underweight, normal weight, and overweight/obese singleton pregnancies versus underweight, normal weight, and overweight/obese twin pregnancies)	Underweight singleton: 17.3<br>Normal weight singleton: 21.9<br>Obese singleton: 30.7<br>Underweight twin: 17.3<br>normal weight twin: 22.0<br>Obese twin: 31.1	Underweight singleton: 29.0<br>Normal weight singleton: 30.4<br>Obese singleton: 30.7<br>Underweight twin: 31.1<br>normal weight twin: 32.2<br>Obese twin: 31.9
Meghelli[52] (2020)	472*	4 groups (no GDM, GDM, GDM with insulin, GDM no insulin)	No GDM: 43.5<br>GDM: 43.8<br>GDM with insulin: 43.6<br>GDM no insulin: 44.0	No GDM: 28.9<br>GDM: 30.4<br>GDM with insulin: 29<br>GDM no insulin: 31.6
Fallatah[53] (2019)	132*	4 groups vitamin D levels (deficient versus optimal versus therapeutic versus excess)	Means not reported; ranges are<br>BMI >30: 50.0-77.8%<br>BMI >35: 22.2-50.0%	Means not reported; ranges of 0%-23.2%, 0%-39.4%, 25.3%-100%, 0-18.2% and 0-5% in the age groups of <30 years, 30-34

Author (Year)	Total Study Size	# of Exposure Groups	Maternal Pre-Pregnancy BMI (range of means or medians across groups)	Maternal Age (range of means or medians across groups)
				years, 35-39, 40-44 and 45-50 years
Bar-Zeev[54] (2020)	222,408*	5 groups prenatal smoking (non-smoker, quit smoking before pregnancy, quit smoking during pregnancy, reduced the amount smoked, smoked the same or more)	Means not reported	Means not reported
Kong[55] (2019)	649,043*	4 groups prematurity diabetes (No diabetes, pregestational diabetes treated with insulin, pregestational type II diabetes not treated with insulin, GDM)	Means not reported; ranges are<br>BMI <18.5: 0.8-4%<br>BMI 18.5 - <25: 24.0-64.3%<br>BMI 25 - <30: 18.7-31.3%<br>BMI >30: 5.7-21.5%<br>BMI >35: 2.0-25.2%	Means not reported; ranges are<br><20: 1.4-2.5%<br>20-24: 10.9-16.1%<br>25-30: 25.4-32.1%<br>30-34: 30.5-32.5%<br>35-55: 17.8-30.7%
Ukah[56] (2019)	165,908*	3 groups on GWG in obese women (optimal, low, excess weight gain)	Means not reported	Means not reported; ranges are<br>15-19: 4.0-6.0%<br>20-24: 21.7-24.7%<br>25-30: 30.5-31.2%<br>30-34: 24.21-25.9%<br>35-39: 11.6-13.7%<br>≥40: 2.7-3.6%
Feghali[57] (2019)	5,814*	3 groups on GWG in obese women at 24-28 weeks (adequate, inadequate, excess)	Adequate: 31.4<br>Inadequate: 34.4<br>Excess: 30.4	Adequate: 29.5<br>Inadequate: 28.9<br>Excess: 29.8
Roussel[58] (2019)	996*	3 groups on GWG in obese women (recommended weight gain, low weight gain, weight loss)	Recommended weight gain: 36.88<br>Low weight gain: 37.17<br>Weight loss: 37.44	Recommended weight gain: 29.24<br>Low weight gain: 28.94<br>Weight loss: 29.48
Nowak[59] (2019)	474*	4 groups on GWG based on BMI (underweight, normal, overweight, obese)	Underweight: 9.07%<br>Normal: 66.0%<br>Overweight: 19.2%<br>Obese: 5.70%	Underweight: 30.14<br>Normal: 31.79<br>Overweight: 32.62<br>Obese: 31.31
Thompson[60] (2019)	10,811,496	3 groups on GWG (<5 kg, 6-9 kg, >9 kg),	Means not reported; ranges are<br>BMI <18.5: 1.1-1.5%<br>BMI 18.5 - <25: 11.3-19.5%<br>BMI 25 - <30: 3.6-16.6%<br>BMI >30: 2.2-9.4%<br>BMI >35: 1.3-3.8%<br>BMI >40: 0.9-2.2%	Means not reported
Benjamin[61] (2019)	694	3 groups on interpregnancy BMI changes (LGA, SGA, preterm birth)	LGA: 27.7<br>SGA: 25.4<br>Preterm birth: 25.2	Means not reported; ranges are<br><20: 6.3-8.2%<br>20-24: 17.9-33.7%<br>25-30: 24.3-36.2%<br>30-34: 17.1-25.0%<br>>35: 8.0-15.8%
Thagaard[62] (2019)	2,503	3 groups on concentrations of adiponectin and leptin biomarker (unaffected, hypertension, preeclampsia)	Means not reported; ranges are<br>BMI <18.5: 21.9-51.2%<br>BMI >30: 33.2-54.7%<br>BMI >35: 15.6-24.1%	Unaffected: 29.3<br>Hypertension: 29.2<br>Preeclampsia: 28.2

Author (Year)	Total Study Size	# of Exposure Groups	Maternal Pre-Pregnancy BMI (range of means or medians across groups)	Maternal Age (range of means or medians across groups)
Grove[63] (2019)	20,069	4 groups on GWG in 2nd and 3rd pregnancies (term birth in 2nd birth, preterm birth in 2nd birth, term birth in 3rd birth, preterm birth in 3rd birth)	Term birth in 2nd birth: 23.5<br>Preterm birth in 2nd birth: 23.7<br>Term birth in 3rd birth: 24.2<br>Preterm birth in 3rd birth: 23.9	Term birth in 2nd birth: 29<br>Preterm birth in 2nd birth: 29<br>Term birth in 3rd birth: 29<br>Preterm birth in 3rd birth: 29
Shaukat[64] (2019)	1,134	4 groups on different outcomes in Arab versus non-Arab (underweight, overweight, obese, missing BMI)	Underweight: 35.63%<br>Overweight: 35.19%<br>Obese: 20.28%<br>Missing BMI info: 8.91%	Means not reported; ranges are<br>18-24: 31.7-52.48%<br>25-30: 38.86-49.57%<br>31-44: 8.66-18.7%
Moore Simas[65] (2019)	2.039	2 groups on GWG, GDM, AGT among Latinas (Proyecto Buena Salud data set and University of Massachusetts Memorial Health Care data set)	Means not reported; ranges are<br>BMI <18.5: 5.1-6.1%<br>BMI 18.5 - <25: 41.2-49.2%<br>BMI 25 - <30: 22.8-25.4%<br>BMI >30: 22.0-28.3%	PBS: 22.7<br>UMMHC: 25.9
Frankenthal[66] (2019)	1,058	2 groups on GWG (assisted reproduction treatment versus spontaneous conception)	Means not reported; ranges are<br>BMI <18.5: 7.5-8.5%<br>BMI 18.5 - <25: 63.7-63.7%<br>BMI 25 - <30: 16.0-17.1%<br>BMI >30: 7.6-11.7%	Assisted reproduction treatment: 33<br>Spontaneous conception: 30.7
Laine[67] (2019)	6,920	2 groups on antidepressant use in pregnancies (non-user versus user)	Means not reported; ranges are<br>BMI <18.5: 2.9-4.1%<br>BMI 18.5 - <25: 56.3-67%<br>BMI 25 - <30: 19.1-24.2%<br>BMI >30: 9.8-16.6%	Non-user: 28.3<br>User: 27.9
Boudet-Berquier[68] (2017)	3,208	4 groups on parity (underweight, normal weight, overweight, obese)	Underweight: 7.48%<br>Normal weight: 64.4%<br>Overweight: 18.2%<br>Obese: 9.91%	Means not reported; ranges are<br>18-24: 15.9-28.6%<br>25-29: 32.1-34.5%<br>30-34: 22.9-33.0%<br>≥35: 10.7-25.7%
Janevic[69] (2018)	668,035	14 groups on different outcomes in ethnicities and places of birth (Black US born, Black foreign born, White US born, White foreign born, all Hispanic US born, all Hispanic foreign born, all Asian US born, all Asian foreign born, Mexican US born, Mexican foreign born, Chinese US born, Chinese foreign born, Indian US born, Indian foreign born)	Means not reported; ranges are<br>BMI <18.5: 2.30-17.20%<br>BMI 18.5 - <25: 37.5-74.60%<br>BMI 25 - <30: 7.90-34.50%<br>BMI >30: 1.00-16.80%<br>BMI >35: 0.20-8.30%<br>BMI >40: 0.00-6.10%	Black US born: 27.1<br>Black foreign born: 30.8<br>White US born: 26.6<br>White foreign born: 28.6<br>All Hispanic US born: 31.9<br>All Hispanic foreign born: 30.2<br>All Asian US born: 31.3<br>All Asian foreign born: 31.1<br>Mexican US born: 23.3<br>Mexican foreign born: 28.3<br>Chinese US born: 30.7<br>Chinese foreign born: 29.3<br>Indian US born: 32.6<br>Indian foreign born: 30.1
Anderson[70] (2016)	5,193,386	4 groups on different outcomes in ethnicities (American Indian/Alaska Native, Black, White, Hispanic)	Means not reported; ranges are<br>BMI <18.5: 4.3-4.7%<br>BMI 18.5 - <25: 43.4-53.4%<br>BMI 25 - <30: 22.9-25.3%<br>BMI >30: 19.1-26.8%	Means not reported; ranges are<br><15: 0.1-0.6%<br>15-19: 12.6-32.7%<br>20-24: 27.9-39.3%<br>25-29: 16.9-30.2%

Author (Year)	Total Study Size	# of Exposure Groups	Maternal Pre-Pregnancy BMI (range of means or medians across groups)	Maternal Age (range of means or medians across groups)
				30-34: 7.4-20.6%<br>35-39: 2.6-7.0%<br>40-44: 0.5-1.5%<br>>45: 0.0-0.1%
Zamora-Kapoor[71] (2016)	71,080	2 groups on different outcomes in ethnicities (American Indian/Alaska Native versus White)	Means not reported; ranges are<br>BMI <18.5: 2.7-3.5%<br>BMI 18.5 - <25: 41.4-52.1%<br>BMI 25 - <30: 24.5-27.6%<br>BMI >30: 19.9-28.3%	Means not reported; ranges are<br>12-19: 13.5-37.1%<br>20-24: 29.3- 37.7%<br>25-29: 15.3-28.0%<br>30-34: 6.8-19.6%<br>35-55: 3.1-9.6%
Gernand[72] (2014)	792	2 groups on Vitamin D status (non-SGA versus SGA)	Means not reported; ranges are<br>BMI 18.5 - <25: 32.5-34%<br>BMI 25 - <30: %<br>BMI >30: 22.5-29.1%<br>BMI >35: 22.3-24.7%	Means not reported; ranges are<br><15: 12.1-12.6%<br>15-19: 55.7-58.3%<br>20-24: 29.1-31.4%
Subramaniam[73] (2015)	14,525	6 groups on 1-hour glucose challenge test results (GCT <120, GCT 120-129, GCT 130-134, GCT 135-139, GCT 140-144, GCT 145-199)	GCT <120: 25<br>GCT 120-129: 26<br>GCT 130-134: 26<br>GCT 135-139: 27<br>GCT 140-144: 27<br>GCT 145-199: 28	GCT <120: 21<br>GCT 120-129: 23<br>GCT 130-134: 23<br>GCT 135-139: 23<br>GCT 140-144: 24<br>GCT 145-199: 25

KEY:DII=dietary inflammatory index; GWG=gestational weight gain; IVF=in-vitro fertilization; LAGB=laparoscopic adjustable gastric bypass; PC=prospective cohort; PCOS=polycystic ovary syndrome; RC=retrospective cohort; RYGB=roux-en-Y gastric bypass; SES=socio-economic status; LSG=laparoscopic sleeve gastrectomy; LGA=large gestational age; AGT=abnormal glucose tolerance.

*denotes cohort included both obese and non-obese participants

Overview of Adjustments for Covariates Across Studies

Study (Year)	Adj?	Covariates Accounted for In Studies Where Multivariable Analysis was Presented															
		Maternal age	Maternal ethnicity	Maternal BMI/ obesity status	Maternal SES	Maternal education	Marital status	Maternal height	Presence or adequacy of prenatal care	History of diabetes	History of smoking	History of alcohol	Parity	Mode of delivery	Gestational age	History of pregnancy complications (PE, GDM, etc)	Other Characteristics Noted
Sen[13] (2016)	Y	X	X	X		X					X		X				Household income
Davies-Tuck[14] (2016)	N																
Marshall[15] (2014)	Y	X				X	X		X		X		X				History of caesarean section, infant gender, Medicaid status
Machtinger[16] (2015)	Y	X	X							X	X		X				Hypertension
Snowden[17] (2016)	Y	X				X			X				X				Insurance status
Elkholi[18] (2014)	N																
Parker[19] (2015)	Y	X	X							X	X						Hypertension
Persson[20] (2012)	Y	X	X					X			X		X				Hypertension
Lamminpaa[21] (2016)	N																
Metsälä[22] (2015)	Y	X															Socioeconomic status
Houde[23] (2015)	Y		X			X	X					X		X			Comorbidities, birth weight
Masho[24] (2012)	N																
Kim[25] (2013)	Y	X	X										X				
Ducarme[26] (2013)	N																
Halloran[27] (2012)	N																
Louis[28] (2012)	Y	X	X	X													
Hedderson[29] (2012)	Y	X				X							X		X		
Olivarez[30] (2011)	N																
Salihu[31] (2010)	Y	X	X	X		X	X		X		X						

Study (Year)	Adj?	Covariates Accounted for In Studies Where Multivariable Analysis was Presented															
		Maternal age	Maternal ethnicity	Maternal BMI/ obesity status	Maternal SES	Maternal education	Marital status	Maternal height	Presence or adequacy of prenatal care	History of diabetes	History of smoking	History of alcohol	Parity	Mode of delivery	Gestational age	History of pregnancy complications (PE, GDM, etc)	Other Characteristics Noted
Aliyu[32] (2010)	Y		X			X	X		X		X	X	X			X	
Aliyu[33] (2010)	Y					X	X		X		X	X	X			X	
Shachar[34] (2015)	Y	X				X			X	X	X		X			X	Payor
Salihu[35] (2009)	Y	X				X	X		X	X	X	X	X			X	Anemia, infection, hypertension
Thrift[36] (2014)	Y	X									X		X				Remoteness Index of Australia category
Barton[37] (2014)	N																
Kim[38] (2014)	Y	X	X	X												X	Enrollment in Women, Infants and Children's Program
Reeske[39] (2012)	Y	X															
Lynch[40] (2012)	Y	X	X								X		X				
Belogolovkin[41] (2012)	Y	X	X	X		X	X				X		X				Event year
Hogh[42] (2020)	Y	X	X	X		X					X		X	X			Exercise
Njagu[43] (2020)	N	X	X	X					X	X	X		X	X	X	X	Private insurance, drug use, chronic hypertension, depression
Malik[44] (2020)	N	X		X						X			X		X	X	Hypertension, hyperlipidemia, asthma,
Porteous[45] (2020)	N	X		X	X		X		X		X		X	X	X	X	Induction, macrosomia, maternal birth country, Indigenous status
Pratt[46] (2019)	Y	X		X									X	X	X	X	Indigenous status, birth country
Dolin[47] (2019)	N	X	X	X					X				X	X	X	X	Gestational weight gain, bariatric procedure type
Browne[48] (2019)	Y	X	X	X		X			X	X	X		X		X	X	
Ijas[49] (2019)	Y	X		X	X						X		X	X		X	
Karadag[50]	N	X		X									X	X	X	X	Gravidity, abortion

Study (Year)	Adj?	Covariates Accounted for In Studies Where Multivariable Analysis was Presented															
		Maternal age	Maternal ethnicity	Maternal BMI/ obesity status	Maternal SES	Maternal education	Marital status	Maternal height	Presence or adequacy of prenatal care	History of diabetes	History of smoking	History of alcohol	Parity	Mode of delivery	Gestational age	History of pregnancy complications (PE, GDM, etc)	Other Characteristics Noted
(2020)																	
Ram[51] (2019)	Y	X	X	X				X			X		X	X	X	X	Pre-pregnancy weight, fertility treatments
Meghelli[52] (2020)	N	X		X							X		X	X	X	X	hospitalization
Fallatah[53] (2019)	N	X		X						X			X		X	X	Gravidity, nationality,
Bar-Zeev[54] (2020)	Y	X	X	X							X		X			X	Gestational weight gain, hypertension
Kong[55] (2019)	Y	X		X						X	X		X	X	X	X	Birth year, country of birth
Ukah[56] (2019)	N	X	X	X		X	X			X	X		X	X	X	X	Insurance, assisted conception, hypertension
Feghali[57] (2019)	N	X	X	X							X		X	X	X	X	Insurance, hypertension, glucose levels, gestational weight gain
Roussel[58] (2019)	Y	X		X			X				X		X	X	X	X	French origin, occupation, chronic hypertension
Nowak[59] (2019)	N	X		X									X		X		Gestational weight gain, pregnancy length, neonatal birth weight
Thompson[60] (2019)	N			X										X		X	
Benjamin[61] (2019)	Y	X	X	X		X	X	X		X	X				X	X	Insurance, hypertension, gestational weight gain
Thagaard[62] (2019)	N	X	X	X							X		X		X	X	Conception
Grove[63] (2019)	Y	X	X	X		X					X			X	X	X	Employment, fertility treatment, gender of infant
Shaukat[64] (2019)	Y	X	X	X						X				X	X	X	Hypertension, thyroid condition
Moore Simas[65] (2019)	Y	X		X					X	X	X				X	X	Gravidity, language preference, gestational weight gain
Frankenthal[66] (2019)	N	X		X		X							X	X	X	X	Gestational weight gain, hypertension
Laine[67] (2019)	Y	X		X		X	X	X			X		X	X		X	Weight, fertility treatment, hospitalization
Boudet-Berquier[68]	Y	X		X		X	X		X		X	X	X	X	X	X	Country of birth, occupation, time of return to work,

Study (Year)	Adj?	Covariates Accounted for In Studies Where Multivariable Analysis was Presented															
		Maternal age	Maternal ethnicity	Maternal BMI/ obesity status	Maternal SES	Maternal education	Marital status	Maternal height	Presence or adequacy of prenatal care	History of diabetes	History of smoking	History of alcohol	Parity	Mode of delivery	Gestational age	History of pregnancy complications (PE, GDM, etc)	Other Characteristics Noted
(2017)																	gestational weight gain, infant sex/weight
Janevic[69] (2018)	Y	X	X	X		X		X					X				Insurance
Anderson[70] (2016)	Y	X	X	X		X	X		X	X						X	Child's sex
Zamora-Kapoor[71] (2016)	Y	X	X	X		X	X				X						Insurance; Women, Infants, and Children (WIC) participation
Gernand[72] (2014)	Y	X	X	X		X	X			X	X		X		X	X	Season of blood sampling, latitude of study site, chronic hypertension, neonatal health
Subramaniam[73] (2015)	Y	X	X	X							X	X			X	X	Street drugs use, glucose levels, infant sex,

File S4: Deviations from Study Protocol

During the process of this review, certain modifications to the a priori protocol took place. These were as follows:

- ***Regarding Study Assessments***. Our protocol indicated we would consider risk of bias evaluations if sufficient resources were available. Due to resource constraints, evaluations were not pursued. The majority of evidence is considered by the research team to be at an increased risk of selection bias due to the non-randomized nature of the included studies as well as the common lack of availability of detailed demographic data for women with obesity women within studies that also enrolled women with underweight, recommended weight and overweight women BMIs. Given the exploratory nature of this review, the team feels there is strong value in a summary of all the evidence located.

- ***Regarding Outcomes Assessed***. During full text screening, due to the volume of literature encountered and limited resources, the research team collectively decided to focus interests on a reduced number of clinical outcomes for reasons of feasibility. Those that were originally of interest were set out to be stillbirth, fetal loss, gestational hypertension, pre-eclampsia, hypertensive disorders of pregnancy, eclampsia, pregnancy-induced hypertension, gestational diabetes, new onset diabetes, hospital admission, preterm birth, ICU admission, SGA, low birth weight, IUGR, maternal mortality and VTE. The collection of endpoints was subsequently reduced to those discussed in the review as presented.

Table S3: PRISMA Checklist

Section/topic	#	Checklist item	Reported on page #
**TITLE**			
Title	1	Identify the report as a systematic review, meta-analysis, or both.	1
**ABSTRACT**			
Structured summary	2	Provide a structured summary including, as applicable: background; objectives; data sources; study eligibility criteria, participants, and interventions; study appraisal and synthesis methods; results; limitations; conclusions and implications of key findings; systematic review registration number.	2
**INTRODUCTION**			
Rationale	3	Describe the rationale for the review in the context of what is already known.	3
Objectives	4	Provide an explicit statement of questions being addressed with reference to participants, interventions, comparisons, outcomes, and study design (PICOS).	4
**METHODS**			
Protocol and registration	5	Indicate if a review protocol exists, if and where it can be accessed (e.g., Web address), and, if available, provide registration information including registration number.	4
Eligibility criteria	6	Specify study characteristics (e.g., PICOS, length of follow-up) and report characteristics (e.g., years considered, language, publication status) used as criteria for eligibility, giving rationale.	4
Information sources	7	Describe all information sources (e.g., databases with dates of coverage, contact with study authors to identify additional studies) in the search and date last searched.	4
Search	8	Present full electronic search strategy for at least one database, including any limits used, such that it could be repeated.	44-62
Study selection	9	State the process for selecting studies (i.e., screening, eligibility, included in systematic review, and, if applicable, included in the meta-analysis).	5
Data collection process	10	Describe method of data extraction from reports (e.g., piloted forms, independently, in duplicate) and any processes for obtaining and confirming data from investigators.	6
Data items	11	List and define all variables for which data were sought (e.g., PICOS, funding sources) and any assumptions and simplifications made.	6-7

Section/topic	#	Checklist item	Reported on page #
Risk of bias in individual studies	12	Describe methods used for assessing risk of bias of individual studies (including specification of whether this was done at the study or outcome level), and how this information is to be used in any data synthesis.	69-147
Summary measures	13	State the principal summary measures (e.g., risk ratio, difference in means).	8
Synthesis of results	14	Describe the methods of handling data and combining results of studies, if done, including measures of consistency (e.g., I^2) for each meta-analysis.	8
Risk of bias across studies	15	Specify any assessment of risk of bias that may affect the cumulative evidence (e.g., publication bias, selective reporting within studies).	N/A
Additional analyses	16	Describe methods of additional analyses (e.g., sensitivity or subgroup analyses, meta-regression), if done, indicating which were pre-specified.	8-9
**RESULTS**			
Study selection	17	Give numbers of studies screened, assessed for eligibility, and included in the review, with reasons for exclusions at each stage, ideally with a flow diagram.	7
Study characteristics	18	For each study, present characteristics for which data were extracted (e.g., study size, PICOS, follow-up period) and provide the citations.	41-43
Risk of bias within studies	19	Present data on risk of bias of each study and, if available, any outcome level assessment (see item 12).	N/A
Results of individual studies	20	For all outcomes considered (benefits or harms), present, for each study: (a) simple summary data for each intervention group (b) effect estimates and confidence intervals, ideally with a forest plot.	9-17
Synthesis of results	21	Present results of each meta-analysis done, including confidence intervals and measures of consistency.	29-32
Risk of bias across studies	22	Present results of any assessment of risk of bias across studies (see Item 15).	N/A
Additional analysis	23	Give results of additional analyses, if done (e.g., sensitivity or subgroup analyses, meta-regression [see Item 16]).	N/A

Section/topic	#	Checklist item	Reported on page #
**DISCUSSION**			
Summary of evidence	24	Summarize the main findings including the strength of evidence for each main outcome; consider their relevance to key groups (e.g., healthcare providers, users, and policy makers).	17-18
Limitations	25	Discuss limitations at study and outcome level (e.g., risk of bias), and at review-level (e.g., incomplete retrieval of identified research, reporting bias).	19-20
Conclusions	26	Provide a general interpretation of the results in the context of other evidence, and implications for future research.	20-21
**FUNDING**			
Funding	27	Describe sources of funding for the systematic review and other support (e.g., supply of data); role of funders for the systematic review.	1
